# New generative methods for single-cell transcriptome data in bulk RNA sequence deconvolution

**DOI:** 10.1038/s41598-024-54798-z

**Published:** 2024-02-20

**Authors:** Toui Nishikawa, Masatoshi Lee, Masataka Amau

**Affiliations:** 1 Faculty of Medicine, Wakayama Medical University, 811-1 Kimiidera, Wakayama, 641-8509 Japan; 2 Faculty of Medicine, Kyoto University, Kyoto, Japan

**Keywords:** Single-cell RNA sequence, Bulk RNA sequence, Deconvolution, Augmentation, Generative AI, Computational biology and bioinformatics, Genome informatics, Machine learning

## Abstract

Numerous methods for bulk RNA sequence deconvolution have been developed to identify cellular targets of diseases by understanding the composition of cell types in disease-related tissues. However, issues of heterogeneity in gene expression between subjects and the shortage of reference single-cell RNA sequence data remain to achieve accurate bulk deconvolution. In our study, we investigated whether a new data generative method named sc-CMGAN and benchmarking generative methods (Copula, CTGAN and TVAE) could solve these issues and improve the bulk deconvolutions. We also evaluated the robustness of sc-CMGAN using three deconvolution methods and four public datasets. In almost all conditions, the generative methods contributed to improved deconvolution. Notably, sc-CMGAN outperformed the benchmarking methods and demonstrated higher robustness. This study is the first to examine the impact of data augmentation on bulk deconvolution. The new generative method, sc-CMGAN, is expected to become one of the powerful tools for the preprocessing of bulk deconvolution.

## Introduction

Recent advancements in single-cell RNA sequencing (scRNA-seq) have enabled the analysis of transcriptome profiles at the individual cell level. ScRNA-seq allows for the determination of cell type composition and ratios, which can facilitate the study of changes in tissue composition associated with diseases and the identification of disease-related cellular targets. For example, different types of infiltrating immune cells have different effects on tumor progression and the mass of A cells was increased in Type 2 diabetes^1,2^. However, the high cost and technical complexity of getting scRNA-seq data pose challenges when dealing with large sample populations^3,4^.

To overcome these challenges, several methods that estimate the proportion of cells from bulk RNA expression data without relying on single-cell sequencing has been attracted attention. This process, known as bulk RNA sequence deconvolution (bulk deconvolution), has seen the early development of statistical and computational methods^5–8^. More recently, methods utilizing scRNA-seq as a reference have achieved higher performance in deconvolution^9–11^. For instance, MuSiC (2019) demonstrated high performance, particularly in tissues with closely related cell types, and Bisque (2020) implemented a regression-based approach to learn gene-specific bulk expression transformations^9,10^. SCDC (2021) proved to be an effective method by leveraging cell type-specific gene expression profiles from multiple scRNA-seq reference datasets^11^.

Despite these advancements, there are several challenges associated with bulk deconvolution using scRNA-seq reference datasets. Firstly, there is heterogeneity in gene expression between subjects, which has been reported to reduce the performance of bulk deconvolution^4,9,12,13^. Secondly, achieving higher performance in bulk deconvolution requires more and higher-quality scRNA-seq data. However, as mentioned, the cost and availability of public scRNA-seq data make it difficult to secure sufficient data for analysis.

**Figure 1:**
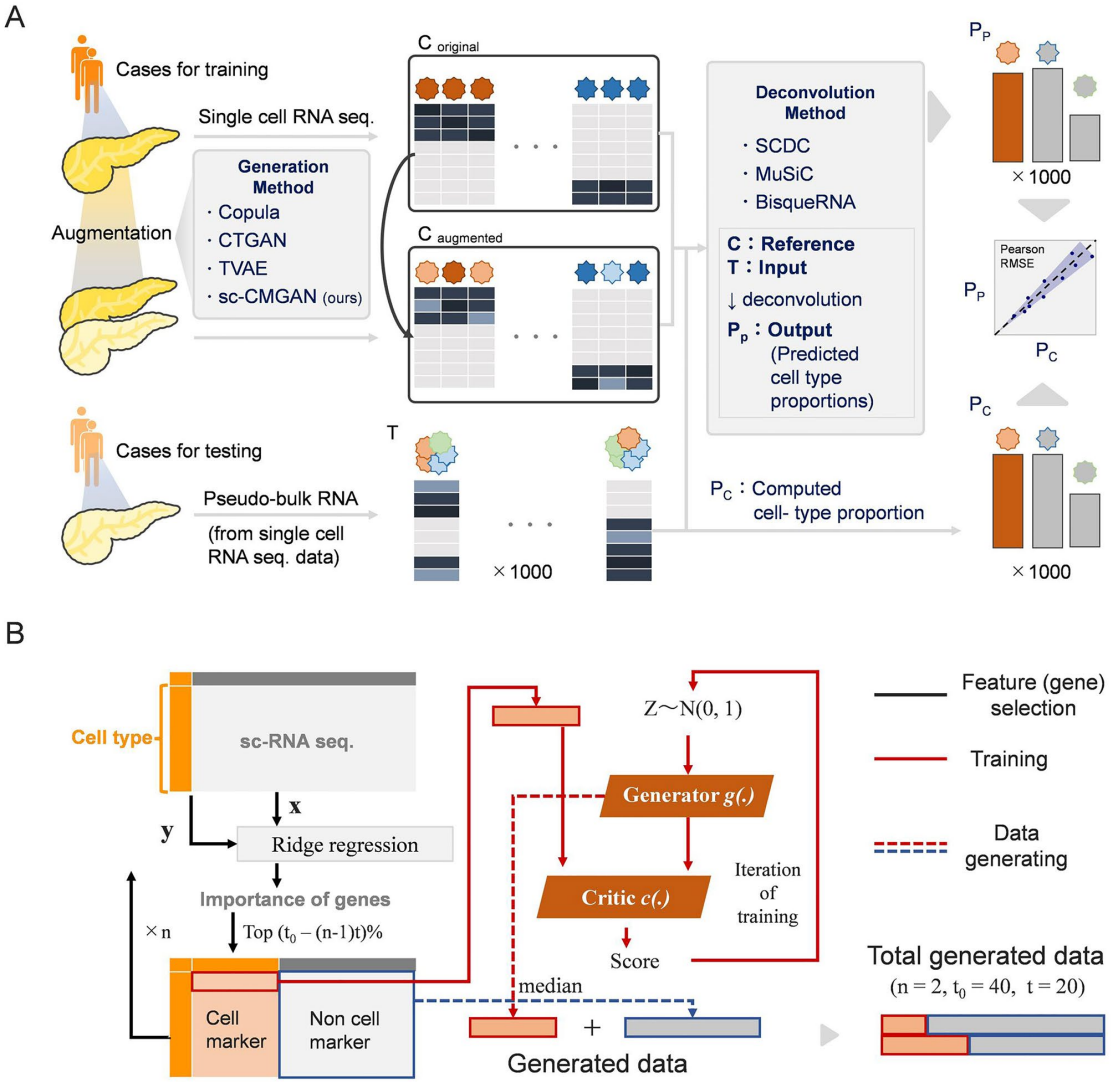
(**A**) Workflow of bulk deconvolution using data augmentation. Matrix C with augmentation is single cell RNA sequence data for reference data in deconvolution. Matrix T is the pseudo-bulk RNA sequence data, which is the input of the deconvolution methods. Finally, predicted cell type propotions and computed cell-type propotions are compared. RNA seq: RNA sequence; RMSE: root-mean-square error; Pearson: Pearson correlation value. (**B**) Summary of sc-CMGAN (Details are described in Method). In the feature selection step, top (t_0_ − t_n_) % of genes was selected as cell marker by ridge regression. sc-CMGAN: Generative Adversarial Network based on cell markers for single-cell genomics data, t_0_: initial value of percentage of cell markers in all genes; n: number of steps of gene selection.

In the study, we aimed to investigate whether the performance of bulk deconvolution could be improved by augmenting scRNA-seq data using benchmarking generative methods (Fig. 1A). Additionally, we developed a new generative method based on a stepwise selection of cell markers called sc-CMGAN (stepwise Generative Adversarial Network based on cell markers for single-cell genomics data) (Fig. 1B).

## Results

### Impact of data augmentation on deconvolution results

**Figure 2:**
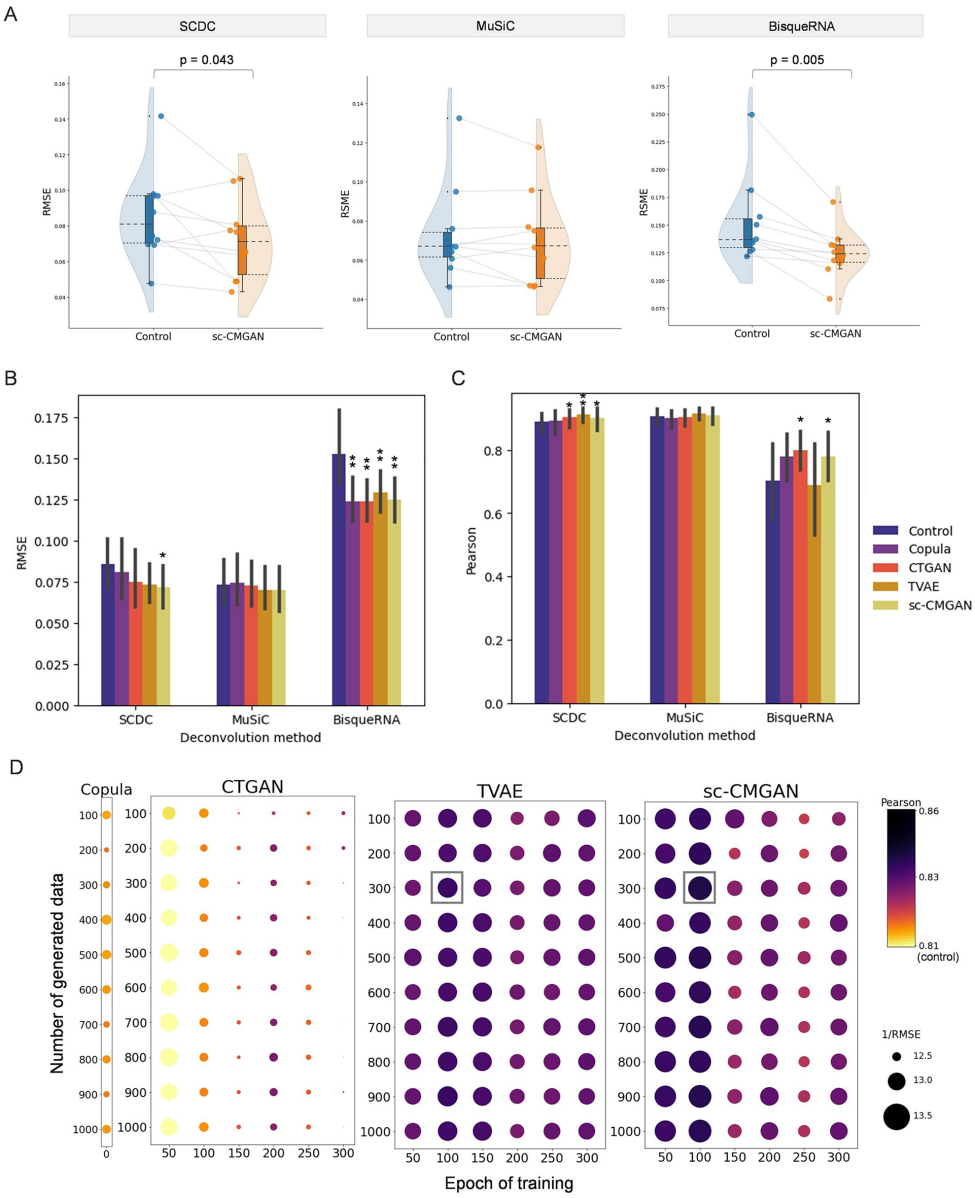
Results of bulk RNA seq. deconvolution using data augmentation. (**A**) Comparison of bulk RNA sequencing deconvolution results with and without sc-CMGAN, represented by root-mean-square error (RMSE) values. (**B**–**C**) Comparison of sc-CMGAN with four data generative methods for bulk deconvolution. (**D**) Relationship between hyperparameters and improvement of bulk deconvolution. Larger circles and darker colors indicate greater improvement. SCDC, which had the best compatibility with scCMGAN was used as deconvolution method. RMSE: root-mean-square error; Pearson: Pearson correlation value.

The results of our study demonstrate the positive impact of sc-CMGAN data augmentation on bulk deconvolution performance (see Table 1 for information about the each scRNA-seq dataset). We focused on Baron's dataset and observed consistent improvements in performance, as measured by the Pearson coefficient and RMSE, across all three deconvolution methods (SCDC, MuSiC, and BisqueRNA) (Fig. 2A). Specifically, significant improvements in deconvolution were observed for SCDC (*p* = 0.043) and BisqueRNA (*p* = 0.005) when using sc-CMGAN data augmentation. We further compared the performance of sc-CMGAN with other benchmarking methods in terms of their impact on the three deconvolution methods (Fig. 2B,C and Table 2). For SCDC, significant improvements were observed only when using sc-CMGAN. For MuSiC, the Copula and CTGAN generative methods led to a decrease of performance, while TVAE and sc-CMGAN showed improvements. In the case of BisqueRNA, all generative methods significantly improved the performance compared to the control (Copula: *p* = 0.003, CTGAN: *p* = 0.003, TVAE: *p* = 0.025, sc-CMGAN: *p* = 0.005).

**Table 1:** Summary of datasets.

Name of datasets (in the paper)	Sample type	Number of samples	Number of cell types	Number of cells for training	Number of cells for testing
Baron	Pancreas	4 (2 for train, 2 for test)	10	3614	4892
GSE81547	Pancreas	8 (4 for train, 4 for test)	5	1159	1385
Kidney.HCL	Kidney	2 (For train and test)	8	2209	3639
PBMCs*	Peripheral blood	1 (For train and test)	6	2340	2343

**Table 2:** RMSE and Pearson correlation values between the computed (known) proportions in 1000 pseudo-bulk RNA-seq. data and the predicted proportions from the different bulk deconvolution methods using different generative methods.

Deconvolution method	Generative method	RMSE			Pearson correlation		
		Value		*p*	Value		*p*
SCDC	Control	0.089	–	–	0.823	–	–
	Copula	0.085	(− 0.003)	0.373	0.826	(+ 0.003)	0.700
	CTGAN	0.080	(− 0.009)	0.074	0.843	(+ 0.021)	0.024*
	TVAE	0.076	(− 0.013)	0.154	0.851	(+ 0.028)	0.002**
	Sc-CMGAN	0.075	(− 0.014)	0.043*	0.854	(+ 0.031)	0.047*
MuSiC	Control	0.077	−	−	0.859	−	−
	Colupa	0.079	(+ 0.002)	0.479	0.849	(− 0.010)	0.083
	CTGAN	0.076	(− 0.001)	0.696	0.858	(− 0.001)	0.342
	TVAE	0.073	(− 0.004)	0.419	0.866	(+ 0.006)	0.149
	Sc-CMGAN	0.074	(− 0.004)	0.259	0.862	(+ 0.003)	0.326
BisqueRNA	Control	0.157	−	−	0.287	−	−
	Colupa	0.126	(− 0.031)	0.003**	0.503	(+ 0.216)	0.085
	CTGAN	0.125	(− 0.031)	0.003**	0.050	(− 0.237)	0.029*
	TVAE	0.131	(− 0.026)	0.025**	0.458	(+ 0.171)	0.852
	Sc-CMGAN	0.127	(− 0.030)	0.005**	0.493	(+ 0.206)	0.038*

*p < 0.05, **p < 0.01.

### Relationship between hyperparameter and performance

Figure 2D presents the relationship between the combination of epoch and the number of generated data and their impact on deconvolution, specifically for the SCDC method using the Baron dataset. It was observed that the performance of deconvolution was most improved when the epoch was set to 100 and the number of generated data was 100. In terms of the effect of epoch on performance, it was found to have a smaller impact on performance in sc-CMGAN compared to CTGAN. When the number of generated data was 300 cells/cell type and the deconvolution method used was sc-CMGAN, the Pearson correlation values were as follows: 0.851 in 50 epochs, 0.854 in 100 epochs, 0.841 in 150 epochs, 0.845 in 200 epochs, 0.838 in 250 epochs, and 0.845 in 300 epochs. The best value and worst value had a difference of 0.016. On the other hand, when the number of generated data was 300 cells/cell type and the deconvolution method was CTGAN, the Pearson correlation values were as follows: 0.809 in 50 epochs, 0.822 in 100 epochs, 0.830 in 150 epochs, 0.840 in 200 epochs, 0. 829 in 250 epochs and 0.834 in 300 epochs. The difference between the best value and worst value was 0.031.

### Evaluation at the cell type level

**Figure 3:**
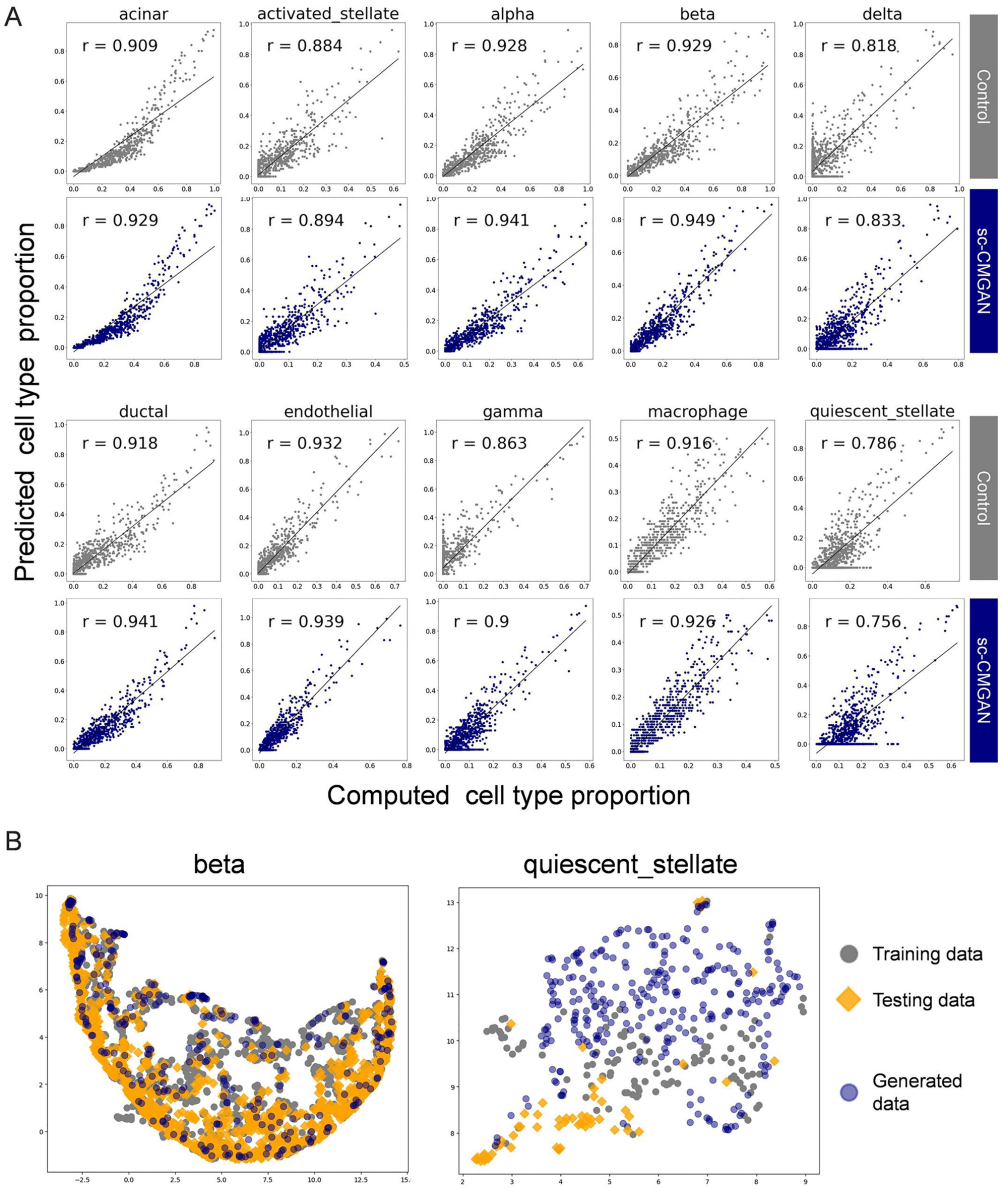
(**A**) Correlation between the computed (known) and predicted cell type proportions in 1000 pseudo-bulk RNA-seq. data, organized by cell types. Pearson correlation values (r) are improved in almost all cell types. (**B**) Scatter plot visualization of the training, testing, and generated data using UMAP. This compares the generated data for cells that showed much improvement and cells that showed a decrease in performance.

The impact of data generation on bulk deconvolution was further analyzed at the cell type level, specifically for the SCDC method using the Baron dataset. The results showed that performance was improved for almost all cell types, with the exception of quiescent stellate cells (Fig. 3A, Table 3). The ratio of RMSE improvement to the RMSE of the control (without data generation) was calculated for each cell type. Among all cell types, beta cells exhibited the highest improvement ratio (+ 0.50), indicating a substantial enhancement of performance. On the other hand, quiescent stellate cells showed a negative improvement ratio (− 0.08), suggesting a decrease of performance with data generation. To provide a visual representation of the training, testing, and generated data in beta cells and quiescent stellate cells, UMAP plots were created (Fig. 3B).

**Table 3:** RMSE and Pearson correlation values by cell types using different generative methods (bulk deconvolution method was SCDC).

Evaluation value	RMSE					Pearson correlation				
Generative method	Control	Copula	CTGAN	TVAE	sc-CMGAN	Control	Copula	CTGAN	TVAE	sc-CMGAN
Acinar	0.141	0.446	0.146	0.096	0.107	0.909	0.916	0.908	0.926	**0.929**
Activated stellate	0.069	0.088	0.072	0.066	0.078	0.884	0.872	0.896	**0.895**	0.894
Alpha	0.074	0.063	0.064	0.062	0.049	0.928	0.934	0.933	0.931	**0.941**
Beta	0.098	0.065	0.047	0.045	0.049	0.929	0.944	0.951	**0.953**	0.949
Delta	0.088	0.075	0.077	0.072	0.081	0.818	0.857	0.862	**0.863**	0.833
Ductal	0.070	0.066	0.063	0.106	0.064	0.918	0.920	0.923	0.938	**0.941**
Endothelial	0.072	0.066	0.072	0.063	0.066	0.932	0.929	0.931	**0.943**	0.939
Gamma	0.097	0.086	0.042	0.085	0.077	0.863	0.903	**0.905**	0.901	0.900
Macrophage	0.048	0.044	0.102	0.047	0.043	0.916	0.920	**0.927**	0.923	0.926
Quiescent stellate	0.097	0.117	0.102	0.092	0.105	0.786	0.723	0.790	**0.834**	0.756

The best values of each cell type are in bold.

### Deconvolution in other datasets

**Figure 4:**
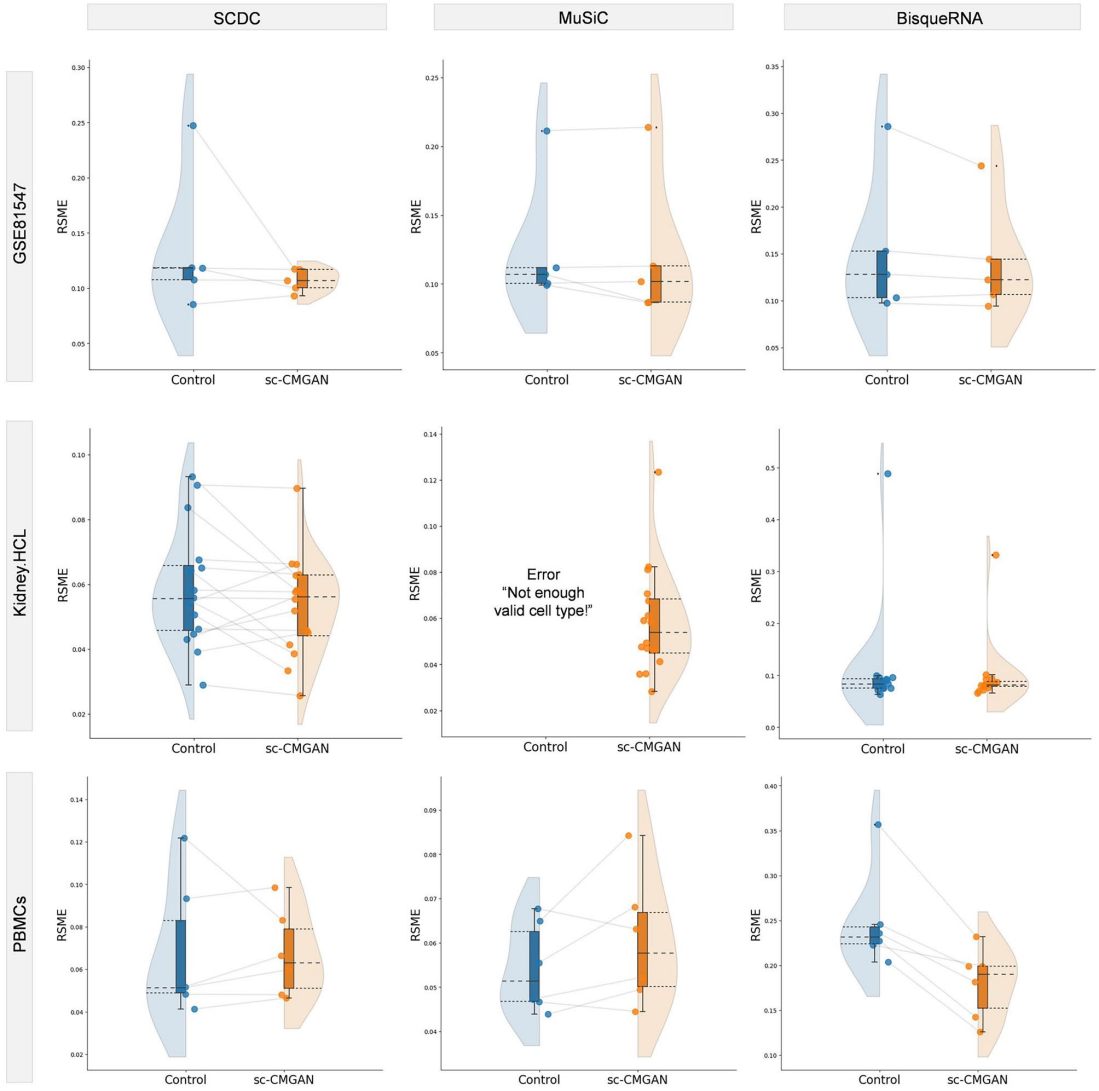
Comparison between bulk deconvolution without sc-CMGAN (Control) and with sc-CMGAN in the datasets of Kidney HCL., PBMCs, and GSE81547. The results showed improvements in Pearson correlation values for most conditions, except for the analysis of PBMCs data using the MuSiC method.

The impact of sc-CMGAN on bulk deconvolution was also examined in other datasets, namely GSE81547, Kidney HCL, and PBMCs datasets (Fig. 4, Table 4). The results showed improvements in Pearson correlation values for most conditions, except for the analysis of PBMCs data using the MuSiC method. In the analysis of Kidney HCL and PBMCs datasets using the BisqueRNA method, sc-CMGAN significantly improved the RMSE (Kidney HCL: *p* = 0.039, PBMCs: *p* = 0.005). Furthermore, MuSiC analysis of the Kidney HCL dataset initially encountered the “Not enough valid cell type” error, however, data augmentation using sc-CMGAN allowed for error-free deconvolution in this dataset. All the detailed results, including the best epoch and cell numbers from this experiment, can be found in the supplementary data.

**Table 4:** RMSE and Pearson correlation values from the datasets of GSE81547, PBMCs and Kidney HCL.

Datasets	Deconvolution method	Generative method	RMSE			Pearson correlation		
			Value		*p*	Value		*p*
GSE81547	SCDC	Control	0.135	− 0.025	–	0.732	0.065	–
		sc-CMGAN	0.110		0.332	0.797		0.566
	MuSiC	Control	0.128	0.001	–	0.760	0.003	–
		sc-CMGAN	0.130		0.288	0.763		0.530
	BisqueRNA	Control	0.162	− 0.015	0.227	0.423	0.050	–
		sc-CMGAN	0.147			0.473		0.435
Kidney HCL	SCDC	Control	0.061	− 0.006	–	0.833	0.028	–
		sc-CMGAN	0.056		0.141	0.861		0.493
	MuSiC	Control	error	–	–	error	–	–
		sc-CMGAN	0.063		–	0.801		–
	BisqueRNA	Control	0.147	− 0.032	**0.039**	0.255	0.082	
		sc-CMGAN	0.115			0.337		0.271
PBMCs	SCDC	Control	0.074	− 0.004	–	0.937	0.015	–
		sc-CMGAN	0.070		0.919	0.951		**0.007**
	MuSiC	Control	0.055	0.007	–	0.966	− 0.008	–
		sc-CMGAN	0.062		0.165	0.958		0.058
	BisqueRNA	Control	0.075	0.109	–	0.254	0.244	–
		sc-CMGAN	0.184		**0.005**	0.498		0.084

Significant values are in bold.

### Deconvolution in real bulk RNA sequence dataset

**Figure 5:**
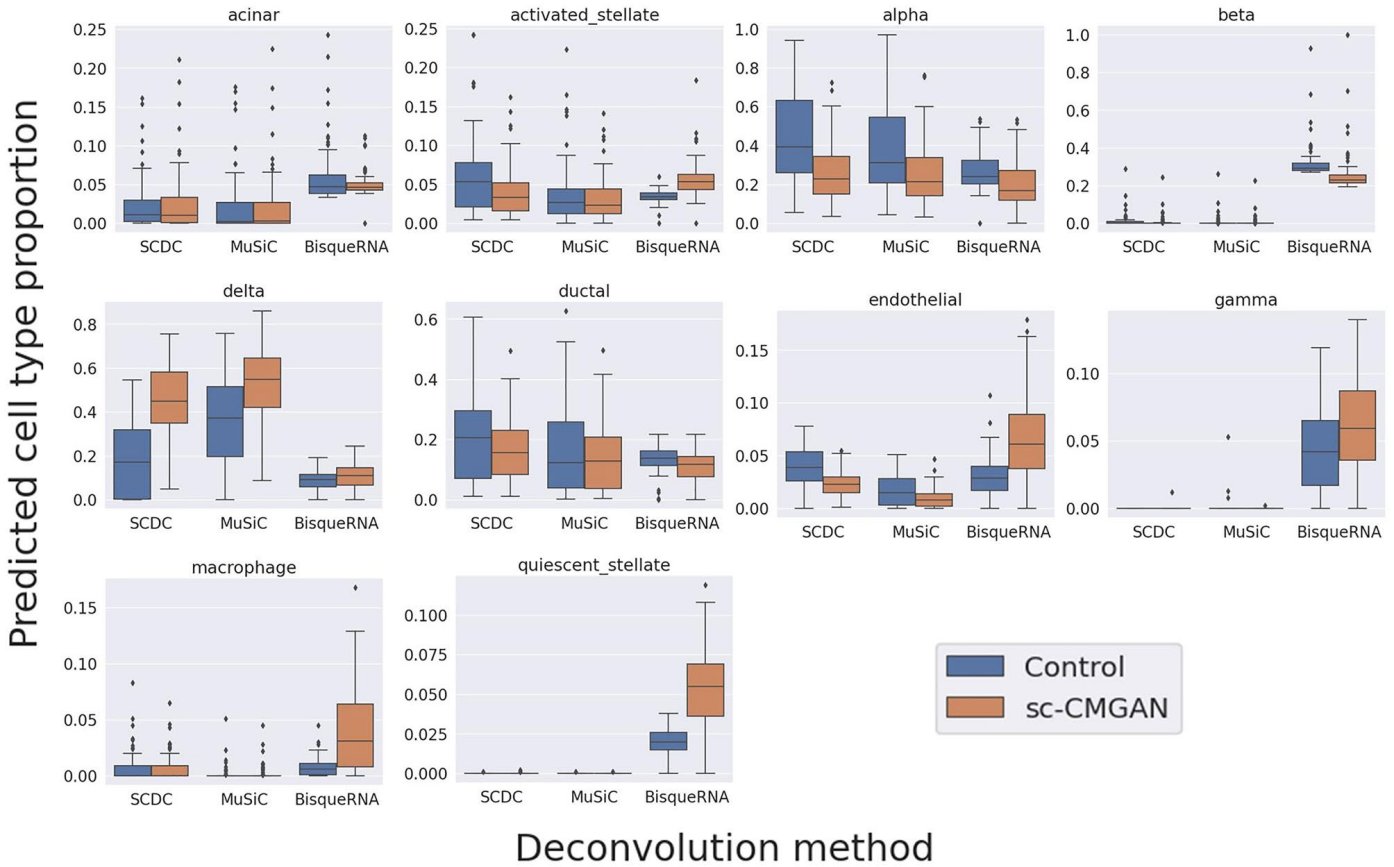
Bulk deconvolution in Fadista’s real bulk RNA sequence data using Baron’s dataset as a reference. Due to the augmentation strategy using sc-CMGAN, the overestimated proportion of alpha cells was relatively underestimated.

Using Baron's dataset as a reference, we performed bulk deconvolution in real bulk RNA sequence data (Fig. 5). As also shown in the study by Wang et al., deconvolution methods overestimated the proportion of α cells^9^. However, all deconvolution methods with augmentation underestimated the proportion of α cells, compared to the deconvolution without augmentation.

## Discussion

Bulk deconvolution is a valuable approach for estimating cell type proportions from bulk RNA-seq data, providing a cost-effective alternative to scRNA-seq. In the study, we attempted to improve the performance of bulk deconvolution using data generative methods. While efforts have been made to generate scRNAseq. In silico, the impact of data augmentation on deconvolution remains uncertain^14^. Additionally, we developed a new stepwise generative method (sc-CMGAN), and its performance was compared the performance of sc-CMGAN with that of benchmarking generative methods.

The results demonstrated that data augmentation using sc-CMGAN consistently improved the performance of all tested bulk deconvolution methods in the Baron’s dataset. While other benchmarking generative methods also led to improvements, sc-CMGAN exhibited two key advantages. Firstly, sc-CMGAN displayed the highest robustness across different deconvolution methods. Notably, significant improvements were observed with SCDC only when using sc-CMGAN. In MuSiC, the performance with TVAE was slightly better than sc-CMGAN, but in BisqueRNA, TVAE showed less improvement compared to the other three methods. Secondly, sc-CMGAN exhibited high stability and improvement regardless of the training epoch, unlike CTGAN, which showed significant performance variations with different epochs. This stability can be attributed to the stepwise strategy employed by sc-CMGAN.

These deconvolution improvements were found to be logically appropriate by two analyses at the cell type level (Fig. 3, Table 3). In the analysis of performance of each cell type, most cell types showed improved performance without bias, which is consistent with comprehensive data augmentation for cell types. In the analysis of the relationship improvement and visualization using UMAP, the highest improvement was seen in beta cells, of which distribution of the generated data was similar to the distribution of test data. Only quiescent stellate cells didn’t showed improvement, but this could be improved by adjusting the epochs for each cell. The two analyses showed that the augmentation strategy had the potential to partially mitigate the heterogeneity in gene expression between subjects and improve the bulk deconvolution.

Furthermore, the study extended its evaluation to other datasets (GSE81547, Kidney HCL, and PBMCs) (Fig. 4, Table 4). Pearson correlation values improved in almost all conditions, with the exception of the analysis of PBMCs data using MuSiC. This improvement was observed in the Baron dataset and GSE81547 with the inter-case variation, as well as in Kidney HCL and PBMCs datasets with the intra-case variation. These findings indicate that sc-CMGAN is effective in addressing both types of variation encountered in bulk deconvolution.

We have two limitations of the study. First, the study is lacking real RNA sequence data analysis. The main purpose of the study is to investigate the influence of augmentation strategy on bulk deconvolution, so we designed the study using only pseudo-RNA sequence data. Our method has a potential to investigate the interesting biology if we deconvolute a real bulk RNA-seq data. Second, it is better to include many results that quantify this improvement under different tissues and conditions. Then, we selected tissues to avoid overlap (kidney, pancreas, peripheral blood cells) and the results that cannot be included in the main text is included in the supplementary data. Further extension of the conditions will make the results of improvements more reliable.

In conclusion, our study demonstrated that both the benchmarking and new generative methods improved the performance of bulk deconvolution. Specifically, our newly developed sc-CMGAN method outperformed the benchmarking methods in enhancing the performance of bulk deconvolution. The sc-CMGAN method, accompanied by its dedicated library and software, shows promising potential to become one of the powerful tools for the preprocessing in bulk deconvolution.

## Materials and methods

### Datasets

The primary dataset used in this study was the pancreatic single-cell transcriptome data from Baron et al., which is widely used in bulk deconvolution^15^. Additionally, we examined other datasets of pancreatic, renal, and peripheral blood single-cell transcriptome data to assess the robustness of our approach (refer to Table 1 for details on the scRNA-seq datasets)^16–19^, and Fadista’s dataset to perform bulk deconvolution in real bulk RNA sequence data^20^. Selection of the primary dataset was based on the criteria of having the highest number of cell types and two cases at least available.

### Pre-processing

In the pre-processing step, we followed the approach described by Cobos et al.^13^ Initially, we removed rows corresponding to genes with zero expression or zero variability. Next, the cells with library size, mitochondrial content or ribosomal content further than three median absolute deviations away were discarded. Subsequently, we retained only those genes that were present in at least 5% of all cells, regardless of cell type, and had a UMI or read count greater than one. TMM normalization was applied to the final scRNA-seq expression dataset^21^.

### Generation of artificial pseudo-bulk mixtures

The deconvolution pipeline, as depicted in Fig. 1A, was implemented in this study. Following the pre-processing step, the dataset was divided into equal proportions of 50% for training data and 50% for testing data. Subsequently, using the testing data, a matrix (referred to as matrix T) comprising 1000 pseudo-bulk mixtures was generated. This involved summing the count values from randomly selected individual cells. For each dataset, the minimum number of cells utilized to construct the pseudo-bulk mixture was set at 100.

### Data augmentation

The training data were augmented by benchmarking or new generative methods. In the study, we employed Gaussian Copula (Copula), Conditional Tabular GAN (CTAGAN) and Triplet-based Variational Autoencoder (TVAE) as benchmarking generative methods^22,23^. Additionally, we developed and tested a new generative method specifically designed for scRNA-seq data. A grid search was performed to investigate optimal data generative conditions (number of images generated and training epoch). Specifically, we varied the number of generated data by increments of 100 cells, ranging from 100 cells per cell type to 1000 cells per cell type. Furthermore, we explored different training epochs, testing values from 50 to 300 epochs with increments of 50 epochs. The augmented data was used as a new independent reference case and added to the matrix C for the bulk deconvolution.

### New generative method

A new data generative method called sc-CMGAN (stepwise Generative adversarial network based on cell markers for single-cell genomics data) was developed (Fig. 1B). The sc-CMGAN approach consists of three main steps: feature selection, training, and generating. In the feature selection step, a set of cell marker genes was identified using ridge regression. These genes serve as key indicators of cell types in the sc-RNA seq. The absolute value of the coefficient in ridge regression was taken as the importance of the genes. Next, in the training step, the sc-RNA seq. data corresponding to the selected cell marker genes were used to train the GAN model (CTGAN). This step involved the learning and capturing of the underlying data distribution of the cell marker genes. In the generating step, the trained models were used to generate sc-RNA seq. data for the cell marker genes. Simultaneously, the non-cell marker genes were assigned the median value of the expression data for the respective cell type. This process was repeated for a specific number of cycles (= n), and the generated data sets from each cycle were combined. To control the selection of cell markers, the top (t_0_ − t_n_) percentage of genes were chosen, where t_0_ represents the initial value of the percentage of cell markers in all genes. In this study, the hyperparameters were set as (n, t_0_, t) = (2, 40, 20). The sc-CMGAN code, library and software can be obtained from the following GitHub link: https://github.com/TouiNishikawa/scCMGAN.

### Bulk deconvolution method

The bulk deconvolution method was employed to estimate the cell type proportions from the artificial pseudo-bulk mixtures (matrix T) and the augmented reference scRNA-seq data (matrix C). Three benchmarking deconvolution methods, namely SCDC, MuSiC, and BisqueRNA, were utilized for the bulk deconvolution analysis^9–11^. The bulk deconvolution process was implemented in the R environment (version 3.6.1.).

### Deconvolution in real bulk RNA sequence dataset

#### Evaluation and visualization of results

Both the Pearson correlation coefficient and root mean square error (RMSE) were calculated to evaluate the performance of different deconvolution methods. The Pearson correlation coefficient measures the linear relationship between the estimated cell type proportions and the true proportions from the pseudo-bulk mixtures. A higher Pearson correlation values indicates better agreement between the estimated and true proportions. The RMSE quantifies the difference between the estimated and true proportions, with lower values indicating better performance. Furthermore, the generated data were visualized using the Uniform Manifold Approximation and Projection (UMAP) technique. UMAP is a dimensionality reduction algorithm that can provide a low-dimensional representation of high-dimensional data, allowing for visualization and clustering of the generated data^24^.

### Statistical analysis

To assess the significance of the improvement achieved through data augmentation, a paired t-test was conducted. This statistical test compared the Pearson correlation values and RMSE between the results obtained with and without data augmentation. The paired t-test was performed in a Python environment, using appropriate statistical packages.

### Supplementary Information

Supplementary Information.

## Data Availability

The four publicly available datasets used in the study can be found at: https://www.ncbi.nlm.nih.gov/geo/query/acc.cgi?acc=GSE84133 (baron), https://www.ncbi.nlm.nih.gov/geo/query/acc.cgi?acc=GSE81547 (GSE81547), https://support.10xgenomics.com/single-cell-gene-expression/datasets/1.1.0/fresh_68k_pbmc_donor_a (PBMCs), https://figshare.com/articles/HCL_DGE_Data/7235471 (kidney.HCL).

## Code availability

Source code can be found at https://github.com/TouiNishikawa/scCMGAN.
