# Supplementary material for: New generative methods for single-cell transcriptome data in bulk RNA sequence deconvolution
Source: Sci Rep. 2024 Feb 20;14:4156. doi: 10.1038/s41598-024-54798-z (PMC10879528; doi:10.1038/s41598-024-54798-z)
Supplement: Supplementary file 1 — Supplementary Information. [file 41598_2024_54798_MOESM1_ESM.pdf]

Dataset	Deconvolu	Augmenta	Cell type	Number o	RSME	Pearson	RSME	Pearson	RSME	Pearson	RSME	Pearson	RSME	Pearson	RSME	Pearson	RSME	Pearson
baron	SCDC	Control	acinar	0	0.141689	0.90867	-	-	-	-	-	-	-	-	-	-	-	-
baron	SCDC	Control	activated_	0	0.06933	0.884062	-	-	-	-	-	-	-	-	-	-	-	-
baron	SCDC	Control	alpha	0	0.074455	0.928009	-	-	-	-	-	-	-	-	-	-	-	-
baron	SCDC	Control	beta	0	0.097975	0.92871	-	-	-	-	-	-	-	-	-	-	-	-
baron	SCDC	Control	delta	0	0.087712	0.818301	-	-	-	-	-	-	-	-	-	-	-	-
baron	SCDC	Control	ductal	0	0.069904	0.918427	-	-	-	-	-	-	-	-	-	-	-	-
baron	SCDC	Control	endothelia	0	0.072216	0.932422	-	-	-	-	-	-	-	-	-	-	-	-
baron	SCDC	Control	gamma	0	0.097033	0.863098	-	-	-	-	-	-	-	-	-	-	-	-
baron	SCDC	Control	macropha	0	0.047813	0.91575	-	-	-	-	-	-	-	-	-	-	-	-
baron	SCDC	Control	quiescent_	0	0.096913	0.785715	-	-	-	-	-	-	-	-	-	-	-	-
baron	SCDC	Copula	acinar	100	0.138737	0.916973	-	-	-	-	-	-	-	-	-	-	-	-
baron	SCDC	Copula	activated_	100	0.08856	0.873521	-	-	-	-	-	-	-	-	-	-	-	-
baron	SCDC	Copula	alpha	100	0.059879	0.933744	-	-	-	-	-	-	-	-	-	-	-	-
baron	SCDC	Copula	beta	100	0.075634	0.940581	-	-	-	-	-	-	-	-	-	-	-	-
baron	SCDC	Copula	delta	100	0.079179	0.836424	-	-	-	-	-	-	-	-	-	-	-	-
baron	SCDC	Copula	ductal	100	0.065898	0.918916	-	-	-	-	-	-	-	-	-	-	-	-
baron	SCDC	Copula	endothelia	100	0.068204	0.913674	-	-	-	-	-	-	-	-	-	-	-	-
baron	SCDC	Copula	gamma	100	0.093514	0.889871	-	-	-	-	-	-	-	-	-	-	-	-
baron	SCDC	Copula	macropha	100	0.043794	0.92044	-	-	-	-	-	-	-	-	-	-	-	-
baron	SCDC	Copula	quiescent_	100	0.114799	0.716443	-	-	-	-	-	-	-	-	-	-	-	-
baron	SCDC	Copula	acinar	200	0.141009	0.916442	-	-	-	-	-	-	-	-	-	-	-	-
baron	SCDC	Copula	activated_	200	0.087797	0.872024	-	-	-	-	-	-	-	-	-	-	-	-
baron	SCDC	Copula	alpha	200	0.063115	0.934137	-	-	-	-	-	-	-	-	-	-	-	-
baron	SCDC	Copula	beta	200	0.065073	0.94418	-	-	-	-	-	-	-	-	-	-	-	-
baron	SCDC	Copula	delta	200	0.074681	0.857386	-	-	-	-	-	-	-	-	-	-	-	-
baron	SCDC	Copula	ductal	200	0.065296	0.920143	-	-	-	-	-	-	-	-	-	-	-	-
baron	SCDC	Copula	endothelia	200	0.066356	0.928851	-	-	-	-	-	-	-	-	-	-	-	-
baron	SCDC	Copula	gamma	200	0.08582	0.903253	-	-	-	-	-	-	-	-	-	-	-	-
baron	SCDC	Copula	macropha	200	0.043658	0.920487	-	-	-	-	-	-	-	-	-	-	-	-
baron	SCDC	Copula	quiescent_	200	0.116796	0.722655	-	-	-	-	-	-	-	-	-	-	-	-
baron	SCDC	Copula	acinar	300	0.143764	0.914466	-	-	-	-	-	-	-	-	-	-	-	-
baron	SCDC	Copula	activated_	300	0.087491	0.873874	-	-	-	-	-	-	-	-	-	-	-	-
baron	SCDC	Copula	alpha	300	0.061132	0.934032	-	-	-	-	-	-	-	-	-	-	-	-
baron	SCDC	Copula	beta	300	0.065357	0.943927	-	-	-	-	-	-	-	-	-	-	-	-
baron	SCDC	Copula	delta	300	0.07569	0.851319	-	-	-	-	-	-	-	-	-	-	-	-

baron	SCDC	Copula	ductal	300	0.068285	0.915416	-	-	-	-	-	-	-	-	-	-	-	-
baron	SCDC	Copula	endothelia	300	0.068234	0.921989	-	-	-	-	-	-	-	-	-	-	-	-
baron	SCDC	Copula	gamma	300	0.089332	0.900852	-	-	-	-	-	-	-	-	-	-	-	-
baron	SCDC	Copula	macropha	300	0.043664	0.921067	-	-	-	-	-	-	-	-	-	-	-	-
baron	SCDC	Copula	quiescent_	300	0.115125	0.724639	-	-	-	-	-	-	-	-	-	-	-	-
baron	SCDC	Copula	acinar	400	0.143438	0.913857	-	-	-	-	-	-	-	-	-	-	-	-
baron	SCDC	Copula	activated_	400	0.089596	0.870709	-	-	-	-	-	-	-	-	-	-	-	-
baron	SCDC	Copula	alpha	400	0.06252	0.934098	-	-	-	-	-	-	-	-	-	-	-	-
baron	SCDC	Copula	beta	400	0.071808	0.94225	-	-	-	-	-	-	-	-	-	-	-	-
baron	SCDC	Copula	delta	400	0.0775	0.845548	-	-	-	-	-	-	-	-	-	-	-	-
baron	SCDC	Copula	ductal	400	0.066636	0.917725	-	-	-	-	-	-	-	-	-	-	-	-
baron	SCDC	Copula	endothelia	400	0.067011	0.924159	-	-	-	-	-	-	-	-	-	-	-	-
baron	SCDC	Copula	gamma	400	0.090189	0.896741	-	-	-	-	-	-	-	-	-	-	-	-
baron	SCDC	Copula	macropha	400	0.04378	0.919928	-	-	-	-	-	-	-	-	-	-	-	-
baron	SCDC	Copula	quiescent_	400	0.117636	0.713163	-	-	-	-	-	-	-	-	-	-	-	-
baron	SCDC	Copula	acinar	500	0.139888	0.916302	-	-	-	-	-	-	-	-	-	-	-	-
baron	SCDC	Copula	activated_	500	0.089986	0.87113	-	-	-	-	-	-	-	-	-	-	-	-
baron	SCDC	Copula	alpha	500	0.062186	0.93396	-	-	-	-	-	-	-	-	-	-	-	-
baron	SCDC	Copula	beta	500	0.072305	0.942013	-	-	-	-	-	-	-	-	-	-	-	-
baron	SCDC	Copula	delta	500	0.077336	0.846143	-	-	-	-	-	-	-	-	-	-	-	-
baron	SCDC	Copula	ductal	500	0.066851	0.920193	-	-	-	-	-	-	-	-	-	-	-	-
baron	SCDC	Copula	endothelia	500	0.067836	0.922742	-	-	-	-	-	-	-	-	-	-	-	-
baron	SCDC	Copula	gamma	500	0.090167	0.89755	-	-	-	-	-	-	-	-	-	-	-	-
baron	SCDC	Copula	macropha	500	0.04357	0.921141	-	-	-	-	-	-	-	-	-	-	-	-
baron	SCDC	Copula	quiescent_	500	0.11918	0.709927	-	-	-	-	-	-	-	-	-	-	-	-
baron	SCDC	Copula	acinar	600	0.142323	0.915405	-	-	-	-	-	-	-	-	-	-	-	-
baron	SCDC	Copula	activated_	600	0.089251	0.870508	-	-	-	-	-	-	-	-	-	-	-	-
baron	SCDC	Copula	alpha	600	0.059518	0.934907	-	-	-	-	-	-	-	-	-	-	-	-
baron	SCDC	Copula	beta	600	0.072169	0.941815	-	-	-	-	-	-	-	-	-	-	-	-
baron	SCDC	Copula	delta	600	0.076899	0.846903	-	-	-	-	-	-	-	-	-	-	-	-
baron	SCDC	Copula	ductal	600	0.068038	0.917803	-	-	-	-	-	-	-	-	-	-	-	-
baron	SCDC	Copula	endothelia	600	0.066645	0.926402	-	-	-	-	-	-	-	-	-	-	-	-
baron	SCDC	Copula	gamma	600	0.089787	0.897097	-	-	-	-	-	-	-	-	-	-	-	-
baron	SCDC	Copula	macropha	600	0.043795	0.919996	-	-	-	-	-	-	-	-	-	-	-	-
baron	SCDC	Copula	quiescent_	600	0.116415	0.717021	-	-	-	-	-	-	-	-	-	-	-	-
baron	SCDC	Copula	acinar	700	0.13992	0.916481	-	-	-	-	-	-	-	-	-	-	-	-

baron	SCDC	Copula	activated_	700	0.089263	0.871676	-	-	-	-	-	-	-	-	-	-	-	-
baron	SCDC	Copula	alpha	700	0.061019	0.934617	-	-	-	-	-	-	-	-	-	-	-	-
baron	SCDC	Copula	beta	700	0.070259	0.942954	-	-	-	-	-	-	-	-	-	-	-	-
baron	SCDC	Copula	delta	700	0.076365	0.850204	-	-	-	-	-	-	-	-	-	-	-	-
baron	SCDC	Copula	ductal	700	0.066595	0.920248	-	-	-	-	-	-	-	-	-	-	-	-
baron	SCDC	Copula	endothelia	700	0.065752	0.926399	-	-	-	-	-	-	-	-	-	-	-	-
baron	SCDC	Copula	gamma	700	0.087832	0.901718	-	-	-	-	-	-	-	-	-	-	-	-
baron	SCDC	Copula	macropha	700	0.043609	0.920438	-	-	-	-	-	-	-	-	-	-	-	-
baron	SCDC	Copula	quiescent_	700	0.116701	0.718078	-	-	-	-	-	-	-	-	-	-	-	-
baron	SCDC	Copula	acinar	800	0.140658	0.915879	-	-	-	-	-	-	-	-	-	-	-	-
baron	SCDC	Copula	activated_	800	0.088935	0.872332	-	-	-	-	-	-	-	-	-	-	-	-
baron	SCDC	Copula	alpha	800	0.061461	0.934558	-	-	-	-	-	-	-	-	-	-	-	-
baron	SCDC	Copula	beta	800	0.071586	0.941653	-	-	-	-	-	-	-	-	-	-	-	-
baron	SCDC	Copula	delta	800	0.07651	0.849778	-	-	-	-	-	-	-	-	-	-	-	-
baron	SCDC	Copula	ductal	800	0.066476	0.918896	-	-	-	-	-	-	-	-	-	-	-	-
baron	SCDC	Copula	endothelia	800	0.067179	0.922342	-	-	-	-	-	-	-	-	-	-	-	-
baron	SCDC	Copula	gamma	800	0.089171	0.899484	-	-	-	-	-	-	-	-	-	-	-	-
baron	SCDC	Copula	macropha	800	0.043772	0.920084	-	-	-	-	-	-	-	-	-	-	-	-
baron	SCDC	Copula	quiescent_	800	0.117727	0.715308	-	-	-	-	-	-	-	-	-	-	-	-
baron	SCDC	Copula	acinar	900	0.141045	0.915728	-	-	-	-	-	-	-	-	-	-	-	-
baron	SCDC	Copula	activated_	900	0.088707	0.870924	-	-	-	-	-	-	-	-	-	-	-	-
baron	SCDC	Copula	alpha	900	0.061388	0.934386	-	-	-	-	-	-	-	-	-	-	-	-
baron	SCDC	Copula	beta	900	0.065354	0.944383	-	-	-	-	-	-	-	-	-	-	-	-
baron	SCDC	Copula	delta	900	0.07513	0.853877	-	-	-	-	-	-	-	-	-	-	-	-
baron	SCDC	Copula	ductal	900	0.066779	0.919871	-	-	-	-	-	-	-	-	-	-	-	-
baron	SCDC	Copula	endothelia	900	0.066627	0.925807	-	-	-	-	-	-	-	-	-	-	-	-
baron	SCDC	Copula	gamma	900	0.088362	0.901354	-	-	-	-	-	-	-	-	-	-	-	-
baron	SCDC	Copula	macropha	900	0.043663	0.920444	-	-	-	-	-	-	-	-	-	-	-	-
baron	SCDC	Copula	quiescent_	900	0.117156	0.719673	-	-	-	-	-	-	-	-	-	-	-	-
baron	SCDC	Copula	acinar	1000	0.14179	0.915306	-	-	-	-	-	-	-	-	-	-	-	-
baron	SCDC	Copula	activated_	1000	0.088974	0.870263	-	-	-	-	-	-	-	-	-	-	-	-
baron	SCDC	Copula	alpha	1000	0.060689	0.934685	-	-	-	-	-	-	-	-	-	-	-	-
baron	SCDC	Copula	beta	1000	0.072979	0.941795	-	-	-	-	-	-	-	-	-	-	-	-
baron	SCDC	Copula	delta	1000	0.077126	0.846726	-	-	-	-	-	-	-	-	-	-	-	-
baron	SCDC	Copula	ductal	1000	0.066449	0.918611	-	-	-	-	-	-	-	-	-	-	-	-
baron	SCDC	Copula	endothelia	1000	0.067288	0.921485	-	-	-	-	-	-	-	-	-	-	-	-

baron	SCDC	Copula	gamma	1000	0.089369	0.89653	-	-	-	-	-	-	-	-	-	-	-	-
baron	SCDC	Copula	macropha	1000	0.043818	0.919978	-	-	-	-	-	-	-	-	-	-	-	-
baron	SCDC	Copula	quiescent_	1000	0.116965	0.715017	-	-	-	-	-	-	-	-	-	-	-	-
baron	SCDC	CTGAN	acinar	100	-	-	0.156936	0.903901	0.146164	0.910001	0.139974	0.910622	0.149091	0.908333	0.136506	0.91283	0.151265	0.905184
baron	SCDC	CTGAN	activated_	100	-	-	0.076509	0.886391	0.07524	0.885042	0.069107	0.887132	0.074911	0.89266	0.071874	0.88952	0.076206	0.889107
baron	SCDC	CTGAN	alpha	100	-	-	0.052967	0.937653	0.065529	0.931368	0.056636	0.934252	0.062921	0.932208	0.063195	0.933059	0.062306	0.935115
baron	SCDC	CTGAN	beta	100	-	-	0.086157	0.937416	0.092189	0.932558	0.0932	0.933578	0.047509	0.951009	0.092066	0.930923	0.052659	0.9483
baron	SCDC	CTGAN	delta	100	-	-	0.092242	0.835548	0.092765	0.824455	0.092245	0.836394	0.077785	0.857279	0.09165	0.824573	0.079807	0.86285
baron	SCDC	CTGAN	ductal	100	-	-	0.06123	0.915731	0.063376	0.915991	0.058073	0.926635	0.060547	0.923089	0.065158	0.914094	0.066126	0.914635
baron	SCDC	CTGAN	endothelia	100	-	-	0.070748	0.913145	0.062474	0.935578	0.070418	0.905486	0.066046	0.929612	0.065894	0.920391	0.064573	0.926549
baron	SCDC	CTGAN	gamma	100	-	-	0.076858	0.856997	0.082606	0.860118	0.07808	0.852834	0.074842	0.904166	0.080812	0.851595	0.070745	0.890718
baron	SCDC	CTGAN	macropha	100	-	-	0.044822	0.91611	0.042733	0.924163	0.043586	0.923155	0.042617	0.926543	0.043306	0.923099	0.042597	0.925736
baron	SCDC	CTGAN	quiescent_	100	-	-	0.114774	0.730141	0.105828	0.760149	0.098709	0.786985	0.10724	0.770314	0.102065	0.763913	0.099554	0.779749
baron	SCDC	CTGAN	acinar	200	-	-	0.157834	0.904257	0.149166	0.908829	0.149606	0.907981	0.14662	0.907909	0.14145	0.912776	0.150139	0.905625
baron	SCDC	CTGAN	activated_	200	-	-	0.079846	0.887151	0.073851	0.887179	0.067629	0.888732	0.071706	0.895897	0.072884	0.890103	0.074412	0.888481
baron	SCDC	CTGAN	alpha	200	-	-	0.0523	0.936585	0.064166	0.932278	0.057137	0.933753	0.066329	0.931795	0.065693	0.93152	0.062162	0.935686
baron	SCDC	CTGAN	beta	200	-	-	0.092881	0.931176	0.08306	0.935589	0.091261	0.933702	0.047871	0.951358	0.085898	0.935357	0.059362	0.946599
baron	SCDC	CTGAN	delta	200	-	-	0.093447	0.826257	0.093131	0.833066	0.091468	0.832518	0.077872	0.862596	0.089632	0.834225	0.083519	0.855212
baron	SCDC	CTGAN	ductal	200	-	-	0.061657	0.914844	0.061986	0.914931	0.061248	0.923315	0.061308	0.923141	0.066701	0.910652	0.066913	0.915704
baron	SCDC	CTGAN	endothelia	200	-	-	0.069596	0.921628	0.063989	0.93408	0.068179	0.911831	0.064925	0.926722	0.064518	0.928439	0.060147	0.938772
baron	SCDC	CTGAN	gamma	200	-	-	0.079333	0.860589	0.078114	0.865338	0.077705	0.861811	0.071419	0.900977	0.080614	0.86223	0.071224	0.885363
baron	SCDC	CTGAN	macropha	200	-	-	0.044749	0.91643	0.042883	0.923834	0.043858	0.921519	0.042124	0.927511	0.04355	0.922703	0.042887	0.923976
baron	SCDC	CTGAN	quiescent_	200	-	-	0.119926	0.719229	0.106927	0.764584	0.095358	0.800787	0.102426	0.786054	0.102892	0.766254	0.097743	0.789396
baron	SCDC	CTGAN	acinar	300	-	-	0.156517	0.904458	0.151426	0.908381	0.148259	0.908434	0.147185	0.907046	0.137009	0.913817	0.154708	0.902963
baron	SCDC	CTGAN	activated_	300	-	-	0.077596	0.886605	0.073194	0.884772	0.066084	0.88878	0.072367	0.895117	0.071544	0.891018	0.074035	0.887039
baron	SCDC	CTGAN	alpha	300	-	-	0.053477	0.936967	0.062164	0.934356	0.056555	0.934159	0.064303	0.932808	0.064133	0.932743	0.066538	0.934533
baron	SCDC	CTGAN	beta	300	-	-	0.093897	0.930553	0.088914	0.932891	0.088037	0.934155	0.046552	0.951801	0.092226	0.932224	0.064495	0.944704
baron	SCDC	CTGAN	delta	300	-	-	0.093905	0.827209	0.094776	0.823213	0.092195	0.838274	0.07577	0.860926	0.090064	0.828642	0.085563	0.845383
baron	SCDC	CTGAN	ductal	300	-	-	0.062512	0.913931	0.062588	0.915456	0.063119	0.918322	0.062698	0.923036	0.066499	0.913116	0.066683	0.910769
baron	SCDC	CTGAN	endothelia	300	-	-	0.070774	0.921225	0.064883	0.932498	0.068761	0.908087	0.064196	0.932582	0.065141	0.928089	0.062285	0.932968
baron	SCDC	CTGAN	gamma	300	-	-	0.079742	0.856821	0.078814	0.857654	0.077075	0.859502	0.074043	0.907535	0.082052	0.856106	0.073151	0.882301
baron	SCDC	CTGAN	macropha	300	-	-	0.044068	0.919089	0.043062	0.9228	0.043492	0.923125	0.042137	0.927854	0.043188	0.92418	0.042959	0.923576
baron	SCDC	CTGAN	quiescent_	300	-	-	0.117943	0.729821	0.105617	0.768298	0.094673	0.805982	0.104669	0.783526	0.102563	0.770047	0.097229	0.790699
baron	SCDC	CTGAN	acinar	400	-	-	0.156119	0.905046	0.150522	0.90796	0.148229	0.907665	0.146303	0.908212	0.13637	0.914171	0.154345	0.903317
baron	SCDC	CTGAN	activated_	400	-	-	0.077176	0.88939	0.073774	0.88369	0.067797	0.887948	0.071718	0.896394	0.071186	0.889728	0.073859	0.890093
baron	SCDC	CTGAN	alpha	400	-	-	0.052235	0.937521	0.064572	0.933114	0.057001	0.93373	0.065224	0.932029	0.066312	0.931813	0.062524	0.935908

baron	SCDC	CTGAN	beta	400	-	-	0.092869	0.930601	0.084494	0.934915	0.09143	0.933593	0.047151	0.951319	0.091002	0.93206	0.062825	0.945849
baron	SCDC	CTGAN	delta	400	-	-	0.095163	0.826353	0.093484	0.829751	0.090436	0.838802	0.077407	0.861961	0.0905	0.828307	0.083365	0.853257
baron	SCDC	CTGAN	ductal	400	-	-	0.063004	0.913369	0.062978	0.914363	0.062518	0.919159	0.061776	0.923736	0.0667	0.913794	0.067791	0.910775
baron	SCDC	CTGAN	endothelia	400	-	-	0.069957	0.921295	0.064435	0.931462	0.069254	0.908721	0.063907	0.929862	0.065155	0.92686	0.060998	0.935175
baron	SCDC	CTGAN	gamma	400	-	-	0.079247	0.850762	0.078288	0.863649	0.077191	0.863131	0.072914	0.905884	0.082969	0.855627	0.072436	0.88675
baron	SCDC	CTGAN	macropha	400	-	-	0.044409	0.917792	0.04282	0.923612	0.043709	0.922826	0.042221	0.927605	0.043255	0.923633	0.042993	0.923819
baron	SCDC	CTGAN	quiescent_	400	-	-	0.117032	0.732745	0.105636	0.768578	0.095864	0.798136	0.103881	0.787269	0.100855	0.775155	0.097426	0.791218
baron	SCDC	CTGAN	acinar	500	-	-	0.156777	0.904107	0.152723	0.908152	0.147803	0.907638	0.147593	0.908124	0.139069	0.913694	0.155032	0.902743
baron	SCDC	CTGAN	activated_	500	-	-	0.07808	0.886514	0.073102	0.885858	0.066977	0.889525	0.072311	0.895808	0.072178	0.888218	0.074472	0.888062
baron	SCDC	CTGAN	alpha	500	-	-	0.055395	0.93613	0.06538	0.932367	0.057954	0.932861	0.063688	0.93237	0.064033	0.932985	0.065145	0.935021
baron	SCDC	CTGAN	beta	500	-	-	0.094288	0.929414	0.085591	0.9346	0.094369	0.932817	0.046578	0.951407	0.092343	0.931855	0.060692	0.945375
baron	SCDC	CTGAN	delta	500	-	-	0.094583	0.825455	0.093863	0.82881	0.091576	0.834033	0.076478	0.864186	0.090408	0.829038	0.084121	0.853227
baron	SCDC	CTGAN	ductal	500	-	-	0.062395	0.913238	0.063276	0.91315	0.061964	0.920039	0.062585	0.922168	0.066143	0.913009	0.067845	0.911847
baron	SCDC	CTGAN	endothelia	500	-	-	0.070416	0.91872	0.064058	0.931931	0.070008	0.905676	0.063808	0.930102	0.064714	0.928459	0.060395	0.93691
baron	SCDC	CTGAN	gamma	500	-	-	0.080616	0.85515	0.078513	0.864239	0.078452	0.857483	0.071709	0.904909	0.081899	0.856004	0.071493	0.88607
baron	SCDC	CTGAN	macropha	500	-	-	0.044628	0.917015	0.04326	0.922007	0.043614	0.923173	0.0424	0.926671	0.043235	0.924012	0.042809	0.924469
baron	SCDC	CTGAN	quiescent_	500	-	-	0.118243	0.724691	0.104367	0.771726	0.095475	0.800014	0.103428	0.787349	0.101887	0.769817	0.096962	0.791202
baron	SCDC	CTGAN	acinar	600	-	-	0.157508	0.902951	0.152561	0.907721	0.14929	0.907617	0.147649	0.907687	0.140224	0.912191	0.154419	0.903001
baron	SCDC	CTGAN	activated_	600	-	-	0.076998	0.887205	0.07523	0.882708	0.067469	0.888949	0.071811	0.896622	0.072671	0.891311	0.07427	0.888206
baron	SCDC	CTGAN	alpha	600	-	-	0.052738	0.936871	0.063074	0.933518	0.057126	0.933966	0.06504	0.932617	0.063297	0.933135	0.064064	0.934966
baron	SCDC	CTGAN	beta	600	-	-	0.093251	0.929689	0.08504	0.934856	0.088496	0.934677	0.046641	0.951514	0.0923	0.931226	0.062731	0.945371
baron	SCDC	CTGAN	delta	600	-	-	0.093174	0.828559	0.093799	0.828679	0.092794	0.837859	0.076482	0.861776	0.090519	0.828879	0.084115	0.852584
baron	SCDC	CTGAN	ductal	600	-	-	0.062655	0.912811	0.062804	0.914197	0.062942	0.919561	0.063365	0.920384	0.06573	0.912889	0.06799	0.910561
baron	SCDC	CTGAN	endothelia	600	-	-	0.070662	0.914021	0.064001	0.933442	0.069919	0.903716	0.06459	0.928254	0.06558	0.926511	0.061758	0.933964
baron	SCDC	CTGAN	gamma	600	-	-	0.079346	0.857248	0.07815	0.863015	0.076537	0.858156	0.073529	0.906939	0.081582	0.860734	0.072219	0.886476
baron	SCDC	CTGAN	macropha	600	-	-	0.044579	0.917208	0.0433	0.921887	0.043481	0.923503	0.042327	0.927323	0.043201	0.924093	0.0429	0.924064
baron	SCDC	CTGAN	quiescent_	600	-	-	0.116887	0.727764	0.107351	0.764017	0.095745	0.798386	0.103441	0.786454	0.103902	0.762964	0.097026	0.790325
baron	SCDC	CTGAN	acinar	700	-	-	0.158663	0.903044	0.151629	0.908367	0.148862	0.907452	0.147997	0.907365	0.140451	0.912783	0.154081	0.903189
baron	SCDC	CTGAN	activated_	700	-	-	0.078205	0.886551	0.073121	0.883975	0.066929	0.889669	0.071553	0.895954	0.071659	0.890891	0.073725	0.889272
baron	SCDC	CTGAN	alpha	700	-	-	0.054271	0.936647	0.062312	0.933617	0.056648	0.933768	0.063769	0.932516	0.063872	0.933857	0.063086	0.935536
baron	SCDC	CTGAN	beta	700	-	-	0.094029	0.927728	0.087204	0.933886	0.091481	0.934129	0.047219	0.951648	0.091229	0.930126	0.065959	0.94445
baron	SCDC	CTGAN	delta	700	-	-	0.09462	0.826196	0.093528	0.826988	0.091583	0.837092	0.077301	0.861098	0.090464	0.828287	0.084817	0.850103
baron	SCDC	CTGAN	ductal	700	-	-	0.062641	0.912567	0.063127	0.914408	0.063372	0.916701	0.062449	0.92294	0.066799	0.912642	0.067403	0.912234
baron	SCDC	CTGAN	endothelia	700	-	-	0.070924	0.917477	0.064237	0.932621	0.069467	0.906504	0.064094	0.929687	0.064858	0.926636	0.060578	0.935559
baron	SCDC	CTGAN	gamma	700	-	-	0.079966	0.852345	0.078723	0.862642	0.077189	0.858694	0.072127	0.90692	0.082148	0.858533	0.072104	0.884055
baron	SCDC	CTGAN	macropha	700	-	-	0.044642	0.916862	0.043034	0.922868	0.043664	0.922524	0.042347	0.926982	0.043285	0.923603	0.042877	0.923978

baron	SCDC	CTGAN	quiescent_	700	-	-	0.118303	0.726441	0.104985	0.770853	0.095484	0.800445	0.102549	0.787501	0.101759	0.770184	0.096041	0.794388
baron	SCDC	CTGAN	acinar	800	-	-	0.158251	0.902856	0.151639	0.908448	0.148278	0.907325	0.145668	0.908021	0.140145	0.913016	0.152448	0.904512
baron	SCDC	CTGAN	activated_	800	-	-	0.077157	0.887191	0.073554	0.883628	0.066711	0.890303	0.071632	0.895713	0.071443	0.889085	0.074138	0.887543
baron	SCDC	CTGAN	alpha	800	-	-	0.054169	0.936852	0.063023	0.933804	0.05811	0.933438	0.064379	0.932788	0.064559	0.932515	0.065757	0.93448
baron	SCDC	CTGAN	beta	800	-	-	0.094233	0.926478	0.085216	0.934835	0.090204	0.934027	0.047018	0.951422	0.090665	0.931777	0.065779	0.944663
baron	SCDC	CTGAN	delta	800	-	-	0.094864	0.825573	0.09413	0.828309	0.091303	0.836926	0.076947	0.861767	0.090034	0.829025	0.085636	0.847075
baron	SCDC	CTGAN	ductal	800	-	-	0.062895	0.912315	0.063495	0.91337	0.06202	0.920655	0.062454	0.922877	0.066614	0.912702	0.068515	0.91073
baron	SCDC	CTGAN	endothelia	800	-	-	0.070303	0.920371	0.064032	0.933045	0.069749	0.905805	0.063156	0.930559	0.065306	0.927273	0.061728	0.936546
baron	SCDC	CTGAN	gamma	800	-	-	0.080388	0.85359	0.077771	0.863513	0.076891	0.861775	0.072185	0.905282	0.082632	0.854543	0.073	0.883811
baron	SCDC	CTGAN	macropha	800	-	-	0.04442	0.917771	0.042914	0.923298	0.043488	0.923404	0.042302	0.927044	0.043513	0.922865	0.042951	0.923882
baron	SCDC	CTGAN	quiescent_	800	-	-	0.117594	0.729372	0.105627	0.769062	0.095068	0.802015	0.102412	0.789808	0.102256	0.77067	0.09731	0.79038
baron	SCDC	CTGAN	acinar	900	-	-	0.156724	0.903667	0.152487	0.908246	0.14852	0.907176	0.147675	0.907918	0.139908	0.912607	0.153426	0.903468
baron	SCDC	CTGAN	activated_	900	-	-	0.077786	0.887524	0.073367	0.884515	0.066837	0.88985	0.071163	0.896093	0.071676	0.890536	0.073714	0.889415
baron	SCDC	CTGAN	alpha	900	-	-	0.053397	0.937339	0.062039	0.934001	0.057009	0.93349	0.06305	0.933625	0.064657	0.932966	0.065629	0.934715
baron	SCDC	CTGAN	beta	900	-	-	0.093481	0.927922	0.086347	0.934902	0.091354	0.934096	0.046813	0.951293	0.091381	0.932296	0.060613	0.946055
baron	SCDC	CTGAN	delta	900	-	-	0.09435	0.826408	0.093193	0.829954	0.0915	0.837802	0.076241	0.863492	0.090223	0.829674	0.083362	0.856921
baron	SCDC	CTGAN	ductal	900	-	-	0.062902	0.912919	0.06342	0.914319	0.062538	0.919451	0.062491	0.922149	0.066492	0.912973	0.068243	0.911579
baron	SCDC	CTGAN	endothelia	900	-	-	0.07024	0.917735	0.064444	0.932757	0.069953	0.904721	0.06442	0.929188	0.064684	0.928591	0.060605	0.936737
baron	SCDC	CTGAN	gamma	900	-	-	0.079448	0.856266	0.077951	0.864728	0.077089	0.859988	0.071732	0.906968	0.081853	0.857323	0.071262	0.887783
baron	SCDC	CTGAN	macropha	900	-	-	0.044462	0.917621	0.043252	0.92204	0.043666	0.922851	0.04239	0.926942	0.043358	0.923394	0.042826	0.924378
baron	SCDC	CTGAN	quiescent_	900	-	-	0.117294	0.727717	0.104682	0.771204	0.095331	0.801324	0.101961	0.792078	0.101588	0.771721	0.096383	0.794314
baron	SCDC	CTGAN	acinar	1000	-	-	0.157101	0.90338	0.151046	0.90863	0.147923	0.907863	0.146017	0.907493	0.139555	0.912231	0.152961	0.904085
baron	SCDC	CTGAN	activated_	1000	-	-	0.077547	0.887445	0.074045	0.884975	0.067039	0.89032	0.072437	0.895307	0.071556	0.890216	0.073663	0.888988
baron	SCDC	CTGAN	alpha	1000	-	-	0.053903	0.936212	0.064531	0.932827	0.058507	0.932919	0.066018	0.932262	0.065473	0.932503	0.063483	0.935266
baron	SCDC	CTGAN	beta	1000	-	-	0.094408	0.931807	0.086497	0.933926	0.09257	0.93357	0.04726	0.951169	0.090889	0.932207	0.066241	0.944369
baron	SCDC	CTGAN	delta	1000	-	-	0.094031	0.827298	0.093626	0.828487	0.091722	0.835836	0.077099	0.861319	0.090209	0.830055	0.084605	0.850166
baron	SCDC	CTGAN	ductal	1000	-	-	0.062865	0.912698	0.063346	0.913711	0.061879	0.920939	0.062971	0.923144	0.066109	0.913198	0.06765	0.911732
baron	SCDC	CTGAN	endothelia	1000	-	-	0.070365	0.91763	0.064894	0.931824	0.069852	0.906425	0.063973	0.929592	0.064664	0.929819	0.060911	0.935685
baron	SCDC	CTGAN	gamma	1000	-	-	0.079427	0.855066	0.078731	0.863815	0.077766	0.858855	0.073471	0.905882	0.081725	0.855228	0.072348	0.885441
baron	SCDC	CTGAN	macropha	1000	-	-	0.044531	0.917232	0.04301	0.923079	0.043531	0.923125	0.042179	0.92763	0.043404	0.923096	0.04291	0.924019
baron	SCDC	CTGAN	quiescent_	1000	-	-	0.116975	0.729413	0.10637	0.76528	0.095797	0.798543	0.103462	0.786997	0.101401	0.774359	0.096386	0.793348
baron	SCDC	TVAE	acinar	100	-	-	0.101896	0.92605	0.096875	0.925531	0.094641	0.927639	0.100981	0.924018	0.099461	0.922888	0.093063	0.926694
baron	SCDC	TVAE	activated_	100	-	-	0.069295	0.896957	0.065475	0.893824	0.067492	0.884375	0.066109	0.88107	0.070358	0.888473	0.066893	0.890528
baron	SCDC	TVAE	alpha	100	-	-	0.052285	0.936265	0.062897	0.930693	0.060279	0.932562	0.077724	0.924528	0.05677	0.934507	0.061136	0.931792
baron	SCDC	TVAE	beta	100	-	-	0.045563	0.952354	0.045288	0.952728	0.045427	0.952471	0.046134	0.951591	0.044998	0.953354	0.045232	0.953535
baron	SCDC	TVAE	delta	100	-	-	0.073223	0.860982	0.072934	0.860648	0.072126	0.865586	0.07359	0.85954	0.073094	0.860743	0.073059	0.860659

baron	SCDC	TVAE	ductal	100	-	-	0.103046	0.938154	0.106819	0.938078	0.110637	0.939071	0.099626	0.932458	0.105871	0.939125	0.109253	0.936908
baron	SCDC	TVAE	endothelia	100	-	-	0.065256	0.94334	0.062697	0.941219	0.063304	0.946353	0.063045	0.941413	0.065681	0.940013	0.063534	0.942842
baron	SCDC	TVAE	gamma	100	-	-	0.088197	0.906131	0.086236	0.899808	0.083524	0.899113	0.090489	0.895034	0.086547	0.905318	0.088829	0.908438
baron	SCDC	TVAE	macropha	100	-	-	0.047703	0.923912	0.046781	0.922734	0.044365	0.924557	0.047879	0.922322	0.048012	0.921155	0.045857	0.9238
baron	SCDC	TVAE	quiescent_	100	-	-	0.096416	0.81855	0.091716	0.836697	0.094043	0.826209	0.09481	0.834059	0.097482	0.810565	0.093155	0.832543
baron	SCDC	TVAE	acinar	200	-	-	0.100164	0.927047	0.095887	0.926109	0.094516	0.927169	0.099231	0.924206	0.09854	0.924485	0.093081	0.926515
baron	SCDC	TVAE	activated_	200	-	-	0.068609	0.896812	0.06664	0.894624	0.067725	0.884909	0.065915	0.882507	0.070148	0.889776	0.066542	0.891765
baron	SCDC	TVAE	alpha	200	-	-	0.053692	0.935865	0.063441	0.930506	0.06133	0.931655	0.071761	0.927392	0.054815	0.935585	0.060612	0.932136
baron	SCDC	TVAE	beta	200	-	-	0.046158	0.951801	0.045334	0.952734	0.045477	0.952508	0.046193	0.95205	0.044989	0.953314	0.045108	0.953226
baron	SCDC	TVAE	delta	200	-	-	0.073271	0.859415	0.072718	0.86135	0.0723	0.866456	0.073334	0.858617	0.072603	0.863952	0.074218	0.855686
baron	SCDC	TVAE	ductal	200	-	-	0.103688	0.937815	0.107082	0.938485	0.111558	0.938193	0.101026	0.934093	0.104452	0.940511	0.111594	0.937352
baron	SCDC	TVAE	endothelia	200	-	-	0.064762	0.942975	0.063409	0.942559	0.063185	0.94663	0.063392	0.941653	0.064889	0.940674	0.063321	0.942603
baron	SCDC	TVAE	gamma	200	-	-	0.089222	0.904861	0.086013	0.899926	0.082432	0.897956	0.091653	0.899951	0.083165	0.9061	0.090042	0.910762
baron	SCDC	TVAE	macropha	200	-	-	0.047844	0.924189	0.046708	0.922717	0.044706	0.922862	0.048085	0.922304	0.04829	0.920651	0.045451	0.923609
baron	SCDC	TVAE	quiescent_	200	-	-	0.096267	0.819347	0.092558	0.830126	0.094568	0.824832	0.094478	0.83454	0.097831	0.810351	0.092473	0.836436
baron	SCDC	TVAE	acinar	300	-	-	0.102039	0.925977	0.09605	0.926002	0.093707	0.928073	0.098761	0.924537	0.098008	0.924646	0.094257	0.926388
baron	SCDC	TVAE	activated_	300	-	-	0.069359	0.896879	0.065719	0.894763	0.067828	0.885949	0.065282	0.882878	0.070566	0.888106	0.06664	0.891548
baron	SCDC	TVAE	alpha	300	-	-	0.053669	0.936027	0.061946	0.931244	0.059124	0.932634	0.074522	0.926253	0.053239	0.936328	0.060392	0.932312
baron	SCDC	TVAE	beta	300	-	-	0.045929	0.951929	0.045415	0.952756	0.045346	0.952699	0.046046	0.952203	0.044932	0.953442	0.045031	0.95335
baron	SCDC	TVAE	delta	300	-	-	0.072925	0.86088	0.072389	0.862878	0.071865	0.866745	0.073256	0.858911	0.072853	0.862602	0.073191	0.859766
baron	SCDC	TVAE	ductal	300	-	-	0.103379	0.937976	0.106282	0.938427	0.113442	0.937932	0.100633	0.933472	0.106383	0.939736	0.111955	0.936297
baron	SCDC	TVAE	endothelia	300	-	-	0.065297	0.942448	0.062723	0.94309	0.063122	0.946806	0.063159	0.940958	0.064573	0.941176	0.063095	0.942227
baron	SCDC	TVAE	gamma	300	-	-	0.088856	0.904413	0.085	0.901495	0.083885	0.899289	0.091341	0.900627	0.084381	0.907464	0.089307	0.910461
baron	SCDC	TVAE	macropha	300	-	-	0.047718	0.923896	0.046797	0.922781	0.044831	0.922727	0.049043	0.922245	0.048295	0.920393	0.04574	0.923577
baron	SCDC	TVAE	quiescent_	300	-	-	0.096585	0.817909	0.09224	0.834105	0.09451	0.824527	0.093671	0.83935	0.097406	0.812902	0.09232	0.83728
baron	SCDC	TVAE	acinar	400	-	-	0.100628	0.926321	0.096	0.92599	0.093737	0.927799	0.099091	0.924187	0.097598	0.924609	0.092393	0.926971
baron	SCDC	TVAE	activated_	400	-	-	0.069053	0.89551	0.066198	0.89393	0.067813	0.885684	0.065712	0.882199	0.069952	0.889633	0.066655	0.8902
baron	SCDC	TVAE	alpha	400	-	-	0.052932	0.936402	0.062359	0.930997	0.058518	0.933097	0.074082	0.92662	0.054214	0.935881	0.060807	0.931945
baron	SCDC	TVAE	beta	400	-	-	0.04583	0.951912	0.045283	0.95285	0.045342	0.952629	0.04597	0.951912	0.044961	0.953374	0.045032	0.953284
baron	SCDC	TVAE	delta	400	-	-	0.073134	0.860451	0.072737	0.861547	0.071497	0.868284	0.073067	0.860204	0.0731	0.861275	0.073546	0.8583
baron	SCDC	TVAE	ductal	400	-	-	0.103918	0.938263	0.105714	0.938551	0.113092	0.938511	0.100315	0.933321	0.10723	0.939735	0.112584	0.936819
baron	SCDC	TVAE	endothelia	400	-	-	0.065162	0.942582	0.062996	0.942463	0.062789	0.94666	0.063368	0.940703	0.064462	0.941197	0.063256	0.941949
baron	SCDC	TVAE	gamma	400	-	-	0.088282	0.906208	0.085084	0.900906	0.08275	0.902204	0.09093	0.899715	0.085221	0.906315	0.09001	0.90908
baron	SCDC	TVAE	macropha	400	-	-	0.047597	0.923788	0.047396	0.922637	0.044564	0.923317	0.048066	0.922866	0.048303	0.920811	0.04599	0.924127
baron	SCDC	TVAE	quiescent_	400	-	-	0.096589	0.81915	0.092577	0.831456	0.094239	0.825783	0.094002	0.836297	0.096955	0.814077	0.092481	0.836055
baron	SCDC	TVAE	acinar	500	-	-	0.100897	0.926173	0.094537	0.927169	0.092935	0.928156	0.100373	0.923677	0.097834	0.924566	0.092713	0.926913

baron	SCDC	TVAE	activated_	500	-	-	0.068959	0.896683	0.067149	0.893606	0.067593	0.885856	0.06573	0.882135	0.070524	0.88996	0.066838	0.891053
baron	SCDC	TVAE	alpha	500	-	-	0.052493	0.936675	0.062468	0.931006	0.059382	0.932767	0.074882	0.926213	0.054513	0.935865	0.060423	0.932109
baron	SCDC	TVAE	beta	500	-	-	0.045945	0.951837	0.045313	0.95287	0.04537	0.952585	0.046063	0.952111	0.044914	0.953539	0.045057	0.95352
baron	SCDC	TVAE	delta	500	-	-	0.07271	0.862167	0.072547	0.862223	0.071948	0.867154	0.073322	0.858558	0.073109	0.861067	0.073711	0.857964
baron	SCDC	TVAE	ductal	500	-	-	0.10279	0.938472	0.107498	0.938428	0.112804	0.938634	0.099569	0.933148	0.106114	0.939502	0.112025	0.937193
baron	SCDC	TVAE	endothelia	500	-	-	0.064817	0.942404	0.063069	0.942881	0.062868	0.946775	0.063304	0.941039	0.064696	0.941152	0.062896	0.942134
baron	SCDC	TVAE	gamma	500	-	-	0.087633	0.907696	0.085053	0.901305	0.082664	0.898972	0.09172	0.900386	0.084626	0.906546	0.089419	0.910707
baron	SCDC	TVAE	macropha	500	-	-	0.047644	0.923836	0.047138	0.922898	0.045097	0.921761	0.047961	0.922463	0.048111	0.920596	0.04566	0.923858
baron	SCDC	TVAE	quiescent_	500	-	-	0.096598	0.8175	0.09269	0.82839	0.094435	0.82583	0.093941	0.837114	0.097487	0.811356	0.092436	0.836609
baron	SCDC	TVAE	acinar	600	-	-	0.101795	0.92628	0.096321	0.925956	0.092865	0.928076	0.099672	0.924408	0.097646	0.924775	0.092758	0.926787
baron	SCDC	TVAE	activated_	600	-	-	0.068983	0.896734	0.066953	0.892992	0.067789	0.885297	0.065779	0.882241	0.070256	0.888632	0.066441	0.892073
baron	SCDC	TVAE	alpha	600	-	-	0.053516	0.936018	0.062374	0.930839	0.058263	0.933046	0.070934	0.927904	0.054425	0.935687	0.061216	0.931637
baron	SCDC	TVAE	beta	600	-	-	0.046082	0.951726	0.045232	0.952901	0.045439	0.95244	0.04592	0.95228	0.044943	0.953584	0.045063	0.953542
baron	SCDC	TVAE	delta	600	-	-	0.073246	0.859809	0.072606	0.862112	0.071549	0.868567	0.072812	0.860831	0.073056	0.861523	0.073369	0.859183
baron	SCDC	TVAE	ductal	600	-	-	0.102909	0.938017	0.107071	0.938168	0.11346	0.938678	0.100137	0.93333	0.107259	0.93968	0.113116	0.936934
baron	SCDC	TVAE	endothelia	600	-	-	0.064801	0.942575	0.062832	0.942775	0.062834	0.946943	0.063146	0.941062	0.065119	0.940826	0.06307	0.942295
baron	SCDC	TVAE	gamma	600	-	-	0.08945	0.904717	0.084973	0.900122	0.082038	0.901524	0.091324	0.902538	0.085378	0.906278	0.08948	0.910816
baron	SCDC	TVAE	macropha	600	-	-	0.047607	0.924017	0.047053	0.922768	0.044864	0.922319	0.047637	0.922645	0.048161	0.920951	0.045836	0.924069
baron	SCDC	TVAE	quiescent_	600	-	-	0.096663	0.817526	0.092672	0.829044	0.094447	0.824895	0.09402	0.836124	0.097888	0.809944	0.092325	0.8372
baron	SCDC	TVAE	acinar	700	-	-	0.102342	0.925898	0.094667	0.926622	0.093192	0.927927	0.099222	0.923985	0.097799	0.924721	0.093626	0.926682
baron	SCDC	TVAE	activated_	700	-	-	0.068916	0.896416	0.066376	0.894214	0.067724	0.887248	0.065604	0.882028	0.070354	0.889403	0.066422	0.891817
baron	SCDC	TVAE	alpha	700	-	-	0.05214	0.93689	0.062361	0.930873	0.05853	0.933048	0.074706	0.926199	0.053873	0.93631	0.061231	0.931802
baron	SCDC	TVAE	beta	700	-	-	0.045792	0.952034	0.045273	0.952833	0.045351	0.952618	0.045988	0.951901	0.044911	0.953483	0.045052	0.953429
baron	SCDC	TVAE	delta	700	-	-	0.073154	0.860951	0.072486	0.862568	0.071551	0.868604	0.072969	0.86009	0.072628	0.863351	0.072804	0.861142
baron	SCDC	TVAE	ductal	700	-	-	0.102548	0.937969	0.106986	0.939026	0.112781	0.938735	0.09962	0.933668	0.106295	0.939944	0.112386	0.936265
baron	SCDC	TVAE	endothelia	700	-	-	0.065031	0.942223	0.06274	0.942939	0.062706	0.946883	0.063096	0.940978	0.064591	0.941233	0.063002	0.942065
baron	SCDC	TVAE	gamma	700	-	-	0.088259	0.906594	0.085203	0.900191	0.08209	0.901936	0.091305	0.901004	0.084663	0.90681	0.088793	0.909417
baron	SCDC	TVAE	macropha	700	-	-	0.047587	0.923671	0.046928	0.922713	0.04455	0.923784	0.047967	0.922616	0.048006	0.92064	0.045716	0.923915
baron	SCDC	TVAE	quiescent_	700	-	-	0.096342	0.819231	0.092535	0.829742	0.094327	0.824443	0.093999	0.836952	0.097505	0.811262	0.092214	0.838315
baron	SCDC	TVAE	acinar	800	-	-	0.102272	0.92579	0.095592	0.926003	0.092318	0.928105	0.099105	0.924376	0.097879	0.924843	0.093711	0.926514
baron	SCDC	TVAE	activated_	800	-	-	0.068734	0.896976	0.066865	0.893691	0.067551	0.88602	0.06543	0.882907	0.070929	0.889073	0.066212	0.892093
baron	SCDC	TVAE	alpha	800	-	-	0.053371	0.935998	0.062341	0.93097	0.058886	0.932598	0.074769	0.926043	0.05328	0.936394	0.061592	0.931468
baron	SCDC	TVAE	beta	800	-	-	0.045906	0.951924	0.045165	0.953088	0.04537	0.952577	0.045845	0.95211	0.044962	0.953375	0.045093	0.953313
baron	SCDC	TVAE	delta	800	-	-	0.072656	0.862003	0.072473	0.862752	0.07195	0.866934	0.072928	0.860591	0.073124	0.861099	0.072906	0.860519
baron	SCDC	TVAE	ductal	800	-	-	0.103245	0.93786	0.106275	0.938554	0.113662	0.938753	0.099673	0.933377	0.107146	0.939678	0.112111	0.936769
baron	SCDC	TVAE	endothelia	800	-	-	0.065319	0.942584	0.062981	0.942617	0.062687	0.947032	0.063313	0.940604	0.064938	0.941551	0.06313	0.942299

baron	SCDC	TVAE	gamma	800	-	-	0.087662	0.905556	0.084533	0.899899	0.082727	0.900043	0.090716	0.901065	0.084495	0.90742	0.088533	0.910794
baron	SCDC	TVAE	macropha	800	-	-	0.047439	0.924268	0.047301	0.922774	0.044812	0.922515	0.047823	0.922397	0.048049	0.920818	0.045762	0.923179
baron	SCDC	TVAE	quiescent_	800	-	-	0.09633	0.819051	0.092762	0.82808	0.094187	0.826536	0.094165	0.835965	0.097625	0.811883	0.092054	0.837864
baron	SCDC	TVAE	acinar	900	-	-	0.102401	0.925848	0.095274	0.926419	0.093128	0.927916	0.099715	0.92422	0.098568	0.923866	0.093484	0.92636
baron	SCDC	TVAE	activated_	900	-	-	0.069156	0.896609	0.066314	0.894063	0.067739	0.885943	0.065799	0.88265	0.070533	0.889094	0.066341	0.891474
baron	SCDC	TVAE	alpha	900	-	-	0.053462	0.935923	0.062529	0.930907	0.059012	0.932885	0.073731	0.926564	0.05471	0.935476	0.061459	0.931452
baron	SCDC	TVAE	beta	900	-	-	0.045875	0.951857	0.04543	0.952667	0.045429	0.952481	0.045864	0.952037	0.044911	0.953539	0.045055	0.953288
baron	SCDC	TVAE	delta	900	-	-	0.07243	0.862904	0.072469	0.862683	0.071823	0.867424	0.072869	0.860799	0.072806	0.862592	0.073637	0.857691
baron	SCDC	TVAE	ductal	900	-	-	0.102713	0.937994	0.105446	0.938676	0.112871	0.938523	0.099263	0.933199	0.106796	0.939556	0.112283	0.936677
baron	SCDC	TVAE	endothelia	900	-	-	0.064985	0.942952	0.062754	0.942615	0.062547	0.946468	0.063212	0.940407	0.06499	0.941049	0.063203	0.941747
baron	SCDC	TVAE	gamma	900	-	-	0.08766	0.906069	0.08469	0.900412	0.082628	0.901521	0.090603	0.900929	0.084555	0.905374	0.089386	0.910215
baron	SCDC	TVAE	macropha	900	-	-	0.047699	0.923744	0.04669	0.922888	0.044808	0.922529	0.047806	0.922764	0.048237	0.92064	0.045862	0.924078
baron	SCDC	TVAE	quiescent_	900	-	-	0.096642	0.817795	0.09273	0.828623	0.094315	0.82511	0.093968	0.836513	0.097641	0.810423	0.09208	0.837725
baron	SCDC	TVAE	acinar	1000	-	-	0.102177	0.926141	0.096117	0.926106	0.093583	0.927859	0.099164	0.924235	0.098238	0.924365	0.09393	0.926151
baron	SCDC	TVAE	activated_	1000	-	-	0.069316	0.897061	0.066596	0.893793	0.067731	0.886585	0.065727	0.88208	0.070434	0.889296	0.066369	0.891722
baron	SCDC	TVAE	alpha	1000	-	-	0.052973	0.936233	0.062611	0.931012	0.058314	0.933052	0.075165	0.926164	0.054697	0.935623	0.060895	0.93188
baron	SCDC	TVAE	beta	1000	-	-	0.045892	0.9519	0.045256	0.952921	0.045366	0.952727	0.046008	0.952118	0.044917	0.95351	0.045079	0.953307
baron	SCDC	TVAE	delta	1000	-	-	0.073061	0.860788	0.072451	0.862626	0.071474	0.868629	0.073241	0.85915	0.072662	0.863159	0.073341	0.859101
baron	SCDC	TVAE	ductal	1000	-	-	0.102154	0.937898	0.105979	0.938471	0.113058	0.938358	0.100235	0.93309	0.106504	0.939441	0.112158	0.936719
baron	SCDC	TVAE	endothelia	1000	-	-	0.065065	0.942936	0.062682	0.942284	0.062813	0.946668	0.06314	0.940694	0.064556	0.941771	0.062964	0.942483
baron	SCDC	TVAE	gamma	1000	-	-	0.088386	0.90649	0.085032	0.900956	0.082102	0.901443	0.091209	0.899849	0.084418	0.906176	0.089584	0.909848
baron	SCDC	TVAE	macropha	1000	-	-	0.047475	0.923957	0.047293	0.922963	0.044584	0.92328	0.047907	0.922231	0.048293	0.921114	0.045881	0.923172
baron	SCDC	TVAE	quiescent_	1000	-	-	0.096994	0.816958	0.092501	0.829605	0.094266	0.825815	0.09411	0.836487	0.097491	0.810862	0.092118	0.839202
baron	SCDC	sc-CMGAI	acinar	100	-	-	0.105498	0.932376	0.109638	0.927311	0.102939	0.93139	0.116127	0.927418	0.111577	0.929089	0.115263	0.926668
baron	SCDC	sc-CMGAI	activated_	100	-	-	0.080077	0.892921	0.078179	0.893028	0.081202	0.891718	0.080598	0.885872	0.085725	0.892027	0.077034	0.889863
baron	SCDC	sc-CMGAI	alpha	100	-	-	0.050852	0.938339	0.049384	0.937223	0.052758	0.93086	0.051161	0.937599	0.050457	0.934192	0.070945	0.925123
baron	SCDC	sc-CMGAI	beta	100	-	-	0.049747	0.948754	0.048163	0.948777	0.052778	0.952247	0.047427	0.950815	0.055337	0.953471	0.057226	0.950777
baron	SCDC	sc-CMGAI	delta	100	-	-	0.075404	0.857144	0.07973	0.839842	0.084144	0.829276	0.078511	0.84674	0.084689	0.82569	0.082628	0.821076
baron	SCDC	sc-CMGAI	ductal	100	-	-	0.064016	0.93413	0.064374	0.93841	0.063406	0.944353	0.060943	0.936987	0.058464	0.94169	0.063962	0.932984
baron	SCDC	sc-CMGAI	endothelia	100	-	-	0.066506	0.938821	0.067649	0.938708	0.06346	0.936396	0.071444	0.941065	0.068983	0.9334	0.067926	0.936775
baron	SCDC	sc-CMGAI	gamma	100	-	-	0.073075	0.904426	0.077476	0.89886	0.078832	0.900529	0.074739	0.892872	0.075542	0.900896	0.07518	0.886041
baron	SCDC	sc-CMGAI	macropha	100	-	-	0.047502	0.927567	0.04302	0.92552	0.043881	0.929158	0.042602	0.926436	0.045349	0.927989	0.044593	0.927663
baron	SCDC	sc-CMGAI	quiescent_	100	-	-	0.113317	0.741312	0.104925	0.759677	0.110717	0.739445	0.112447	0.738173	0.119829	0.72951	0.102077	0.769352
baron	SCDC	sc-CMGAI	acinar	200	-	-	0.107362	0.9309	0.10919	0.928806	0.108194	0.929776	0.115868	0.928752	0.117105	0.927549	0.11233	0.928698
baron	SCDC	sc-CMGAI	activated_	200	-	-	0.079557	0.89297	0.078912	0.890493	0.084585	0.888788	0.080601	0.888006	0.08399	0.893249	0.076505	0.890837
baron	SCDC	sc-CMGAI	alpha	200	-	-	0.049966	0.939043	0.048933	0.939704	0.05171	0.932413	0.051147	0.939074	0.050715	0.93378	0.065321	0.926895

baron	SCDC	sc-CMGAI beta	200	-	-	0.053025	0.948036	0.04766	0.949291	0.056332	0.951986	0.047977	0.949805	0.055606	0.953184	0.057865	0.952173
baron	SCDC	sc-CMGAI delta	200	-	-	0.076435	0.849818	0.080325	0.837201	0.086707	0.827564	0.077592	0.846233	0.085514	0.82515	0.081169	0.828868
baron	SCDC	sc-CMGAI ductal	200	-	-	0.066897	0.934265	0.062688	0.941523	0.065905	0.940977	0.062322	0.936589	0.06028	0.939113	0.06319	0.93699
baron	SCDC	sc-CMGAI endothelia	200	-	-	0.065913	0.941955	0.065489	0.937224	0.064829	0.938437	0.069792	0.941271	0.068401	0.935601	0.067107	0.935939
baron	SCDC	sc-CMGAI gamma	200	-	-	0.073538	0.909231	0.077016	0.901562	0.080496	0.899554	0.075414	0.897186	0.076578	0.902765	0.073659	0.891038
baron	SCDC	sc-CMGAI macropha	200	-	-	0.047572	0.925693	0.042883	0.926518	0.044762	0.928762	0.043433	0.925934	0.044971	0.926978	0.045048	0.928676
baron	SCDC	sc-CMGAI quiescent_	200	-	-	0.110766	0.746705	0.106399	0.749416	0.114132	0.736773	0.10965	0.751846	0.116004	0.739822	0.104612	0.763276
baron	SCDC	sc-CMGAI acinar	300	-	-	0.107761	0.930933	0.106613	0.929252	0.105801	0.929591	0.116665	0.928819	0.11406	0.928787	0.1125	0.92807
baron	SCDC	sc-CMGAI activated_	300	-	-	0.077991	0.89389	0.077659	0.894377	0.081616	0.891505	0.080315	0.889351	0.082484	0.893847	0.076747	0.892238
baron	SCDC	sc-CMGAI alpha	300	-	-	0.050139	0.938087	0.04906	0.940884	0.051228	0.932962	0.050669	0.938165	0.050508	0.934114	0.065688	0.927027
baron	SCDC	sc-CMGAI beta	300	-	-	0.051523	0.948956	0.048929	0.948717	0.055689	0.952717	0.046839	0.950818	0.056737	0.953694	0.05639	0.951671
baron	SCDC	sc-CMGAI delta	300	-	-	0.074142	0.859567	0.08075	0.833153	0.086002	0.830493	0.078311	0.849756	0.085666	0.825643	0.081127	0.829249
baron	SCDC	sc-CMGAI ductal	300	-	-	0.06748	0.934402	0.063522	0.940681	0.065159	0.942118	0.063528	0.935792	0.061132	0.940743	0.064369	0.935074
baron	SCDC	sc-CMGAI endothelia	300	-	-	0.067041	0.940535	0.065616	0.939002	0.064327	0.935628	0.070668	0.941292	0.06694	0.936124	0.067157	0.934806
baron	SCDC	sc-CMGAI gamma	300	-	-	0.072097	0.903756	0.076734	0.900122	0.079462	0.900595	0.074538	0.897306	0.075957	0.903756	0.074475	0.891896
baron	SCDC	sc-CMGAI macropha	300	-	-	0.047519	0.927074	0.04304	0.92649	0.044747	0.928828	0.042453	0.927754	0.04505	0.926768	0.044963	0.928665
baron	SCDC	sc-CMGAI quiescent_	300	-	-	0.110315	0.753183	0.105431	0.755762	0.111651	0.744558	0.10947	0.753562	0.112362	0.75031	0.103292	0.768732
baron	SCDC	sc-CMGAI acinar	400	-	-	0.108375	0.931082	0.108639	0.927595	0.104795	0.930622	0.117344	0.92811	0.113579	0.929274	0.111516	0.929481
baron	SCDC	sc-CMGAI activated_	400	-	-	0.08057	0.890701	0.079464	0.89374	0.082767	0.890351	0.080038	0.889496	0.083795	0.893445	0.076046	0.890938
baron	SCDC	sc-CMGAI alpha	400	-	-	0.049909	0.938496	0.049339	0.939673	0.051529	0.932428	0.050701	0.937661	0.050547	0.933726	0.0646	0.927723
baron	SCDC	sc-CMGAI beta	400	-	-	0.052695	0.948222	0.051699	0.948559	0.054647	0.952851	0.046808	0.949829	0.057128	0.953851	0.058547	0.951624
baron	SCDC	sc-CMGAI delta	400	-	-	0.076203	0.849996	0.080535	0.831924	0.085	0.829752	0.078151	0.848048	0.086162	0.825327	0.081651	0.827967
baron	SCDC	sc-CMGAI ductal	400	-	-	0.066887	0.934269	0.061748	0.940246	0.066598	0.943464	0.063235	0.936201	0.062745	0.939944	0.062739	0.936017
baron	SCDC	sc-CMGAI endothelia	400	-	-	0.067856	0.941565	0.065128	0.93852	0.064834	0.937643	0.069144	0.941857	0.067633	0.934718	0.067009	0.937443
baron	SCDC	sc-CMGAI gamma	400	-	-	0.07359	0.906691	0.076381	0.899958	0.079277	0.901102	0.074834	0.898722	0.075636	0.905651	0.072973	0.891438
baron	SCDC	sc-CMGAI macropha	400	-	-	0.04711	0.92665	0.043166	0.92566	0.044787	0.929039	0.043231	0.92566	0.04458	0.927681	0.045143	0.928632
baron	SCDC	sc-CMGAI quiescent_	400	-	-	0.114227	0.73899	0.107212	0.745792	0.11383	0.737357	0.108514	0.755684	0.113801	0.746145	0.103366	0.769722
baron	SCDC	sc-CMGAI acinar	500	-	-	0.107615	0.931777	0.107197	0.929369	0.106377	0.931653	0.115904	0.928766	0.113943	0.928973	0.111209	0.92849
baron	SCDC	sc-CMGAI activated_	500	-	-	0.079038	0.892312	0.079024	0.893152	0.081745	0.892272	0.079738	0.890917	0.08355	0.894692	0.076982	0.891609
baron	SCDC	sc-CMGAI alpha	500	-	-	0.050343	0.937995	0.049166	0.939446	0.05198	0.931962	0.050654	0.937693	0.050778	0.933484	0.069223	0.926317
baron	SCDC	sc-CMGAI beta	500	-	-	0.049209	0.948946	0.053103	0.948394	0.053411	0.952713	0.047464	0.950897	0.053427	0.953592	0.057411	0.951904
baron	SCDC	sc-CMGAI delta	500	-	-	0.07531	0.856433	0.078984	0.837489	0.084321	0.831696	0.078053	0.849174	0.08405	0.830138	0.081754	0.827254
baron	SCDC	sc-CMGAI ductal	500	-	-	0.066011	0.935208	0.062076	0.940969	0.066775	0.941688	0.063789	0.936464	0.061148	0.940163	0.064506	0.935937
baron	SCDC	sc-CMGAI endothelia	500	-	-	0.065536	0.941945	0.065732	0.940075	0.064541	0.93806	0.069637	0.941653	0.067205	0.933847	0.067202	0.93649
baron	SCDC	sc-CMGAI gamma	500	-	-	0.072698	0.905572	0.075672	0.902721	0.079618	0.902968	0.07422	0.900295	0.076019	0.907092	0.074085	0.890432
baron	SCDC	sc-CMGAI macropha	500	-	-	0.047153	0.926987	0.042966	0.925823	0.044555	0.928822	0.043079	0.925958	0.044917	0.92806	0.044774	0.929123

baron	SCDC	sc-CMGAI	quiescent_	500	-	-	0.110571	0.750637	0.10744	0.74669	0.112639	0.74151	0.10937	0.75799	0.113614	0.74522	0.103285	0.768427
baron	SCDC	sc-CMGAI	acinar	600	-	-	0.107523	0.931186	0.107646	0.928292	0.105719	0.930378	0.117451	0.927856	0.114829	0.928309	0.110896	0.92994
baron	SCDC	sc-CMGAI	activated_	600	-	-	0.079908	0.89299	0.079016	0.893262	0.082672	0.891329	0.080378	0.889837	0.083194	0.89401	0.076007	0.891004
baron	SCDC	sc-CMGAI	alpha	600	-	-	0.049747	0.938895	0.049196	0.939458	0.0508	0.933375	0.050756	0.938827	0.050632	0.933843	0.06816	0.92724
baron	SCDC	sc-CMGAI	beta	600	-	-	0.053724	0.948301	0.048638	0.94937	0.057809	0.952604	0.047301	0.94975	0.056382	0.9534	0.057877	0.952115
baron	SCDC	sc-CMGAI	delta	600	-	-	0.07498	0.854524	0.080545	0.835873	0.086789	0.828055	0.077012	0.849319	0.085472	0.826627	0.082506	0.82407
baron	SCDC	sc-CMGAI	ductal	600	-	-	0.06678	0.934069	0.062886	0.940235	0.06573	0.942239	0.064726	0.936002	0.061917	0.940534	0.064182	0.936311
baron	SCDC	sc-CMGAI	endothelia	600	-	-	0.066113	0.94184	0.066261	0.939747	0.064701	0.937296	0.070279	0.94139	0.067391	0.934676	0.066903	0.935783
baron	SCDC	sc-CMGAI	gamma	600	-	-	0.072973	0.9056	0.076389	0.900364	0.078826	0.900533	0.074918	0.899165	0.076181	0.905015	0.074487	0.890249
baron	SCDC	sc-CMGAI	macropha	600	-	-	0.047551	0.926578	0.043251	0.926245	0.044828	0.928825	0.043031	0.926163	0.04516	0.926616	0.045114	0.928678
baron	SCDC	sc-CMGAI	quiescent_	600	-	-	0.111736	0.745821	0.107129	0.749247	0.115229	0.736242	0.108997	0.754335	0.113275	0.748775	0.102612	0.770232
baron	SCDC	sc-CMGAI	acinar	700	-	-	0.108095	0.931194	0.108204	0.928404	0.104949	0.930805	0.115677	0.928576	0.114313	0.929006	0.111301	0.928981
baron	SCDC	sc-CMGAI	activated_	700	-	-	0.079375	0.892767	0.078947	0.894204	0.082407	0.891361	0.079808	0.889527	0.083457	0.89336	0.076532	0.89247
baron	SCDC	sc-CMGAI	alpha	700	-	-	0.049953	0.93812	0.049157	0.938778	0.051175	0.932821	0.050478	0.937601	0.050407	0.934083	0.067057	0.927305
baron	SCDC	sc-CMGAI	beta	700	-	-	0.051663	0.948592	0.048687	0.949167	0.055768	0.952677	0.046683	0.950384	0.056633	0.953311	0.05718	0.951341
baron	SCDC	sc-CMGAI	delta	700	-	-	0.0747	0.856049	0.079742	0.839785	0.084792	0.832662	0.077129	0.851108	0.085607	0.826952	0.081576	0.827562
baron	SCDC	sc-CMGAI	ductal	700	-	-	0.066574	0.934342	0.062628	0.940215	0.066418	0.942663	0.063631	0.937429	0.061788	0.940773	0.06343	0.936837
baron	SCDC	sc-CMGAI	endothelia	700	-	-	0.066152	0.942295	0.065518	0.938264	0.064527	0.939016	0.070259	0.941913	0.06727	0.935154	0.067352	0.935848
baron	SCDC	sc-CMGAI	gamma	700	-	-	0.072362	0.905772	0.076447	0.904056	0.077956	0.901462	0.074018	0.899718	0.074977	0.905543	0.074035	0.8906
baron	SCDC	sc-CMGAI	macropha	700	-	-	0.047569	0.926774	0.043208	0.926442	0.044737	0.928623	0.042969	0.926221	0.045152	0.926826	0.044893	0.928794
baron	SCDC	sc-CMGAI	quiescent_	700	-	-	0.111339	0.75077	0.106457	0.75113	0.113528	0.739944	0.1093	0.755573	0.113933	0.745482	0.103461	0.769224
baron	SCDC	sc-CMGAI	acinar	800	-	-	0.108091	0.931356	0.107738	0.928767	0.105739	0.930429	0.114995	0.928915	0.115273	0.928408	0.111867	0.928151
baron	SCDC	sc-CMGAI	activated_	800	-	-	0.078511	0.894242	0.079154	0.893564	0.082179	0.889884	0.080531	0.888886	0.083727	0.893956	0.076233	0.891262
baron	SCDC	sc-CMGAI	alpha	800	-	-	0.049818	0.938963	0.049127	0.939779	0.051118	0.933002	0.050907	0.938351	0.050374	0.934146	0.067366	0.926748
baron	SCDC	sc-CMGAI	beta	800	-	-	0.051877	0.948304	0.048856	0.94921	0.056058	0.952626	0.046676	0.950517	0.057234	0.95325	0.057219	0.951408
baron	SCDC	sc-CMGAI	delta	800	-	-	0.07515	0.854676	0.080341	0.836208	0.086324	0.827978	0.078593	0.847245	0.085986	0.824622	0.082307	0.825918
baron	SCDC	sc-CMGAI	ductal	800	-	-	0.06599	0.934714	0.06331	0.940394	0.066832	0.942145	0.065226	0.936248	0.061479	0.940773	0.063423	0.937239
baron	SCDC	sc-CMGAI	endothelia	800	-	-	0.066164	0.941677	0.066052	0.939713	0.064803	0.939407	0.070624	0.941573	0.068046	0.932344	0.067722	0.93746
baron	SCDC	sc-CMGAI	gamma	800	-	-	0.072346	0.907945	0.076435	0.902166	0.079262	0.902296	0.075135	0.897388	0.07515	0.90635	0.074388	0.891554
baron	SCDC	sc-CMGAI	macropha	800	-	-	0.046964	0.927399	0.043244	0.925651	0.044585	0.928533	0.043253	0.925755	0.044897	0.92757	0.044762	0.928106
baron	SCDC	sc-CMGAI	quiescent_	800	-	-	0.110747	0.751503	0.107266	0.750875	0.11391	0.739192	0.110341	0.750778	0.113862	0.744412	0.103367	0.768743
baron	SCDC	sc-CMGAI	acinar	900	-	-	0.107182	0.93145	0.106026	0.929871	0.10545	0.930169	0.116063	0.928222	0.114245	0.928765	0.111257	0.929801
baron	SCDC	sc-CMGAI	activated_	900	-	-	0.079125	0.892869	0.07933	0.893284	0.082547	0.890267	0.079448	0.890186	0.083888	0.893639	0.076126	0.892191
baron	SCDC	sc-CMGAI	alpha	900	-	-	0.04974	0.93902	0.048813	0.939723	0.051466	0.932595	0.050928	0.938446	0.050544	0.934043	0.067003	0.927581
baron	SCDC	sc-CMGAI	beta	900	-	-	0.051805	0.948423	0.051179	0.948355	0.054799	0.952779	0.046623	0.950284	0.055489	0.95336	0.057596	0.951655
baron	SCDC	sc-CMGAI	delta	900	-	-	0.075163	0.855203	0.079861	0.835624	0.084627	0.831712	0.077627	0.850318	0.084466	0.828813	0.081819	0.82743

baron	SCDC	sc-CMGAI	ductal	900	-	-	0.06599	0.934971	0.063383	0.940809	0.066844	0.942273	0.063403	0.936806	0.061883	0.940784	0.064096	0.935657
baron	SCDC	sc-CMGAI	endothelia	900	-	-	0.0667	0.942255	0.06523	0.94014	0.064657	0.938837	0.06935	0.942291	0.067436	0.934031	0.066649	0.935878
baron	SCDC	sc-CMGAI	gamma	900	-	-	0.072231	0.907663	0.076584	0.90251	0.078679	0.901449	0.074419	0.900079	0.075523	0.906899	0.073817	0.892606
baron	SCDC	sc-CMGAI	macropha	900	-	-	0.046576	0.927437	0.043132	0.926991	0.04472	0.928886	0.042934	0.925962	0.044927	0.927305	0.045286	0.928383
baron	SCDC	sc-CMGAI	quiescent_	900	-	-	0.110932	0.749863	0.107112	0.74824	0.113557	0.739753	0.10844	0.756372	0.114131	0.744241	0.102983	0.769938
baron	SCDC	sc-CMGAI	acinar	1000	-	-	0.108586	0.931091	0.107983	0.928536	0.104736	0.930533	0.115501	0.928838	0.114917	0.928554	0.110035	0.930026
baron	SCDC	sc-CMGAI	activated_	1000	-	-	0.07953	0.892095	0.078746	0.892359	0.082508	0.890417	0.079766	0.889514	0.083499	0.894531	0.077156	0.892893
baron	SCDC	sc-CMGAI	alpha	1000	-	-	0.049908	0.939067	0.049449	0.939018	0.051167	0.932814	0.050478	0.93834	0.050536	0.933845	0.06595	0.927738
baron	SCDC	sc-CMGAI	beta	1000	-	-	0.050957	0.948802	0.048261	0.949149	0.055052	0.952844	0.046714	0.95027	0.056283	0.953283	0.057474	0.951816
baron	SCDC	sc-CMGAI	delta	1000	-	-	0.074993	0.855242	0.080264	0.837401	0.085846	0.830031	0.077475	0.847821	0.084992	0.826645	0.081696	0.82837
baron	SCDC	sc-CMGAI	ductal	1000	-	-	0.065936	0.934351	0.063846	0.940114	0.06704	0.941473	0.064049	0.936544	0.062405	0.939892	0.062772	0.938129
baron	SCDC	sc-CMGAI	endothelia	1000	-	-	0.06566	0.942093	0.065873	0.940066	0.065402	0.938782	0.070411	0.941711	0.067512	0.935515	0.066708	0.935485
baron	SCDC	sc-CMGAI	gamma	1000	-	-	0.07219	0.907499	0.075642	0.903497	0.079048	0.90138	0.075172	0.899566	0.074614	0.907211	0.073852	0.890697
baron	SCDC	sc-CMGAI	macropha	1000	-	-	0.047041	0.926977	0.043118	0.925911	0.044597	0.928771	0.042831	0.926979	0.04509	0.926853	0.045036	0.928835
baron	SCDC	sc-CMGAI	quiescent_	1000	-	-	0.110952	0.750252	0.106618	0.750138	0.114638	0.737299	0.109738	0.755755	0.113014	0.747658	0.103757	0.767382
baron	MuSiC	Control	acinar	0	0.049192	0.959995	-	-	-	-	-	-	-	-	-	-	-	-
baron	MuSiC	Control	activated_	0	0.0614	0.934624	-	-	-	-	-	-	-	-	-	-	-	-
baron	MuSiC	Control	alpha	0	0.053324	0.938037	-	-	-	-	-	-	-	-	-	-	-	-
baron	MuSiC	Control	beta	0	0.058283	0.948088	-	-	-	-	-	-	-	-	-	-	-	-
baron	MuSiC	Control	delta	0	0.056225	0.935443	-	-	-	-	-	-	-	-	-	-	-	-
baron	MuSiC	Control	ductal	0	0.0431	0.959867	-	-	-	-	-	-	-	-	-	-	-	-
baron	MuSiC	Control	endothelia	0	0.039728	0.964961	-	-	-	-	-	-	-	-	-	-	-	-
baron	MuSiC	Control	gamma	0	0.042976	0.953741	-	-	-	-	-	-	-	-	-	-	-	-
baron	MuSiC	Control	macropha	0	0.044874	0.95431	-	-	-	-	-	-	-	-	-	-	-	-
baron	MuSiC	Control	quiescent_	0	0.058885	0.946115	-	-	-	-	-	-	-	-	-	-	-	-
baron	MuSiC	Copula	acinar	100	0.139128	0.923426	-	-	-	-	-	-	-	-	-	-	-	-
baron	MuSiC	Copula	activated_	100	0.079524	0.889604	-	-	-	-	-	-	-	-	-	-	-	-
baron	MuSiC	Copula	alpha	100	0.051057	0.935381	-	-	-	-	-	-	-	-	-	-	-	-
baron	MuSiC	Copula	beta	100	0.055277	0.948756	-	-	-	-	-	-	-	-	-	-	-	-
baron	MuSiC	Copula	delta	100	0.067379	0.882548	-	-	-	-	-	-	-	-	-	-	-	-
baron	MuSiC	Copula	ductal	100	0.072231	0.926227	-	-	-	-	-	-	-	-	-	-	-	-
baron	MuSiC	Copula	endothelia	100	0.071208	0.887761	-	-	-	-	-	-	-	-	-	-	-	-
baron	MuSiC	Copula	gamma	100	0.061251	0.915955	-	-	-	-	-	-	-	-	-	-	-	-
baron	MuSiC	Copula	macropha	100	0.049676	0.919241	-	-	-	-	-	-	-	-	-	-	-	-
baron	MuSiC	Copula	quiescent_	100	0.098636	0.773701	-	-	-	-	-	-	-	-	-	-	-	-
baron	MuSiC	Copula	acinar	200	0.138258	0.924391	-	-	-	-	-	-	-	-	-	-	-	-

baron	MuSiC	Copula	activated_	200	0.078919	0.890211	-	-	-	-	-	-	-	-	-	-	-	-
baron	MuSiC	Copula	alpha	200	0.052249	0.93577	-	-	-	-	-	-	-	-	-	-	-	-
baron	MuSiC	Copula	beta	200	0.050376	0.949505	-	-	-	-	-	-	-	-	-	-	-	-
baron	MuSiC	Copula	delta	200	0.066147	0.886569	-	-	-	-	-	-	-	-	-	-	-	-
baron	MuSiC	Copula	ductal	200	0.070449	0.928811	-	-	-	-	-	-	-	-	-	-	-	-
baron	MuSiC	Copula	endothelia	200	0.066493	0.91442	-	-	-	-	-	-	-	-	-	-	-	-
baron	MuSiC	Copula	gamma	200	0.060736	0.916825	-	-	-	-	-	-	-	-	-	-	-	-
baron	MuSiC	Copula	macropha	200	0.049112	0.921855	-	-	-	-	-	-	-	-	-	-	-	-
baron	MuSiC	Copula	quiescent_	200	0.101943	0.770455	-	-	-	-	-	-	-	-	-	-	-	-
baron	MuSiC	Copula	acinar	300	0.143807	0.921902	-	-	-	-	-	-	-	-	-	-	-	-
baron	MuSiC	Copula	activated_	300	0.078072	0.889521	-	-	-	-	-	-	-	-	-	-	-	-
baron	MuSiC	Copula	alpha	300	0.051212	0.936378	-	-	-	-	-	-	-	-	-	-	-	-
baron	MuSiC	Copula	beta	300	0.050491	0.949316	-	-	-	-	-	-	-	-	-	-	-	-
baron	MuSiC	Copula	delta	300	0.066816	0.88386	-	-	-	-	-	-	-	-	-	-	-	-
baron	MuSiC	Copula	ductal	300	0.074636	0.923176	-	-	-	-	-	-	-	-	-	-	-	-
baron	MuSiC	Copula	endothelia	300	0.068695	0.906791	-	-	-	-	-	-	-	-	-	-	-	-
baron	MuSiC	Copula	gamma	300	0.061159	0.918929	-	-	-	-	-	-	-	-	-	-	-	-
baron	MuSiC	Copula	macropha	300	0.050123	0.918293	-	-	-	-	-	-	-	-	-	-	-	-
baron	MuSiC	Copula	quiescent_	300	0.098684	0.77775	-	-	-	-	-	-	-	-	-	-	-	-
baron	MuSiC	Copula	acinar	400	0.141514	0.922349	-	-	-	-	-	-	-	-	-	-	-	-
baron	MuSiC	Copula	activated_	400	0.079312	0.888991	-	-	-	-	-	-	-	-	-	-	-	-
baron	MuSiC	Copula	alpha	400	0.052017	0.936082	-	-	-	-	-	-	-	-	-	-	-	-
baron	MuSiC	Copula	beta	400	0.053649	0.949157	-	-	-	-	-	-	-	-	-	-	-	-
baron	MuSiC	Copula	delta	400	0.067436	0.881412	-	-	-	-	-	-	-	-	-	-	-	-
baron	MuSiC	Copula	ductal	400	0.071299	0.927298	-	-	-	-	-	-	-	-	-	-	-	-
baron	MuSiC	Copula	endothelia	400	0.067625	0.90467	-	-	-	-	-	-	-	-	-	-	-	-
baron	MuSiC	Copula	gamma	400	0.061521	0.920085	-	-	-	-	-	-	-	-	-	-	-	-
baron	MuSiC	Copula	macropha	400	0.049314	0.919994	-	-	-	-	-	-	-	-	-	-	-	-
baron	MuSiC	Copula	quiescent_	400	0.099696	0.772469	-	-	-	-	-	-	-	-	-	-	-	-
baron	MuSiC	Copula	acinar	500	0.137705	0.924464	-	-	-	-	-	-	-	-	-	-	-	-
baron	MuSiC	Copula	activated_	500	0.080501	0.887777	-	-	-	-	-	-	-	-	-	-	-	-
baron	MuSiC	Copula	alpha	500	0.051945	0.93578	-	-	-	-	-	-	-	-	-	-	-	-
baron	MuSiC	Copula	beta	500	0.054048	0.948686	-	-	-	-	-	-	-	-	-	-	-	-
baron	MuSiC	Copula	delta	500	0.066925	0.883498	-	-	-	-	-	-	-	-	-	-	-	-
baron	MuSiC	Copula	ductal	500	0.072599	0.927694	-	-	-	-	-	-	-	-	-	-	-	-
baron	MuSiC	Copula	endothelia	500	0.068665	0.905326	-	-	-	-	-	-	-	-	-	-	-	-

baron	MuSiC	Copula	gamma	500	0.061316	0.917593	-	-	-	-	-	-	-	-	-	-	-
baron	MuSiC	Copula	macropha	500	0.049541	0.920594	-	-	-	-	-	-	-	-	-	-	-
baron	MuSiC	Copula	quiescent_	500	0.102001	0.765252	-	-	-	-	-	-	-	-	-	-	-
baron	MuSiC	Copula	acinar	600	0.140126	0.923341	-	-	-	-	-	-	-	-	-	-	-
baron	MuSiC	Copula	activated_	600	0.079028	0.888785	-	-	-	-	-	-	-	-	-	-	-
baron	MuSiC	Copula	alpha	600	0.05063	0.936624	-	-	-	-	-	-	-	-	-	-	-
baron	MuSiC	Copula	beta	600	0.053748	0.949217	-	-	-	-	-	-	-	-	-	-	-
baron	MuSiC	Copula	delta	600	0.067388	0.881603	-	-	-	-	-	-	-	-	-	-	-
baron	MuSiC	Copula	ductal	600	0.074369	0.925696	-	-	-	-	-	-	-	-	-	-	-
baron	MuSiC	Copula	endothelia	600	0.066753	0.912571	-	-	-	-	-	-	-	-	-	-	-
baron	MuSiC	Copula	gamma	600	0.061064	0.918521	-	-	-	-	-	-	-	-	-	-	-
baron	MuSiC	Copula	macropha	600	0.049378	0.919759	-	-	-	-	-	-	-	-	-	-	-
baron	MuSiC	Copula	quiescent_	600	0.098793	0.777346	-	-	-	-	-	-	-	-	-	-	-
baron	MuSiC	Copula	acinar	700	0.139379	0.923999	-	-	-	-	-	-	-	-	-	-	-
baron	MuSiC	Copula	activated_	700	0.079416	0.889873	-	-	-	-	-	-	-	-	-	-	-
baron	MuSiC	Copula	alpha	700	0.051206	0.936578	-	-	-	-	-	-	-	-	-	-	-
baron	MuSiC	Copula	beta	700	0.05298	0.949123	-	-	-	-	-	-	-	-	-	-	-
baron	MuSiC	Copula	delta	700	0.066915	0.883375	-	-	-	-	-	-	-	-	-	-	-
baron	MuSiC	Copula	ductal	700	0.073199	0.927343	-	-	-	-	-	-	-	-	-	-	-
baron	MuSiC	Copula	endothelia	700	0.066534	0.910818	-	-	-	-	-	-	-	-	-	-	-
baron	MuSiC	Copula	gamma	700	0.061272	0.918796	-	-	-	-	-	-	-	-	-	-	-
baron	MuSiC	Copula	macropha	700	0.049	0.919981	-	-	-	-	-	-	-	-	-	-	-
baron	MuSiC	Copula	quiescent_	700	0.099113	0.77644	-	-	-	-	-	-	-	-	-	-	-
baron	MuSiC	Copula	acinar	800	0.13855	0.924121	-	-	-	-	-	-	-	-	-	-	-
baron	MuSiC	Copula	activated_	800	0.08032	0.888695	-	-	-	-	-	-	-	-	-	-	-
baron	MuSiC	Copula	alpha	800	0.051641	0.935968	-	-	-	-	-	-	-	-	-	-	-
baron	MuSiC	Copula	beta	800	0.053535	0.948904	-	-	-	-	-	-	-	-	-	-	-
baron	MuSiC	Copula	delta	800	0.066306	0.88576	-	-	-	-	-	-	-	-	-	-	-
baron	MuSiC	Copula	ductal	800	0.072428	0.927566	-	-	-	-	-	-	-	-	-	-	-
baron	MuSiC	Copula	endothelia	800	0.067819	0.906126	-	-	-	-	-	-	-	-	-	-	-
baron	MuSiC	Copula	gamma	800	0.060774	0.919497	-	-	-	-	-	-	-	-	-	-	-
baron	MuSiC	Copula	macropha	800	0.04942	0.920607	-	-	-	-	-	-	-	-	-	-	-
baron	MuSiC	Copula	quiescent_	800	0.100967	0.76856	-	-	-	-	-	-	-	-	-	-	-
baron	MuSiC	Copula	acinar	900	0.139135	0.923912	-	-	-	-	-	-	-	-	-	-	-
baron	MuSiC	Copula	activated_	900	0.079523	0.888146	-	-	-	-	-	-	-	-	-	-	-
baron	MuSiC	Copula	alpha	900	0.051513	0.936207	-	-	-	-	-	-	-	-	-	-	-

baron	MuSiC	Copula	beta	900	0.050382	0.949533	-	-	-	-	-	-	-	-	-	-	-	-
baron	MuSiC	Copula	delta	900	0.066337	0.885578	-	-	-	-	-	-	-	-	-	-	-	-
baron	MuSiC	Copula	ductal	900	0.072032	0.928333	-	-	-	-	-	-	-	-	-	-	-	-
baron	MuSiC	Copula	endothelia	900	0.067491	0.909745	-	-	-	-	-	-	-	-	-	-	-	-
baron	MuSiC	Copula	gamma	900	0.060953	0.917667	-	-	-	-	-	-	-	-	-	-	-	-
baron	MuSiC	Copula	macropha	900	0.049422	0.919909	-	-	-	-	-	-	-	-	-	-	-	-
baron	MuSiC	Copula	quiescent_	900	0.101432	0.770266	-	-	-	-	-	-	-	-	-	-	-	-
baron	MuSiC	Copula	acinar	1000	0.140151	0.923087	-	-	-	-	-	-	-	-	-	-	-	-
baron	MuSiC	Copula	activated_	1000	0.079256	0.888455	-	-	-	-	-	-	-	-	-	-	-	-
baron	MuSiC	Copula	alpha	1000	0.051092	0.936478	-	-	-	-	-	-	-	-	-	-	-	-
baron	MuSiC	Copula	beta	1000	0.054372	0.948796	-	-	-	-	-	-	-	-	-	-	-	-
baron	MuSiC	Copula	delta	1000	0.067069	0.882789	-	-	-	-	-	-	-	-	-	-	-	-
baron	MuSiC	Copula	ductal	1000	0.072342	0.925904	-	-	-	-	-	-	-	-	-	-	-	-
baron	MuSiC	Copula	endothelia	1000	0.067869	0.906029	-	-	-	-	-	-	-	-	-	-	-	-
baron	MuSiC	Copula	gamma	1000	0.061033	0.918911	-	-	-	-	-	-	-	-	-	-	-	-
baron	MuSiC	Copula	macropha	1000	0.049372	0.920148	-	-	-	-	-	-	-	-	-	-	-	-
baron	MuSiC	Copula	quiescent_	1000	0.100009	0.771791	-	-	-	-	-	-	-	-	-	-	-	-
baron	MuSiC	CTGAN	acinar	100	-	-	0.138642	0.920869	0.13165	0.925292	0.1271	0.927953	0.130287	0.926124	0.125974	0.928155	0.135228	0.922258
baron	MuSiC	CTGAN	activated_	100	-	-	0.086585	0.88561	0.08301	0.891487	0.075697	0.891336	0.089287	0.882415	0.078465	0.891634	0.081362	0.891393
baron	MuSiC	CTGAN	alpha	100	-	-	0.048591	0.938381	0.053339	0.934018	0.049794	0.936487	0.050834	0.935643	0.051477	0.935032	0.050733	0.935957
baron	MuSiC	CTGAN	beta	100	-	-	0.056516	0.948664	0.064939	0.947836	0.062617	0.948286	0.047749	0.952704	0.063509	0.9478	0.046034	0.951268
baron	MuSiC	CTGAN	delta	100	-	-	0.07196	0.870275	0.072233	0.865745	0.071309	0.874671	0.088	0.851365	0.07037	0.872842	0.075801	0.867484
baron	MuSiC	CTGAN	ductal	100	-	-	0.058901	0.938426	0.069743	0.930541	0.063558	0.937536	0.063864	0.936622	0.069299	0.930673	0.065761	0.934286
baron	MuSiC	CTGAN	endothelia	100	-	-	0.070942	0.891884	0.06225	0.930133	0.071136	0.881686	0.063973	0.925305	0.06985	0.896316	0.064944	0.912055
baron	MuSiC	CTGAN	gamma	100	-	-	0.06416	0.912035	0.066941	0.916773	0.065446	0.907617	0.068763	0.920853	0.067374	0.889626	0.063476	0.913559
baron	MuSiC	CTGAN	macropha	100	-	-	0.049614	0.917097	0.051932	0.923668	0.049036	0.923975	0.04988	0.926964	0.051372	0.921397	0.049267	0.922156
baron	MuSiC	CTGAN	quiescent_	100	-	-	0.111672	0.727081	0.104676	0.750931	0.093042	0.796382	0.10959	0.740507	0.099503	0.770841	0.09862	0.768167
baron	MuSiC	CTGAN	acinar	200	-	-	0.139614	0.921405	0.133367	0.924487	0.132671	0.925958	0.129209	0.926426	0.127034	0.929009	0.134843	0.922609
baron	MuSiC	CTGAN	activated_	200	-	-	0.09087	0.880602	0.084774	0.88729	0.075242	0.893107	0.085187	0.885853	0.079885	0.891472	0.081989	0.887568
baron	MuSiC	CTGAN	alpha	200	-	-	0.048869	0.937802	0.052456	0.934044	0.049665	0.93676	0.05224	0.934154	0.05283	0.934105	0.050542	0.93652
baron	MuSiC	CTGAN	beta	200	-	-	0.064708	0.947605	0.06228	0.947707	0.064051	0.947779	0.047178	0.952886	0.058672	0.949092	0.046604	0.950776
baron	MuSiC	CTGAN	delta	200	-	-	0.071022	0.868606	0.071372	0.872203	0.075337	0.86341	0.084603	0.85977	0.069356	0.875453	0.07575	0.869706
baron	MuSiC	CTGAN	ductal	200	-	-	0.05943	0.937288	0.061542	0.934389	0.065266	0.935446	0.063188	0.937748	0.071124	0.927375	0.066558	0.932981
baron	MuSiC	CTGAN	endothelia	200	-	-	0.069008	0.907894	0.063642	0.923579	0.069016	0.89047	0.064667	0.913454	0.06708	0.913186	0.059968	0.930404
baron	MuSiC	CTGAN	gamma	200	-	-	0.064341	0.908396	0.064743	0.919716	0.066855	0.912072	0.065928	0.91979	0.065532	0.900576	0.064311	0.917937
baron	MuSiC	CTGAN	macropha	200	-	-	0.050614	0.917177	0.0512	0.924912	0.049795	0.923636	0.051256	0.925384	0.051096	0.922864	0.050113	0.919278

baron	MuSiC	CTGAN	quiescent_	200	-	-	0.118955	0.702052	0.108132	0.741686	0.090862	0.806362	0.102352	0.764938	0.100808	0.76996	0.099236	0.770279
baron	MuSiC	CTGAN	acinar	300	-	-	0.139065	0.921228	0.134554	0.924373	0.130345	0.927432	0.130553	0.924674	0.124397	0.930023	0.137001	0.921876
baron	MuSiC	CTGAN	activated_	300	-	-	0.087281	0.884204	0.082553	0.888746	0.073184	0.894198	0.087148	0.884508	0.079546	0.891043	0.080932	0.887921
baron	MuSiC	CTGAN	alpha	300	-	-	0.049064	0.93709	0.051227	0.93513	0.049367	0.936929	0.05078	0.935585	0.05213	0.934719	0.052388	0.93534
baron	MuSiC	CTGAN	beta	300	-	-	0.066873	0.946865	0.062597	0.947542	0.062754	0.947937	0.048596	0.953279	0.062903	0.948388	0.047847	0.950037
baron	MuSiC	CTGAN	delta	300	-	-	0.071043	0.868222	0.072328	0.868387	0.07215	0.872572	0.089611	0.852352	0.070333	0.871755	0.07709	0.866858
baron	MuSiC	CTGAN	ductal	300	-	-	0.060726	0.935698	0.064104	0.933916	0.066176	0.933429	0.065278	0.937419	0.071104	0.929222	0.063721	0.934797
baron	MuSiC	CTGAN	endothelia	300	-	-	0.070059	0.902497	0.06318	0.926384	0.070536	0.883649	0.061485	0.928283	0.068287	0.910004	0.063031	0.915395
baron	MuSiC	CTGAN	gamma	300	-	-	0.06466	0.90824	0.064127	0.91776	0.064921	0.91138	0.067567	0.920581	0.066139	0.898903	0.065267	0.921456
baron	MuSiC	CTGAN	macropha	300	-	-	0.05029	0.918405	0.051508	0.923727	0.05004	0.924133	0.051611	0.924367	0.051352	0.92324	0.050073	0.917632
baron	MuSiC	CTGAN	quiescent_	300	-	-	0.11492	0.719503	0.10543	0.752352	0.089998	0.812594	0.104874	0.757296	0.101163	0.768287	0.097035	0.776916
baron	MuSiC	CTGAN	acinar	400	-	-	0.137894	0.921637	0.133789	0.924576	0.131931	0.925876	0.129255	0.925613	0.123625	0.929935	0.136217	0.921873
baron	MuSiC	CTGAN	activated_	400	-	-	0.088567	0.883098	0.082843	0.889922	0.075238	0.894245	0.086564	0.884436	0.078877	0.891569	0.082883	0.887404
baron	MuSiC	CTGAN	alpha	400	-	-	0.048631	0.938443	0.051899	0.934787	0.049499	0.937006	0.051303	0.935297	0.053171	0.934539	0.050472	0.936734
baron	MuSiC	CTGAN	beta	400	-	-	0.065515	0.947758	0.061396	0.948316	0.062939	0.948527	0.04812	0.953041	0.064373	0.94848	0.047273	0.950567
baron	MuSiC	CTGAN	delta	400	-	-	0.071503	0.867125	0.070876	0.872992	0.072624	0.872596	0.087305	0.852555	0.070379	0.870846	0.075476	0.869443
baron	MuSiC	CTGAN	ductal	400	-	-	0.061306	0.93575	0.064373	0.93361	0.06584	0.933051	0.065171	0.935872	0.072329	0.928251	0.066468	0.932836
baron	MuSiC	CTGAN	endothelia	400	-	-	0.070206	0.90143	0.063611	0.92095	0.070187	0.885698	0.062882	0.923268	0.068217	0.908505	0.060514	0.92446
baron	MuSiC	CTGAN	gamma	400	-	-	0.064391	0.907356	0.063697	0.920333	0.065421	0.90891	0.067887	0.922269	0.066459	0.898186	0.063561	0.920656
baron	MuSiC	CTGAN	macropha	400	-	-	0.050062	0.91741	0.051653	0.924374	0.049521	0.924553	0.051937	0.925061	0.050944	0.922277	0.049883	0.919847
baron	MuSiC	CTGAN	quiescent_	400	-	-	0.114383	0.718741	0.105284	0.752737	0.090537	0.808623	0.106024	0.755523	0.100625	0.769651	0.098725	0.769373
baron	MuSiC	CTGAN	acinar	500	-	-	0.138436	0.921054	0.135596	0.924371	0.132557	0.925958	0.130155	0.925485	0.125496	0.929312	0.137179	0.921602
baron	MuSiC	CTGAN	activated_	500	-	-	0.088435	0.882326	0.082345	0.889862	0.074852	0.892686	0.086201	0.885057	0.078064	0.892755	0.082541	0.886837
baron	MuSiC	CTGAN	alpha	500	-	-	0.049083	0.937066	0.052808	0.93445	0.04988	0.936333	0.050657	0.935466	0.051773	0.934698	0.051593	0.936059
baron	MuSiC	CTGAN	beta	500	-	-	0.066813	0.947391	0.062034	0.94782	0.06348	0.948149	0.049089	0.952607	0.064076	0.948586	0.046897	0.95034
baron	MuSiC	CTGAN	delta	500	-	-	0.070765	0.869323	0.070743	0.872889	0.07347	0.869018	0.087872	0.853453	0.070125	0.872162	0.075712	0.871259
baron	MuSiC	CTGAN	ductal	500	-	-	0.060829	0.935209	0.063283	0.933275	0.065385	0.934042	0.066243	0.934816	0.069839	0.929451	0.066385	0.933594
baron	MuSiC	CTGAN	endothelia	500	-	-	0.07	0.901634	0.063532	0.921375	0.070499	0.883672	0.062924	0.921796	0.067095	0.912393	0.060171	0.927278
baron	MuSiC	CTGAN	gamma	500	-	-	0.064275	0.90807	0.064299	0.92014	0.065849	0.909305	0.066935	0.920071	0.066141	0.896892	0.063352	0.921862
baron	MuSiC	CTGAN	macropha	500	-	-	0.050022	0.916879	0.051933	0.923243	0.049595	0.923688	0.05192	0.923753	0.050554	0.923521	0.049733	0.920662
baron	MuSiC	CTGAN	quiescent_	500	-	-	0.115023	0.714319	0.104764	0.754535	0.090955	0.805196	0.103735	0.762557	0.099358	0.774952	0.097988	0.770568
baron	MuSiC	CTGAN	acinar	600	-	-	0.139666	0.919915	0.135196	0.924123	0.132592	0.925724	0.130628	0.925606	0.126814	0.9286	0.136295	0.921955
baron	MuSiC	CTGAN	activated_	600	-	-	0.087938	0.883041	0.084146	0.886206	0.0747	0.893975	0.08649	0.885375	0.080567	0.8895	0.083033	0.887865
baron	MuSiC	CTGAN	alpha	600	-	-	0.048928	0.937655	0.051376	0.935019	0.049522	0.936994	0.05116	0.935386	0.051608	0.934804	0.051269	0.93592
baron	MuSiC	CTGAN	beta	600	-	-	0.065426	0.947557	0.061849	0.948219	0.062254	0.948246	0.048638	0.953074	0.0647	0.948175	0.047397	0.950213
baron	MuSiC	CTGAN	delta	600	-	-	0.071153	0.868285	0.071859	0.869835	0.071362	0.874925	0.086836	0.854137	0.07007	0.872364	0.077635	0.866527

baron	MuSiC	CTGAN	ductal	600	-	-	0.060224	0.935827	0.063853	0.933978	0.065186	0.934115	0.066527	0.935202	0.069616	0.929461	0.067036	0.932314
baron	MuSiC	CTGAN	endothelia	600	-	-	0.070867	0.894211	0.063061	0.926768	0.071896	0.877045	0.063023	0.920476	0.068205	0.908641	0.062218	0.919774
baron	MuSiC	CTGAN	gamma	600	-	-	0.064411	0.90621	0.064287	0.917417	0.064514	0.910651	0.067327	0.920117	0.065784	0.898747	0.065398	0.921319
baron	MuSiC	CTGAN	macropha	600	-	-	0.050192	0.916997	0.051351	0.922787	0.049972	0.92426	0.05142	0.925476	0.051153	0.922609	0.05017	0.920841
baron	MuSiC	CTGAN	quiescent_	600	-	-	0.11443	0.717114	0.106548	0.748706	0.090888	0.806005	0.103991	0.75958	0.103002	0.759702	0.098637	0.767199
baron	MuSiC	CTGAN	acinar	700	-	-	0.139795	0.920525	0.134764	0.924591	0.132514	0.926065	0.130376	0.925587	0.125748	0.92892	0.136083	0.921775
baron	MuSiC	CTGAN	activated_	700	-	-	0.088809	0.882116	0.082004	0.888394	0.074768	0.892918	0.086646	0.884765	0.078765	0.891733	0.08223	0.887666
baron	MuSiC	CTGAN	alpha	700	-	-	0.049064	0.937078	0.051126	0.935148	0.04936	0.937255	0.05085	0.935232	0.051749	0.934858	0.050789	0.936543
baron	MuSiC	CTGAN	beta	700	-	-	0.066801	0.947033	0.062263	0.947979	0.063447	0.948037	0.04782	0.953211	0.065217	0.948196	0.048322	0.950086
baron	MuSiC	CTGAN	delta	700	-	-	0.071149	0.868132	0.072043	0.86991	0.0729	0.870559	0.086319	0.855462	0.070256	0.871634	0.075006	0.871704
baron	MuSiC	CTGAN	ductal	700	-	-	0.059772	0.935904	0.064699	0.93325	0.065323	0.932799	0.065593	0.937266	0.071418	0.92873	0.066154	0.933541
baron	MuSiC	CTGAN	endothelia	700	-	-	0.070183	0.901554	0.063504	0.92355	0.071374	0.879529	0.062752	0.922301	0.067762	0.911062	0.060618	0.924703
baron	MuSiC	CTGAN	gamma	700	-	-	0.064355	0.907535	0.0643	0.917953	0.064923	0.910507	0.066517	0.920907	0.065917	0.899878	0.063483	0.921737
baron	MuSiC	CTGAN	macropha	700	-	-	0.050313	0.916909	0.051595	0.923846	0.049956	0.923061	0.051347	0.924696	0.050697	0.922986	0.04997	0.92038
baron	MuSiC	CTGAN	quiescent_	700	-	-	0.116441	0.712101	0.104026	0.757681	0.090819	0.807093	0.104327	0.758388	0.09986	0.771114	0.097705	0.772144
baron	MuSiC	CTGAN	acinar	800	-	-	0.139512	0.920459	0.134749	0.924664	0.132021	0.926401	0.129692	0.925251	0.126117	0.928833	0.134942	0.922488
baron	MuSiC	CTGAN	activated_	800	-	-	0.087816	0.88337	0.082395	0.889714	0.074041	0.894199	0.086511	0.88404	0.079015	0.889835	0.08274	0.885122
baron	MuSiC	CTGAN	alpha	800	-	-	0.049124	0.936911	0.051476	0.935052	0.049745	0.93695	0.051037	0.935393	0.052027	0.934341	0.051902	0.935819
baron	MuSiC	CTGAN	beta	800	-	-	0.067685	0.946785	0.061849	0.947892	0.063275	0.948266	0.048005	0.953125	0.06372	0.948416	0.048138	0.95034
baron	MuSiC	CTGAN	delta	800	-	-	0.070665	0.869882	0.071047	0.872025	0.072853	0.871486	0.085668	0.855581	0.069959	0.87302	0.076544	0.867899
baron	MuSiC	CTGAN	ductal	800	-	-	0.060489	0.93521	0.064399	0.932963	0.065315	0.933666	0.06626	0.936277	0.070562	0.92844	0.06777	0.932182
baron	MuSiC	CTGAN	endothelia	800	-	-	0.069555	0.904459	0.063374	0.922653	0.070751	0.882553	0.061648	0.925963	0.067842	0.908821	0.060656	0.926362
baron	MuSiC	CTGAN	gamma	800	-	-	0.064576	0.908897	0.063428	0.919118	0.064968	0.912095	0.066355	0.919631	0.065981	0.897314	0.064818	0.923479
baron	MuSiC	CTGAN	macropha	800	-	-	0.050379	0.917022	0.051262	0.92328	0.049799	0.923339	0.051239	0.924275	0.050722	0.922103	0.05008	0.920963
baron	MuSiC	CTGAN	quiescent_	800	-	-	0.114851	0.720082	0.104796	0.754798	0.090103	0.810258	0.104008	0.761036	0.10078	0.767215	0.098777	0.768305
baron	MuSiC	CTGAN	acinar	900	-	-	0.138344	0.920696	0.135104	0.924568	0.132184	0.925768	0.130647	0.925083	0.126192	0.928746	0.135501	0.922117
baron	MuSiC	CTGAN	activated_	900	-	-	0.08802	0.881858	0.082255	0.890197	0.073772	0.894557	0.08541	0.885826	0.078729	0.892072	0.082736	0.886747
baron	MuSiC	CTGAN	alpha	900	-	-	0.04886	0.937655	0.050886	0.935616	0.04936	0.937253	0.0504	0.936181	0.052277	0.934566	0.051965	0.935905
baron	MuSiC	CTGAN	beta	900	-	-	0.066412	0.947369	0.061545	0.948145	0.062647	0.948178	0.04861	0.952873	0.064055	0.948393	0.047003	0.95028
baron	MuSiC	CTGAN	delta	900	-	-	0.071233	0.86799	0.071634	0.87113	0.072842	0.872292	0.087544	0.855075	0.070203	0.872078	0.07486	0.872145
baron	MuSiC	CTGAN	ductal	900	-	-	0.06033	0.936387	0.065094	0.933223	0.065138	0.933973	0.064856	0.936197	0.07016	0.929659	0.067075	0.933086
baron	MuSiC	CTGAN	endothelia	900	-	-	0.070187	0.897047	0.063479	0.923307	0.071715	0.8779	0.062578	0.923164	0.067379	0.912057	0.05997	0.928659
baron	MuSiC	CTGAN	gamma	900	-	-	0.064339	0.908172	0.064144	0.917957	0.065286	0.91033	0.066736	0.919566	0.066166	0.89696	0.063067	0.921958
baron	MuSiC	CTGAN	macropha	900	-	-	0.050173	0.917509	0.051482	0.923272	0.049678	0.923705	0.050878	0.924321	0.050916	0.92213	0.049753	0.920713
baron	MuSiC	CTGAN	quiescent_	900	-	-	0.11392	0.718707	0.104098	0.75745	0.090217	0.810049	0.102732	0.766377	0.099847	0.772396	0.098237	0.772711
baron	MuSiC	CTGAN	acinar	1000	-	-	0.139161	0.920459	0.134386	0.924297	0.132503	0.926298	0.129397	0.925663	0.126813	0.928596	0.135347	0.921934

baron	MuSiC	CTGAN	activated_	1000	-	-	0.088271	0.882392	0.08328	0.888199	0.074622	0.894313	0.087082	0.883599	0.079198	0.890389	0.082386	0.887173
baron	MuSiC	CTGAN	alpha	1000	-	-	0.048869	0.937593	0.052143	0.934839	0.049925	0.936636	0.051649	0.935136	0.052504	0.934356	0.051056	0.936386
baron	MuSiC	CTGAN	beta	1000	-	-	0.065048	0.94793	0.062488	0.947735	0.063299	0.948206	0.048027	0.952875	0.063988	0.948296	0.048372	0.950271
baron	MuSiC	CTGAN	delta	1000	-	-	0.070924	0.869236	0.071207	0.871496	0.072838	0.871587	0.087315	0.853942	0.070245	0.87184	0.075662	0.869606
baron	MuSiC	CTGAN	ductal	1000	-	-	0.060363	0.936081	0.065068	0.932338	0.064634	0.934499	0.066595	0.936233	0.070474	0.929097	0.066422	0.933684
baron	MuSiC	CTGAN	endothelia	1000	-	-	0.070499	0.898092	0.063707	0.92175	0.070584	0.883406	0.06262	0.921037	0.066922	0.914224	0.061208	0.923216
baron	MuSiC	CTGAN	gamma	1000	-	-	0.064281	0.908549	0.064325	0.920027	0.065574	0.911011	0.067766	0.922694	0.066223	0.895439	0.063981	0.921866
baron	MuSiC	CTGAN	macropha	1000	-	-	0.050409	0.917318	0.051954	0.923762	0.049944	0.92373	0.051754	0.924382	0.050984	0.921317	0.050154	0.91964
baron	MuSiC	CTGAN	quiescent_	1000	-	-	0.115038	0.71772	0.105458	0.751213	0.090896	0.805486	0.104187	0.758766	0.100349	0.769247	0.098318	0.769848
baron	MuSiC	TVAE	acinar	100	-	-	0.122656	0.924274	0.1148	0.925714	0.111683	0.929524	0.117492	0.925798	0.11739	0.923983	0.111529	0.92855
baron	MuSiC	TVAE	activated_	100	-	-	0.078137	0.894832	0.069888	0.898146	0.075528	0.887761	0.071163	0.890258	0.078724	0.889803	0.074385	0.89472
baron	MuSiC	TVAE	alpha	100	-	-	0.049431	0.93756	0.054299	0.93236	0.05282	0.934478	0.063465	0.928262	0.051477	0.936737	0.05322	0.934601
baron	MuSiC	TVAE	beta	100	-	-	0.044678	0.954202	0.044416	0.954737	0.044824	0.954101	0.045462	0.952609	0.0446	0.954572	0.04454	0.954627
baron	MuSiC	TVAE	delta	100	-	-	0.069443	0.883393	0.067144	0.886222	0.064129	0.894002	0.065523	0.890714	0.066733	0.887371	0.065576	0.890626
baron	MuSiC	TVAE	ductal	100	-	-	0.09112	0.933644	0.093921	0.936276	0.100029	0.935495	0.090387	0.931859	0.094542	0.935277	0.098003	0.933438
baron	MuSiC	TVAE	endothelia	100	-	-	0.061156	0.938493	0.057522	0.939651	0.061716	0.941603	0.058973	0.939417	0.061777	0.935972	0.059867	0.940522
baron	MuSiC	TVAE	gamma	100	-	-	0.06626	0.917382	0.065091	0.914612	0.063277	0.918806	0.065255	0.910575	0.066579	0.912393	0.063625	0.919016
baron	MuSiC	TVAE	macropha	100	-	-	0.049674	0.921881	0.049674	0.918027	0.046454	0.922642	0.051806	0.91633	0.05081	0.915692	0.04888	0.917725
baron	MuSiC	TVAE	quiescent_	100	-	-	0.096221	0.804341	0.087796	0.843353	0.092652	0.817699	0.093709	0.814202	0.096923	0.803817	0.091801	0.822033
baron	MuSiC	TVAE	acinar	200	-	-	0.12097	0.92526	0.114078	0.927314	0.111669	0.928576	0.116811	0.925836	0.116069	0.924752	0.112124	0.927797
baron	MuSiC	TVAE	activated_	200	-	-	0.077446	0.894161	0.070657	0.896041	0.075555	0.888594	0.0699	0.892072	0.078539	0.891386	0.073571	0.896339
baron	MuSiC	TVAE	alpha	200	-	-	0.050087	0.936839	0.054638	0.93168	0.053339	0.933493	0.059438	0.930046	0.050701	0.937806	0.052948	0.934711
baron	MuSiC	TVAE	beta	200	-	-	0.044653	0.954053	0.044247	0.955035	0.04494	0.954084	0.045168	0.953316	0.044437	0.954701	0.044506	0.954591
baron	MuSiC	TVAE	delta	200	-	-	0.069322	0.881941	0.067219	0.885363	0.063631	0.89595	0.065169	0.890929	0.065819	0.890099	0.067336	0.885186
baron	MuSiC	TVAE	ductal	200	-	-	0.092103	0.934127	0.09362	0.936098	0.100378	0.935138	0.090994	0.932612	0.093281	0.937126	0.099108	0.933677
baron	MuSiC	TVAE	endothelia	200	-	-	0.060969	0.93713	0.057551	0.940394	0.061319	0.943207	0.059402	0.939255	0.061698	0.937062	0.059193	0.941172
baron	MuSiC	TVAE	gamma	200	-	-	0.066291	0.921142	0.064356	0.915537	0.062912	0.917917	0.065402	0.91385	0.065588	0.913741	0.064923	0.918178
baron	MuSiC	TVAE	macropha	200	-	-	0.050063	0.922145	0.049726	0.918114	0.046857	0.921	0.05154	0.916282	0.050975	0.915248	0.048644	0.917786
baron	MuSiC	TVAE	quiescent_	200	-	-	0.096416	0.802806	0.088438	0.839724	0.093471	0.814808	0.092851	0.820639	0.097977	0.798628	0.091035	0.826949
baron	MuSiC	TVAE	acinar	300	-	-	0.122378	0.924587	0.114164	0.926923	0.111109	0.929929	0.115664	0.925991	0.116063	0.924729	0.112308	0.928141
baron	MuSiC	TVAE	activated_	300	-	-	0.078213	0.894098	0.070308	0.896969	0.076189	0.889124	0.069198	0.892296	0.078682	0.891326	0.074231	0.895784
baron	MuSiC	TVAE	alpha	300	-	-	0.0501	0.93685	0.053689	0.932772	0.05224	0.934876	0.061147	0.929186	0.049994	0.938728	0.05298	0.934498
baron	MuSiC	TVAE	beta	300	-	-	0.04466	0.954101	0.04428	0.954877	0.044861	0.954195	0.045277	0.953035	0.044526	0.954652	0.044569	0.95448
baron	MuSiC	TVAE	delta	300	-	-	0.068746	0.883911	0.066682	0.887487	0.06407	0.894632	0.065389	0.889959	0.066455	0.888519	0.066062	0.889179
baron	MuSiC	TVAE	ductal	300	-	-	0.090935	0.934553	0.092508	0.937052	0.101745	0.93401	0.090892	0.933036	0.094409	0.936154	0.10053	0.933513
baron	MuSiC	TVAE	endothelia	300	-	-	0.060936	0.937423	0.057133	0.939915	0.061205	0.942822	0.059113	0.93874	0.061953	0.936374	0.059489	0.940822

baron	MuSiC	TVAE	gamma	300	-	-	0.065862	0.919124	0.064638	0.913927	0.063241	0.918139	0.064988	0.913349	0.065612	0.913367	0.063738	0.919555
baron	MuSiC	TVAE	macropha	300	-	-	0.049827	0.921808	0.049815	0.918128	0.047117	0.920363	0.051907	0.916627	0.050966	0.915238	0.048698	0.918782
baron	MuSiC	TVAE	quiescent_	300	-	-	0.096843	0.80209	0.088201	0.842196	0.094237	0.810317	0.091075	0.827855	0.097421	0.801459	0.090894	0.825845
baron	MuSiC	TVAE	acinar	400	-	-	0.121194	0.924537	0.114074	0.92683	0.111135	0.92946	0.116095	0.925861	0.11499	0.924815	0.111291	0.928318
baron	MuSiC	TVAE	activated_	400	-	-	0.077707	0.894987	0.070877	0.89686	0.076006	0.88829	0.070327	0.891511	0.078537	0.890896	0.073976	0.89546
baron	MuSiC	TVAE	alpha	400	-	-	0.049766	0.937339	0.054024	0.932417	0.052007	0.935251	0.060945	0.929083	0.050419	0.938059	0.053184	0.934432
baron	MuSiC	TVAE	beta	400	-	-	0.044698	0.954119	0.044299	0.954903	0.044836	0.954139	0.045171	0.95321	0.044473	0.954753	0.044603	0.95447
baron	MuSiC	TVAE	delta	400	-	-	0.068808	0.883512	0.067203	0.886065	0.063776	0.895656	0.065083	0.891705	0.066716	0.887792	0.066193	0.888971
baron	MuSiC	TVAE	ductal	400	-	-	0.091697	0.934304	0.092743	0.936649	0.102273	0.934383	0.091066	0.933264	0.09598	0.936706	0.099766	0.934046
baron	MuSiC	TVAE	endothelia	400	-	-	0.061173	0.936872	0.057638	0.93993	0.060683	0.943361	0.05945	0.937924	0.06148	0.937535	0.059371	0.940714
baron	MuSiC	TVAE	gamma	400	-	-	0.065654	0.920439	0.064847	0.913056	0.062931	0.918476	0.065234	0.913178	0.066215	0.913835	0.064217	0.918976
baron	MuSiC	TVAE	macropha	400	-	-	0.049958	0.921185	0.050387	0.917529	0.046769	0.921434	0.051844	0.917046	0.050899	0.915436	0.049067	0.918709
baron	MuSiC	TVAE	quiescent_	400	-	-	0.096389	0.805033	0.088771	0.838812	0.093739	0.811958	0.092257	0.820848	0.096855	0.802844	0.09085	0.825824
baron	MuSiC	TVAE	acinar	500	-	-	0.121601	0.924478	0.112921	0.926794	0.110722	0.929609	0.117107	0.925368	0.115582	0.924878	0.111786	0.927961
baron	MuSiC	TVAE	activated_	500	-	-	0.077972	0.895931	0.071252	0.8962	0.076154	0.888464	0.06999	0.891615	0.07928	0.890417	0.074104	0.896025
baron	MuSiC	TVAE	alpha	500	-	-	0.049589	0.937485	0.054107	0.932299	0.052381	0.934655	0.061511	0.928862	0.050562	0.938076	0.052973	0.93474
baron	MuSiC	TVAE	beta	500	-	-	0.044575	0.954301	0.044253	0.954953	0.044933	0.954019	0.04522	0.953197	0.044616	0.954532	0.044496	0.954646
baron	MuSiC	TVAE	delta	500	-	-	0.068463	0.885017	0.066717	0.887157	0.064064	0.894461	0.065406	0.889892	0.066736	0.886987	0.066053	0.889367
baron	MuSiC	TVAE	ductal	500	-	-	0.090442	0.9348	0.093811	0.937381	0.101218	0.935343	0.090011	0.932204	0.094698	0.936111	0.099704	0.934102
baron	MuSiC	TVAE	endothelia	500	-	-	0.060962	0.936992	0.057561	0.940681	0.061135	0.942861	0.059057	0.939077	0.06185	0.936861	0.059141	0.940814
baron	MuSiC	TVAE	gamma	500	-	-	0.065657	0.920168	0.064569	0.914065	0.062932	0.917865	0.065203	0.913089	0.065651	0.914296	0.064109	0.918694
baron	MuSiC	TVAE	macropha	500	-	-	0.049862	0.92198	0.050084	0.918102	0.046951	0.920215	0.051585	0.917171	0.050814	0.915333	0.048855	0.917971
baron	MuSiC	TVAE	quiescent_	500	-	-	0.096773	0.801787	0.088746	0.838415	0.094177	0.81039	0.091983	0.822123	0.098039	0.797988	0.091009	0.82539
baron	MuSiC	TVAE	acinar	600	-	-	0.122183	0.924653	0.114023	0.926352	0.110764	0.929521	0.116908	0.925728	0.115309	0.924843	0.111816	0.927839
baron	MuSiC	TVAE	activated_	600	-	-	0.077753	0.894844	0.070795	0.896496	0.076329	0.887823	0.069982	0.891841	0.078796	0.890032	0.07362	0.896551
baron	MuSiC	TVAE	alpha	600	-	-	0.050099	0.936714	0.054015	0.932348	0.051809	0.935208	0.05881	0.930231	0.050455	0.938054	0.053408	0.934139
baron	MuSiC	TVAE	beta	600	-	-	0.044632	0.95414	0.044304	0.954904	0.044878	0.954145	0.045168	0.953246	0.044611	0.954598	0.044517	0.954644
baron	MuSiC	TVAE	delta	600	-	-	0.069069	0.883604	0.066744	0.887257	0.063696	0.895816	0.065027	0.891273	0.066453	0.888417	0.066267	0.888229
baron	MuSiC	TVAE	ductal	600	-	-	0.090678	0.934316	0.093557	0.936352	0.101813	0.934892	0.090945	0.932674	0.095324	0.936934	0.100595	0.933849
baron	MuSiC	TVAE	endothelia	600	-	-	0.060859	0.937148	0.057355	0.940572	0.061192	0.942639	0.05899	0.938669	0.061575	0.937589	0.059099	0.941087
baron	MuSiC	TVAE	gamma	600	-	-	0.066418	0.920136	0.064533	0.91342	0.062822	0.918232	0.065128	0.914841	0.066121	0.913886	0.064072	0.919681
baron	MuSiC	TVAE	macropha	600	-	-	0.049694	0.922514	0.050095	0.918156	0.047111	0.919637	0.051572	0.916803	0.05091	0.914912	0.048893	0.918585
baron	MuSiC	TVAE	quiescent_	600	-	-	0.096645	0.802994	0.088678	0.838737	0.093837	0.811804	0.091725	0.824335	0.097896	0.799513	0.090636	0.827558
baron	MuSiC	TVAE	acinar	700	-	-	0.122463	0.924433	0.113125	0.926602	0.110929	0.929552	0.11638	0.925528	0.11571	0.92472	0.112382	0.928268
baron	MuSiC	TVAE	activated_	700	-	-	0.077719	0.894947	0.070341	0.897595	0.076093	0.888109	0.069698	0.890892	0.078702	0.891921	0.073812	0.896106
baron	MuSiC	TVAE	alpha	700	-	-	0.049432	0.937743	0.053962	0.932513	0.051983	0.934918	0.061339	0.928947	0.050229	0.938579	0.053391	0.934228

baron	MuSiC	TVAE	beta	700	-	-	0.044575	0.954398	0.044368	0.954785	0.044805	0.954185	0.045258	0.953009	0.044593	0.954498	0.044543	0.954569
baron	MuSiC	TVAE	delta	700	-	-	0.069395	0.883513	0.066875	0.88708	0.063716	0.895792	0.065055	0.891103	0.066395	0.888664	0.065733	0.889678
baron	MuSiC	TVAE	ductal	700	-	-	0.090513	0.934199	0.093181	0.937225	0.102009	0.934732	0.090418	0.933308	0.094171	0.936926	0.100223	0.933434
baron	MuSiC	TVAE	endothelia	700	-	-	0.061066	0.937011	0.057441	0.939996	0.06094	0.942975	0.059141	0.939056	0.061354	0.937465	0.058891	0.941044
baron	MuSiC	TVAE	gamma	700	-	-	0.066213	0.919788	0.064457	0.913925	0.062852	0.918047	0.064945	0.914572	0.065745	0.913957	0.063902	0.918178
baron	MuSiC	TVAE	macropha	700	-	-	0.049696	0.921846	0.049832	0.918148	0.046846	0.921806	0.051869	0.916313	0.050482	0.91568	0.048811	0.918707
baron	MuSiC	TVAE	quiescent_	700	-	-	0.096231	0.805204	0.088528	0.839722	0.093371	0.813979	0.09183	0.823088	0.09743	0.800912	0.090943	0.826414
baron	MuSiC	TVAE	acinar	800	-	-	0.123141	0.924255	0.11365	0.926947	0.110503	0.92928	0.116794	0.925748	0.115267	0.924923	0.112676	0.928082
baron	MuSiC	TVAE	activated_	800	-	-	0.077559	0.895512	0.071135	0.896333	0.075899	0.887878	0.069746	0.891208	0.079285	0.891253	0.073688	0.896349
baron	MuSiC	TVAE	alpha	800	-	-	0.049959	0.936979	0.053965	0.932316	0.052063	0.935016	0.061393	0.928891	0.050012	0.938648	0.053557	0.933895
baron	MuSiC	TVAE	beta	800	-	-	0.044595	0.954237	0.044334	0.954818	0.044844	0.954169	0.045261	0.953025	0.044537	0.954642	0.044607	0.954428
baron	MuSiC	TVAE	delta	800	-	-	0.068167	0.885575	0.066755	0.886988	0.063942	0.895099	0.065005	0.891656	0.066396	0.888561	0.065823	0.88919
baron	MuSiC	TVAE	ductal	800	-	-	0.090892	0.933916	0.092564	0.936911	0.101893	0.934954	0.090408	0.932535	0.095357	0.93665	0.099635	0.933695
baron	MuSiC	TVAE	endothelia	800	-	-	0.061191	0.936958	0.057476	0.940007	0.060883	0.94299	0.059239	0.938378	0.061615	0.937569	0.05913	0.941747
baron	MuSiC	TVAE	gamma	800	-	-	0.06542	0.919775	0.064441	0.913529	0.063102	0.917232	0.064599	0.913638	0.065589	0.913685	0.063721	0.91858
baron	MuSiC	TVAE	macropha	800	-	-	0.049757	0.921926	0.050275	0.91821	0.046903	0.920547	0.051692	0.916222	0.050599	0.915854	0.048845	0.917296
baron	MuSiC	TVAE	quiescent_	800	-	-	0.096176	0.80505	0.088807	0.83819	0.09362	0.813738	0.092174	0.821748	0.097884	0.799546	0.090966	0.825691
baron	MuSiC	TVAE	acinar	900	-	-	0.122706	0.924256	0.113531	0.926845	0.110947	0.929516	0.116995	0.925721	0.115862	0.924141	0.112538	0.927774
baron	MuSiC	TVAE	activated_	900	-	-	0.077995	0.894747	0.070356	0.897681	0.076007	0.88818	0.069833	0.892078	0.079176	0.890578	0.073507	0.896243
baron	MuSiC	TVAE	alpha	900	-	-	0.050001	0.936972	0.054122	0.932307	0.052276	0.934608	0.060702	0.929212	0.05056	0.938182	0.053579	0.933996
baron	MuSiC	TVAE	beta	900	-	-	0.044607	0.954258	0.044317	0.954814	0.044906	0.954045	0.045305	0.952897	0.044641	0.954488	0.044554	0.954523
baron	MuSiC	TVAE	delta	900	-	-	0.068421	0.884885	0.066744	0.887001	0.064085	0.894449	0.064855	0.892277	0.066468	0.88818	0.065887	0.889404
baron	MuSiC	TVAE	ductal	900	-	-	0.090491	0.934025	0.092157	0.937312	0.101226	0.93507	0.090504	0.932156	0.094905	0.936712	0.099726	0.933889
baron	MuSiC	TVAE	endothelia	900	-	-	0.061027	0.936963	0.057166	0.940643	0.060617	0.94273	0.059227	0.938383	0.061609	0.937063	0.059284	0.941003
baron	MuSiC	TVAE	gamma	900	-	-	0.065828	0.919482	0.064143	0.913597	0.063	0.91804	0.064895	0.912404	0.065787	0.912353	0.064008	0.917939
baron	MuSiC	TVAE	macropha	900	-	-	0.049871	0.922038	0.049767	0.917985	0.046973	0.920525	0.051821	0.916728	0.050815	0.915238	0.048942	0.918639
baron	MuSiC	TVAE	quiescent_	900	-	-	0.096652	0.803192	0.088549	0.839146	0.093605	0.813452	0.092166	0.822042	0.09782	0.798675	0.090627	0.827467
baron	MuSiC	TVAE	acinar	1000	-	-	0.122525	0.924574	0.114252	0.92641	0.111262	0.929382	0.116212	0.925968	0.115823	0.924391	0.113131	0.927701
baron	MuSiC	TVAE	activated_	1000	-	-	0.078202	0.894899	0.070579	0.897354	0.076246	0.887603	0.069809	0.891485	0.078968	0.891745	0.073514	0.896319
baron	MuSiC	TVAE	alpha	1000	-	-	0.049731	0.937321	0.054203	0.932291	0.051892	0.935001	0.061703	0.928845	0.050595	0.937805	0.053207	0.934422
baron	MuSiC	TVAE	beta	1000	-	-	0.044566	0.954315	0.044238	0.955011	0.044875	0.954233	0.045341	0.952896	0.044525	0.95466	0.044638	0.954349
baron	MuSiC	TVAE	delta	1000	-	-	0.068906	0.883773	0.066823	0.887111	0.063549	0.896395	0.064932	0.891975	0.066077	0.889678	0.065907	0.889492
baron	MuSiC	TVAE	ductal	1000	-	-	0.090433	0.934028	0.092782	0.936444	0.101832	0.934724	0.090774	0.932725	0.094874	0.936556	0.099556	0.933789
baron	MuSiC	TVAE	endothelia	1000	-	-	0.061152	0.937146	0.057307	0.940116	0.061013	0.942856	0.058944	0.939	0.061674	0.93721	0.059184	0.941021
baron	MuSiC	TVAE	gamma	1000	-	-	0.066036	0.920128	0.064455	0.913661	0.062794	0.91827	0.065042	0.912802	0.065826	0.913283	0.064158	0.918055
baron	MuSiC	TVAE	macropha	1000	-	-	0.049636	0.922062	0.050307	0.918166	0.046718	0.921433	0.051889	0.916147	0.05089	0.915478	0.048858	0.918041

baron	MuSiC	TVAE	quiescent_	1000	-	-	0.096948	0.802523	0.088399	0.839855	0.093773	0.8122	0.092015	0.823141	0.097458	0.799936	0.090734	0.827944
baron	MuSiC	sc-CMGAI	acinar	100	-	-	0.114215	0.934831	0.119325	0.926907	0.113865	0.93333	0.124113	0.928261	0.121503	0.929438	0.12355	0.927337
baron	MuSiC	sc-CMGAI	activated_	100	-	-	0.080478	0.891136	0.077776	0.895795	0.07795	0.895083	0.079347	0.88908	0.083107	0.889732	0.076072	0.896389
baron	MuSiC	sc-CMGAI	alpha	100	-	-	0.047573	0.941449	0.047884	0.940193	0.049453	0.938254	0.04758	0.941429	0.048463	0.938712	0.062275	0.929928
baron	MuSiC	sc-CMGAI	beta	100	-	-	0.04678	0.950468	0.046355	0.950332	0.052498	0.953524	0.048586	0.952045	0.053419	0.95477	0.053893	0.953451
baron	MuSiC	sc-CMGAI	delta	100	-	-	0.072768	0.880529	0.075585	0.872183	0.087278	0.853716	0.079942	0.866465	0.078422	0.865647	0.078757	0.852849
baron	MuSiC	sc-CMGAI	ductal	100	-	-	0.070979	0.933045	0.0699	0.937025	0.065666	0.941522	0.066069	0.940315	0.06229	0.938414	0.070504	0.934156
baron	MuSiC	sc-CMGAI	endothelia	100	-	-	0.061177	0.936795	0.061229	0.936779	0.059911	0.933009	0.063361	0.938113	0.063291	0.930382	0.061112	0.934112
baron	MuSiC	sc-CMGAI	gamma	100	-	-	0.064878	0.915494	0.06696	0.906281	0.071426	0.91411	0.069056	0.905208	0.065255	0.917247	0.064226	0.911803
baron	MuSiC	sc-CMGAI	macropha	100	-	-	0.053241	0.922589	0.046929	0.924253	0.047929	0.925823	0.046789	0.923746	0.048985	0.924626	0.049638	0.923371
baron	MuSiC	sc-CMGAI	quiescent_	100	-	-	0.105454	0.772576	0.095086	0.806929	0.098669	0.792252	0.103336	0.777153	0.108604	0.772536	0.090823	0.820443
baron	MuSiC	sc-CMGAI	acinar	200	-	-	0.11553	0.932319	0.121144	0.926862	0.119269	0.929833	0.121677	0.930098	0.12673	0.927157	0.122036	0.928974
baron	MuSiC	sc-CMGAI	activated_	200	-	-	0.080081	0.890531	0.077849	0.894517	0.079342	0.891229	0.079689	0.890914	0.083018	0.889873	0.075598	0.897938
baron	MuSiC	sc-CMGAI	alpha	200	-	-	0.04759	0.941225	0.047624	0.941203	0.04889	0.938901	0.047386	0.942244	0.048333	0.939185	0.057734	0.931497
baron	MuSiC	sc-CMGAI	beta	200	-	-	0.048218	0.949711	0.046364	0.950391	0.054221	0.953888	0.045876	0.95138	0.054012	0.954477	0.054289	0.95366
baron	MuSiC	sc-CMGAI	delta	200	-	-	0.072444	0.877594	0.076277	0.868702	0.08847	0.851995	0.077888	0.865997	0.079269	0.863331	0.076326	0.859853
baron	MuSiC	sc-CMGAI	ductal	200	-	-	0.07377	0.933397	0.068415	0.93873	0.0684	0.938574	0.067809	0.938477	0.064463	0.936909	0.070022	0.936827
baron	MuSiC	sc-CMGAI	endothelia	200	-	-	0.060018	0.941128	0.061411	0.932403	0.059177	0.937345	0.063783	0.937851	0.062694	0.933164	0.06041	0.935791
baron	MuSiC	sc-CMGAI	gamma	200	-	-	0.064986	0.917678	0.066735	0.90558	0.071158	0.916091	0.068228	0.912118	0.064269	0.918759	0.062914	0.912938
baron	MuSiC	sc-CMGAI	macropha	200	-	-	0.052268	0.920875	0.04727	0.923578	0.049307	0.924773	0.047326	0.925572	0.048733	0.924193	0.049489	0.924736
baron	MuSiC	sc-CMGAI	quiescent_	200	-	-	0.102455	0.778939	0.095398	0.799108	0.100222	0.79206	0.102163	0.788457	0.106114	0.775949	0.09206	0.814476
baron	MuSiC	sc-CMGAI	acinar	300	-	-	0.116026	0.932717	0.117765	0.928041	0.116725	0.931334	0.122926	0.929675	0.123659	0.928582	0.121539	0.92895
baron	MuSiC	sc-CMGAI	activated_	300	-	-	0.078829	0.892921	0.076955	0.896845	0.078474	0.893992	0.079988	0.890311	0.082122	0.889783	0.07584	0.898297
baron	MuSiC	sc-CMGAI	alpha	300	-	-	0.047441	0.941597	0.04702	0.942629	0.048597	0.939374	0.047519	0.941719	0.048478	0.938763	0.058015	0.931543
baron	MuSiC	sc-CMGAI	beta	300	-	-	0.04738	0.950758	0.046505	0.950069	0.054783	0.953461	0.047339	0.952397	0.055102	0.954705	0.053494	0.953542
baron	MuSiC	sc-CMGAI	delta	300	-	-	0.070636	0.881568	0.075037	0.871739	0.08917	0.851551	0.079075	0.868307	0.081307	0.859942	0.078119	0.856415
baron	MuSiC	sc-CMGAI	ductal	300	-	-	0.07439	0.933061	0.068112	0.939471	0.068283	0.939765	0.068349	0.937965	0.065374	0.937394	0.069461	0.935538
baron	MuSiC	sc-CMGAI	endothelia	300	-	-	0.061161	0.938933	0.061068	0.934811	0.059646	0.933115	0.063484	0.938821	0.06218	0.933367	0.060399	0.933177
baron	MuSiC	sc-CMGAI	gamma	300	-	-	0.064296	0.914863	0.066566	0.907322	0.071346	0.914102	0.066621	0.911058	0.064642	0.920383	0.063805	0.912201
baron	MuSiC	sc-CMGAI	macropha	300	-	-	0.052399	0.922362	0.047086	0.923583	0.048872	0.925753	0.046384	0.926775	0.048333	0.924305	0.04945	0.924889
baron	MuSiC	sc-CMGAI	quiescent_	300	-	-	0.102186	0.783489	0.095723	0.801783	0.09915	0.791203	0.102138	0.784737	0.103972	0.784906	0.090788	0.819814
baron	MuSiC	sc-CMGAI	acinar	400	-	-	0.116389	0.932611	0.11935	0.926386	0.115623	0.932009	0.123622	0.928401	0.123132	0.928734	0.121834	0.929108
baron	MuSiC	sc-CMGAI	activated_	400	-	-	0.079593	0.889762	0.078477	0.895146	0.079648	0.892476	0.080183	0.890985	0.082824	0.890521	0.075047	0.898166
baron	MuSiC	sc-CMGAI	alpha	400	-	-	0.047139	0.942188	0.047224	0.942389	0.048679	0.93952	0.047857	0.940972	0.048516	0.938582	0.057101	0.931887
baron	MuSiC	sc-CMGAI	beta	400	-	-	0.047964	0.949885	0.047276	0.949742	0.053892	0.953816	0.046269	0.951496	0.055228	0.954908	0.054839	0.954024
baron	MuSiC	sc-CMGAI	delta	400	-	-	0.072314	0.876668	0.074812	0.870799	0.087181	0.852567	0.078337	0.867999	0.081106	0.863192	0.079041	0.855533

baron	MuSiC	sc-CMGAI ductal	400	-	-	0.072739	0.933259	0.06763	0.93864	0.070897	0.939916	0.068954	0.937913	0.066038	0.937169	0.068388	0.93677
baron	MuSiC	sc-CMGAI endothelia	400	-	-	0.060924	0.941181	0.06077	0.934804	0.059256	0.935702	0.063762	0.937558	0.062175	0.932879	0.060867	0.936083
baron	MuSiC	sc-CMGAI gamma	400	-	-	0.065045	0.916072	0.066944	0.90434	0.071716	0.91468	0.067626	0.911533	0.064099	0.921192	0.063601	0.910603
baron	MuSiC	sc-CMGAI macropha	400	-	-	0.051873	0.922361	0.047524	0.923369	0.049262	0.925743	0.047575	0.925118	0.048311	0.925547	0.049791	0.924457
baron	MuSiC	sc-CMGAI quiescent_	400	-	-	0.104142	0.776883	0.096934	0.792721	0.101499	0.782956	0.103011	0.779638	0.104723	0.780255	0.09126	0.819136
baron	MuSiC	sc-CMGAI acinar	500	-	-	0.115497	0.933201	0.119086	0.927936	0.116337	0.93209	0.122708	0.929262	0.123834	0.928109	0.121264	0.929026
baron	MuSiC	sc-CMGAI activated_	500	-	-	0.079492	0.890598	0.077781	0.89586	0.079125	0.893852	0.080558	0.890412	0.082608	0.89075	0.075448	0.898332
baron	MuSiC	sc-CMGAI alpha	500	-	-	0.047276	0.94194	0.047424	0.941732	0.049017	0.938919	0.047514	0.941673	0.048692	0.938245	0.059887	0.931393
baron	MuSiC	sc-CMGAI beta	500	-	-	0.046562	0.950568	0.047811	0.949883	0.052834	0.953961	0.048025	0.952152	0.052864	0.95467	0.054184	0.953588
baron	MuSiC	sc-CMGAI delta	500	-	-	0.072229	0.879607	0.073627	0.872491	0.086568	0.853174	0.07893	0.868847	0.079169	0.864496	0.078723	0.85451
baron	MuSiC	sc-CMGAI ductal	500	-	-	0.073086	0.933862	0.067794	0.938448	0.069925	0.938836	0.068484	0.939092	0.065197	0.937891	0.069668	0.936138
baron	MuSiC	sc-CMGAI endothelia	500	-	-	0.06057	0.94087	0.061228	0.936338	0.059326	0.936085	0.064199	0.938931	0.062596	0.9312	0.060345	0.934887
baron	MuSiC	sc-CMGAI gamma	500	-	-	0.064656	0.915594	0.06613	0.906921	0.070933	0.915718	0.066568	0.913142	0.06445	0.920396	0.063714	0.910739
baron	MuSiC	sc-CMGAI macropha	500	-	-	0.051811	0.922755	0.047262	0.923412	0.048968	0.92559	0.047226	0.925651	0.048458	0.92568	0.049285	0.925444
baron	MuSiC	sc-CMGAI quiescent_	500	-	-	0.103189	0.779388	0.097447	0.793638	0.100837	0.78728	0.104043	0.780696	0.104651	0.780473	0.090715	0.820192
baron	MuSiC	sc-CMGAI acinar	600	-	-	0.116256	0.932543	0.119113	0.927109	0.116725	0.932098	0.123674	0.928726	0.124386	0.92811	0.12098	0.929424
baron	MuSiC	sc-CMGAI activated_	600	-	-	0.079524	0.891015	0.077883	0.89692	0.080066	0.891745	0.081014	0.889072	0.082744	0.890829	0.075642	0.897352
baron	MuSiC	sc-CMGAI alpha	600	-	-	0.046984	0.942562	0.047453	0.941657	0.048221	0.939856	0.047235	0.942316	0.048884	0.93773	0.059469	0.931364
baron	MuSiC	sc-CMGAI beta	600	-	-	0.048621	0.949778	0.046182	0.950824	0.055517	0.954026	0.045956	0.951246	0.054605	0.954614	0.054101	0.954565
baron	MuSiC	sc-CMGAI delta	600	-	-	0.071024	0.879766	0.075932	0.870469	0.088757	0.852696	0.077276	0.868728	0.079598	0.863025	0.078318	0.855703
baron	MuSiC	sc-CMGAI ductal	600	-	-	0.073885	0.933143	0.068352	0.938674	0.068994	0.939103	0.069918	0.938365	0.065942	0.937522	0.070844	0.936133
baron	MuSiC	sc-CMGAI endothelia	600	-	-	0.060578	0.941033	0.060923	0.93842	0.05955	0.934937	0.06384	0.940115	0.062743	0.931952	0.060484	0.934338
baron	MuSiC	sc-CMGAI gamma	600	-	-	0.064218	0.917361	0.067068	0.904892	0.07115	0.91334	0.067918	0.912971	0.063697	0.920408	0.063404	0.913243
baron	MuSiC	sc-CMGAI macropha	600	-	-	0.052407	0.922244	0.047642	0.923276	0.049627	0.925576	0.047503	0.924727	0.048762	0.923809	0.049819	0.924805
baron	MuSiC	sc-CMGAI quiescent_	600	-	-	0.102773	0.780676	0.097169	0.796224	0.103271	0.777246	0.103882	0.777532	0.1051	0.777602	0.090782	0.819398
baron	MuSiC	sc-CMGAI acinar	700	-	-	0.116356	0.932819	0.119553	0.927055	0.115751	0.932153	0.122264	0.929553	0.123486	0.928631	0.121455	0.928893
baron	MuSiC	sc-CMGAI activated_	700	-	-	0.079964	0.88854	0.078003	0.896041	0.079472	0.893287	0.079457	0.890699	0.082158	0.8911	0.075839	0.897733
baron	MuSiC	sc-CMGAI alpha	700	-	-	0.047328	0.941664	0.047444	0.941491	0.04845	0.939671	0.047569	0.941447	0.048613	0.938377	0.058603	0.93166
baron	MuSiC	sc-CMGAI beta	700	-	-	0.047554	0.950217	0.046335	0.950437	0.054625	0.953542	0.046651	0.95199	0.054806	0.954811	0.053743	0.953582
baron	MuSiC	sc-CMGAI delta	700	-	-	0.071287	0.87913	0.075639	0.87189	0.088177	0.853521	0.078039	0.870519	0.080746	0.861105	0.077599	0.857692
baron	MuSiC	sc-CMGAI ductal	700	-	-	0.073431	0.93389	0.067831	0.93918	0.069895	0.940055	0.068867	0.939125	0.065317	0.938255	0.068753	0.936877
baron	MuSiC	sc-CMGAI endothelia	700	-	-	0.061027	0.940698	0.060863	0.935771	0.05908	0.936601	0.063686	0.938894	0.062416	0.932251	0.060663	0.933933
baron	MuSiC	sc-CMGAI gamma	700	-	-	0.064814	0.915538	0.066142	0.908873	0.070551	0.915608	0.066966	0.912215	0.063524	0.921234	0.06314	0.913518
baron	MuSiC	sc-CMGAI macropha	700	-	-	0.052284	0.921732	0.047739	0.922724	0.049086	0.925587	0.047203	0.925306	0.048739	0.924412	0.049513	0.924942
baron	MuSiC	sc-CMGAI quiescent_	700	-	-	0.103349	0.779704	0.096351	0.797814	0.101039	0.786133	0.101986	0.78481	0.104574	0.782658	0.09101	0.819963
baron	MuSiC	sc-CMGAI acinar	800	-	-	0.116689	0.932842	0.118691	0.927519	0.11656	0.931386	0.121808	0.929451	0.124631	0.928627	0.122005	0.928589

baron	MuSiC	sc-CMGAI	activated_	800	-	-	0.079672	0.890745	0.077979	0.895872	0.079271	0.892883	0.079838	0.890932	0.083395	0.890514	0.075484	0.898126
baron	MuSiC	sc-CMGAI	alpha	800	-	-	0.046958	0.942622	0.047305	0.941962	0.048395	0.939754	0.047457	0.941959	0.048437	0.938773	0.058768	0.931711
baron	MuSiC	sc-CMGAI	beta	800	-	-	0.04777	0.949748	0.046242	0.950747	0.054609	0.953419	0.046806	0.952174	0.055161	0.95471	0.053995	0.954051
baron	MuSiC	sc-CMGAI	delta	800	-	-	0.071288	0.878898	0.075281	0.872007	0.087969	0.852684	0.079357	0.867921	0.079675	0.863666	0.079942	0.852959
baron	MuSiC	sc-CMGAI	ductal	800	-	-	0.072726	0.933281	0.068733	0.939134	0.069769	0.939549	0.070129	0.938467	0.064929	0.938056	0.069309	0.936981
baron	MuSiC	sc-CMGAI	endothelia	800	-	-	0.061111	0.939157	0.061219	0.9356	0.059202	0.937128	0.063581	0.938789	0.062584	0.931113	0.060668	0.9365
baron	MuSiC	sc-CMGAI	gamma	800	-	-	0.064542	0.915266	0.066815	0.905992	0.070771	0.914824	0.067822	0.910468	0.063752	0.920897	0.064084	0.911813
baron	MuSiC	sc-CMGAI	macropha	800	-	-	0.051981	0.92243	0.047537	0.923255	0.049172	0.925387	0.047441	0.924905	0.048691	0.924119	0.049647	0.924159
baron	MuSiC	sc-CMGAI	quiescent_	800	-	-	0.103765	0.778436	0.096616	0.798533	0.101183	0.787019	0.102345	0.784447	0.10519	0.778021	0.091252	0.818366
baron	MuSiC	sc-CMGAI	acinar	900	-	-	0.115629	0.932961	0.118076	0.928086	0.116209	0.931964	0.122662	0.92882	0.123845	0.928448	0.121538	0.929054
baron	MuSiC	sc-CMGAI	activated_	900	-	-	0.079405	0.890644	0.078345	0.895368	0.079198	0.892847	0.079649	0.891315	0.082462	0.8912	0.075168	0.898401
baron	MuSiC	sc-CMGAI	alpha	900	-	-	0.0471	0.94225	0.047241	0.941981	0.048817	0.939011	0.047395	0.942159	0.048679	0.938276	0.058766	0.931471
baron	MuSiC	sc-CMGAI	beta	900	-	-	0.047604	0.950004	0.047153	0.949731	0.053769	0.95376	0.046403	0.952108	0.053984	0.954732	0.054331	0.95416
baron	MuSiC	sc-CMGAI	delta	900	-	-	0.072322	0.877043	0.075274	0.870338	0.086349	0.855536	0.078346	0.870108	0.079213	0.862735	0.079023	0.855291
baron	MuSiC	sc-CMGAI	ductal	900	-	-	0.072892	0.934784	0.068969	0.938501	0.069689	0.939208	0.068747	0.939237	0.065604	0.937782	0.069912	0.935645
baron	MuSiC	sc-CMGAI	endothelia	900	-	-	0.060856	0.940007	0.06062	0.937808	0.058821	0.937216	0.063427	0.939622	0.062537	0.931002	0.060155	0.933955
baron	MuSiC	sc-CMGAI	gamma	900	-	-	0.065016	0.915606	0.066876	0.906106	0.070629	0.914691	0.066857	0.914186	0.063802	0.921194	0.063601	0.913488
baron	MuSiC	sc-CMGAI	macropha	900	-	-	0.05155	0.92296	0.04752	0.923827	0.049272	0.925255	0.04713	0.924818	0.048613	0.924506	0.049919	0.924429
baron	MuSiC	sc-CMGAI	quiescent_	900	-	-	0.10238	0.782552	0.097028	0.793927	0.10132	0.786323	0.102221	0.784324	0.104094	0.78206	0.090516	0.821394
baron	MuSiC	sc-CMGAI	acinar	1000	-	-	0.117148	0.932494	0.119104	0.927175	0.115962	0.931729	0.122053	0.92955	0.124094	0.927926	0.120126	0.929608
baron	MuSiC	sc-CMGAI	activated_	1000	-	-	0.079549	0.890092	0.077289	0.895934	0.079342	0.892381	0.079673	0.890917	0.082256	0.892759	0.076485	0.897894
baron	MuSiC	sc-CMGAI	alpha	1000	-	-	0.047332	0.941755	0.047205	0.942175	0.048495	0.939502	0.047445	0.941877	0.048417	0.938861	0.057945	0.93224
baron	MuSiC	sc-CMGAI	beta	1000	-	-	0.0473	0.950123	0.046322	0.950385	0.053856	0.953882	0.04593	0.95175	0.054432	0.954621	0.053914	0.954116
baron	MuSiC	sc-CMGAI	delta	1000	-	-	0.072064	0.878152	0.075601	0.872525	0.08825	0.853744	0.077838	0.868762	0.078176	0.865659	0.078802	0.856161
baron	MuSiC	sc-CMGAI	ductal	1000	-	-	0.072303	0.93396	0.069548	0.938637	0.070109	0.938662	0.06931	0.938817	0.06631	0.937179	0.068542	0.937875
baron	MuSiC	sc-CMGAI	endothelia	1000	-	-	0.060617	0.939909	0.060623	0.938343	0.059019	0.938366	0.063694	0.938922	0.062513	0.932495	0.060472	0.933573
baron	MuSiC	sc-CMGAI	gamma	1000	-	-	0.064689	0.916775	0.065989	0.908207	0.071193	0.915694	0.067673	0.912962	0.062878	0.921142	0.063383	0.913222
baron	MuSiC	sc-CMGAI	macropha	1000	-	-	0.051804	0.922515	0.047384	0.922853	0.04898	0.925704	0.047014	0.92603	0.04867	0.924243	0.049576	0.924906
baron	MuSiC	sc-CMGAI	quiescent_	1000	-	-	0.103106	0.779365	0.09568	0.801819	0.10171	0.786498	0.102319	0.786107	0.104375	0.782558	0.091905	0.81525
baron	BisqueRN	Control	acinar	0	0.157524	0.895428	-	-	-	-	-	-	-	-	-	-	-	-
baron	BisqueRN	Control	activated_	0	0.134314	0.637096	-	-	-	-	-	-	-	-	-	-	-	-
baron	BisqueRN	Control	alpha	0	0.181582	0.916573	-	-	-	-	-	-	-	-	-	-	-	-
baron	BisqueRN	Control	beta	0	0.24979	0.93569	-	-	-	-	-	-	-	-	-	-	-	-
baron	BisqueRN	Control	delta	0	0.135988	0.324097	-	-	-	-	-	-	-	-	-	-	-	-
baron	BisqueRN	Control	ductal	0	0.121975	0.708778	-	-	-	-	-	-	-	-	-	-	-	-
baron	BisqueRN	Control	endothelia	0	0.137512	0.571667	-	-	-	-	-	-	-	-	-	-	-	-

baron	BisqueRN Control	gamma	0	0.128013	0.730827	-	-	-	-	-	-	-	-	-	-	-	-
baron	BisqueRN Control	macropha	0	0.126349	0.844039	-	-	-	-	-	-	-	-	-	-	-	-
baron	BisqueRN Control	quiescent_	0	0.150449	0.474361	-	-	-	-	-	-	-	-	-	-	-	-
baron	BisqueRN Copula	acinar	100	0.137495	0.898905	-	-	-	-	-	-	-	-	-	-	-	-
baron	BisqueRN Copula	activated_	100	0.110919	0.811132	-	-	-	-	-	-	-	-	-	-	-	-
baron	BisqueRN Copula	alpha	100	0.13169	0.918844	-	-	-	-	-	-	-	-	-	-	-	-
baron	BisqueRN Copula	beta	100	0.170854	0.937199	-	-	-	-	-	-	-	-	-	-	-	-
baron	BisqueRN Copula	delta	100	0.113206	0.634697	-	-	-	-	-	-	-	-	-	-	-	-
baron	BisqueRN Copula	ductal	100	0.114041	0.705803	-	-	-	-	-	-	-	-	-	-	-	-
baron	BisqueRN Copula	endothelia	100	0.120031	0.733682	-	-	-	-	-	-	-	-	-	-	-	-
baron	BisqueRN Copula	gamma	100	0.125406	0.618254	-	-	-	-	-	-	-	-	-	-	-	-
baron	BisqueRN Copula	macropha	100	0.083519	0.89558	-	-	-	-	-	-	-	-	-	-	-	-
baron	BisqueRN Copula	quiescent_	100	0.130467	0.649043	-	-	-	-	-	-	-	-	-	-	-	-
baron	BisqueRN Copula	acinar	200	0.137405	0.899427	-	-	-	-	-	-	-	-	-	-	-	-
baron	BisqueRN Copula	activated_	200	0.111217	0.81415	-	-	-	-	-	-	-	-	-	-	-	-
baron	BisqueRN Copula	alpha	200	0.131667	0.918875	-	-	-	-	-	-	-	-	-	-	-	-
baron	BisqueRN Copula	beta	200	0.170908	0.936963	-	-	-	-	-	-	-	-	-	-	-	-
baron	BisqueRN Copula	delta	200	0.114129	0.628222	-	-	-	-	-	-	-	-	-	-	-	-
baron	BisqueRN Copula	ductal	200	0.114425	0.702562	-	-	-	-	-	-	-	-	-	-	-	-
baron	BisqueRN Copula	endothelia	200	0.119675	0.755465	-	-	-	-	-	-	-	-	-	-	-	-
baron	BisqueRN Copula	gamma	200	0.12561	0.625521	-	-	-	-	-	-	-	-	-	-	-	-
baron	BisqueRN Copula	macropha	200	0.083681	0.896509	-	-	-	-	-	-	-	-	-	-	-	-
baron	BisqueRN Copula	quiescent_	200	0.130858	0.650058	-	-	-	-	-	-	-	-	-	-	-	-
baron	BisqueRN Copula	acinar	300	0.137308	0.89883	-	-	-	-	-	-	-	-	-	-	-	-
baron	BisqueRN Copula	activated_	300	0.110631	0.813494	-	-	-	-	-	-	-	-	-	-	-	-
baron	BisqueRN Copula	alpha	300	0.131703	0.918911	-	-	-	-	-	-	-	-	-	-	-	-
baron	BisqueRN Copula	beta	300	0.170853	0.937306	-	-	-	-	-	-	-	-	-	-	-	-
baron	BisqueRN Copula	delta	300	0.114508	0.626244	-	-	-	-	-	-	-	-	-	-	-	-
baron	BisqueRN Copula	ductal	300	0.115679	0.680857	-	-	-	-	-	-	-	-	-	-	-	-
baron	BisqueRN Copula	endothelia	300	0.11975	0.747466	-	-	-	-	-	-	-	-	-	-	-	-
baron	BisqueRN Copula	gamma	300	0.124773	0.64968	-	-	-	-	-	-	-	-	-	-	-	-
baron	BisqueRN Copula	macropha	300	0.083595	0.893898	-	-	-	-	-	-	-	-	-	-	-	-
baron	BisqueRN Copula	quiescent_	300	0.130619	0.6757	-	-	-	-	-	-	-	-	-	-	-	-
baron	BisqueRN Copula	acinar	400	0.137567	0.898061	-	-	-	-	-	-	-	-	-	-	-	-
baron	BisqueRN Copula	activated_	400	0.110912	0.810287	-	-	-	-	-	-	-	-	-	-	-	-
baron	BisqueRN Copula	alpha	400	0.131672	0.918943	-	-	-	-	-	-	-	-	-	-	-	-

baron	BisqueRN Copula	beta	400	0.170844	0.937263	-	-	-	-	-	-	-	-	-	-	-	-
baron	BisqueRN Copula	delta	400	0.114088	0.626341	-	-	-	-	-	-	-	-	-	-	-	-
baron	BisqueRN Copula	ductal	400	0.114652	0.692167	-	-	-	-	-	-	-	-	-	-	-	-
baron	BisqueRN Copula	endothelia	400	0.120286	0.720362	-	-	-	-	-	-	-	-	-	-	-	-
baron	BisqueRN Copula	gamma	400	0.125161	0.644348	-	-	-	-	-	-	-	-	-	-	-	-
baron	BisqueRN Copula	macropha	400	0.082817	0.897385	-	-	-	-	-	-	-	-	-	-	-	-
baron	BisqueRN Copula	quiescent_	400	0.131147	0.657366	-	-	-	-	-	-	-	-	-	-	-	-
baron	BisqueRN Copula	acinar	500	0.137439	0.899063	-	-	-	-	-	-	-	-	-	-	-	-
baron	BisqueRN Copula	activated_	500	0.110928	0.810682	-	-	-	-	-	-	-	-	-	-	-	-
baron	BisqueRN Copula	alpha	500	0.131679	0.918895	-	-	-	-	-	-	-	-	-	-	-	-
baron	BisqueRN Copula	beta	500	0.170857	0.937233	-	-	-	-	-	-	-	-	-	-	-	-
baron	BisqueRN Copula	delta	500	0.113638	0.637665	-	-	-	-	-	-	-	-	-	-	-	-
baron	BisqueRN Copula	ductal	500	0.115065	0.691442	-	-	-	-	-	-	-	-	-	-	-	-
baron	BisqueRN Copula	endothelia	500	0.119989	0.741291	-	-	-	-	-	-	-	-	-	-	-	-
baron	BisqueRN Copula	gamma	500	0.125368	0.631848	-	-	-	-	-	-	-	-	-	-	-	-
baron	BisqueRN Copula	macropha	500	0.083241	0.897503	-	-	-	-	-	-	-	-	-	-	-	-
baron	BisqueRN Copula	quiescent_	500	0.131818	0.633198	-	-	-	-	-	-	-	-	-	-	-	-
baron	BisqueRN Copula	acinar	600	0.137248	0.899191	-	-	-	-	-	-	-	-	-	-	-	-
baron	BisqueRN Copula	activated_	600	0.110928	0.811134	-	-	-	-	-	-	-	-	-	-	-	-
baron	BisqueRN Copula	alpha	600	0.1317	0.918913	-	-	-	-	-	-	-	-	-	-	-	-
baron	BisqueRN Copula	beta	600	0.170868	0.937154	-	-	-	-	-	-	-	-	-	-	-	-
baron	BisqueRN Copula	delta	600	0.114097	0.633687	-	-	-	-	-	-	-	-	-	-	-	-
baron	BisqueRN Copula	ductal	600	0.115446	0.679482	-	-	-	-	-	-	-	-	-	-	-	-
baron	BisqueRN Copula	endothelia	600	0.119707	0.760074	-	-	-	-	-	-	-	-	-	-	-	-
baron	BisqueRN Copula	gamma	600	0.125741	0.624865	-	-	-	-	-	-	-	-	-	-	-	-
baron	BisqueRN Copula	macropha	600	0.082672	0.896349	-	-	-	-	-	-	-	-	-	-	-	-
baron	BisqueRN Copula	quiescent_	600	0.130904	0.66202	-	-	-	-	-	-	-	-	-	-	-	-
baron	BisqueRN Copula	acinar	700	0.137377	0.899045	-	-	-	-	-	-	-	-	-	-	-	-
baron	BisqueRN Copula	activated_	700	0.110811	0.812186	-	-	-	-	-	-	-	-	-	-	-	-
baron	BisqueRN Copula	alpha	700	0.131672	0.91894	-	-	-	-	-	-	-	-	-	-	-	-
baron	BisqueRN Copula	beta	700	0.170915	0.93709	-	-	-	-	-	-	-	-	-	-	-	-
baron	BisqueRN Copula	delta	700	0.114025	0.634116	-	-	-	-	-	-	-	-	-	-	-	-
baron	BisqueRN Copula	ductal	700	0.115301	0.68355	-	-	-	-	-	-	-	-	-	-	-	-
baron	BisqueRN Copula	endothelia	700	0.119483	0.7559	-	-	-	-	-	-	-	-	-	-	-	-
baron	BisqueRN Copula	gamma	700	0.124882	0.658239	-	-	-	-	-	-	-	-	-	-	-	-
baron	BisqueRN Copula	macropha	700	0.082653	0.89804	-	-	-	-	-	-	-	-	-	-	-	-

baron	BisqueRN Copula	quiescent_	700	0.131065	0.660127	-	-	-	-	-	-	-	-	-	-	-	-
baron	BisqueRN Copula	acinar	800	0.137355	0.899215	-	-	-	-	-	-	-	-	-	-	-	-
baron	BisqueRN Copula	activated_	800	0.111139	0.812408	-	-	-	-	-	-	-	-	-	-	-	-
baron	BisqueRN Copula	alpha	800	0.131676	0.919048	-	-	-	-	-	-	-	-	-	-	-	-
baron	BisqueRN Copula	beta	800	0.170858	0.937247	-	-	-	-	-	-	-	-	-	-	-	-
baron	BisqueRN Copula	delta	800	0.113712	0.638506	-	-	-	-	-	-	-	-	-	-	-	-
baron	BisqueRN Copula	ductal	800	0.11587	0.673857	-	-	-	-	-	-	-	-	-	-	-	-
baron	BisqueRN Copula	endothelia	800	0.120195	0.734031	-	-	-	-	-	-	-	-	-	-	-	-
baron	BisqueRN Copula	gamma	800	0.124971	0.639847	-	-	-	-	-	-	-	-	-	-	-	-
baron	BisqueRN Copula	macropha	800	0.082584	0.897438	-	-	-	-	-	-	-	-	-	-	-	-
baron	BisqueRN Copula	quiescent_	800	0.131317	0.651482	-	-	-	-	-	-	-	-	-	-	-	-
baron	BisqueRN Copula	acinar	900	0.137374	0.899	-	-	-	-	-	-	-	-	-	-	-	-
baron	BisqueRN Copula	activated_	900	0.110797	0.812064	-	-	-	-	-	-	-	-	-	-	-	-
baron	BisqueRN Copula	alpha	900	0.131695	0.918977	-	-	-	-	-	-	-	-	-	-	-	-
baron	BisqueRN Copula	beta	900	0.170882	0.937152	-	-	-	-	-	-	-	-	-	-	-	-
baron	BisqueRN Copula	delta	900	0.113895	0.638244	-	-	-	-	-	-	-	-	-	-	-	-
baron	BisqueRN Copula	ductal	900	0.115084	0.684824	-	-	-	-	-	-	-	-	-	-	-	-
baron	BisqueRN Copula	endothelia	900	0.119143	0.758159	-	-	-	-	-	-	-	-	-	-	-	-
baron	BisqueRN Copula	gamma	900	0.126287	0.602977	-	-	-	-	-	-	-	-	-	-	-	-
baron	BisqueRN Copula	macropha	900	0.083006	0.896196	-	-	-	-	-	-	-	-	-	-	-	-
baron	BisqueRN Copula	quiescent_	900	0.131192	0.658321	-	-	-	-	-	-	-	-	-	-	-	-
baron	BisqueRN Copula	acinar	1000	0.137291	0.898972	-	-	-	-	-	-	-	-	-	-	-	-
baron	BisqueRN Copula	activated_	1000	0.110765	0.812058	-	-	-	-	-	-	-	-	-	-	-	-
baron	BisqueRN Copula	alpha	1000	0.131677	0.918949	-	-	-	-	-	-	-	-	-	-	-	-
baron	BisqueRN Copula	beta	1000	0.170875	0.937315	-	-	-	-	-	-	-	-	-	-	-	-
baron	BisqueRN Copula	delta	1000	0.112667	0.646115	-	-	-	-	-	-	-	-	-	-	-	-
baron	BisqueRN Copula	ductal	1000	0.115723	0.681169	-	-	-	-	-	-	-	-	-	-	-	-
baron	BisqueRN Copula	endothelia	1000	0.11954	0.765048	-	-	-	-	-	-	-	-	-	-	-	-
baron	BisqueRN Copula	gamma	1000	0.125933	0.613289	-	-	-	-	-	-	-	-	-	-	-	-
baron	BisqueRN Copula	macropha	1000	0.082553	0.895826	-	-	-	-	-	-	-	-	-	-	-	-
baron	BisqueRN Copula	quiescent_	1000	0.130748	0.662015	-	-	-	-	-	-	-	-	-	-	-	-
baron	BisqueRN CTGAN	acinar	100	-	-	0.138652	0.891365	0.137325	0.898673	0.137678	0.899793	0.137689	0.895553	0.137519	0.898858	0.13814	0.89666
baron	BisqueRN CTGAN	activated_	100	-	-	0.110838	0.812611	0.109726	0.807296	0.110963	0.804268	0.111523	0.813324	0.111045	0.799646	0.11156	0.812749
baron	BisqueRN CTGAN	alpha	100	-	-	0.131697	0.919043	0.131728	0.919118	0.131774	0.918923	0.131691	0.919111	0.131703	0.919001	0.131728	0.919016
baron	BisqueRN CTGAN	beta	100	-	-	0.170629	0.937522	0.170816	0.937064	0.170724	0.937642	0.170895	0.937053	0.170761	0.937818	0.170964	0.936858
baron	BisqueRN CTGAN	delta	100	-	-	0.114725	0.61542	0.117537	0.576882	0.120543	0.546579	0.11553	0.600622	0.116713	0.607013	0.114333	0.632109

baron	BisqueRN CTGAN	ductal	100	-	-	0.114923	0.666906	0.114964	0.678788	0.115568	0.712959	0.111011	0.750878	0.113843	0.714569	0.112126	0.75195
baron	BisqueRN CTGAN	endothelia	100	-	-	0.124913	0.592263	0.12007	0.757405	0.123437	0.676855	0.118709	0.75749	0.122181	0.656412	0.118583	0.764204
baron	BisqueRN CTGAN	gamma	100	-	-	0.125295	0.629447	0.127278	0.597321	0.12079	0.777165	0.129289	0.525707	0.126985	0.556174	0.123016	0.703869
baron	BisqueRN CTGAN	macropha	100	-	-	0.083614	0.899655	0.084812	0.901688	0.083476	0.891176	0.084009	0.896017	0.086572	0.888974	0.084712	0.88997
baron	BisqueRN CTGAN	quiescent_	100	-	-	0.133645	0.566766	0.135236	0.525182	0.134406	0.594959	0.13213	0.641147	0.134195	0.546824	0.130613	0.692922
baron	BisqueRN CTGAN	acinar	200	-	-	0.139052	0.888424	0.137007	0.898514	0.137824	0.899717	0.138052	0.893335	0.13765	0.898697	0.138302	0.895699
baron	BisqueRN CTGAN	activated_	200	-	-	0.110935	0.811993	0.109651	0.80262	0.111228	0.783911	0.111345	0.810964	0.110805	0.799885	0.111623	0.81153
baron	BisqueRN CTGAN	alpha	200	-	-	0.131711	0.918939	0.131738	0.91902	0.13181	0.918901	0.131646	0.918845	0.131659	0.918763	0.131785	0.918844
baron	BisqueRN CTGAN	beta	200	-	-	0.170799	0.937379	0.170888	0.937196	0.170732	0.937666	0.170898	0.937067	0.170963	0.937169	0.171031	0.936906
baron	BisqueRN CTGAN	delta	200	-	-	0.116706	0.607273	0.121556	0.531573	0.123854	0.506903	0.121428	0.533916	0.118532	0.590893	0.119572	0.563681
baron	BisqueRN CTGAN	ductal	200	-	-	0.115502	0.659782	0.114757	0.677089	0.115715	0.711264	0.110863	0.729864	0.115585	0.69539	0.112601	0.742725
baron	BisqueRN CTGAN	endothelia	200	-	-	0.123493	0.653468	0.121232	0.735002	0.124332	0.659385	0.120624	0.716971	0.122149	0.6793	0.120332	0.744653
baron	BisqueRN CTGAN	gamma	200	-	-	0.128017	0.553703	0.128679	0.600481	0.123743	0.729374	0.131001	0.512976	0.127326	0.55536	0.122387	0.757278
baron	BisqueRN CTGAN	macropha	200	-	-	0.084344	0.898854	0.085105	0.907287	0.083548	0.890352	0.084439	0.89067	0.086866	0.889004	0.084098	0.88608
baron	BisqueRN CTGAN	quiescent_	200	-	-	0.134756	0.550819	0.136701	0.490151	0.138379	0.490084	0.13345	0.630783	0.136482	0.500083	0.131416	0.695296
baron	BisqueRN CTGAN	acinar	300	-	-	0.139131	0.888408	0.136962	0.899091	0.138032	0.898774	0.137854	0.892636	0.137509	0.899081	0.138255	0.894954
baron	BisqueRN CTGAN	activated_	300	-	-	0.110921	0.813875	0.109099	0.801007	0.111147	0.774923	0.111466	0.810969	0.110801	0.799218	0.111488	0.812526
baron	BisqueRN CTGAN	alpha	300	-	-	0.131708	0.919152	0.131759	0.919088	0.13188	0.918859	0.13166	0.918945	0.131693	0.919021	0.131747	0.918948
baron	BisqueRN CTGAN	beta	300	-	-	0.170724	0.937433	0.170903	0.937155	0.170595	0.938049	0.171075	0.936604	0.170921	0.937527	0.171084	0.936873
baron	BisqueRN CTGAN	delta	300	-	-	0.118785	0.57876	0.122724	0.517798	0.125191	0.488874	0.122039	0.52744	0.120047	0.575752	0.121032	0.546375
baron	BisqueRN CTGAN	ductal	300	-	-	0.116079	0.653193	0.115074	0.671177	0.117039	0.671939	0.11022	0.736923	0.116423	0.684272	0.112691	0.739933
baron	BisqueRN CTGAN	endothelia	300	-	-	0.123465	0.649552	0.120745	0.753557	0.126561	0.597585	0.121464	0.763714	0.123405	0.663271	0.119892	0.74749
baron	BisqueRN CTGAN	gamma	300	-	-	0.128289	0.56605	0.129722	0.566256	0.125086	0.711369	0.131632	0.462127	0.127264	0.563104	0.12329	0.745419
baron	BisqueRN CTGAN	macropha	300	-	-	0.084922	0.900324	0.085232	0.906563	0.082894	0.890984	0.084756	0.887589	0.087178	0.887783	0.084264	0.885634
baron	BisqueRN CTGAN	quiescent_	300	-	-	0.136509	0.508171	0.138864	0.442308	0.139807	0.458769	0.131841	0.680483	0.137541	0.473177	0.131912	0.693482
baron	BisqueRN CTGAN	acinar	400	-	-	0.139331	0.88671	0.13701	0.898144	0.138124	0.89873	0.137972	0.892302	0.137461	0.899386	0.138428	0.894759
baron	BisqueRN CTGAN	activated_	400	-	-	0.111022	0.814581	0.108751	0.802597	0.110766	0.778118	0.111675	0.80913	0.110536	0.798628	0.111648	0.810301
baron	BisqueRN CTGAN	alpha	400	-	-	0.131695	0.918961	0.131756	0.919148	0.13182	0.919266	0.131708	0.919027	0.131691	0.91901	0.131768	0.919127
baron	BisqueRN CTGAN	beta	400	-	-	0.170727	0.937696	0.170835	0.937381	0.170604	0.938152	0.171037	0.936669	0.170873	0.937859	0.171088	0.936789
baron	BisqueRN CTGAN	delta	400	-	-	0.119824	0.565101	0.12171	0.529612	0.127034	0.465948	0.122961	0.51639	0.120597	0.572987	0.122075	0.541831
baron	BisqueRN CTGAN	ductal	400	-	-	0.116512	0.643103	0.115698	0.65951	0.117644	0.675508	0.112441	0.705291	0.117387	0.668292	0.113297	0.739495
baron	BisqueRN CTGAN	endothelia	400	-	-	0.124051	0.634195	0.121819	0.73624	0.126322	0.603633	0.121766	0.743269	0.124544	0.636493	0.121873	0.694899
baron	BisqueRN CTGAN	gamma	400	-	-	0.128162	0.562403	0.12993	0.553815	0.125892	0.673938	0.132656	0.45327	0.127751	0.541308	0.122299	0.75272
baron	BisqueRN CTGAN	macropha	400	-	-	0.084933	0.899198	0.085206	0.904885	0.083397	0.890575	0.084698	0.886404	0.087286	0.885008	0.083807	0.890128
baron	BisqueRN CTGAN	quiescent_	400	-	-	0.136381	0.516823	0.138298	0.458315	0.13947	0.474944	0.133091	0.652797	0.137688	0.464582	0.132364	0.681393
baron	BisqueRN CTGAN	acinar	500	-	-	0.139509	0.886356	0.136895	0.898592	0.138078	0.898848	0.138102	0.890612	0.137449	0.899438	0.138504	0.894302

baron	BisqueRN CTGAN	activated_	500	-	-	0.111222	0.813393	0.108771	0.798726	0.111131	0.762563	0.11159	0.810184	0.110623	0.799114	0.111893	0.813449
baron	BisqueRN CTGAN	alpha	500	-	-	0.131725	0.919172	0.131764	0.919195	0.131824	0.919368	0.131658	0.918896	0.131613	0.91873	0.131789	0.919206
baron	BisqueRN CTGAN	beta	500	-	-	0.170597	0.937785	0.170885	0.937212	0.170545	0.938108	0.171067	0.93673	0.170882	0.937586	0.171096	0.936704
baron	BisqueRN CTGAN	delta	500	-	-	0.119504	0.578582	0.121861	0.527773	0.127234	0.461314	0.124326	0.499456	0.121317	0.568681	0.122766	0.52514
baron	BisqueRN CTGAN	ductal	500	-	-	0.117732	0.630613	0.115962	0.656631	0.11807	0.672107	0.111718	0.714337	0.117064	0.676169	0.113398	0.729748
baron	BisqueRN CTGAN	endothelia	500	-	-	0.124577	0.623915	0.121864	0.729064	0.127051	0.586539	0.122306	0.735223	0.124666	0.635135	0.121056	0.734083
baron	BisqueRN CTGAN	gamma	500	-	-	0.128225	0.560851	0.130573	0.537023	0.126888	0.639795	0.132007	0.472665	0.127813	0.524793	0.124091	0.724187
baron	BisqueRN CTGAN	macropha	500	-	-	0.084675	0.899307	0.084963	0.906435	0.083091	0.889575	0.084024	0.887192	0.087395	0.88825	0.083871	0.890036
baron	BisqueRN CTGAN	quiescent_	500	-	-	0.137725	0.486272	0.139114	0.434712	0.140336	0.445741	0.132858	0.662002	0.138687	0.448256	0.132001	0.701145
baron	BisqueRN CTGAN	acinar	600	-	-	0.139706	0.88223	0.137049	0.898679	0.137986	0.898732	0.137822	0.89223	0.137497	0.899075	0.138409	0.89445
baron	BisqueRN CTGAN	activated_	600	-	-	0.111259	0.813483	0.108889	0.798456	0.110705	0.763072	0.111654	0.810457	0.110678	0.798878	0.111679	0.81439
baron	BisqueRN CTGAN	alpha	600	-	-	0.131693	0.918918	0.131779	0.9193	0.131833	0.919226	0.131676	0.918947	0.131651	0.918787	0.131789	0.919189
baron	BisqueRN CTGAN	beta	600	-	-	0.170546	0.937813	0.17086	0.937143	0.170401	0.938138	0.171103	0.936685	0.170979	0.937492	0.171124	0.936535
baron	BisqueRN CTGAN	delta	600	-	-	0.119961	0.57408	0.122219	0.523925	0.129213	0.438698	0.124769	0.493809	0.121461	0.571331	0.122762	0.532242
baron	BisqueRN CTGAN	ductal	600	-	-	0.118758	0.608469	0.115524	0.66665	0.117151	0.684851	0.111271	0.724472	0.117591	0.677882	0.113969	0.721937
baron	BisqueRN CTGAN	endothelia	600	-	-	0.12619	0.587387	0.121806	0.7533	0.127849	0.54763	0.122631	0.724351	0.124239	0.656283	0.12134	0.722996
baron	BisqueRN CTGAN	gamma	600	-	-	0.127416	0.567621	0.130109	0.549256	0.127827	0.620918	0.13353	0.423988	0.127758	0.52819	0.123371	0.755313
baron	BisqueRN CTGAN	macropha	600	-	-	0.084073	0.89932	0.085376	0.905164	0.083356	0.889192	0.084138	0.885911	0.087763	0.886054	0.083254	0.891487
baron	BisqueRN CTGAN	quiescent_	600	-	-	0.137053	0.506348	0.139383	0.429409	0.141637	0.410644	0.133533	0.650356	0.139171	0.427166	0.132295	0.697075
baron	BisqueRN CTGAN	acinar	700	-	-	0.139608	0.884965	0.136969	0.898363	0.138	0.898601	0.138007	0.890849	0.137438	0.899248	0.138393	0.894884
baron	BisqueRN CTGAN	activated_	700	-	-	0.11127	0.813163	0.108664	0.794346	0.111066	0.768997	0.111627	0.80882	0.110615	0.797363	0.111696	0.812715
baron	BisqueRN CTGAN	alpha	700	-	-	0.13169	0.918962	0.13177	0.919159	0.131912	0.919021	0.131595	0.918708	0.131667	0.918783	0.131829	0.919124
baron	BisqueRN CTGAN	beta	700	-	-	0.17067	0.937542	0.170899	0.937151	0.17043	0.938132	0.171093	0.936594	0.170927	0.937728	0.171107	0.936691
baron	BisqueRN CTGAN	delta	700	-	-	0.120829	0.573296	0.122138	0.524773	0.128973	0.437838	0.124742	0.494422	0.121654	0.567476	0.12333	0.52181
baron	BisqueRN CTGAN	ductal	700	-	-	0.117898	0.626083	0.116884	0.64617	0.117866	0.667262	0.111635	0.714063	0.117921	0.666583	0.114294	0.716542
baron	BisqueRN CTGAN	endothelia	700	-	-	0.12441	0.631165	0.122419	0.733235	0.127943	0.552875	0.123426	0.72081	0.125028	0.638107	0.121877	0.70506
baron	BisqueRN CTGAN	gamma	700	-	-	0.126931	0.58987	0.130784	0.525255	0.127215	0.619409	0.13217	0.465512	0.128091	0.517899	0.123857	0.733291
baron	BisqueRN CTGAN	macropha	700	-	-	0.084531	0.899862	0.085164	0.90571	0.082942	0.88985	0.083882	0.887638	0.087073	0.888117	0.083448	0.890917
baron	BisqueRN CTGAN	quiescent_	700	-	-	0.137787	0.484332	0.139347	0.43232	0.140554	0.445812	0.132262	0.678266	0.138494	0.451487	0.133059	0.676124
baron	BisqueRN CTGAN	acinar	800	-	-	0.139748	0.883288	0.13687	0.898398	0.138112	0.898667	0.137996	0.891577	0.137353	0.899262	0.138412	0.894467
baron	BisqueRN CTGAN	activated_	800	-	-	0.11103	0.816568	0.109078	0.796423	0.110941	0.761245	0.111679	0.809488	0.110613	0.798158	0.111702	0.812345
baron	BisqueRN CTGAN	alpha	800	-	-	0.131713	0.919113	0.131757	0.919083	0.131889	0.919039	0.13164	0.918821	0.131614	0.918579	0.131729	0.919124
baron	BisqueRN CTGAN	beta	800	-	-	0.17072	0.937435	0.170922	0.937254	0.170519	0.938103	0.171139	0.936557	0.170924	0.937523	0.171148	0.936851
baron	BisqueRN CTGAN	delta	800	-	-	0.120228	0.582156	0.122265	0.523002	0.12913	0.436689	0.125318	0.486254	0.121908	0.56545	0.124183	0.507236
baron	BisqueRN CTGAN	ductal	800	-	-	0.117749	0.637192	0.11663	0.641566	0.11907	0.659628	0.111443	0.718627	0.117951	0.670727	0.114579	0.706256
baron	BisqueRN CTGAN	endothelia	800	-	-	0.123961	0.645056	0.122356	0.724448	0.127519	0.566729	0.123561	0.720253	0.12508	0.631672	0.122898	0.67657

baron	BisqueRN CTGAN	gamma	800	-	-	0.127498	0.548169	0.130731	0.535192	0.127834	0.6078	0.133239	0.429369	0.128295	0.496944	0.124686	0.70879
baron	BisqueRN CTGAN	macropha	800	-	-	0.084127	0.90042	0.085238	0.906141	0.083167	0.888617	0.084135	0.884884	0.087615	0.886264	0.083018	0.89141
baron	BisqueRN CTGAN	quiescent_	800	-	-	0.137464	0.501994	0.139649	0.42265	0.141507	0.414771	0.132691	0.676112	0.138997	0.440691	0.133478	0.659705
baron	BisqueRN CTGAN	acinar	900	-	-	0.139606	0.885267	0.136878	0.899108	0.138259	0.897889	0.137854	0.892208	0.137415	0.899362	0.138613	0.893098
baron	BisqueRN CTGAN	activated_	900	-	-	0.111302	0.813782	0.108947	0.794181	0.111008	0.752442	0.111623	0.808927	0.110449	0.798774	0.111643	0.812751
baron	BisqueRN CTGAN	alpha	900	-	-	0.131702	0.919008	0.131779	0.919191	0.131831	0.919437	0.131646	0.918749	0.13164	0.918659	0.131809	0.919236
baron	BisqueRN CTGAN	beta	900	-	-	0.170593	0.937639	0.170881	0.93714	0.170365	0.937821	0.171161	0.936586	0.170919	0.9375	0.171126	0.936627
baron	BisqueRN CTGAN	delta	900	-	-	0.121142	0.571181	0.121968	0.52693	0.12887	0.437395	0.125817	0.479983	0.122173	0.563484	0.123639	0.523748
baron	BisqueRN CTGAN	ductal	900	-	-	0.118015	0.62823	0.116589	0.64501	0.118543	0.659511	0.110903	0.72736	0.11795	0.671924	0.114833	0.707658
baron	BisqueRN CTGAN	endothelia	900	-	-	0.125483	0.608183	0.122479	0.720044	0.128539	0.528813	0.123483	0.718227	0.125201	0.629398	0.123259	0.665511
baron	BisqueRN CTGAN	gamma	900	-	-	0.127054	0.57111	0.131399	0.490376	0.127406	0.602496	0.132401	0.454192	0.128172	0.5069	0.12349	0.715691
baron	BisqueRN CTGAN	macropha	900	-	-	0.084233	0.899246	0.085173	0.90548	0.083015	0.88851	0.08394	0.886609	0.087581	0.88766	0.083108	0.891231
baron	BisqueRN CTGAN	quiescent_	900	-	-	0.137607	0.494339	0.139484	0.432303	0.141316	0.416263	0.132601	0.676104	0.13886	0.442992	0.132908	0.678842
baron	BisqueRN CTGAN	acinar	1000	-	-	0.139583	0.885094	0.136758	0.898905	0.138132	0.898766	0.1379	0.891959	0.137317	0.89924	0.138568	0.893534
baron	BisqueRN CTGAN	activated_	1000	-	-	0.111288	0.813687	0.108713	0.7972	0.110938	0.762738	0.11167	0.80815	0.110488	0.798459	0.111723	0.81231
baron	BisqueRN CTGAN	alpha	1000	-	-	0.131684	0.918989	0.131761	0.919139	0.131877	0.919227	0.131681	0.918975	0.131635	0.918597	0.1318	0.919117
baron	BisqueRN CTGAN	beta	1000	-	-	0.170562	0.937542	0.17088	0.937141	0.17046	0.937946	0.171122	0.936481	0.171026	0.937044	0.171134	0.936612
baron	BisqueRN CTGAN	delta	1000	-	-	0.120057	0.586512	0.121576	0.531519	0.129064	0.434962	0.125463	0.4853	0.122694	0.561862	0.123544	0.523053
baron	BisqueRN CTGAN	ductal	1000	-	-	0.117473	0.634259	0.117242	0.631122	0.118427	0.67785	0.111422	0.725255	0.118307	0.672348	0.114392	0.719065
baron	BisqueRN CTGAN	endothelia	1000	-	-	0.125241	0.617932	0.12232	0.732678	0.12765	0.558562	0.123632	0.71882	0.124336	0.664685	0.12271	0.689656
baron	BisqueRN CTGAN	gamma	1000	-	-	0.127546	0.554717	0.130596	0.528936	0.127888	0.599457	0.133457	0.422946	0.128015	0.508145	0.124398	0.717473
baron	BisqueRN CTGAN	macropha	1000	-	-	0.084462	0.898354	0.084865	0.905416	0.083264	0.889423	0.083771	0.884079	0.087476	0.887927	0.083287	0.890658
baron	BisqueRN CTGAN	quiescent_	1000	-	-	0.137225	0.504543	0.13968	0.426709	0.141115	0.430117	0.13246	0.687785	0.13907	0.438076	0.132578	0.691819
baron	BisqueRN TVAE	acinar	100	-	-	0.13704	0.897134	0.137097	0.896511	0.136875	0.898849	0.13705	0.897211	0.137151	0.896762	0.137087	0.89708
baron	BisqueRN TVAE	activated_	100	-	-	0.112273	0.810225	0.111867	0.807154	0.111853	0.807322	0.110685	0.783989	0.111343	0.805858	0.112341	0.802516
baron	BisqueRN TVAE	alpha	100	-	-	0.131575	0.918555	0.131573	0.918487	0.131576	0.918531	0.131553	0.91846	0.131534	0.91852	0.131595	0.918392
baron	BisqueRN TVAE	beta	100	-	-	0.17095	0.937295	0.17087	0.937387	0.170977	0.937414	0.170969	0.937521	0.17093	0.937417	0.170991	0.93731
baron	BisqueRN TVAE	delta	100	-	-	0.126496	0.484212	0.126375	0.481728	0.123094	0.513899	0.12581	0.488716	0.126801	0.478244	0.124954	0.495612
baron	BisqueRN TVAE	ductal	100	-	-	0.125657	0.505563	0.127849	0.475703	0.128939	0.463019	0.126364	0.497001	0.12608	0.501627	0.128897	0.46386
baron	BisqueRN TVAE	endothelia	100	-	-	0.123973	0.647884	0.123563	0.583876	0.123749	0.602669	0.124164	0.617526	0.124939	0.65768	0.124582	0.613257
baron	BisqueRN TVAE	gamma	100	-	-	0.141595	0.192502	0.141892	0.181906	0.140857	0.203559	0.143234	0.136968	0.143001	0.149487	0.144827	0.089117
baron	BisqueRN TVAE	macropha	100	-	-	0.087267	0.876	0.086044	0.873901	0.08558	0.876552	0.087147	0.873259	0.088146	0.870406	0.084166	0.887911
baron	BisqueRN TVAE	quiescent_	100	-	-	0.135308	0.605656	0.138801	0.517651	0.134657	0.655872	0.136486	0.592655	0.133527	0.681899	0.138324	0.542374
baron	BisqueRN TVAE	acinar	200	-	-	0.136766	0.897349	0.136762	0.89611	0.13654	0.898139	0.136734	0.897357	0.13685	0.896729	0.136708	0.896345
baron	BisqueRN TVAE	activated_	200	-	-	0.112563	0.804036	0.112213	0.800929	0.112647	0.767323	0.111462	0.734645	0.111513	0.780778	0.113096	0.776592
baron	BisqueRN TVAE	alpha	200	-	-	0.131556	0.91848	0.131569	0.918266	0.131578	0.918289	0.131509	0.918332	0.131505	0.91852	0.13154	0.918256

baron	BisqueRN TVAE	beta	200	-	-	0.171003	0.937042	0.170965	0.937131	0.171086	0.93712	0.171037	0.937261	0.171033	0.937183	0.171038	0.937054
baron	BisqueRN TVAE	delta	200	-	-	0.12912	0.453092	0.128705	0.455321	0.125066	0.488551	0.127635	0.468339	0.128693	0.450361	0.127243	0.466242
baron	BisqueRN TVAE	ductal	200	-	-	0.130244	0.449179	0.13102	0.43388	0.133125	0.411082	0.12812	0.473656	0.130614	0.448571	0.134778	0.39263
baron	BisqueRN TVAE	endothelia	200	-	-	0.126024	0.613552	0.12535	0.560306	0.125428	0.57748	0.126031	0.592129	0.126725	0.627843	0.126889	0.567574
baron	BisqueRN TVAE	gamma	200	-	-	0.145792	0.066229	0.146618	0.031645	0.143482	0.117353	0.146757	0.02573	0.146998	0.026676	0.148146	-0.00589
baron	BisqueRN TVAE	macropha	200	-	-	0.086779	0.877044	0.085475	0.87221	0.085076	0.873908	0.086525	0.874208	0.088035	0.869798	0.083306	0.886212
baron	BisqueRN TVAE	quiescent_	200	-	-	0.136719	0.5889	0.139206	0.525782	0.135567	0.651775	0.137784	0.564846	0.134637	0.673539	0.138663	0.554506
baron	BisqueRN TVAE	acinar	300	-	-	0.136547	0.896893	0.136558	0.895911	0.136346	0.898013	0.136554	0.897026	0.136626	0.896563	0.136502	0.89568
baron	BisqueRN TVAE	activated_	300	-	-	0.112735	0.800118	0.112202	0.794661	0.113316	0.742209	0.111378	0.713205	0.111744	0.7662	0.11315	0.770176
baron	BisqueRN TVAE	alpha	300	-	-	0.131559	0.918554	0.131633	0.918169	0.131582	0.918235	0.131521	0.91811	0.131518	0.918509	0.131595	0.918146
baron	BisqueRN TVAE	beta	300	-	-	0.171036	0.93683	0.170983	0.936897	0.171138	0.936917	0.171071	0.937008	0.171051	0.936936	0.171085	0.936712
baron	BisqueRN TVAE	delta	300	-	-	0.129729	0.446212	0.129163	0.446075	0.12569	0.480082	0.128398	0.457206	0.129696	0.437424	0.12825	0.45279
baron	BisqueRN TVAE	ductal	300	-	-	0.131822	0.429801	0.131935	0.421366	0.134523	0.393161	0.129164	0.458708	0.131405	0.438659	0.136272	0.374514
baron	BisqueRN TVAE	endothelia	300	-	-	0.126885	0.602592	0.125922	0.553904	0.126053	0.575943	0.126654	0.572132	0.127061	0.626091	0.127232	0.564994
baron	BisqueRN TVAE	gamma	300	-	-	0.147431	0.01323	0.147103	0.013109	0.144634	0.080279	0.147825	-0.00698	0.148681	-0.01739	0.149259	-0.03525
baron	BisqueRN TVAE	macropha	300	-	-	0.086507	0.876397	0.08529	0.871499	0.08482	0.872641	0.086081	0.873805	0.087965	0.868302	0.082567	0.888532
baron	BisqueRN TVAE	quiescent_	300	-	-	0.136552	0.59906	0.138808	0.551304	0.135548	0.663193	0.138639	0.541639	0.134721	0.684982	0.139866	0.524747
baron	BisqueRN TVAE	acinar	400	-	-	0.136356	0.897168	0.136394	0.895411	0.136134	0.897632	0.136379	0.896762	0.136431	0.896501	0.136381	0.895774
baron	BisqueRN TVAE	activated_	400	-	-	0.112733	0.799661	0.112079	0.787322	0.112955	0.734222	0.111785	0.692712	0.111775	0.7536	0.11334	0.75233
baron	BisqueRN TVAE	alpha	400	-	-	0.131599	0.918444	0.131695	0.918121	0.131644	0.918155	0.131554	0.918045	0.131552	0.918447	0.131605	0.91814
baron	BisqueRN TVAE	beta	400	-	-	0.171095	0.936672	0.171012	0.936716	0.171151	0.936722	0.171116	0.93683	0.171072	0.936822	0.17113	0.936577
baron	BisqueRN TVAE	delta	400	-	-	0.130126	0.439117	0.129042	0.44431	0.126229	0.472586	0.128212	0.456791	0.129923	0.433052	0.128782	0.444377
baron	BisqueRN TVAE	ductal	400	-	-	0.132921	0.416837	0.132363	0.41388	0.134518	0.390958	0.129566	0.452872	0.131601	0.435032	0.136474	0.370477
baron	BisqueRN TVAE	endothelia	400	-	-	0.127382	0.59495	0.126153	0.545983	0.126237	0.565928	0.127037	0.565784	0.126973	0.621771	0.12731	0.561687
baron	BisqueRN TVAE	gamma	400	-	-	0.147705	0.006872	0.146691	0.018496	0.144859	0.071784	0.148413	-0.02658	0.14913	-0.02928	0.149943	-0.05265
baron	BisqueRN TVAE	macropha	400	-	-	0.08645	0.876195	0.085164	0.872164	0.084085	0.874378	0.085788	0.874563	0.08735	0.868783	0.082244	0.887102
baron	BisqueRN TVAE	quiescent_	400	-	-	0.136624	0.607044	0.139418	0.527719	0.136047	0.657356	0.139329	0.514684	0.135162	0.672676	0.139619	0.542603
baron	BisqueRN TVAE	acinar	500	-	-	0.136276	0.896794	0.136247	0.895564	0.136142	0.897128	0.136236	0.896483	0.136277	0.896515	0.136247	0.894883
baron	BisqueRN TVAE	activated_	500	-	-	0.112681	0.794396	0.112118	0.782193	0.113246	0.718917	0.111756	0.679516	0.111883	0.747572	0.113192	0.747004
baron	BisqueRN TVAE	alpha	500	-	-	0.131606	0.918455	0.131735	0.918047	0.131713	0.91809	0.13161	0.917913	0.131572	0.91848	0.131651	0.91803
baron	BisqueRN TVAE	beta	500	-	-	0.171084	0.936479	0.171056	0.936522	0.171189	0.936603	0.17111	0.93668	0.171073	0.936658	0.171159	0.936399
baron	BisqueRN TVAE	delta	500	-	-	0.130178	0.43865	0.128764	0.445422	0.125996	0.474719	0.128305	0.454901	0.129901	0.431998	0.128545	0.445365
baron	BisqueRN TVAE	ductal	500	-	-	0.132963	0.414418	0.132306	0.413449	0.134352	0.393091	0.128643	0.464536	0.131371	0.435544	0.13651	0.370262
baron	BisqueRN TVAE	endothelia	500	-	-	0.127311	0.593341	0.126178	0.543453	0.126185	0.568867	0.126994	0.563502	0.127507	0.612706	0.127433	0.561949
baron	BisqueRN TVAE	gamma	500	-	-	0.148035	-0.00167	0.147293	-0.00498	0.144425	0.080805	0.14865	-0.03485	0.149524	-0.04191	0.15024	-0.06088
baron	BisqueRN TVAE	macropha	500	-	-	0.086197	0.876733	0.084675	0.872869	0.084332	0.872749	0.08564	0.876013	0.087515	0.869792	0.082132	0.887354

baron	BisqueRN TVAE	quiescent_	500	-	-	0.137122	0.591779	0.139849	0.516026	0.135995	0.657673	0.139779	0.498273	0.134616	0.689485	0.139821	0.534585
baron	BisqueRN TVAE	acinar	600	-	-	0.136108	0.896881	0.136119	0.894993	0.13604	0.896498	0.13608	0.896236	0.136151	0.89617	0.136189	0.894375
baron	BisqueRN TVAE	activated_	600	-	-	0.112765	0.794685	0.112137	0.780231	0.113444	0.705155	0.111824	0.667226	0.111939	0.735841	0.113323	0.742099
baron	BisqueRN TVAE	alpha	600	-	-	0.131609	0.918444	0.131791	0.917985	0.131727	0.918069	0.131638	0.917909	0.131588	0.918471	0.131666	0.918007
baron	BisqueRN TVAE	beta	600	-	-	0.1711	0.936357	0.171052	0.936393	0.171196	0.936481	0.171146	0.936553	0.171097	0.936522	0.17118	0.936233
baron	BisqueRN TVAE	delta	600	-	-	0.130331	0.436906	0.128043	0.452282	0.125949	0.475331	0.128089	0.455717	0.130386	0.425122	0.128911	0.440698
baron	BisqueRN TVAE	ductal	600	-	-	0.132633	0.417324	0.131732	0.420079	0.134205	0.392647	0.128481	0.466601	0.131221	0.4364	0.136641	0.367556
baron	BisqueRN TVAE	endothelia	600	-	-	0.127347	0.592552	0.12626	0.538965	0.12635	0.564122	0.127114	0.558392	0.127387	0.613979	0.127556	0.562142
baron	BisqueRN TVAE	gamma	600	-	-	0.148155	-0.0061	0.146593	0.00598	0.144719	0.071979	0.148554	-0.03512	0.149658	-0.04345	0.150627	-0.0686
baron	BisqueRN TVAE	macropha	600	-	-	0.085933	0.87691	0.084595	0.872935	0.084108	0.872942	0.085545	0.875003	0.087254	0.869633	0.081641	0.88815
baron	BisqueRN TVAE	quiescent_	600	-	-	0.137187	0.592365	0.140347	0.503211	0.13643	0.643337	0.139848	0.496361	0.135162	0.676064	0.140161	0.5274
baron	BisqueRN TVAE	acinar	700	-	-	0.135946	0.896862	0.136093	0.894614	0.135874	0.896406	0.136051	0.896297	0.136062	0.895396	0.136059	0.894231
baron	BisqueRN TVAE	activated_	700	-	-	0.112704	0.792509	0.112073	0.774641	0.113434	0.705479	0.111887	0.658517	0.111904	0.731254	0.113172	0.740521
baron	BisqueRN TVAE	alpha	700	-	-	0.13163	0.918528	0.131808	0.917993	0.131754	0.918075	0.13169	0.917612	0.131623	0.91853	0.131699	0.917972
baron	BisqueRN TVAE	beta	700	-	-	0.171121	0.93631	0.171047	0.936353	0.171219	0.93634	0.171126	0.936447	0.171091	0.936448	0.171173	0.936162
baron	BisqueRN TVAE	delta	700	-	-	0.130374	0.435923	0.128307	0.448987	0.125835	0.477175	0.127789	0.459669	0.13	0.428628	0.12902	0.438472
baron	BisqueRN TVAE	ductal	700	-	-	0.13264	0.41601	0.131636	0.420576	0.13364	0.398149	0.127756	0.477348	0.131537	0.431509	0.136385	0.367294
baron	BisqueRN TVAE	endothelia	700	-	-	0.127295	0.594065	0.126511	0.531551	0.126264	0.562816	0.127218	0.555717	0.127567	0.610347	0.127685	0.557077
baron	BisqueRN TVAE	gamma	700	-	-	0.148078	-0.00415	0.146768	0.003281	0.143907	0.094522	0.148001	-0.02234	0.149612	-0.04448	0.150499	-0.06639
baron	BisqueRN TVAE	macropha	700	-	-	0.085965	0.876373	0.084489	0.872275	0.083408	0.875372	0.085679	0.874972	0.086956	0.869992	0.081657	0.887481
baron	BisqueRN TVAE	quiescent_	700	-	-	0.136742	0.609389	0.140074	0.514722	0.13668	0.645317	0.140003	0.492776	0.135056	0.68026	0.140125	0.531766
baron	BisqueRN TVAE	acinar	800	-	-	0.135879	0.897024	0.135982	0.894541	0.135881	0.895959	0.135961	0.895509	0.135944	0.895495	0.13609	0.893979
baron	BisqueRN TVAE	activated_	800	-	-	0.112617	0.789133	0.111916	0.764516	0.113205	0.697461	0.112073	0.646881	0.111994	0.725686	0.113256	0.727078
baron	BisqueRN TVAE	alpha	800	-	-	0.131646	0.918539	0.131853	0.917943	0.131766	0.918117	0.131704	0.917603	0.131638	0.918516	0.131727	0.917986
baron	BisqueRN TVAE	beta	800	-	-	0.171131	0.936148	0.171062	0.936207	0.171203	0.936323	0.171152	0.93637	0.171107	0.936333	0.171173	0.936103
baron	BisqueRN TVAE	delta	800	-	-	0.130262	0.435461	0.127498	0.457909	0.125506	0.481629	0.127409	0.461834	0.1299	0.430583	0.129076	0.437804
baron	BisqueRN TVAE	ductal	800	-	-	0.132893	0.412932	0.130775	0.432945	0.133128	0.403271	0.127967	0.475062	0.131278	0.43321	0.135434	0.376421
baron	BisqueRN TVAE	endothelia	800	-	-	0.127417	0.58993	0.126468	0.531423	0.126274	0.560527	0.127535	0.550185	0.127793	0.60545	0.127451	0.557796
baron	BisqueRN TVAE	gamma	800	-	-	0.147905	0.002071	0.146016	0.02232	0.143996	0.089341	0.148037	-0.02722	0.15009	-0.05724	0.150578	-0.06784
baron	BisqueRN TVAE	macropha	800	-	-	0.085659	0.877125	0.084559	0.872103	0.083701	0.873303	0.0854	0.874723	0.086845	0.869513	0.081609	0.88687
baron	BisqueRN TVAE	quiescent_	800	-	-	0.137059	0.599456	0.140462	0.498107	0.136744	0.643071	0.139836	0.500469	0.135039	0.678899	0.140589	0.515326
baron	BisqueRN TVAE	acinar	900	-	-	0.13585	0.89673	0.135918	0.893985	0.135787	0.895769	0.135847	0.895831	0.135828	0.895448	0.135971	0.893629
baron	BisqueRN TVAE	activated_	900	-	-	0.112656	0.78992	0.111956	0.765523	0.113251	0.69136	0.112009	0.647049	0.112015	0.717276	0.11316	0.724815
baron	BisqueRN TVAE	alpha	900	-	-	0.131655	0.918505	0.13187	0.917948	0.131812	0.918089	0.131729	0.917644	0.131662	0.918487	0.131764	0.917876
baron	BisqueRN TVAE	beta	900	-	-	0.171122	0.936125	0.171058	0.936141	0.171199	0.93623	0.17114	0.936308	0.171085	0.936306	0.171175	0.935976
baron	BisqueRN TVAE	delta	900	-	-	0.130146	0.436989	0.126934	0.464381	0.125858	0.477032	0.126884	0.468003	0.129141	0.438096	0.128844	0.440894

baron	BisqueRN	TVAE	ductal	900	-	-	0.132747	0.414577	0.130304	0.440386	0.132534	0.410029	0.127483	0.482901	0.130415	0.443185	0.135016	0.379735
baron	BisqueRN	TVAE	endothelia	900	-	-	0.127358	0.590782	0.126716	0.523953	0.126301	0.55457	0.127591	0.545146	0.127521	0.602902	0.127415	0.556523
baron	BisqueRN	TVAE	gamma	900	-	-	0.148025	-0.0004	0.145612	0.027342	0.143828	0.095497	0.147376	-0.01184	0.149048	-0.03749	0.150595	-0.06991
baron	BisqueRN	TVAE	macropha	900	-	-	0.085671	0.876271	0.084137	0.873465	0.083607	0.874244	0.08537	0.875255	0.086948	0.869315	0.081268	0.888076
baron	BisqueRN	TVAE	quiescent_	900	-	-	0.136964	0.602841	0.14063	0.497635	0.13687	0.638305	0.139722	0.504005	0.135039	0.681909	0.140726	0.512272
baron	BisqueRN	TVAE	acinar	1000	-	-	0.135754	0.896793	0.135867	0.89378	0.135711	0.895735	0.135822	0.895473	0.135769	0.895496	0.135928	0.893314
baron	BisqueRN	TVAE	activated_	1000	-	-	0.112595	0.789565	0.111884	0.76099	0.112943	0.68772	0.112157	0.639263	0.112059	0.71552	0.11323	0.724899
baron	BisqueRN	TVAE	alpha	1000	-	-	0.131661	0.918566	0.131859	0.917976	0.131819	0.918065	0.131774	0.917374	0.131682	0.918475	0.13176	0.917944
baron	BisqueRN	TVAE	beta	1000	-	-	0.171155	0.936029	0.171049	0.936084	0.171228	0.936185	0.171151	0.936214	0.17108	0.936228	0.17119	0.9359
baron	BisqueRN	TVAE	delta	1000	-	-	0.129858	0.439184	0.127123	0.461948	0.12557	0.481274	0.12693	0.466524	0.129173	0.43763	0.129032	0.437811
baron	BisqueRN	TVAE	ductal	1000	-	-	0.13243	0.417773	0.130444	0.438001	0.131985	0.418121	0.127578	0.482431	0.129901	0.449651	0.135425	0.374442
baron	BisqueRN	TVAE	endothelia	1000	-	-	0.1274	0.590696	0.126913	0.517767	0.126313	0.559164	0.127557	0.544497	0.127603	0.601953	0.127561	0.554798
baron	BisqueRN	TVAE	gamma	1000	-	-	0.147664	0.004284	0.145724	0.026242	0.143576	0.102396	0.147464	-0.01571	0.149008	-0.03433	0.150724	-0.07387
baron	BisqueRN	TVAE	macropha	1000	-	-	0.085697	0.877046	0.0845	0.872876	0.083464	0.87395	0.085442	0.874904	0.087005	0.869457	0.081376	0.887249
baron	BisqueRN	TVAE	quiescent_	1000	-	-	0.13697	0.601042	0.140901	0.483503	0.136808	0.64438	0.139753	0.502572	0.135014	0.683466	0.140361	0.523335
baron	BisqueRN	sc-CMGAI	acinar	100	-	-	0.137111	0.899191	0.137607	0.897696	0.137372	0.897982	0.137313	0.898722	0.137593	0.898578	0.13725	0.898085
baron	BisqueRN	sc-CMGAI	activated_	100	-	-	0.110855	0.805763	0.111158	0.817705	0.111133	0.807525	0.11066	0.809413	0.111013	0.81191	0.111713	0.810883
baron	BisqueRN	sc-CMGAI	alpha	100	-	-	0.131735	0.918695	0.131698	0.918862	0.131721	0.918718	0.131699	0.91878	0.131936	0.917859	0.131732	0.9189
baron	BisqueRN	sc-CMGAI	beta	100	-	-	0.170965	0.93634	0.171046	0.936199	0.170961	0.936544	0.17086	0.936867	0.170982	0.936804	0.17093	0.93664
baron	BisqueRN	sc-CMGAI	delta	100	-	-	0.118746	0.564013	0.117078	0.587942	0.120033	0.554524	0.118361	0.570475	0.11984	0.556963	0.119577	0.556394
baron	BisqueRN	sc-CMGAI	ductal	100	-	-	0.115152	0.677783	0.117225	0.644352	0.115834	0.667564	0.115823	0.667676	0.117163	0.639821	0.116907	0.666484
baron	BisqueRN	sc-CMGAI	endothelia	100	-	-	0.121813	0.756869	0.120864	0.73185	0.120045	0.748368	0.122434	0.70841	0.12226	0.73596	0.123124	0.669071
baron	BisqueRN	sc-CMGAI	gamma	100	-	-	0.126903	0.682782	0.126779	0.670335	0.125539	0.733114	0.12568	0.737093	0.127239	0.696037	0.129802	0.60806
baron	BisqueRN	sc-CMGAI	macropha	100	-	-	0.086296	0.894035	0.084145	0.89398	0.082785	0.895854	0.083654	0.898424	0.085859	0.886276	0.083944	0.901444
baron	BisqueRN	sc-CMGAI	quiescent_	100	-	-	0.134922	0.557371	0.130665	0.6933	0.13477	0.595794	0.132192	0.644978	0.131829	0.656649	0.133165	0.634656
baron	BisqueRN	sc-CMGAI	acinar	200	-	-	0.137051	0.898089	0.137614	0.897204	0.137	0.898154	0.137285	0.8986	0.137694	0.897337	0.137063	0.899137
baron	BisqueRN	sc-CMGAI	activated_	200	-	-	0.11085	0.804279	0.1114	0.814002	0.111045	0.803377	0.109867	0.816329	0.111318	0.809018	0.112	0.808353
baron	BisqueRN	sc-CMGAI	alpha	200	-	-	0.131801	0.91862	0.131772	0.91865	0.131781	0.918619	0.131752	0.918818	0.131945	0.917796	0.131737	0.918826
baron	BisqueRN	sc-CMGAI	beta	200	-	-	0.170976	0.936378	0.1711	0.936244	0.170973	0.936276	0.170935	0.936495	0.171082	0.936456	0.170968	0.936622
baron	BisqueRN	sc-CMGAI	delta	200	-	-	0.120196	0.547354	0.120015	0.55706	0.122108	0.527431	0.119265	0.56148	0.121459	0.541813	0.122517	0.521058
baron	BisqueRN	sc-CMGAI	ductal	200	-	-	0.117882	0.633582	0.119247	0.614997	0.11747	0.635327	0.117436	0.639266	0.120164	0.593832	0.119044	0.631768
baron	BisqueRN	sc-CMGAI	endothelia	200	-	-	0.124152	0.715381	0.121482	0.730774	0.12303	0.659059	0.124734	0.661316	0.124402	0.708456	0.124842	0.644849
baron	BisqueRN	sc-CMGAI	gamma	200	-	-	0.129317	0.635089	0.127737	0.671266	0.127709	0.691526	0.127568	0.715947	0.126546	0.708553	0.13038	0.608726
baron	BisqueRN	sc-CMGAI	macropha	200	-	-	0.08615	0.892495	0.083898	0.891778	0.082477	0.893887	0.083639	0.897108	0.085385	0.887168	0.084083	0.900974
baron	BisqueRN	sc-CMGAI	quiescent_	200	-	-	0.135151	0.559829	0.131641	0.694214	0.136181	0.570446	0.133363	0.644509	0.13273	0.647678	0.133256	0.648065
baron	BisqueRN	sc-CMGAI	acinar	300	-	-	0.136883	0.89863	0.137607	0.896944	0.137101	0.897969	0.137236	0.898426	0.137768	0.897693	0.137067	0.898698

baron	BisqueRN	sc-CMGAI	activated_	300	-	-	0.110633	0.80249	0.11099	0.814459	0.110878	0.801743	0.11016	0.812685	0.111123	0.807533	0.111916	0.809762
baron	BisqueRN	sc-CMGAI	alpha	300	-	-	0.131765	0.918461	0.131739	0.918689	0.131679	0.918662	0.131709	0.918716	0.131988	0.917216	0.131769	0.918673
baron	BisqueRN	sc-CMGAI	beta	300	-	-	0.170922	0.936114	0.171049	0.936353	0.170976	0.936193	0.170958	0.936511	0.171165	0.936474	0.170969	0.936654
baron	BisqueRN	sc-CMGAI	delta	300	-	-	0.122376	0.522386	0.119873	0.561063	0.122969	0.517276	0.12002	0.554741	0.121638	0.537775	0.122926	0.516976
baron	BisqueRN	sc-CMGAI	ductal	300	-	-	0.118344	0.628233	0.119892	0.602236	0.118139	0.626885	0.118194	0.626129	0.120014	0.593373	0.118605	0.63223
baron	BisqueRN	sc-CMGAI	endothelia	300	-	-	0.12497	0.696716	0.122391	0.676539	0.121636	0.698532	0.125208	0.64493	0.125207	0.684596	0.126836	0.612008
baron	BisqueRN	sc-CMGAI	gamma	300	-	-	0.129082	0.651173	0.129441	0.650293	0.128313	0.697098	0.126923	0.72894	0.128384	0.670881	0.130728	0.594748
baron	BisqueRN	sc-CMGAI	macropha	300	-	-	0.086167	0.891273	0.082937	0.892091	0.081959	0.8949	0.082948	0.900801	0.085232	0.886436	0.083245	0.900627
baron	BisqueRN	sc-CMGAI	quiescent_	300	-	-	0.136032	0.541569	0.132592	0.699669	0.137627	0.540066	0.133619	0.64285	0.132107	0.660824	0.133502	0.655577
baron	BisqueRN	sc-CMGAI	acinar	400	-	-	0.136719	0.899021	0.137809	0.894914	0.136989	0.898159	0.137289	0.897754	0.1376	0.898012	0.137008	0.898709
baron	BisqueRN	sc-CMGAI	activated_	400	-	-	0.110781	0.80259	0.110994	0.813715	0.111014	0.797835	0.109841	0.811569	0.111185	0.807492	0.111985	0.806183
baron	BisqueRN	sc-CMGAI	alpha	400	-	-	0.131788	0.918654	0.131705	0.918673	0.131771	0.918756	0.13172	0.918671	0.131988	0.917138	0.13179	0.918616
baron	BisqueRN	sc-CMGAI	beta	400	-	-	0.170984	0.936042	0.171077	0.936115	0.170979	0.93608	0.171011	0.936221	0.171079	0.936328	0.170971	0.936244
baron	BisqueRN	sc-CMGAI	delta	400	-	-	0.12102	0.539065	0.119965	0.568902	0.123896	0.507257	0.120673	0.553189	0.123109	0.522075	0.123811	0.50544
baron	BisqueRN	sc-CMGAI	ductal	400	-	-	0.11871	0.624429	0.120156	0.595839	0.119618	0.604557	0.118916	0.613282	0.120842	0.581165	0.118928	0.635798
baron	BisqueRN	sc-CMGAI	endothelia	400	-	-	0.125325	0.684654	0.123085	0.662364	0.12342	0.650228	0.12544	0.640055	0.124998	0.683464	0.127202	0.582936
baron	BisqueRN	sc-CMGAI	gamma	400	-	-	0.129754	0.634075	0.12856	0.647467	0.127452	0.708987	0.126542	0.736235	0.127437	0.7077	0.130446	0.610044
baron	BisqueRN	sc-CMGAI	macropha	400	-	-	0.086442	0.891225	0.082971	0.89095	0.081956	0.892848	0.082757	0.900102	0.084639	0.888074	0.083332	0.901288
baron	BisqueRN	sc-CMGAI	quiescent_	400	-	-	0.137183	0.513207	0.132775	0.688835	0.138706	0.505043	0.133859	0.630594	0.133421	0.634853	0.133848	0.653562
baron	BisqueRN	sc-CMGAI	acinar	500	-	-	0.136863	0.898543	0.137488	0.896441	0.137074	0.897727	0.137255	0.898297	0.137533	0.897636	0.136928	0.899332
baron	BisqueRN	sc-CMGAI	activated_	500	-	-	0.110813	0.801177	0.110779	0.812839	0.110904	0.797227	0.110142	0.81013	0.111188	0.805346	0.11188	0.807549
baron	BisqueRN	sc-CMGAI	alpha	500	-	-	0.131795	0.918493	0.131685	0.918553	0.131754	0.918776	0.131718	0.91865	0.131879	0.917675	0.131766	0.918672
baron	BisqueRN	sc-CMGAI	beta	500	-	-	0.170944	0.935887	0.171065	0.93618	0.170994	0.936187	0.171021	0.936109	0.171072	0.93631	0.17098	0.936388
baron	BisqueRN	sc-CMGAI	delta	500	-	-	0.122133	0.525391	0.120175	0.563966	0.12332	0.515806	0.120532	0.552255	0.121772	0.544684	0.123387	0.512695
baron	BisqueRN	sc-CMGAI	ductal	500	-	-	0.11874	0.626675	0.121015	0.587667	0.119055	0.614373	0.118895	0.616929	0.12	0.593488	0.118763	0.642925
baron	BisqueRN	sc-CMGAI	endothelia	500	-	-	0.125612	0.679217	0.124338	0.624471	0.124319	0.638663	0.126183	0.624482	0.125178	0.683611	0.127103	0.57874
baron	BisqueRN	sc-CMGAI	gamma	500	-	-	0.129485	0.644295	0.127302	0.697455	0.127825	0.718165	0.127742	0.722063	0.127146	0.742022	0.129528	0.6296
baron	BisqueRN	sc-CMGAI	macropha	500	-	-	0.086407	0.892051	0.082552	0.891871	0.08191	0.892327	0.083373	0.899123	0.084875	0.887335	0.083196	0.902378
baron	BisqueRN	sc-CMGAI	quiescent_	500	-	-	0.137338	0.506812	0.132888	0.685928	0.137756	0.538818	0.134041	0.631287	0.132934	0.648676	0.134602	0.637645
baron	BisqueRN	sc-CMGAI	acinar	600	-	-	0.136808	0.898886	0.137704	0.895841	0.136827	0.89806	0.13717	0.897419	0.137694	0.897302	0.137156	0.89885
baron	BisqueRN	sc-CMGAI	activated_	600	-	-	0.110408	0.800677	0.110922	0.811793	0.110906	0.792814	0.110073	0.810603	0.11094	0.808858	0.111962	0.805767
baron	BisqueRN	sc-CMGAI	alpha	600	-	-	0.131831	0.918646	0.131758	0.918602	0.131753	0.918572	0.131715	0.918837	0.131997	0.917044	0.131761	0.91862
baron	BisqueRN	sc-CMGAI	beta	600	-	-	0.170982	0.935793	0.17109	0.936046	0.170994	0.936061	0.170949	0.936327	0.171106	0.936246	0.170974	0.936421
baron	BisqueRN	sc-CMGAI	delta	600	-	-	0.122002	0.526997	0.12033	0.56958	0.12435	0.507549	0.120527	0.552803	0.122529	0.540079	0.123826	0.507975
baron	BisqueRN	sc-CMGAI	ductal	600	-	-	0.119	0.621784	0.121282	0.582332	0.118939	0.612686	0.11946	0.604148	0.121319	0.574642	0.119383	0.635515
baron	BisqueRN	sc-CMGAI	endothelia	600	-	-	0.12601	0.675496	0.125667	0.592742	0.122946	0.66118	0.127592	0.56498	0.125597	0.675947	0.127938	0.566979

baron	BisqueRN	sc-CMGAI	gamma	600	-	-	0.130301	0.635482	0.127745	0.649058	0.126401	0.714218	0.127662	0.73426	0.12693	0.727451	0.130186	0.614322
baron	BisqueRN	sc-CMGAI	macropha	600	-	-	0.086619	0.889234	0.082989	0.891128	0.082238	0.888649	0.082722	0.899178	0.085207	0.885302	0.083284	0.901249
baron	BisqueRN	sc-CMGAI	quiescent_	600	-	-	0.136747	0.528375	0.133094	0.678437	0.139102	0.489445	0.13423	0.627823	0.133327	0.638678	0.134438	0.635195
baron	BisqueRN	sc-CMGAI	acinar	700	-	-	0.136546	0.899059	0.137529	0.89648	0.136945	0.897661	0.137262	0.897533	0.137677	0.897005	0.136808	0.899472
baron	BisqueRN	sc-CMGAI	activated_	700	-	-	0.110774	0.801195	0.11099	0.811265	0.110871	0.794042	0.109846	0.805125	0.111081	0.8069	0.111918	0.806768
baron	BisqueRN	sc-CMGAI	alpha	700	-	-	0.131815	0.918733	0.131735	0.918548	0.131762	0.91868	0.131744	0.918718	0.131882	0.917141	0.131801	0.91842
baron	BisqueRN	sc-CMGAI	beta	700	-	-	0.170923	0.935791	0.171087	0.93618	0.171028	0.935864	0.170965	0.936269	0.171089	0.936062	0.171014	0.936207
baron	BisqueRN	sc-CMGAI	delta	700	-	-	0.121887	0.528643	0.120028	0.572617	0.123361	0.518928	0.120223	0.56092	0.12244	0.537714	0.123938	0.507752
baron	BisqueRN	sc-CMGAI	ductal	700	-	-	0.118882	0.634053	0.120446	0.600311	0.119313	0.610215	0.118921	0.617314	0.120767	0.582149	0.118978	0.644389
baron	BisqueRN	sc-CMGAI	endothelia	700	-	-	0.125902	0.666311	0.124922	0.618409	0.124385	0.627132	0.127108	0.582277	0.125772	0.667848	0.128187	0.569401
baron	BisqueRN	sc-CMGAI	gamma	700	-	-	0.129535	0.65856	0.12666	0.68378	0.126975	0.71133	0.126553	0.747814	0.12739	0.73393	0.129304	0.626827
baron	BisqueRN	sc-CMGAI	macropha	700	-	-	0.08713	0.886094	0.083031	0.891177	0.081959	0.890612	0.082398	0.899938	0.084591	0.886394	0.083098	0.902262
baron	BisqueRN	sc-CMGAI	quiescent_	700	-	-	0.136971	0.52335	0.132488	0.688907	0.138636	0.511542	0.134252	0.627229	0.133646	0.631744	0.134297	0.646987
baron	BisqueRN	sc-CMGAI	acinar	800	-	-	0.136597	0.898927	0.137781	0.895389	0.136939	0.897643	0.137222	0.897576	0.137677	0.896858	0.137057	0.898769
baron	BisqueRN	sc-CMGAI	activated_	800	-	-	0.110515	0.800039	0.111006	0.809964	0.110896	0.791537	0.110081	0.80603	0.111254	0.806676	0.111962	0.80716
baron	BisqueRN	sc-CMGAI	alpha	800	-	-	0.1318	0.918762	0.131741	0.91863	0.131734	0.918648	0.131725	0.918743	0.131903	0.917477	0.13179	0.918577
baron	BisqueRN	sc-CMGAI	beta	800	-	-	0.170968	0.935704	0.171048	0.936107	0.170999	0.935875	0.171036	0.936237	0.171081	0.936145	0.170993	0.936296
baron	BisqueRN	sc-CMGAI	delta	800	-	-	0.12194	0.528199	0.11952	0.591126	0.123712	0.515097	0.120444	0.564306	0.122837	0.539748	0.124293	0.503977
baron	BisqueRN	sc-CMGAI	ductal	800	-	-	0.118962	0.629975	0.120611	0.594843	0.119086	0.612677	0.119196	0.612221	0.121352	0.576395	0.119261	0.644293
baron	BisqueRN	sc-CMGAI	endothelia	800	-	-	0.126094	0.672764	0.125596	0.589385	0.124558	0.61496	0.126524	0.605237	0.125833	0.661452	0.128485	0.553871
baron	BisqueRN	sc-CMGAI	gamma	800	-	-	0.129914	0.64507	0.127718	0.634728	0.127203	0.710912	0.127279	0.691634	0.126755	0.737346	0.128672	0.636407
baron	BisqueRN	sc-CMGAI	macropha	800	-	-	0.08687	0.887878	0.082901	0.890461	0.081385	0.892734	0.082821	0.899041	0.084732	0.88677	0.083335	0.902183
baron	BisqueRN	sc-CMGAI	quiescent_	800	-	-	0.137049	0.518172	0.132799	0.686422	0.138446	0.523318	0.134464	0.619863	0.133492	0.631817	0.134491	0.633331
baron	BisqueRN	sc-CMGAI	acinar	900	-	-	0.136518	0.899326	0.137287	0.896806	0.1369	0.897925	0.137112	0.897756	0.137609	0.897462	0.136799	0.899567
baron	BisqueRN	sc-CMGAI	activated_	900	-	-	0.11068	0.80031	0.11097	0.804646	0.110866	0.790089	0.109884	0.805635	0.111187	0.807145	0.111978	0.805005
baron	BisqueRN	sc-CMGAI	alpha	900	-	-	0.131803	0.9188	0.131775	0.918691	0.131767	0.918753	0.131749	0.918757	0.1319	0.917458	0.131821	0.918246
baron	BisqueRN	sc-CMGAI	beta	900	-	-	0.170978	0.935658	0.171099	0.936038	0.17102	0.936001	0.171002	0.936344	0.171124	0.936059	0.171004	0.936353
baron	BisqueRN	sc-CMGAI	delta	900	-	-	0.121991	0.527516	0.119507	0.585386	0.123647	0.518773	0.120253	0.561577	0.122263	0.546652	0.123658	0.511429
baron	BisqueRN	sc-CMGAI	ductal	900	-	-	0.11881	0.636517	0.121063	0.59275	0.119153	0.614126	0.119208	0.614849	0.120602	0.587645	0.118647	0.654398
baron	BisqueRN	sc-CMGAI	endothelia	900	-	-	0.125705	0.69663	0.124907	0.605582	0.124998	0.606154	0.127289	0.579753	0.125522	0.67065	0.128281	0.563903
baron	BisqueRN	sc-CMGAI	gamma	900	-	-	0.129606	0.662993	0.12744	0.649453	0.126073	0.743111	0.127211	0.732914	0.126752	0.732047	0.129119	0.618136
baron	BisqueRN	sc-CMGAI	macropha	900	-	-	0.086618	0.888616	0.082847	0.890118	0.081843	0.891742	0.082908	0.899923	0.084471	0.887288	0.083079	0.901407
baron	BisqueRN	sc-CMGAI	quiescent_	900	-	-	0.136705	0.532476	0.132929	0.684785	0.139371	0.48611	0.134219	0.623994	0.13318	0.640877	0.134455	0.637837
baron	BisqueRN	sc-CMGAI	acinar	1000	-	-	0.136408	0.899121	0.137568	0.895539	0.13679	0.898298	0.137215	0.897638	0.137592	0.897426	0.13683	0.899613
baron	BisqueRN	sc-CMGAI	activated_	1000	-	-	0.110702	0.80039	0.110942	0.808406	0.110742	0.789487	0.110055	0.803003	0.111034	0.806037	0.111878	0.805981
baron	BisqueRN	sc-CMGAI	alpha	1000	-	-	0.131805	0.918656	0.131728	0.91864	0.131765	0.918733	0.131743	0.918721	0.13194	0.917535	0.131787	0.918514

baron	BisqueRN	sc-CMGAI	beta	1000	-	-	0.170978	0.935574	0.171085	0.936102	0.171002	0.935985	0.170962	0.936421	0.171075	0.936084	0.171006	0.936277
baron	BisqueRN	sc-CMGAI	delta	1000	-	-	0.122325	0.523587	0.11989	0.57659	0.124128	0.513843	0.119901	0.568739	0.122534	0.547177	0.124052	0.506397
baron	BisqueRN	sc-CMGAI	ductal	1000	-	-	0.118707	0.642793	0.120912	0.591788	0.119321	0.609997	0.119034	0.616271	0.120889	0.582598	0.118789	0.658776
baron	BisqueRN	sc-CMGAI	endothelia	1000	-	-	0.125353	0.698063	0.125779	0.584373	0.124864	0.602303	0.127446	0.56904	0.125663	0.664368	0.128286	0.567067
baron	BisqueRN	sc-CMGAI	gamma	1000	-	-	0.129424	0.673706	0.127193	0.652355	0.126269	0.711124	0.127088	0.717328	0.126583	0.748658	0.128781	0.63222
baron	BisqueRN	sc-CMGAI	macropha	1000	-	-	0.086704	0.886076	0.082504	0.89172	0.081586	0.89081	0.082922	0.899615	0.08481	0.88647	0.083208	0.901317
baron	BisqueRN	sc-CMGAI	quiescent_	1000	-	-	0.1366	0.536494	0.133257	0.674037	0.139203	0.493611	0.134574	0.615371	0.133321	0.633284	0.134478	0.641499
GSE81547	SCDC	Control	acinar	0	0.117974	0.758204	-	-	-	-	-	-	-	-	-	-	-	-
GSE81547	SCDC	Control	alpha	0	0.107455	0.861384	-	-	-	-	-	-	-	-	-	-	-	-
GSE81547	SCDC	Control	beta	0	0.11858	0.731147	-	-	-	-	-	-	-	-	-	-	-	-
GSE81547	SCDC	Control	delta	0	0.085358	0.82089	-	-	-	-	-	-	-	-	-	-	-	-
GSE81547	SCDC	Control	ductal	0	0.247537	0.876418	-	-	-	-	-	-	-	-	-	-	-	-
GSE81547	SCDC	sc-CMGAI	acinar	100	-	-	0.119805	0.758611	0.117052	0.764003	0.116818	0.768271	0.116762	0.765484	0.117894	0.764126	0.11692	0.767448
GSE81547	SCDC	sc-CMGAI	alpha	100	-	-	0.10655	0.844245	0.106506	0.850477	0.101838	0.855875	0.097674	0.860012	0.106176	0.850411	0.105501	0.854035
GSE81547	SCDC	sc-CMGAI	beta	100	-	-	0.104495	0.730022	0.104886	0.724502	0.106292	0.705474	0.107979	0.719527	0.104734	0.733626	0.098939	0.743193
GSE81547	SCDC	sc-CMGAI	delta	100	-	-	0.092606	0.80715	0.095726	0.795782	0.098663	0.782228	0.097137	0.782341	0.091955	0.803002	0.098012	0.809189
GSE81547	SCDC	sc-CMGAI	ductal	100	-	-	0.099759	0.878403	0.100609	0.878539	0.106755	0.87933	0.098508	0.876575	0.100964	0.881678	0.108575	0.880852
GSE81547	SCDC	sc-CMGAI	acinar	200	-	-	0.11855	0.760941	0.117474	0.761764	0.117502	0.767131	0.117228	0.764908	0.118081	0.762099	0.117249	0.768297
GSE81547	SCDC	sc-CMGAI	alpha	200	-	-	0.108657	0.848468	0.101678	0.855607	0.104639	0.856521	0.097484	0.859536	0.108182	0.851915	0.107781	0.852599
GSE81547	SCDC	sc-CMGAI	beta	200	-	-	0.102917	0.738846	0.106054	0.726941	0.106391	0.709717	0.107064	0.725431	0.105584	0.734391	0.099719	0.741943
GSE81547	SCDC	sc-CMGAI	delta	200	-	-	0.090081	0.81328	0.095093	0.791574	0.095613	0.790661	0.094849	0.783172	0.088767	0.80415	0.093833	0.815566
GSE81547	SCDC	sc-CMGAI	ductal	200	-	-	0.11086	0.881097	0.108446	0.879689	0.106637	0.879645	0.110165	0.878223	0.109722	0.882612	0.113823	0.882183
GSE81547	SCDC	sc-CMGAI	acinar	300	-	-	0.118589	0.761942	0.117522	0.761602	0.117653	0.767489	0.116858	0.764732	0.117828	0.763603	0.11688	0.768325
GSE81547	SCDC	sc-CMGAI	alpha	300	-	-	0.106453	0.847627	0.104693	0.854592	0.106101	0.854637	0.098393	0.856507	0.106907	0.850987	0.107386	0.853833
GSE81547	SCDC	sc-CMGAI	beta	300	-	-	0.103704	0.740723	0.104561	0.730719	0.107821	0.707672	0.107365	0.728963	0.106185	0.734322	0.099927	0.743339
GSE81547	SCDC	sc-CMGAI	delta	300	-	-	0.09038	0.811104	0.093134	0.796363	0.094062	0.792658	0.093514	0.789787	0.088541	0.806373	0.093682	0.812742
GSE81547	SCDC	sc-CMGAI	ductal	300	-	-	0.112775	0.882321	0.11288	0.880546	0.10488	0.87922	0.11048	0.878275	0.11191	0.882555	0.114868	0.881741
GSE81547	SCDC	sc-CMGAI	acinar	400	-	-	0.118616	0.76135	0.117625	0.761007	0.118344	0.764609	0.11696	0.766342	0.117886	0.763921	0.116738	0.769103
GSE81547	SCDC	sc-CMGAI	alpha	400	-	-	0.108683	0.848133	0.103354	0.854939	0.104324	0.855485	0.102097	0.854879	0.109104	0.849362	0.106899	0.854529
GSE81547	SCDC	sc-CMGAI	beta	400	-	-	0.102573	0.745335	0.105456	0.728824	0.107074	0.709251	0.107128	0.727638	0.105521	0.735868	0.099849	0.74279
GSE81547	SCDC	sc-CMGAI	delta	400	-	-	0.089647	0.81338	0.093197	0.793917	0.094825	0.789443	0.091689	0.796584	0.088044	0.809305	0.09297	0.813774
GSE81547	SCDC	sc-CMGAI	ductal	400	-	-	0.113608	0.882518	0.114043	0.880672	0.106625	0.879166	0.107912	0.878779	0.109774	0.882851	0.11596	0.882324
GSE81547	SCDC	sc-CMGAI	acinar	500	-	-	0.118725	0.761935	0.117489	0.762226	0.118243	0.764998	0.11709	0.765048	0.118106	0.761991	0.116872	0.768729
GSE81547	SCDC	sc-CMGAI	alpha	500	-	-	0.105509	0.84792	0.105648	0.853594	0.104531	0.85739	0.100935	0.856793	0.108499	0.849881	0.107715	0.853925
GSE81547	SCDC	sc-CMGAI	beta	500	-	-	0.103247	0.7383	0.104424	0.736418	0.107535	0.710189	0.106132	0.732637	0.105696	0.736303	0.09989	0.74261
GSE81547	SCDC	sc-CMGAI	delta	500	-	-	0.091444	0.80895	0.091649	0.799514	0.09383	0.789924	0.092354	0.796266	0.088243	0.808697	0.093617	0.812848

GSE81547	SCDC	sc-CMGAI	ductal	500	-	-	0.113072	0.881563	0.114172	0.880593	0.107464	0.879757	0.11031	0.877736	0.111129	0.882973	0.115255	0.88234
GSE81547	SCDC	sc-CMGAI	acinar	600	-	-	0.119071	0.759606	0.117833	0.760772	0.117995	0.76574	0.116729	0.765557	0.118063	0.76252	0.116885	0.768757
GSE81547	SCDC	sc-CMGAI	alpha	600	-	-	0.107103	0.849012	0.102911	0.853611	0.103135	0.856233	0.100278	0.85574	0.108845	0.849455	0.106644	0.854322
GSE81547	SCDC	sc-CMGAI	beta	600	-	-	0.103539	0.740545	0.10502	0.732549	0.107763	0.70871	0.106564	0.731724	0.106021	0.736556	0.100003	0.742105
GSE81547	SCDC	sc-CMGAI	delta	600	-	-	0.09008	0.811994	0.092986	0.793132	0.094896	0.78822	0.092596	0.793267	0.088287	0.807151	0.093842	0.811912
GSE81547	SCDC	sc-CMGAI	ductal	600	-	-	0.114196	0.882779	0.11594	0.88007	0.107459	0.879536	0.110366	0.878616	0.109288	0.883201	0.117395	0.882248
GSE81547	SCDC	sc-CMGAI	acinar	700	-	-	0.118409	0.762625	0.117648	0.761139	0.117719	0.765932	0.11717	0.764395	0.117765	0.763176	0.116896	0.768554
GSE81547	SCDC	sc-CMGAI	alpha	700	-	-	0.1082	0.846501	0.104389	0.854826	0.103204	0.857504	0.099391	0.857865	0.109361	0.849052	0.107585	0.853113
GSE81547	SCDC	sc-CMGAI	beta	700	-	-	0.102571	0.747224	0.104856	0.734301	0.107727	0.712314	0.106983	0.731097	0.105001	0.738929	0.100142	0.742095
GSE81547	SCDC	sc-CMGAI	delta	700	-	-	0.089742	0.81378	0.092313	0.796487	0.094371	0.789673	0.092667	0.793062	0.088194	0.807479	0.093718	0.81185
GSE81547	SCDC	sc-CMGAI	ductal	700	-	-	0.113944	0.882104	0.114818	0.880629	0.108036	0.880824	0.110552	0.878987	0.111991	0.883172	0.115677	0.882806
GSE81547	SCDC	sc-CMGAI	acinar	800	-	-	0.118867	0.76054	0.117756	0.760516	0.117784	0.766192	0.117034	0.76523	0.118182	0.762528	0.117151	0.767339
GSE81547	SCDC	sc-CMGAI	alpha	800	-	-	0.107648	0.84724	0.104859	0.854472	0.102924	0.857379	0.099093	0.857647	0.109619	0.849253	0.107775	0.852944
GSE81547	SCDC	sc-CMGAI	beta	800	-	-	0.103015	0.744192	0.104475	0.735839	0.107443	0.708907	0.106738	0.7325	0.105423	0.737188	0.099591	0.744777
GSE81547	SCDC	sc-CMGAI	delta	800	-	-	0.089444	0.813142	0.092105	0.796711	0.0951	0.787507	0.092733	0.790829	0.087889	0.808641	0.093043	0.812748
GSE81547	SCDC	sc-CMGAI	ductal	800	-	-	0.115678	0.883428	0.115827	0.880758	0.10888	0.880605	0.111235	0.878425	0.110866	0.883824	0.118903	0.882809
GSE81547	SCDC	sc-CMGAI	acinar	900	-	-	0.118839	0.761089	0.117535	0.760605	0.117956	0.765723	0.117109	0.764578	0.11776	0.762896	0.11684	0.767951
GSE81547	SCDC	sc-CMGAI	alpha	900	-	-	0.108053	0.847383	0.104486	0.854524	0.103538	0.856988	0.100167	0.857548	0.109609	0.848895	0.107025	0.854671
GSE81547	SCDC	sc-CMGAI	beta	900	-	-	0.103354	0.741765	0.104463	0.735398	0.108443	0.707501	0.106519	0.732528	0.105397	0.738885	0.100303	0.741384
GSE81547	SCDC	sc-CMGAI	delta	900	-	-	0.08959	0.813457	0.092378	0.796796	0.094048	0.789579	0.092264	0.793794	0.087936	0.807368	0.093498	0.813075
GSE81547	SCDC	sc-CMGAI	ductal	900	-	-	0.114283	0.882476	0.11648	0.879985	0.108601	0.880417	0.111573	0.87897	0.112372	0.883401	0.115788	0.881815
GSE81547	SCDC	sc-CMGAI	acinar	1000	-	-	0.118612	0.761422	0.117587	0.761031	0.118188	0.764929	0.116864	0.765343	0.117996	0.763242	0.116879	0.768037
GSE81547	SCDC	sc-CMGAI	alpha	1000	-	-	0.10837	0.847322	0.105071	0.854169	0.103912	0.857155	0.100639	0.856227	0.108768	0.850405	0.107006	0.854869
GSE81547	SCDC	sc-CMGAI	beta	1000	-	-	0.102359	0.746417	0.105062	0.734022	0.10723	0.709455	0.10703	0.730583	0.105977	0.736462	0.100174	0.741707
GSE81547	SCDC	sc-CMGAI	delta	1000	-	-	0.08977	0.813784	0.091886	0.796732	0.09466	0.789696	0.092173	0.794118	0.088054	0.807371	0.093222	0.813049
GSE81547	SCDC	sc-CMGAI	ductal	1000	-	-	0.114895	0.882396	0.116414	0.88053	0.107873	0.879477	0.110504	0.878238	0.110043	0.882937	0.117163	0.882935
GSE81547	MuSiC	Control	acinar	0	0.111898	0.764171	-	-	-	-	-	-	-	-	-	-	-	-
GSE81547	MuSiC	Control	alpha	0	0.100566	0.873945	-	-	-	-	-	-	-	-	-	-	-	-
GSE81547	MuSiC	Control	beta	0	0.106864	0.782974	-	-	-	-	-	-	-	-	-	-	-	-
GSE81547	MuSiC	Control	delta	0	0.099142	0.804722	-	-	-	-	-	-	-	-	-	-	-	-
GSE81547	MuSiC	Control	ductal	0	0.211417	0.90641	-	-	-	-	-	-	-	-	-	-	-	-
GSE81547	MuSiC	sc-CMGAI	acinar	100	-	-	0.116815	0.734899	0.112805	0.752073	0.113211	0.75255	0.112543	0.753091	0.114193	0.747555	0.112997	0.75307
GSE81547	MuSiC	sc-CMGAI	alpha	100	-	-	0.09689	0.857236	0.095553	0.864662	0.096349	0.868743	0.09284	0.870389	0.097041	0.861924	0.101702	0.859634
GSE81547	MuSiC	sc-CMGAI	beta	100	-	-	0.08988	0.808162	0.089855	0.80988	0.089164	0.807356	0.092206	0.801561	0.091154	0.810076	0.086908	0.818349
GSE81547	MuSiC	sc-CMGAI	delta	100	-	-	0.086148	0.833049	0.086539	0.833919	0.087893	0.829521	0.089517	0.825752	0.08665	0.837002	0.086625	0.833723
GSE81547	MuSiC	sc-CMGAI	ductal	100	-	-	0.21929	0.900337	0.21543	0.900275	0.216699	0.902044	0.215254	0.901405	0.213432	0.901216	0.21392	0.903247

GSE81547	MuSiC	sc-CMGAI	acinar	200	-	-	0.115108	0.742763	0.113463	0.749665	0.113737	0.750553	0.112749	0.753556	0.114552	0.745582	0.113307	0.75287
GSE81547	MuSiC	sc-CMGAI	alpha	200	-	-	0.098659	0.862039	0.094167	0.867934	0.099016	0.867512	0.091967	0.872497	0.098043	0.862526	0.101967	0.856281
GSE81547	MuSiC	sc-CMGAI	beta	200	-	-	0.088747	0.810044	0.089642	0.808159	0.088672	0.80853	0.09258	0.800467	0.092351	0.806398	0.086988	0.819819
GSE81547	MuSiC	sc-CMGAI	delta	200	-	-	0.087195	0.831622	0.086735	0.833387	0.08706	0.833223	0.088813	0.829832	0.087724	0.835742	0.085983	0.83556
GSE81547	MuSiC	sc-CMGAI	ductal	200	-	-	0.216671	0.900085	0.215016	0.902066	0.21647	0.901434	0.216875	0.90148	0.214476	0.901018	0.215618	0.901257
GSE81547	MuSiC	sc-CMGAI	acinar	300	-	-	0.115218	0.743145	0.113687	0.748751	0.114042	0.749462	0.112299	0.754342	0.114015	0.748176	0.112843	0.754618
GSE81547	MuSiC	sc-CMGAI	alpha	300	-	-	0.097191	0.858843	0.095861	0.866627	0.098723	0.865319	0.091206	0.868716	0.096549	0.860144	0.10034	0.858775
GSE81547	MuSiC	sc-CMGAI	beta	300	-	-	0.089253	0.811282	0.090304	0.807264	0.089823	0.804183	0.091899	0.804349	0.092828	0.806506	0.087707	0.81794
GSE81547	MuSiC	sc-CMGAI	delta	300	-	-	0.08669	0.832262	0.086743	0.834527	0.086965	0.83424	0.088343	0.829609	0.087052	0.837313	0.086437	0.834451
GSE81547	MuSiC	sc-CMGAI	ductal	300	-	-	0.215669	0.901419	0.214603	0.902237	0.217583	0.901511	0.215817	0.90162	0.214946	0.901222	0.215238	0.900948
GSE81547	MuSiC	sc-CMGAI	acinar	400	-	-	0.115381	0.741879	0.113428	0.74969	0.114723	0.746094	0.112325	0.755118	0.114312	0.747141	0.112522	0.755925
GSE81547	MuSiC	sc-CMGAI	alpha	400	-	-	0.098489	0.860417	0.094924	0.867791	0.097215	0.865818	0.093981	0.866171	0.097355	0.858488	0.100625	0.859308
GSE81547	MuSiC	sc-CMGAI	beta	400	-	-	0.088716	0.811492	0.089946	0.806894	0.090081	0.804706	0.091249	0.806566	0.092183	0.807502	0.087422	0.81669
GSE81547	MuSiC	sc-CMGAI	delta	400	-	-	0.086876	0.831621	0.087067	0.832925	0.087409	0.832709	0.088715	0.830282	0.086855	0.837241	0.086535	0.833872
GSE81547	MuSiC	sc-CMGAI	ductal	400	-	-	0.216295	0.899932	0.215114	0.901895	0.216991	0.901841	0.216542	0.902044	0.215437	0.901451	0.215242	0.90094
GSE81547	MuSiC	sc-CMGAI	acinar	500	-	-	0.115243	0.742574	0.113267	0.750796	0.114252	0.74843	0.112669	0.753604	0.114514	0.745783	0.113025	0.753842
GSE81547	MuSiC	sc-CMGAI	alpha	500	-	-	0.095975	0.860346	0.097269	0.865716	0.097878	0.867188	0.093438	0.86794	0.096954	0.859897	0.101057	0.85971
GSE81547	MuSiC	sc-CMGAI	beta	500	-	-	0.089089	0.809903	0.088965	0.810992	0.089602	0.807162	0.091418	0.804591	0.091977	0.809061	0.087499	0.817172
GSE81547	MuSiC	sc-CMGAI	delta	500	-	-	0.085964	0.833847	0.086918	0.834176	0.087825	0.831376	0.088425	0.829513	0.087019	0.836067	0.086393	0.834538
GSE81547	MuSiC	sc-CMGAI	ductal	500	-	-	0.217014	0.900176	0.215284	0.901827	0.217139	0.901868	0.215778	0.90202	0.215507	0.901554	0.2155	0.901134
GSE81547	MuSiC	sc-CMGAI	acinar	600	-	-	0.115679	0.74027	0.113455	0.74975	0.114126	0.748653	0.112098	0.755264	0.114368	0.746396	0.112876	0.754547
GSE81547	MuSiC	sc-CMGAI	alpha	600	-	-	0.097315	0.860121	0.095404	0.86636	0.096365	0.867514	0.09315	0.867622	0.097273	0.858647	0.098212	0.860234
GSE81547	MuSiC	sc-CMGAI	beta	600	-	-	0.089304	0.809899	0.089941	0.808195	0.089775	0.805873	0.091227	0.80662	0.0924	0.808134	0.088079	0.815696
GSE81547	MuSiC	sc-CMGAI	delta	600	-	-	0.086678	0.832443	0.086947	0.83394	0.087391	0.832058	0.08862	0.8293	0.087001	0.836497	0.086148	0.83502
GSE81547	MuSiC	sc-CMGAI	ductal	600	-	-	0.217044	0.900333	0.215201	0.901755	0.217201	0.90196	0.216574	0.901459	0.214751	0.901648	0.215653	0.901402
GSE81547	MuSiC	sc-CMGAI	acinar	700	-	-	0.11484	0.744595	0.1136	0.749085	0.114188	0.748458	0.112685	0.75351	0.114003	0.747805	0.1129	0.754409
GSE81547	MuSiC	sc-CMGAI	alpha	700	-	-	0.097457	0.859057	0.096331	0.866956	0.096961	0.868679	0.092327	0.870514	0.098027	0.858602	0.100798	0.857949
GSE81547	MuSiC	sc-CMGAI	beta	700	-	-	0.088593	0.813566	0.089922	0.806899	0.088933	0.808487	0.091258	0.805486	0.091921	0.806959	0.087039	0.819351
GSE81547	MuSiC	sc-CMGAI	delta	700	-	-	0.087017	0.831644	0.086849	0.834392	0.087275	0.83183	0.088582	0.828871	0.0869	0.836659	0.086596	0.833399
GSE81547	MuSiC	sc-CMGAI	ductal	700	-	-	0.216349	0.900698	0.214973	0.902187	0.217291	0.901488	0.216167	0.901415	0.215529	0.901305	0.215578	0.900956
GSE81547	MuSiC	sc-CMGAI	acinar	800	-	-	0.115336	0.74208	0.11355	0.749322	0.114085	0.749099	0.112707	0.753761	0.114597	0.745824	0.112985	0.753822
GSE81547	MuSiC	sc-CMGAI	alpha	800	-	-	0.097522	0.85896	0.096817	0.866671	0.0967	0.867619	0.091938	0.869827	0.097759	0.858561	0.101015	0.8587
GSE81547	MuSiC	sc-CMGAI	beta	800	-	-	0.088826	0.811313	0.090148	0.806671	0.089591	0.805761	0.091263	0.806142	0.09217	0.807341	0.087073	0.819274
GSE81547	MuSiC	sc-CMGAI	delta	800	-	-	0.086767	0.832082	0.086762	0.834519	0.087503	0.831468	0.088906	0.828202	0.087187	0.836238	0.086548	0.833797
GSE81547	MuSiC	sc-CMGAI	ductal	800	-	-	0.217571	0.90043	0.214499	0.902011	0.217032	0.901486	0.216256	0.90137	0.215399	0.901345	0.216398	0.900213
GSE81547	MuSiC	sc-CMGAI	acinar	900	-	-	0.115516	0.741688	0.113378	0.749898	0.114396	0.74768	0.112699	0.753465	0.114089	0.747423	0.112862	0.754269

GSE81547	MuSiC	sc-CMGAI	alpha	900	-	-	0.097475	0.859298	0.096842	0.867066	0.09717	0.868062	0.09376	0.869675	0.09804	0.857711	0.0999	0.860994
GSE81547	MuSiC	sc-CMGAI	beta	900	-	-	0.08899	0.811289	0.089506	0.808805	0.089685	0.805688	0.091141	0.806121	0.092143	0.807316	0.087449	0.817985
GSE81547	MuSiC	sc-CMGAI	delta	900	-	-	0.0865	0.832715	0.086746	0.834611	0.087465	0.831872	0.088658	0.829253	0.086822	0.837325	0.086184	0.834925
GSE81547	MuSiC	sc-CMGAI	ductal	900	-	-	0.217216	0.899967	0.215181	0.9021	0.217387	0.9018	0.216293	0.901242	0.215535	0.901312	0.215142	0.900845
GSE81547	MuSiC	sc-CMGAI	acinar	1000	-	-	0.114957	0.743936	0.113667	0.748854	0.114191	0.74817	0.112499	0.754136	0.114247	0.747191	0.112799	0.754629
GSE81547	MuSiC	sc-CMGAI	alpha	1000	-	-	0.097996	0.859125	0.096554	0.86628	0.097246	0.868131	0.093093	0.868049	0.097959	0.859334	0.099318	0.860529
GSE81547	MuSiC	sc-CMGAI	beta	1000	-	-	0.088417	0.812017	0.089745	0.808727	0.089183	0.807798	0.091471	0.805523	0.092239	0.807226	0.087477	0.817444
GSE81547	MuSiC	sc-CMGAI	delta	1000	-	-	0.086532	0.832869	0.086811	0.834527	0.087565	0.831451	0.088589	0.829776	0.087192	0.836534	0.086382	0.834759
GSE81547	MuSiC	sc-CMGAI	ductal	1000	-	-	0.216188	0.900318	0.214727	0.901769	0.21758	0.901135	0.216495	0.9014	0.214508	0.90159	0.215245	0.900662
GSE81547	BisqueRN	Control	acinar	0	0.103173	0.815628	-	-	-	-	-	-	-	-	-	-	-	-
GSE81547	BisqueRN	Control	alpha	0	0.285919	0.883221	-	-	-	-	-	-	-	-	-	-	-	-
GSE81547	BisqueRN	Control	beta	0	0.153166	0.676321	-	-	-	-	-	-	-	-	-	-	-	-
GSE81547	BisqueRN	Control	delta	0	0.128316	0.726595	-	-	-	-	-	-	-	-	-	-	-	-
GSE81547	BisqueRN	Control	ductal	0	0.097416	0.758197	-	-	-	-	-	-	-	-	-	-	-	-
GSE81547	BisqueRN	sc-CMGAI	acinar	100	-	-	0.102744	0.826043	0.106662	0.810314	0.105873	0.81791	0.105632	0.81955	0.106236	0.815209	0.106121	0.812579
GSE81547	BisqueRN	sc-CMGAI	alpha	100	-	-	0.247318	0.882937	0.243846	0.881798	0.244527	0.885087	0.244008	0.881298	0.246071	0.883669	0.244732	0.881214
GSE81547	BisqueRN	sc-CMGAI	beta	100	-	-	0.150154	0.697352	0.144135	0.687488	0.146693	0.730972	0.146288	0.681755	0.148829	0.677718	0.142715	0.693568
GSE81547	BisqueRN	sc-CMGAI	delta	100	-	-	0.118093	0.738508	0.122403	0.594763	0.121181	0.656972	0.124376	0.587258	0.116591	0.703274	0.128724	0.505336
GSE81547	BisqueRN	sc-CMGAI	ductal	100	-	-	0.097472	0.772168	0.094197	0.767652	0.095836	0.765068	0.096165	0.757654	0.093542	0.781853	0.093294	0.776331
GSE81547	BisqueRN	sc-CMGAI	acinar	200	-	-	0.103061	0.82092	0.107024	0.807159	0.105607	0.816799	0.106745	0.806891	0.106196	0.814794	0.106195	0.81094
GSE81547	BisqueRN	sc-CMGAI	alpha	200	-	-	0.246626	0.882949	0.243012	0.879363	0.244184	0.885373	0.243409	0.876472	0.245499	0.883131	0.244137	0.878873
GSE81547	BisqueRN	sc-CMGAI	beta	200	-	-	0.149752	0.700195	0.146452	0.683313	0.147563	0.744492	0.147087	0.676698	0.150767	0.657402	0.143036	0.685168
GSE81547	BisqueRN	sc-CMGAI	delta	200	-	-	0.119027	0.703537	0.125224	0.568586	0.121765	0.658636	0.125887	0.566643	0.11499	0.723787	0.128131	0.506762
GSE81547	BisqueRN	sc-CMGAI	ductal	200	-	-	0.095859	0.789645	0.09495	0.774631	0.097038	0.767509	0.09597	0.770211	0.093428	0.792588	0.094431	0.777577
GSE81547	BisqueRN	sc-CMGAI	acinar	300	-	-	0.102343	0.826129	0.107084	0.802651	0.106116	0.805618	0.10705	0.807627	0.106378	0.812989	0.107383	0.801879
GSE81547	BisqueRN	sc-CMGAI	alpha	300	-	-	0.24702	0.883397	0.242273	0.877864	0.244102	0.886805	0.243302	0.875634	0.245985	0.882655	0.243433	0.875715
GSE81547	BisqueRN	sc-CMGAI	beta	300	-	-	0.151442	0.696368	0.147698	0.686975	0.148852	0.759518	0.147335	0.663865	0.151774	0.644953	0.140848	0.66929
GSE81547	BisqueRN	sc-CMGAI	delta	300	-	-	0.118391	0.748107	0.124674	0.582463	0.120567	0.699149	0.125933	0.557108	0.114336	0.72175	0.131694	0.47056
GSE81547	BisqueRN	sc-CMGAI	ductal	300	-	-	0.09864	0.779739	0.095224	0.779012	0.097557	0.769307	0.097282	0.774497	0.094015	0.800138	0.094908	0.779704
GSE81547	BisqueRN	sc-CMGAI	acinar	400	-	-	0.103482	0.822115	0.106887	0.803007	0.104354	0.809065	0.106614	0.808897	0.10679	0.810889	0.107604	0.802574
GSE81547	BisqueRN	sc-CMGAI	alpha	400	-	-	0.24741	0.883402	0.24189	0.876333	0.244152	0.888235	0.243202	0.875682	0.246486	0.882335	0.242605	0.87567
GSE81547	BisqueRN	sc-CMGAI	beta	400	-	-	0.151673	0.708743	0.148466	0.684972	0.149559	0.766739	0.148667	0.672272	0.152341	0.652232	0.142231	0.682309
GSE81547	BisqueRN	sc-CMGAI	delta	400	-	-	0.116052	0.762699	0.124322	0.586266	0.119965	0.723006	0.124941	0.572577	0.1123	0.743863	0.130494	0.480489
GSE81547	BisqueRN	sc-CMGAI	ductal	400	-	-	0.097806	0.786717	0.096631	0.775637	0.099564	0.754289	0.098013	0.775944	0.094013	0.803294	0.096387	0.773251
GSE81547	BisqueRN	sc-CMGAI	acinar	500	-	-	0.102343	0.824136	0.108334	0.795419	0.104959	0.807358	0.106876	0.803898	0.107147	0.807956	0.107208	0.801726
GSE81547	BisqueRN	sc-CMGAI	alpha	500	-	-	0.246908	0.883449	0.242287	0.875642	0.244153	0.886825	0.242993	0.872134	0.24631	0.882133	0.243166	0.875538

GSE81547	BisqueRN	sc-CMGAI	beta	500	-	-	0.151257	0.698358	0.14928	0.671864	0.149963	0.773505	0.14802	0.68118	0.151861	0.642389	0.141969	0.683492
GSE81547	BisqueRN	sc-CMGAI	delta	500	-	-	0.117565	0.749305	0.123763	0.590612	0.119244	0.725586	0.127738	0.528958	0.112382	0.726835	0.128815	0.497579
GSE81547	BisqueRN	sc-CMGAI	ductal	500	-	-	0.099214	0.779001	0.096587	0.781614	0.099339	0.758347	0.09881	0.774269	0.094589	0.802125	0.096144	0.780069
GSE81547	BisqueRN	sc-CMGAI	acinar	600	-	-	0.102493	0.822791	0.107511	0.799601	0.105142	0.799681	0.108469	0.791201	0.108068	0.808439	0.107269	0.795428
GSE81547	BisqueRN	sc-CMGAI	alpha	600	-	-	0.24732	0.883726	0.242474	0.877124	0.24389	0.886554	0.243295	0.871356	0.247667	0.881686	0.24201	0.869051
GSE81547	BisqueRN	sc-CMGAI	beta	600	-	-	0.152038	0.702516	0.150093	0.677474	0.149421	0.772628	0.148793	0.662254	0.154042	0.641037	0.141983	0.673636
GSE81547	BisqueRN	sc-CMGAI	delta	600	-	-	0.116387	0.768479	0.121998	0.62352	0.120943	0.688181	0.125669	0.559131	0.109726	0.776803	0.132011	0.466166
GSE81547	BisqueRN	sc-CMGAI	ductal	600	-	-	0.099681	0.781177	0.097441	0.77918	0.100286	0.753473	0.099003	0.775549	0.094664	0.802254	0.096883	0.778017
GSE81547	BisqueRN	sc-CMGAI	acinar	700	-	-	0.103157	0.823241	0.108363	0.795364	0.106591	0.787953	0.106942	0.799341	0.109114	0.800845	0.108229	0.791413
GSE81547	BisqueRN	sc-CMGAI	alpha	700	-	-	0.247454	0.882826	0.241878	0.874604	0.244476	0.885074	0.24329	0.865713	0.24738	0.882024	0.243	0.871864
GSE81547	BisqueRN	sc-CMGAI	beta	700	-	-	0.151383	0.706783	0.150506	0.662498	0.149144	0.774131	0.149025	0.670427	0.153548	0.643475	0.140891	0.674075
GSE81547	BisqueRN	sc-CMGAI	delta	700	-	-	0.115027	0.746402	0.122057	0.615574	0.122181	0.658186	0.12752	0.534931	0.109787	0.766291	0.13102	0.48062
GSE81547	BisqueRN	sc-CMGAI	ductal	700	-	-	0.098213	0.789986	0.097312	0.782357	0.099248	0.761911	0.099546	0.780535	0.094167	0.805567	0.097835	0.774724
GSE81547	BisqueRN	sc-CMGAI	acinar	800	-	-	0.102463	0.826634	0.108499	0.793476	0.106208	0.791725	0.10714	0.794241	0.108162	0.807733	0.108426	0.789425
GSE81547	BisqueRN	sc-CMGAI	alpha	800	-	-	0.247858	0.88184	0.242177	0.875162	0.244579	0.884837	0.243616	0.866212	0.247836	0.881421	0.243177	0.872287
GSE81547	BisqueRN	sc-CMGAI	beta	800	-	-	0.152594	0.698822	0.150656	0.666519	0.149634	0.777287	0.149983	0.661195	0.154874	0.633693	0.14128	0.67669
GSE81547	BisqueRN	sc-CMGAI	delta	800	-	-	0.114658	0.767313	0.121791	0.622182	0.12105	0.682408	0.126063	0.559984	0.108662	0.782476	0.127807	0.510238
GSE81547	BisqueRN	sc-CMGAI	ductal	800	-	-	0.100142	0.780422	0.097306	0.781871	0.099535	0.757495	0.100209	0.771502	0.09578	0.796546	0.097256	0.777593
GSE81547	BisqueRN	sc-CMGAI	acinar	900	-	-	0.103342	0.82577	0.108879	0.791745	0.10653	0.787165	0.108326	0.788054	0.108938	0.802972	0.108139	0.788818
GSE81547	BisqueRN	sc-CMGAI	alpha	900	-	-	0.247842	0.882184	0.241944	0.873538	0.244489	0.886026	0.243578	0.863806	0.247964	0.881639	0.242349	0.87104
GSE81547	BisqueRN	sc-CMGAI	beta	900	-	-	0.152053	0.716218	0.151142	0.662152	0.149895	0.774215	0.150545	0.64786	0.153665	0.644408	0.14094	0.67715
GSE81547	BisqueRN	sc-CMGAI	delta	900	-	-	0.114039	0.76558	0.121267	0.624285	0.120443	0.687937	0.124702	0.576815	0.109126	0.769893	0.128904	0.499722
GSE81547	BisqueRN	sc-CMGAI	ductal	900	-	-	0.099356	0.779781	0.097824	0.783063	0.099428	0.760638	0.099404	0.777414	0.095106	0.799942	0.09705	0.780708
GSE81547	BisqueRN	sc-CMGAI	acinar	1000	-	-	0.103508	0.823113	0.107948	0.796424	0.106121	0.792118	0.108385	0.79161	0.108828	0.803934	0.108376	0.786921
GSE81547	BisqueRN	sc-CMGAI	alpha	1000	-	-	0.247667	0.882512	0.24187	0.875409	0.244948	0.88473	0.243316	0.867281	0.247469	0.881907	0.242777	0.870571
GSE81547	BisqueRN	sc-CMGAI	beta	1000	-	-	0.15228	0.707778	0.151649	0.667244	0.150298	0.777542	0.150506	0.650529	0.155543	0.629757	0.141183	0.679883
GSE81547	BisqueRN	sc-CMGAI	delta	1000	-	-	0.113094	0.762269	0.12038	0.644569	0.119798	0.703593	0.124133	0.582139	0.109131	0.784229	0.127715	0.510878
GSE81547	BisqueRN	sc-CMGAI	ductal	1000	-	-	0.098028	0.791301	0.097656	0.783745	0.099369	0.75712	0.100778	0.769499	0.095287	0.797671	0.097774	0.779253
HCLKidne	SCDC	Control	B_cell(Pla	0	0.028896	0.964295	-	-	-	-	-	-	-	-	-	-	-	-
HCLKidne	SCDC	Control	Dendritic_	0	0.050658	0.935672	-	-	-	-	-	-	-	-	-	-	-	-
HCLKidne	SCDC	Control	Distal_tub	0	0.093324	0.80107	-	-	-	-	-	-	-	-	-	-	-	-
HCLKidne	SCDC	Control	Distal_tub	0	0.044711	0.911192	-	-	-	-	-	-	-	-	-	-	-	-
HCLKidne	SCDC	Control	Endothelia	0	0.05591	0.905822	-	-	-	-	-	-	-	-	-	-	-	-
HCLKidne	SCDC	Control	Endothelia	0	0.042988	0.936861	-	-	-	-	-	-	-	-	-	-	-	-
HCLKidne	SCDC	Control	Intercalate	0	0.046205	0.908246	-	-	-	-	-	-	-	-	-	-	-	-
HCLKidne	SCDC	Control	Intercalate	0	0.058234	0.901662	-	-	-	-	-	-	-	-	-	-	-	-

HCLKidne SCDC	Control	Loop_of_H	0	0.064238	0.934485	-	-	-	-	-	-	-	-	-	-	-	-
HCLKidne SCDC	Control	Loop_of_h	0	0.067651	0.722658	-	-	-	-	-	-	-	-	-	-	-	-
HCLKidne SCDC	Control	Loop_of_h	0	0.083787	0.75624	-	-	-	-	-	-	-	-	-	-	-	-
HCLKidne SCDC	Control	Principle_	0	0.065115	0.856998	-	-	-	-	-	-	-	-	-	-	-	-
HCLKidne SCDC	Control	Proximal_	0	0.055213	0.926555	-	-	-	-	-	-	-	-	-	-	-	-
HCLKidne SCDC	Control	Proximal_	0	0.090704	0.758707	-	-	-	-	-	-	-	-	-	-	-	-
HCLKidne SCDC	Control	Proximal_	0	0.054378	0.878859	-	-	-	-	-	-	-	-	-	-	-	-
HCLKidne SCDC	Control	Smooth_m	0	0.039212	0.94049	-	-	-	-	-	-	-	-	-	-	-	-
HCLKidne SCDC	sc-CMGAI	B_cell(Pla	100	-	-	0.025663	0.967858	0.02688	0.968023	0.02682	0.96738	0.025628	0.968939	0.02596	0.967669	0.026543	0.969027
HCLKidne SCDC	sc-CMGAI	Dendritic_	100	-	-	0.033367	0.946508	0.03473	0.94264	0.033784	0.946195	0.033114	0.948612	0.035404	0.942343	0.032869	0.948983
HCLKidne SCDC	sc-CMGAI	Distal_tub	100	-	-	0.062808	0.800864	0.073937	0.768287	0.07531	0.736273	0.063078	0.801618	0.068098	0.764164	0.067177	0.767065
HCLKidne SCDC	sc-CMGAI	Distal_tub	100	-	-	0.051788	0.881007	0.055187	0.872373	0.057009	0.856732	0.053017	0.880455	0.056151	0.863885	0.054349	0.871667
HCLKidne SCDC	sc-CMGAI	Endothelia	100	-	-	0.055511	0.8764	0.05821	0.871081	0.056842	0.878141	0.054897	0.883033	0.05478	0.880864	0.055358	0.879679
HCLKidne SCDC	sc-CMGAI	Endothelia	100	-	-	0.056887	0.919568	0.056263	0.91527	0.056791	0.914366	0.057691	0.908039	0.055985	0.918963	0.055889	0.920177
HCLKidne SCDC	sc-CMGAI	Intercalate	100	-	-	0.045755	0.925496	0.048206	0.922695	0.047815	0.923751	0.050978	0.924363	0.050095	0.924609	0.047767	0.922936
HCLKidne SCDC	sc-CMGAI	Intercalate	100	-	-	0.057647	0.900986	0.060157	0.909465	0.060974	0.904536	0.063265	0.912155	0.059928	0.911254	0.062626	0.878476
HCLKidne SCDC	sc-CMGAI	Loop_of_H	100	-	-	0.041333	0.953893	0.044362	0.917716	0.042493	0.924364	0.039486	0.937951	0.04433	0.932025	0.038747	0.949962
HCLKidne SCDC	sc-CMGAI	Loop_of_h	100	-	-	0.066298	0.707608	0.061243	0.731685	0.061666	0.727417	0.063142	0.714111	0.062153	0.725985	0.064357	0.717632
HCLKidne SCDC	sc-CMGAI	Loop_of_h	100	-	-	0.058158	0.836125	0.060097	0.835094	0.059994	0.833934	0.059782	0.833163	0.058535	0.839674	0.058357	0.838848
HCLKidne SCDC	sc-CMGAI	Principle_	100	-	-	0.06306	0.818262	0.061789	0.820528	0.065534	0.781243	0.066511	0.787983	0.06167	0.819238	0.063719	0.81892
HCLKidne SCDC	sc-CMGAI	Proximal_	100	-	-	0.03862	0.930498	0.0395	0.931221	0.038959	0.932091	0.038171	0.931434	0.038529	0.932557	0.038391	0.932284
HCLKidne SCDC	sc-CMGAI	Proximal_	100	-	-	0.08968	0.651599	0.090168	0.629857	0.091156	0.653395	0.092285	0.619536	0.092967	0.594922	0.089965	0.64189
HCLKidne SCDC	sc-CMGAI	Proximal_	100	-	-	0.066254	0.864181	0.071847	0.848361	0.072045	0.838259	0.069548	0.840469	0.071482	0.83881	0.069283	0.844941
HCLKidne SCDC	sc-CMGAI	Smooth_m	100	-	-	0.045104	0.951029	0.044671	0.954913	0.044071	0.954741	0.045554	0.950937	0.046279	0.950781	0.044269	0.947317
HCLKidne SCDC	sc-CMGAI	B_cell(Pla	200	-	-	0.026779	0.966709	0.028105	0.966978	0.026041	0.96789	0.025957	0.967031	0.025574	0.968691	0.025333	0.968791
HCLKidne SCDC	sc-CMGAI	Dendritic_	200	-	-	0.032703	0.947833	0.034718	0.94264	0.033753	0.946424	0.032824	0.949661	0.035308	0.943234	0.032538	0.950581
HCLKidne SCDC	sc-CMGAI	Distal_tub	200	-	-	0.063607	0.807751	0.071747	0.781102	0.073585	0.752594	0.060477	0.820591	0.068589	0.766627	0.067195	0.783407
HCLKidne SCDC	sc-CMGAI	Distal_tub	200	-	-	0.050293	0.886601	0.055446	0.870171	0.052709	0.875123	0.051204	0.894673	0.055508	0.868133	0.053172	0.873756
HCLKidne SCDC	sc-CMGAI	Endothelia	200	-	-	0.055584	0.876833	0.059819	0.864674	0.056142	0.880261	0.054093	0.885592	0.054944	0.879303	0.055534	0.877536
HCLKidne SCDC	sc-CMGAI	Endothelia	200	-	-	0.056742	0.914563	0.0584	0.918591	0.057038	0.917237	0.058029	0.910678	0.05685	0.921453	0.056499	0.921896
HCLKidne SCDC	sc-CMGAI	Intercalate	200	-	-	0.04684	0.921236	0.048011	0.920365	0.043913	0.927623	0.049909	0.923956	0.048149	0.925321	0.047282	0.922961
HCLKidne SCDC	sc-CMGAI	Intercalate	200	-	-	0.057997	0.894938	0.060177	0.90521	0.058569	0.911711	0.063623	0.908007	0.059289	0.913542	0.061239	0.883134
HCLKidne SCDC	sc-CMGAI	Loop_of_H	200	-	-	0.039637	0.954853	0.044558	0.91479	0.038112	0.937614	0.037263	0.940961	0.042432	0.93891	0.039549	0.95424
HCLKidne SCDC	sc-CMGAI	Loop_of_h	200	-	-	0.067175	0.701562	0.061279	0.72949	0.061192	0.731503	0.062949	0.717531	0.060933	0.737453	0.062573	0.727161
HCLKidne SCDC	sc-CMGAI	Loop_of_h	200	-	-	0.058356	0.830716	0.060319	0.831564	0.061067	0.831566	0.059809	0.831075	0.058217	0.842401	0.058427	0.836977
HCLKidne SCDC	sc-CMGAI	Principle_	200	-	-	0.061657	0.831564	0.061611	0.819276	0.064568	0.784854	0.06381	0.819487	0.0604	0.825553	0.064048	0.807209

HCLKidne	SCDC	sc-CMGAI	Proximal_	200	-	-	0.039532	0.931525	0.039458	0.930738	0.03941	0.932161	0.037727	0.93259	0.038901	0.93121	0.038627	0.931911
HCLKidne	SCDC	sc-CMGAI	Proximal_	200	-	-	0.091097	0.632955	0.091317	0.613957	0.09183	0.6659	0.089923	0.633047	0.090816	0.619225	0.089611	0.641093
HCLKidne	SCDC	sc-CMGAI	Proximal_	200	-	-	0.067185	0.858192	0.070144	0.847642	0.070695	0.852471	0.071084	0.840862	0.069622	0.850949	0.069654	0.848815
HCLKidne	SCDC	sc-CMGAI	Smooth_m	200	-	-	0.045304	0.952258	0.043861	0.952403	0.043806	0.953905	0.04599	0.950817	0.044878	0.951644	0.043748	0.947933
HCLKidne	SCDC	sc-CMGAI	B_cell(Pla	300	-	-	0.027017	0.967333	0.027189	0.968124	0.026257	0.968225	0.026049	0.966565	0.025848	0.968075	0.026073	0.968691
HCLKidne	SCDC	sc-CMGAI	Dendritic_	300	-	-	0.03279	0.947953	0.034539	0.943662	0.033803	0.946628	0.032855	0.949727	0.03602	0.943893	0.032319	0.950048
HCLKidne	SCDC	sc-CMGAI	Distal_tub	300	-	-	0.064833	0.810175	0.073012	0.775831	0.073551	0.753727	0.061749	0.80519	0.067672	0.768531	0.066346	0.779625
HCLKidne	SCDC	sc-CMGAI	Distal_tub	300	-	-	0.0511	0.883375	0.055272	0.870525	0.053557	0.872174	0.049924	0.898009	0.055659	0.866645	0.052422	0.877509
HCLKidne	SCDC	sc-CMGAI	Endothelia	300	-	-	0.056188	0.874669	0.05817	0.871333	0.056798	0.877442	0.052905	0.890864	0.053636	0.885359	0.054438	0.882712
HCLKidne	SCDC	sc-CMGAI	Endothelia	300	-	-	0.057529	0.914128	0.055832	0.919677	0.057867	0.918717	0.055532	0.918554	0.055591	0.92481	0.05518	0.92306
HCLKidne	SCDC	sc-CMGAI	Intercalate	300	-	-	0.046562	0.921731	0.047358	0.921991	0.046667	0.926368	0.049871	0.925019	0.04905	0.926251	0.048407	0.921456
HCLKidne	SCDC	sc-CMGAI	Intercalate	300	-	-	0.058691	0.891661	0.059465	0.908656	0.059166	0.91753	0.063205	0.913417	0.059601	0.910814	0.061537	0.8876
HCLKidne	SCDC	sc-CMGAI	Loop_of_H	300	-	-	0.040167	0.953872	0.044884	0.91504	0.039677	0.932416	0.036716	0.943035	0.042342	0.938083	0.037871	0.952779
HCLKidne	SCDC	sc-CMGAI	Loop_of_h	300	-	-	0.066523	0.705363	0.061278	0.729984	0.061273	0.730937	0.062539	0.72145	0.061461	0.731875	0.062507	0.728569
HCLKidne	SCDC	sc-CMGAI	Loop_of_h	300	-	-	0.058494	0.831117	0.059133	0.836347	0.060415	0.832527	0.059332	0.83436	0.058391	0.840783	0.058171	0.840069
HCLKidne	SCDC	sc-CMGAI	Principle_	300	-	-	0.062734	0.814043	0.06274	0.80908	0.066057	0.777655	0.064858	0.809359	0.060065	0.82639	0.06293	0.818696
HCLKidne	SCDC	sc-CMGAI	Proximal_	300	-	-	0.039454	0.931387	0.0389	0.932658	0.039503	0.931592	0.038643	0.931614	0.038593	0.931561	0.038103	0.932414
HCLKidne	SCDC	sc-CMGAI	Proximal_	300	-	-	0.089841	0.642132	0.090403	0.624763	0.091116	0.665402	0.089273	0.652833	0.091963	0.602562	0.089256	0.645157
HCLKidne	SCDC	sc-CMGAI	Proximal_	300	-	-	0.067226	0.856548	0.070896	0.845811	0.071254	0.849009	0.069312	0.852227	0.071956	0.841486	0.069094	0.854775
HCLKidne	SCDC	sc-CMGAI	Smooth_m	300	-	-	0.044216	0.952927	0.044079	0.953483	0.044333	0.954298	0.044639	0.952195	0.045097	0.952972	0.043287	0.947998
HCLKidne	SCDC	sc-CMGAI	B_cell(Pla	400	-	-	0.026424	0.967304	0.027717	0.967099	0.026549	0.968936	0.026001	0.966947	0.025977	0.967668	0.02586	0.969009
HCLKidne	SCDC	sc-CMGAI	Dendritic_	400	-	-	0.032894	0.947158	0.034315	0.943621	0.033589	0.947205	0.032812	0.949012	0.036621	0.942083	0.032418	0.94929
HCLKidne	SCDC	sc-CMGAI	Distal_tub	400	-	-	0.06345	0.818766	0.072899	0.776947	0.073926	0.762301	0.061204	0.806852	0.068777	0.770029	0.066615	0.780457
HCLKidne	SCDC	sc-CMGAI	Distal_tub	400	-	-	0.050843	0.883477	0.05489	0.872913	0.053242	0.872854	0.049687	0.897794	0.05534	0.871208	0.052408	0.876845
HCLKidne	SCDC	sc-CMGAI	Endothelia	400	-	-	0.054884	0.8823	0.058091	0.873848	0.055892	0.882703	0.052766	0.89227	0.053989	0.886447	0.053845	0.885734
HCLKidne	SCDC	sc-CMGAI	Endothelia	400	-	-	0.055525	0.91755	0.055342	0.921843	0.056272	0.919556	0.055188	0.917984	0.055229	0.920127	0.054892	0.923643
HCLKidne	SCDC	sc-CMGAI	Intercalate	400	-	-	0.046007	0.925481	0.048037	0.920939	0.045635	0.925844	0.049415	0.923968	0.05018	0.925899	0.048685	0.922105
HCLKidne	SCDC	sc-CMGAI	Intercalate	400	-	-	0.057948	0.895063	0.059212	0.912624	0.058944	0.912238	0.062735	0.907425	0.05973	0.910748	0.061834	0.889459
HCLKidne	SCDC	sc-CMGAI	Loop_of_H	400	-	-	0.039118	0.955188	0.044523	0.917446	0.039502	0.933712	0.037174	0.942699	0.042523	0.935604	0.037624	0.954273
HCLKidne	SCDC	sc-CMGAI	Loop_of_h	400	-	-	0.067809	0.700527	0.061474	0.7275	0.060978	0.734981	0.06324	0.721286	0.060778	0.736503	0.062287	0.729437
HCLKidne	SCDC	sc-CMGAI	Loop_of_h	400	-	-	0.058216	0.832961	0.05933	0.833382	0.060238	0.835034	0.058878	0.835336	0.058528	0.840411	0.058122	0.837616
HCLKidne	SCDC	sc-CMGAI	Principle_	400	-	-	0.062042	0.822501	0.061167	0.820541	0.064172	0.791943	0.065186	0.805593	0.060007	0.829421	0.062041	0.822829
HCLKidne	SCDC	sc-CMGAI	Proximal_	400	-	-	0.039591	0.93035	0.038836	0.933147	0.039417	0.931808	0.038152	0.931383	0.039257	0.931345	0.038101	0.933039
HCLKidne	SCDC	sc-CMGAI	Proximal_	400	-	-	0.089212	0.663737	0.089877	0.630692	0.090487	0.673772	0.090327	0.629282	0.092002	0.60509	0.08919	0.646201
HCLKidne	SCDC	sc-CMGAI	Proximal_	400	-	-	0.065375	0.866694	0.069923	0.848041	0.070994	0.850272	0.070815	0.838001	0.071871	0.841001	0.069446	0.854391
HCLKidne	SCDC	sc-CMGAI	Smooth_m	400	-	-	0.044141	0.952991	0.043631	0.954616	0.043608	0.954268	0.04482	0.952969	0.044985	0.952834	0.043805	0.948972

HCLKidne	SCDC	sc-CMGAI	B_cell(Pla	500	-	-	0.026642	0.967264	0.027989	0.967919	0.026396	0.9682	0.025778	0.967865	0.025372	0.968543	0.026259	0.969114
HCLKidne	SCDC	sc-CMGAI	Dendritic_	500	-	-	0.032845	0.947147	0.034667	0.943209	0.033014	0.947582	0.032693	0.949593	0.035725	0.944379	0.032192	0.950138
HCLKidne	SCDC	sc-CMGAI	Distal_tub	500	-	-	0.064201	0.811449	0.072955	0.781042	0.074758	0.751269	0.061458	0.809138	0.067232	0.776331	0.065661	0.783549
HCLKidne	SCDC	sc-CMGAI	Distal_tub	500	-	-	0.051325	0.880786	0.054879	0.872767	0.053152	0.875816	0.050277	0.897853	0.054436	0.873156	0.052205	0.878084
HCLKidne	SCDC	sc-CMGAI	Endothelia	500	-	-	0.055239	0.880836	0.058213	0.871997	0.056291	0.879857	0.053007	0.89114	0.054277	0.884392	0.054563	0.882926
HCLKidne	SCDC	sc-CMGAI	Endothelia	500	-	-	0.0562	0.916557	0.056217	0.9218	0.056813	0.91947	0.056062	0.91513	0.056063	0.919336	0.054519	0.92147
HCLKidne	SCDC	sc-CMGAI	Intercalate	500	-	-	0.04628	0.924579	0.047926	0.92106	0.046312	0.926412	0.050124	0.923241	0.050026	0.924074	0.048789	0.919972
HCLKidne	SCDC	sc-CMGAI	Intercalate	500	-	-	0.058048	0.893848	0.060181	0.909682	0.059168	0.913974	0.063092	0.910403	0.061008	0.914223	0.06119	0.88826
HCLKidne	SCDC	sc-CMGAI	Loop_of_H	500	-	-	0.03892	0.955691	0.04504	0.9165	0.038856	0.936302	0.037345	0.943269	0.041756	0.938449	0.03741	0.952673
HCLKidne	SCDC	sc-CMGAI	Loop_of_h	500	-	-	0.06804	0.699613	0.061193	0.730783	0.061032	0.732802	0.062078	0.723793	0.060562	0.741405	0.062195	0.730404
HCLKidne	SCDC	sc-CMGAI	Loop_of_h	500	-	-	0.05835	0.831236	0.059481	0.833658	0.060321	0.832619	0.05896	0.835777	0.058295	0.840925	0.058179	0.838321
HCLKidne	SCDC	sc-CMGAI	Principle_	500	-	-	0.060971	0.828972	0.061403	0.818133	0.0646	0.789649	0.064745	0.799989	0.059478	0.831132	0.062794	0.824235
HCLKidne	SCDC	sc-CMGAI	Proximal_	500	-	-	0.040062	0.930605	0.038888	0.933129	0.038939	0.932271	0.038603	0.930809	0.039609	0.930941	0.038822	0.931551
HCLKidne	SCDC	sc-CMGAI	Proximal_	500	-	-	0.090152	0.64875	0.09024	0.627419	0.090646	0.672301	0.089525	0.643473	0.091085	0.61738	0.08898	0.644779
HCLKidne	SCDC	sc-CMGAI	Proximal_	500	-	-	0.066244	0.859901	0.070211	0.848029	0.071441	0.851971	0.070884	0.84402	0.071326	0.847651	0.070202	0.851177
HCLKidne	SCDC	sc-CMGAI	Smooth_m	500	-	-	0.044182	0.95284	0.043106	0.954684	0.043501	0.954964	0.044721	0.952895	0.045114	0.952897	0.043312	0.949442
HCLKidne	SCDC	sc-CMGAI	B_cell(Pla	600	-	-	0.026392	0.967761	0.027117	0.96751	0.026079	0.968235	0.025531	0.968119	0.026018	0.967361	0.025822	0.969406
HCLKidne	SCDC	sc-CMGAI	Dendritic_	600	-	-	0.032594	0.948099	0.034924	0.942721	0.033346	0.947762	0.032955	0.949059	0.035632	0.943203	0.032385	0.94969
HCLKidne	SCDC	sc-CMGAI	Distal_tub	600	-	-	0.064118	0.805879	0.072712	0.785859	0.074228	0.751794	0.060899	0.812196	0.068413	0.768813	0.067029	0.777084
HCLKidne	SCDC	sc-CMGAI	Distal_tub	600	-	-	0.05	0.888039	0.053976	0.876585	0.0531	0.872504	0.049984	0.895737	0.054631	0.873755	0.052582	0.875635
HCLKidne	SCDC	sc-CMGAI	Endothelia	600	-	-	0.055362	0.879748	0.05768	0.873504	0.054998	0.885473	0.053223	0.890098	0.054665	0.881434	0.054179	0.884648
HCLKidne	SCDC	sc-CMGAI	Endothelia	600	-	-	0.055905	0.914366	0.055692	0.920105	0.055188	0.918327	0.055903	0.915779	0.055823	0.920584	0.054827	0.921422
HCLKidne	SCDC	sc-CMGAI	Intercalate	600	-	-	0.047329	0.922874	0.047369	0.9205	0.045002	0.927461	0.049261	0.924571	0.049654	0.924636	0.048569	0.920442
HCLKidne	SCDC	sc-CMGAI	Intercalate	600	-	-	0.057853	0.896999	0.059767	0.908911	0.058763	0.91362	0.062859	0.910653	0.05931	0.91202	0.061042	0.887919
HCLKidne	SCDC	sc-CMGAI	Loop_of_H	600	-	-	0.039563	0.955018	0.043733	0.919975	0.038464	0.936462	0.037353	0.942043	0.041398	0.940853	0.037862	0.951654
HCLKidne	SCDC	sc-CMGAI	Loop_of_h	600	-	-	0.067655	0.703416	0.061133	0.730021	0.06111	0.73264	0.062733	0.721994	0.060853	0.734808	0.06256	0.724506
HCLKidne	SCDC	sc-CMGAI	Loop_of_h	600	-	-	0.058035	0.833693	0.059706	0.833638	0.060245	0.833187	0.059277	0.833418	0.058862	0.838293	0.058098	0.839757
HCLKidne	SCDC	sc-CMGAI	Principle_	600	-	-	0.061715	0.823035	0.061192	0.821287	0.065114	0.780604	0.065714	0.802344	0.059658	0.830968	0.061901	0.824011
HCLKidne	SCDC	sc-CMGAI	Proximal_	600	-	-	0.040056	0.929951	0.038762	0.933351	0.039682	0.932299	0.038391	0.931429	0.039572	0.931302	0.038234	0.933221
HCLKidne	SCDC	sc-CMGAI	Proximal_	600	-	-	0.090287	0.654477	0.090366	0.622063	0.090527	0.679291	0.089494	0.643856	0.090688	0.623941	0.088834	0.649105
HCLKidne	SCDC	sc-CMGAI	Proximal_	600	-	-	0.066162	0.861945	0.070626	0.845997	0.071524	0.852397	0.070573	0.842602	0.070171	0.852624	0.069622	0.852735
HCLKidne	SCDC	sc-CMGAI	Smooth_m	600	-	-	0.044013	0.952084	0.043336	0.953985	0.043632	0.955088	0.044266	0.952298	0.044971	0.952944	0.043978	0.949171
HCLKidne	SCDC	sc-CMGAI	B_cell(Pla	700	-	-	0.026414	0.9672	0.027488	0.967929	0.026777	0.969012	0.025605	0.967802	0.02559	0.968368	0.026202	0.968868
HCLKidne	SCDC	sc-CMGAI	Dendritic_	700	-	-	0.032864	0.947285	0.034962	0.942057	0.033729	0.947289	0.032733	0.949249	0.036258	0.942734	0.032059	0.950217
HCLKidne	SCDC	sc-CMGAI	Distal_tub	700	-	-	0.064405	0.809913	0.072457	0.782666	0.074283	0.756017	0.061527	0.808034	0.068718	0.768796	0.066202	0.783994
HCLKidne	SCDC	sc-CMGAI	Distal_tub	700	-	-	0.050448	0.885185	0.055622	0.867551	0.053104	0.875186	0.049808	0.897271	0.054691	0.871916	0.051844	0.880353

HCLKidne	SCDC	sc-CMGAI	Endothelia	700	-	-	0.055972	0.875571	0.058652	0.869118	0.056134	0.881706	0.052479	0.893075	0.054296	0.883158	0.054138	0.884662
HCLKidne	SCDC	sc-CMGAI	Endothelia	700	-	-	0.057505	0.918452	0.057148	0.920272	0.056404	0.919274	0.055137	0.919715	0.055663	0.923823	0.054173	0.921422
HCLKidne	SCDC	sc-CMGAI	Intercalate	700	-	-	0.046051	0.924066	0.047787	0.920635	0.045539	0.927794	0.049438	0.92379	0.049193	0.923618	0.047391	0.921704
HCLKidne	SCDC	sc-CMGAI	Intercalate	700	-	-	0.05798	0.89292	0.059424	0.911351	0.058336	0.913146	0.063306	0.908719	0.059632	0.91118	0.061524	0.881515
HCLKidne	SCDC	sc-CMGAI	Loop_of_H	700	-	-	0.040575	0.953998	0.045667	0.913308	0.038705	0.936314	0.03657	0.943918	0.041687	0.938984	0.037492	0.953849
HCLKidne	SCDC	sc-CMGAI	Loop_of_h	700	-	-	0.067611	0.702055	0.061299	0.729904	0.060927	0.73445	0.062842	0.723218	0.061194	0.735819	0.063008	0.723885
HCLKidne	SCDC	sc-CMGAI	Loop_of_h	700	-	-	0.057999	0.833398	0.059427	0.83431	0.060182	0.834574	0.058835	0.835338	0.058084	0.840638	0.058046	0.838013
HCLKidne	SCDC	sc-CMGAI	Principle_	700	-	-	0.062008	0.821487	0.061338	0.821203	0.064463	0.789028	0.06606	0.797596	0.059501	0.832614	0.062963	0.820732
HCLKidne	SCDC	sc-CMGAI	Proximal_	700	-	-	0.040126	0.929796	0.038532	0.933448	0.039303	0.932538	0.038825	0.930528	0.038519	0.932709	0.038382	0.932554
HCLKidne	SCDC	sc-CMGAI	Proximal_	700	-	-	0.089926	0.655599	0.090103	0.62653	0.090769	0.668356	0.089256	0.645397	0.091531	0.610872	0.089156	0.643808
HCLKidne	SCDC	sc-CMGAI	Proximal_	700	-	-	0.066422	0.861821	0.070607	0.846437	0.070595	0.850228	0.070491	0.845417	0.071253	0.842367	0.069145	0.851923
HCLKidne	SCDC	sc-CMGAI	Smooth_m	700	-	-	0.043965	0.953476	0.043239	0.954729	0.043391	0.955534	0.044578	0.95189	0.045295	0.953112	0.043583	0.948774
HCLKidne	SCDC	sc-CMGAI	B_cell(Pla	800	-	-	0.0265	0.967317	0.027274	0.967837	0.026325	0.968243	0.025673	0.967662	0.025812	0.967137	0.026131	0.969007
HCLKidne	SCDC	sc-CMGAI	Dendritic_	800	-	-	0.032866	0.947274	0.034666	0.943135	0.03344	0.948436	0.032889	0.948983	0.036611	0.942406	0.032101	0.950211
HCLKidne	SCDC	sc-CMGAI	Distal_tub	800	-	-	0.063704	0.818635	0.072914	0.781473	0.073932	0.746899	0.061465	0.809839	0.067885	0.768957	0.06646	0.778694
HCLKidne	SCDC	sc-CMGAI	Distal_tub	800	-	-	0.050456	0.885058	0.054365	0.874468	0.053582	0.873432	0.049756	0.896332	0.054768	0.871398	0.051467	0.882093
HCLKidne	SCDC	sc-CMGAI	Endothelia	800	-	-	0.055004	0.880837	0.057677	0.873242	0.056177	0.879739	0.05276	0.890713	0.05455	0.880178	0.053534	0.886812
HCLKidne	SCDC	sc-CMGAI	Endothelia	800	-	-	0.055664	0.916714	0.056197	0.919593	0.057025	0.919239	0.056155	0.918508	0.056627	0.924097	0.054125	0.922195
HCLKidne	SCDC	sc-CMGAI	Intercalate	800	-	-	0.046512	0.922792	0.047871	0.919978	0.04559	0.926919	0.049766	0.924121	0.049681	0.923976	0.047807	0.921592
HCLKidne	SCDC	sc-CMGAI	Intercalate	800	-	-	0.057738	0.897087	0.059871	0.909839	0.058809	0.91386	0.062861	0.910177	0.059346	0.914355	0.061376	0.885579
HCLKidne	SCDC	sc-CMGAI	Loop_of_H	800	-	-	0.03995	0.954233	0.044061	0.918621	0.039471	0.933495	0.03681	0.94354	0.04211	0.937942	0.037208	0.953827
HCLKidne	SCDC	sc-CMGAI	Loop_of_h	800	-	-	0.066957	0.703488	0.061535	0.727642	0.061	0.733706	0.063107	0.722077	0.061003	0.735912	0.062443	0.727954
HCLKidne	SCDC	sc-CMGAI	Loop_of_h	800	-	-	0.057915	0.834672	0.059397	0.83329	0.060055	0.833501	0.05841	0.838062	0.058336	0.840081	0.058088	0.839115
HCLKidne	SCDC	sc-CMGAI	Principle_	800	-	-	0.061373	0.826207	0.06146	0.820088	0.064791	0.784232	0.06593	0.798583	0.059061	0.836215	0.062817	0.817005
HCLKidne	SCDC	sc-CMGAI	Proximal_	800	-	-	0.040219	0.930255	0.038873	0.933146	0.039137	0.932324	0.039029	0.930324	0.03934	0.93134	0.038388	0.932489
HCLKidne	SCDC	sc-CMGAI	Proximal_	800	-	-	0.089648	0.658086	0.090694	0.619576	0.090769	0.672139	0.089466	0.644187	0.091336	0.615254	0.088981	0.643509
HCLKidne	SCDC	sc-CMGAI	Proximal_	800	-	-	0.066521	0.8625	0.070512	0.845173	0.070705	0.8513	0.071258	0.841048	0.071638	0.845531	0.069497	0.851468
HCLKidne	SCDC	sc-CMGAI	Smooth_m	800	-	-	0.043729	0.953176	0.043001	0.953908	0.043245	0.954637	0.04453	0.951975	0.044999	0.952907	0.043541	0.949951
HCLKidne	SCDC	sc-CMGAI	B_cell(Pla	900	-	-	0.026436	0.967607	0.027154	0.967859	0.026217	0.96868	0.025879	0.967519	0.025902	0.968201	0.025879	0.969155
HCLKidne	SCDC	sc-CMGAI	Dendritic_	900	-	-	0.032779	0.947652	0.034616	0.943051	0.033041	0.948581	0.033013	0.949343	0.036327	0.94382	0.032525	0.949073
HCLKidne	SCDC	sc-CMGAI	Distal_tub	900	-	-	0.063925	0.814838	0.07269	0.78245	0.074284	0.752076	0.061331	0.811156	0.06814	0.767708	0.065729	0.784199
HCLKidne	SCDC	sc-CMGAI	Distal_tub	900	-	-	0.050829	0.884254	0.055421	0.868182	0.053427	0.873396	0.049246	0.89695	0.055264	0.868809	0.051763	0.881
HCLKidne	SCDC	sc-CMGAI	Endothelia	900	-	-	0.055038	0.879227	0.058109	0.871006	0.056573	0.879619	0.052124	0.893764	0.054394	0.881219	0.053692	0.886165
HCLKidne	SCDC	sc-CMGAI	Endothelia	900	-	-	0.05648	0.913442	0.056419	0.921406	0.057097	0.917822	0.055179	0.918933	0.0566	0.922423	0.054271	0.920995
HCLKidne	SCDC	sc-CMGAI	Intercalate	900	-	-	0.046242	0.924134	0.048287	0.919478	0.044921	0.928146	0.049899	0.923847	0.049942	0.924124	0.047776	0.92218
HCLKidne	SCDC	sc-CMGAI	Intercalate	900	-	-	0.057716	0.897169	0.059457	0.909848	0.058568	0.912097	0.063267	0.912414	0.059726	0.909835	0.0609	0.886942

HCLKidne	SCDC	sc-CMGAI	Loop_of_H	900	-	-	0.039988	0.954253	0.044234	0.917752	0.038573	0.936098	0.036636	0.944647	0.041939	0.94015	0.037426	0.952375
HCLKidne	SCDC	sc-CMGAI	Loop_of_h	900	-	-	0.067414	0.702967	0.061398	0.726465	0.061053	0.732852	0.06284	0.721605	0.060747	0.739459	0.062799	0.723833
HCLKidne	SCDC	sc-CMGAI	Loop_of_h	900	-	-	0.057988	0.833945	0.059582	0.834094	0.060105	0.832939	0.05875	0.836095	0.058024	0.841954	0.058361	0.836913
HCLKidne	SCDC	sc-CMGAI	Principle_	900	-	-	0.0619	0.821646	0.061225	0.824387	0.064084	0.790985	0.064199	0.80818	0.059475	0.832365	0.062809	0.826141
HCLKidne	SCDC	sc-CMGAI	Proximal_	900	-	-	0.039945	0.931299	0.038503	0.933626	0.038773	0.932822	0.03876	0.93113	0.038806	0.932816	0.038671	0.931713
HCLKidne	SCDC	sc-CMGAI	Proximal_	900	-	-	0.089669	0.658681	0.090037	0.630856	0.090911	0.668463	0.09014	0.631722	0.091137	0.615596	0.088954	0.647726
HCLKidne	SCDC	sc-CMGAI	Proximal_	900	-	-	0.066192	0.864164	0.070245	0.847902	0.071103	0.850697	0.07097	0.840494	0.071048	0.843076	0.06945	0.854013
HCLKidne	SCDC	sc-CMGAI	Smooth_m	900	-	-	0.043994	0.953447	0.043265	0.953978	0.043194	0.953808	0.044598	0.952465	0.044835	0.953409	0.04313	0.949537
HCLKidne	SCDC	sc-CMGAI	B_cell(Pla	1000	-	-	0.026383	0.96701	0.02748	0.967643	0.026649	0.968485	0.025681	0.967941	0.026121	0.967139	0.025889	0.968966
HCLKidne	SCDC	sc-CMGAI	Dendritic_	1000	-	-	0.032772	0.94745	0.034407	0.943356	0.033591	0.947033	0.032873	0.949757	0.036398	0.942957	0.032091	0.9505
HCLKidne	SCDC	sc-CMGAI	Distal_tub	1000	-	-	0.063548	0.810879	0.073265	0.783181	0.074162	0.751882	0.060594	0.815639	0.067262	0.770789	0.066206	0.777894
HCLKidne	SCDC	sc-CMGAI	Distal_tub	1000	-	-	0.05021	0.88675	0.054987	0.870566	0.052761	0.875924	0.049816	0.896022	0.055657	0.867243	0.051995	0.879499
HCLKidne	SCDC	sc-CMGAI	Endothelia	1000	-	-	0.054946	0.880524	0.057447	0.874746	0.055915	0.881641	0.052593	0.891837	0.053719	0.884779	0.054539	0.883253
HCLKidne	SCDC	sc-CMGAI	Endothelia	1000	-	-	0.055968	0.915874	0.055324	0.920511	0.055835	0.919419	0.055362	0.919556	0.05581	0.924931	0.054644	0.922849
HCLKidne	SCDC	sc-CMGAI	Intercalate	1000	-	-	0.046864	0.922954	0.048067	0.920433	0.045045	0.927567	0.049687	0.923618	0.049042	0.924133	0.048064	0.921189
HCLKidne	SCDC	sc-CMGAI	Intercalate	1000	-	-	0.057772	0.896984	0.059565	0.912221	0.058373	0.913286	0.062684	0.90985	0.059735	0.913187	0.061231	0.886476
HCLKidne	SCDC	sc-CMGAI	Loop_of_H	1000	-	-	0.039758	0.954755	0.045145	0.914306	0.038534	0.936631	0.037183	0.942804	0.042228	0.937233	0.03748	0.954053
HCLKidne	SCDC	sc-CMGAI	Loop_of_h	1000	-	-	0.06724	0.704286	0.061247	0.730227	0.061257	0.731196	0.06257	0.724042	0.060894	0.737015	0.062398	0.728457
HCLKidne	SCDC	sc-CMGAI	Loop_of_h	1000	-	-	0.057985	0.833432	0.059182	0.835694	0.060091	0.833209	0.058437	0.837372	0.058568	0.839874	0.058167	0.838295
HCLKidne	SCDC	sc-CMGAI	Principle_	1000	-	-	0.061592	0.824279	0.061184	0.820468	0.064103	0.791648	0.06512	0.804611	0.059151	0.835799	0.062913	0.822263
HCLKidne	SCDC	sc-CMGAI	Proximal_	1000	-	-	0.039726	0.931021	0.038801	0.933353	0.039455	0.931978	0.038466	0.931852	0.039087	0.93246	0.038177	0.932966
HCLKidne	SCDC	sc-CMGAI	Proximal_	1000	-	-	0.089806	0.657195	0.090306	0.625386	0.090748	0.678035	0.089594	0.643361	0.091232	0.614527	0.089072	0.649118
HCLKidne	SCDC	sc-CMGAI	Proximal_	1000	-	-	0.06622	0.864073	0.070381	0.846513	0.070511	0.853179	0.07051	0.844236	0.071078	0.846865	0.0688	0.854565
HCLKidne	SCDC	sc-CMGAI	Smooth_m	1000	-	-	0.04395	0.953294	0.043224	0.954182	0.043572	0.954	0.044491	0.953352	0.045055	0.952732	0.043623	0.949843
HCLKidne	MuSiC	Control	B_cell(Pla	0	-	-	-	-	-	-	-	-	-	-	-	-	-	-
HCLKidne	MuSiC	Control	Dendritic_	0	-	-	-	-	-	-	-	-	-	-	-	-	-	-
HCLKidne	MuSiC	Control	Distal_tub	0	-	-	-	-	-	-	-	-	-	-	-	-	-	-
HCLKidne	MuSiC	Control	Distal_tub	0	-	-	-	-	-	-	-	-	-	-	-	-	-	-
HCLKidne	MuSiC	Control	Endothelia	0	-	-	-	-	-	-	-	-	-	-	-	-	-	-
HCLKidne	MuSiC	Control	Endothelia	0	-	-	-	-	-	-	-	-	-	-	-	-	-	-
HCLKidne	MuSiC	Control	Intercalate	0	-	-	-	-	-	-	-	-	-	-	-	-	-	-
HCLKidne	MuSiC	Control	Intercalate	0	-	-	-	-	-	-	-	-	-	-	-	-	-	-
HCLKidne	MuSiC	Control	Loop_of_H	0	-	-	-	-	-	-	-	-	-	-	-	-	-	-
HCLKidne	MuSiC	Control	Loop_of_h	0	-	-	-	-	-	-	-	-	-	-	-	-	-	-
HCLKidne	MuSiC	Control	Loop_of_h	0	-	-	-	-	-	-	-	-	-	-	-	-	-	-
HCLKidne	MuSiC	Control	Principle_	0	-	-	-	-	-	-	-	-	-	-	-	-	-	-

HCLKidne	MuSiC	Control	Proximal_	0	-	-	-	-	-	-	-	-	-	-	-	-	-	-
HCLKidne	MuSiC	Control	Proximal_	0	-	-	-	-	-	-	-	-	-	-	-	-	-	-
HCLKidne	MuSiC	Control	Proximal_	0	-	-	-	-	-	-	-	-	-	-	-	-	-	-
HCLKidne	MuSiC	Control	Smooth_m	0	-	-	-	-	-	-	-	-	-	-	-	-	-	-
HCLKidne	MuSiC	sc-CMGAI	B_cell(Pla	100	-	-	0.027666	0.965166	0.028331	0.966849	0.029044	0.96618	0.027402	0.96764	0.027815	0.96603	0.02819	0.967543
HCLKidne	MuSiC	sc-CMGAI	Dendritic_	100	-	-	0.034133	0.955863	0.033408	0.954647	0.032648	0.956062	0.03457	0.9572	0.032486	0.95323	0.035867	0.956453
HCLKidne	MuSiC	sc-CMGAI	Distal_tub	100	-	-	0.077553	0.8361	0.08925	0.776886	0.091119	0.782652	0.074019	0.837883	0.082024	0.795846	0.081223	0.812624
HCLKidne	MuSiC	sc-CMGAI	Distal_tub	100	-	-	0.048627	0.890258	0.052	0.875789	0.047395	0.897865	0.04902	0.896527	0.052803	0.870725	0.049407	0.88625
HCLKidne	MuSiC	sc-CMGAI	Endothelia	100	-	-	0.049082	0.913434	0.051455	0.905635	0.050856	0.905093	0.049027	0.917991	0.050409	0.903865	0.046988	0.918364
HCLKidne	MuSiC	sc-CMGAI	Endothelia	100	-	-	0.048143	0.927338	0.049157	0.929811	0.048483	0.930303	0.047044	0.927491	0.048796	0.929153	0.046171	0.932143
HCLKidne	MuSiC	sc-CMGAI	Intercalate	100	-	-	0.13072	0.895998	0.125933	0.897182	0.129692	0.896329	0.138207	0.885525	0.139226	0.886669	0.123408	0.898391
HCLKidne	MuSiC	sc-CMGAI	Intercalate	100	-	-	0.069166	0.913836	0.0732	0.921431	0.072515	0.92121	0.076766	0.9118	0.07578	0.915426	0.070688	0.894363
HCLKidne	MuSiC	sc-CMGAI	Loop_of_H	100	-	-	0.050451	0.955776	0.040857	0.941743	0.038822	0.946989	0.044676	0.951924	0.050664	0.953011	0.047725	0.956867
HCLKidne	MuSiC	sc-CMGAI	Loop_of_h	100	-	-	0.06174	0.717052	0.059361	0.788067	0.059941	0.763376	0.059752	0.751954	0.05957	0.778723	0.059096	0.748116
HCLKidne	MuSiC	sc-CMGAI	Loop_of_h	100	-	-	0.070958	0.827551	0.078304	0.833873	0.074798	0.835035	0.073452	0.828725	0.069909	0.834974	0.067631	0.8359
HCLKidne	MuSiC	sc-CMGAI	Principle_	100	-	-	0.060598	0.817534	0.060293	0.814262	0.065601	0.78243	0.058896	0.830055	0.061021	0.808984	0.058307	0.828865
HCLKidne	MuSiC	sc-CMGAI	Proximal_	100	-	-	0.038464	0.93133	0.035687	0.939251	0.036956	0.936499	0.036018	0.936518	0.037264	0.934537	0.036056	0.936282
HCLKidne	MuSiC	sc-CMGAI	Proximal_	100	-	-	0.089488	0.671731	0.085663	0.673457	0.087465	0.703963	0.087317	0.648718	0.086653	0.688975	0.082336	0.702295
HCLKidne	MuSiC	sc-CMGAI	Proximal_	100	-	-	0.063641	0.872958	0.063172	0.878268	0.064618	0.864013	0.064805	0.8625	0.066523	0.86753	0.06112	0.880594
HCLKidne	MuSiC	sc-CMGAI	Smooth_m	100	-	-	0.043067	0.958246	0.044137	0.959743	0.045957	0.957791	0.044358	0.957536	0.044983	0.956118	0.041314	0.956037
HCLKidne	MuSiC	sc-CMGAI	B_cell(Pla	200	-	-	0.028716	0.965053	0.029356	0.9668	0.028166	0.965689	0.027478	0.965327	0.027358	0.967404	0.026967	0.967039
HCLKidne	MuSiC	sc-CMGAI	Dendritic_	200	-	-	0.034324	0.95597	0.032214	0.956078	0.031744	0.956074	0.034141	0.957355	0.032357	0.952993	0.035592	0.957251
HCLKidne	MuSiC	sc-CMGAI	Distal_tub	200	-	-	0.078425	0.840579	0.086175	0.79645	0.090526	0.799911	0.073019	0.854162	0.084044	0.795622	0.08334	0.806255
HCLKidne	MuSiC	sc-CMGAI	Distal_tub	200	-	-	0.047804	0.894778	0.051089	0.880303	0.045738	0.902749	0.046446	0.90873	0.052081	0.87557	0.048497	0.88949
HCLKidne	MuSiC	sc-CMGAI	Endothelia	200	-	-	0.049133	0.911621	0.052179	0.902425	0.050228	0.910406	0.047495	0.921833	0.049642	0.908998	0.047161	0.916267
HCLKidne	MuSiC	sc-CMGAI	Endothelia	200	-	-	0.047429	0.9297	0.050435	0.92475	0.047587	0.929034	0.046475	0.929422	0.048492	0.929594	0.047045	0.929589
HCLKidne	MuSiC	sc-CMGAI	Intercalate	200	-	-	0.128666	0.890929	0.125575	0.898087	0.121909	0.902982	0.135307	0.885426	0.133404	0.891691	0.127776	0.892833
HCLKidne	MuSiC	sc-CMGAI	Intercalate	200	-	-	0.070239	0.908259	0.072367	0.916224	0.069182	0.925713	0.076678	0.90895	0.075641	0.91722	0.073618	0.901656
HCLKidne	MuSiC	sc-CMGAI	Loop_of_H	200	-	-	0.04747	0.955576	0.040564	0.941261	0.037817	0.949884	0.040478	0.950877	0.049689	0.953636	0.048717	0.955531
HCLKidne	MuSiC	sc-CMGAI	Loop_of_h	200	-	-	0.061089	0.723279	0.060148	0.782779	0.060669	0.764522	0.060163	0.751772	0.058042	0.774953	0.058283	0.762123
HCLKidne	MuSiC	sc-CMGAI	Loop_of_h	200	-	-	0.07294	0.829325	0.077816	0.832178	0.080432	0.827692	0.077296	0.826734	0.06725	0.843111	0.069668	0.839097
HCLKidne	MuSiC	sc-CMGAI	Principle_	200	-	-	0.060018	0.820154	0.060344	0.814451	0.065225	0.789203	0.056379	0.850594	0.062505	0.80145	0.060776	0.809956
HCLKidne	MuSiC	sc-CMGAI	Proximal_	200	-	-	0.038073	0.932468	0.034867	0.941011	0.036841	0.936687	0.036094	0.936867	0.0363	0.937692	0.036832	0.934495
HCLKidne	MuSiC	sc-CMGAI	Proximal_	200	-	-	0.089007	0.668583	0.083666	0.689367	0.089597	0.703616	0.087874	0.648028	0.085013	0.682174	0.083643	0.694398
HCLKidne	MuSiC	sc-CMGAI	Proximal_	200	-	-	0.062893	0.873421	0.061854	0.882724	0.064378	0.87203	0.065384	0.863565	0.065183	0.867648	0.062284	0.878347
HCLKidne	MuSiC	sc-CMGAI	Smooth_m	200	-	-	0.043313	0.95852	0.043367	0.958863	0.045987	0.957953	0.044478	0.957769	0.044267	0.957076	0.040254	0.956978

HCLKidne	MuSiC	sc-CMGAI	B_cell(Pla	300	-	-	0.029044	0.965545	0.0288	0.9664	0.028136	0.966761	0.02764	0.964885	0.027737	0.966666	0.027779	0.966986
HCLKidne	MuSiC	sc-CMGAI	Dendritic_	300	-	-	0.033771	0.955804	0.03251	0.95478	0.032222	0.956012	0.034152	0.957134	0.032097	0.952874	0.03491	0.957087
HCLKidne	MuSiC	sc-CMGAI	Distal_tub	300	-	-	0.081389	0.83078	0.088823	0.789794	0.091237	0.786995	0.074616	0.844947	0.08237	0.802278	0.081654	0.805337
HCLKidne	MuSiC	sc-CMGAI	Distal_tub	300	-	-	0.048354	0.891579	0.050328	0.883814	0.046076	0.903217	0.045587	0.910132	0.052078	0.873931	0.048094	0.891205
HCLKidne	MuSiC	sc-CMGAI	Endothelia	300	-	-	0.048945	0.912568	0.051203	0.907335	0.0498	0.909448	0.0476	0.919697	0.048817	0.913332	0.046067	0.920933
HCLKidne	MuSiC	sc-CMGAI	Endothelia	300	-	-	0.047752	0.929064	0.049031	0.927913	0.047678	0.930588	0.046204	0.932082	0.047511	0.930892	0.045778	0.93321
HCLKidne	MuSiC	sc-CMGAI	Intercalate	300	-	-	0.127405	0.889991	0.123269	0.900599	0.127942	0.896985	0.136835	0.885977	0.133588	0.893197	0.127871	0.889757
HCLKidne	MuSiC	sc-CMGAI	Intercalate	300	-	-	0.070244	0.908791	0.072062	0.92057	0.072787	0.925708	0.076873	0.910019	0.076115	0.914001	0.073379	0.902082
HCLKidne	MuSiC	sc-CMGAI	Loop_of_H	300	-	-	0.048349	0.95484	0.040552	0.940437	0.038717	0.946689	0.040784	0.951567	0.049454	0.953715	0.047697	0.955918
HCLKidne	MuSiC	sc-CMGAI	Loop_of_h	300	-	-	0.060506	0.728424	0.059441	0.780665	0.060285	0.770826	0.059699	0.760049	0.05878	0.77051	0.058477	0.758655
HCLKidne	MuSiC	sc-CMGAI	Loop_of_h	300	-	-	0.072309	0.832341	0.07495	0.83642	0.078447	0.830464	0.075433	0.830801	0.070552	0.837369	0.069515	0.841124
HCLKidne	MuSiC	sc-CMGAI	Principle_	300	-	-	0.061397	0.80888	0.062436	0.802845	0.067283	0.778531	0.058735	0.82747	0.060278	0.811656	0.059169	0.821995
HCLKidne	MuSiC	sc-CMGAI	Proximal_	300	-	-	0.038442	0.931496	0.034678	0.941741	0.036762	0.93785	0.036398	0.936115	0.036211	0.937256	0.036595	0.934113
HCLKidne	MuSiC	sc-CMGAI	Proximal_	300	-	-	0.087674	0.673077	0.083219	0.691232	0.088621	0.700148	0.087	0.65976	0.086184	0.681579	0.083784	0.683843
HCLKidne	MuSiC	sc-CMGAI	Proximal_	300	-	-	0.062909	0.872134	0.062691	0.88052	0.064529	0.870184	0.064664	0.868667	0.065695	0.870775	0.061108	0.885102
HCLKidne	MuSiC	sc-CMGAI	Smooth_m	300	-	-	0.042094	0.959491	0.044113	0.958434	0.045676	0.958681	0.043857	0.957211	0.044489	0.956768	0.040309	0.956839
HCLKidne	MuSiC	sc-CMGAI	B_cell(Pla	400	-	-	0.02824	0.965757	0.029256	0.966267	0.028465	0.967062	0.027757	0.965193	0.027721	0.966395	0.027672	0.967451
HCLKidne	MuSiC	sc-CMGAI	Dendritic_	400	-	-	0.033741	0.955952	0.032517	0.95469	0.032261	0.955996	0.034074	0.956973	0.031936	0.953407	0.034604	0.95698
HCLKidne	MuSiC	sc-CMGAI	Distal_tub	400	-	-	0.079253	0.840713	0.088535	0.787375	0.091439	0.787251	0.075039	0.841413	0.085113	0.789716	0.08111	0.815169
HCLKidne	MuSiC	sc-CMGAI	Distal_tub	400	-	-	0.047282	0.896682	0.050248	0.883621	0.046469	0.900773	0.045991	0.906872	0.052422	0.87348	0.048597	0.889072
HCLKidne	MuSiC	sc-CMGAI	Endothelia	400	-	-	0.04857	0.915215	0.051695	0.906797	0.049594	0.910365	0.047419	0.921574	0.049451	0.911552	0.047545	0.915896
HCLKidne	MuSiC	sc-CMGAI	Endothelia	400	-	-	0.047332	0.927428	0.048927	0.927207	0.047107	0.931903	0.046103	0.932009	0.047693	0.930241	0.046859	0.931474
HCLKidne	MuSiC	sc-CMGAI	Intercalate	400	-	-	0.126076	0.895957	0.123674	0.898342	0.126354	0.896969	0.134824	0.886041	0.136895	0.887326	0.12848	0.891213
HCLKidne	MuSiC	sc-CMGAI	Intercalate	400	-	-	0.069116	0.907632	0.071966	0.920697	0.071685	0.922541	0.075489	0.908029	0.077234	0.912772	0.074297	0.905442
HCLKidne	MuSiC	sc-CMGAI	Loop_of_H	400	-	-	0.046977	0.955055	0.039774	0.942291	0.038132	0.948474	0.041706	0.951921	0.050438	0.953523	0.047652	0.955651
HCLKidne	MuSiC	sc-CMGAI	Loop_of_h	400	-	-	0.061224	0.719309	0.059449	0.780926	0.059822	0.770961	0.059607	0.759641	0.058868	0.771934	0.058315	0.763709
HCLKidne	MuSiC	sc-CMGAI	Loop_of_h	400	-	-	0.072923	0.828033	0.075022	0.836362	0.077613	0.830543	0.0751	0.828612	0.071642	0.836085	0.069691	0.83988
HCLKidne	MuSiC	sc-CMGAI	Principle_	400	-	-	0.060138	0.81876	0.060634	0.812922	0.066206	0.782657	0.059742	0.820293	0.062043	0.799545	0.058357	0.825646
HCLKidne	MuSiC	sc-CMGAI	Proximal_	400	-	-	0.037732	0.93291	0.034727	0.941665	0.036788	0.937127	0.036303	0.936369	0.037054	0.935681	0.036559	0.934617
HCLKidne	MuSiC	sc-CMGAI	Proximal_	400	-	-	0.086977	0.6926	0.083504	0.685964	0.087314	0.702071	0.087748	0.649209	0.086445	0.685056	0.083472	0.688472
HCLKidne	MuSiC	sc-CMGAI	Proximal_	400	-	-	0.061949	0.879681	0.061842	0.881924	0.064316	0.870198	0.065625	0.861263	0.066089	0.866365	0.061581	0.882842
HCLKidne	MuSiC	sc-CMGAI	Smooth_m	400	-	-	0.04258	0.958473	0.043592	0.959416	0.045079	0.958731	0.043919	0.957612	0.044594	0.957607	0.041598	0.956806
HCLKidne	MuSiC	sc-CMGAI	B_cell(Pla	500	-	-	0.028631	0.965293	0.029839	0.966312	0.028496	0.966692	0.027538	0.966303	0.027348	0.966258	0.028257	0.967308
HCLKidne	MuSiC	sc-CMGAI	Dendritic_	500	-	-	0.034151	0.955175	0.032399	0.954412	0.032613	0.955872	0.034395	0.957473	0.03246	0.952714	0.034764	0.95714
HCLKidne	MuSiC	sc-CMGAI	Distal_tub	500	-	-	0.079474	0.836476	0.088247	0.792823	0.090689	0.788019	0.074574	0.840176	0.083208	0.79571	0.079766	0.820584
HCLKidne	MuSiC	sc-CMGAI	Distal_tub	500	-	-	0.048002	0.893682	0.049454	0.88759	0.046586	0.901488	0.047084	0.903613	0.051592	0.877071	0.047992	0.89128

HCLKidne	MuSiC	sc-CMGAI	Endothelia	500	-	-	0.048552	0.914258	0.051455	0.906293	0.049571	0.910452	0.047476	0.921291	0.049047	0.912113	0.047375	0.915733
HCLKidne	MuSiC	sc-CMGAI	Endothelia	500	-	-	0.047358	0.929311	0.049416	0.92661	0.047222	0.930964	0.045822	0.931354	0.047586	0.931797	0.046615	0.930805
HCLKidne	MuSiC	sc-CMGAI	Intercalate	500	-	-	0.129076	0.895156	0.125424	0.898659	0.12702	0.898591	0.137296	0.882754	0.135556	0.886216	0.129352	0.891209
HCLKidne	MuSiC	sc-CMGAI	Intercalate	500	-	-	0.069814	0.908818	0.073498	0.918642	0.071753	0.924521	0.07742	0.908775	0.078674	0.912065	0.073268	0.90395
HCLKidne	MuSiC	sc-CMGAI	Loop_of_H	500	-	-	0.047378	0.955775	0.038591	0.943779	0.037263	0.94968	0.043197	0.952779	0.049545	0.953844	0.047452	0.955498
HCLKidne	MuSiC	sc-CMGAI	Loop_of_h	500	-	-	0.061421	0.717653	0.059472	0.782488	0.059586	0.771657	0.060237	0.758202	0.058558	0.778934	0.058735	0.75866
HCLKidne	MuSiC	sc-CMGAI	Loop_of_h	500	-	-	0.072326	0.828205	0.076033	0.834073	0.076338	0.832167	0.076163	0.830133	0.071099	0.837631	0.070379	0.838765
HCLKidne	MuSiC	sc-CMGAI	Principle_	500	-	-	0.059376	0.823207	0.060377	0.813991	0.065621	0.789024	0.059245	0.822523	0.060616	0.808248	0.056726	0.837343
HCLKidne	MuSiC	sc-CMGAI	Proximal_	500	-	-	0.037875	0.932974	0.034799	0.941812	0.037221	0.936236	0.036165	0.936714	0.037138	0.935593	0.036723	0.934227
HCLKidne	MuSiC	sc-CMGAI	Proximal_	500	-	-	0.087837	0.681769	0.083532	0.689772	0.088239	0.704854	0.086549	0.662759	0.085188	0.685646	0.083385	0.692552
HCLKidne	MuSiC	sc-CMGAI	Proximal_	500	-	-	0.062065	0.876264	0.062018	0.882497	0.064522	0.871359	0.064655	0.867285	0.065553	0.869342	0.061462	0.883418
HCLKidne	MuSiC	sc-CMGAI	Smooth_m	500	-	-	0.042623	0.959143	0.043233	0.958596	0.045188	0.958632	0.043758	0.957922	0.044325	0.958039	0.041056	0.957468
HCLKidne	MuSiC	sc-CMGAI	B_cell(Pla	600	-	-	0.028319	0.965971	0.028648	0.966942	0.028043	0.966542	0.027051	0.966655	0.027687	0.966244	0.027615	0.967617
HCLKidne	MuSiC	sc-CMGAI	Dendritic_	600	-	-	0.033851	0.955898	0.032377	0.954551	0.032567	0.956073	0.034279	0.957064	0.03217	0.953213	0.034668	0.956982
HCLKidne	MuSiC	sc-CMGAI	Distal_tub	600	-	-	0.080329	0.832162	0.08783	0.795388	0.09088	0.789626	0.07489	0.844347	0.085024	0.790052	0.081858	0.810605
HCLKidne	MuSiC	sc-CMGAI	Distal_tub	600	-	-	0.047561	0.895414	0.049383	0.888126	0.045934	0.902811	0.046333	0.905265	0.051747	0.876544	0.048168	0.890426
HCLKidne	MuSiC	sc-CMGAI	Endothelia	600	-	-	0.04849	0.915309	0.051783	0.904731	0.049377	0.912024	0.047966	0.919475	0.048977	0.912773	0.046978	0.917681
HCLKidne	MuSiC	sc-CMGAI	Endothelia	600	-	-	0.047217	0.928509	0.049677	0.926768	0.047058	0.932096	0.046313	0.929984	0.04764	0.931048	0.046443	0.930078
HCLKidne	MuSiC	sc-CMGAI	Intercalate	600	-	-	0.130494	0.891375	0.123802	0.899325	0.125722	0.899275	0.135236	0.887578	0.137203	0.887708	0.12942	0.890121
HCLKidne	MuSiC	sc-CMGAI	Intercalate	600	-	-	0.070539	0.908355	0.072204	0.91941	0.071537	0.923931	0.075997	0.909768	0.0767	0.913865	0.073406	0.904599
HCLKidne	MuSiC	sc-CMGAI	Loop_of_H	600	-	-	0.048292	0.954689	0.039185	0.942731	0.037946	0.949481	0.041844	0.952257	0.049692	0.953418	0.047582	0.955784
HCLKidne	MuSiC	sc-CMGAI	Loop_of_h	600	-	-	0.06094	0.723604	0.059396	0.78113	0.059603	0.774122	0.060009	0.753463	0.059076	0.769612	0.058215	0.76239
HCLKidne	MuSiC	sc-CMGAI	Loop_of_h	600	-	-	0.070446	0.830806	0.075449	0.834419	0.076632	0.832336	0.076349	0.829032	0.07039	0.836448	0.068922	0.843706
HCLKidne	MuSiC	sc-CMGAI	Principle_	600	-	-	0.060794	0.813831	0.061038	0.81261	0.066382	0.781046	0.058203	0.832694	0.061488	0.801777	0.05839	0.825194
HCLKidne	MuSiC	sc-CMGAI	Proximal_	600	-	-	0.038412	0.931419	0.034766	0.941585	0.036899	0.93735	0.036364	0.93655	0.036532	0.936873	0.036779	0.934208
HCLKidne	MuSiC	sc-CMGAI	Proximal_	600	-	-	0.088323	0.68405	0.083241	0.691456	0.087711	0.708284	0.08796	0.649195	0.085188	0.692459	0.082606	0.69778
HCLKidne	MuSiC	sc-CMGAI	Proximal_	600	-	-	0.062086	0.875829	0.062097	0.881335	0.064635	0.870944	0.065374	0.863159	0.065089	0.871122	0.060998	0.886486
HCLKidne	MuSiC	sc-CMGAI	Smooth_m	600	-	-	0.042429	0.95921	0.043513	0.958793	0.04542	0.959285	0.043745	0.958464	0.044581	0.957488	0.041328	0.957199
HCLKidne	MuSiC	sc-CMGAI	B_cell(Pla	700	-	-	0.028235	0.965516	0.029301	0.966337	0.028863	0.967208	0.027141	0.96631	0.027614	0.966276	0.027982	0.967287
HCLKidne	MuSiC	sc-CMGAI	Dendritic_	700	-	-	0.03397	0.955786	0.032321	0.955165	0.032288	0.956282	0.033894	0.95819	0.031996	0.953389	0.034453	0.956899
HCLKidne	MuSiC	sc-CMGAI	Distal_tub	700	-	-	0.080371	0.834998	0.088199	0.789614	0.091519	0.790872	0.075171	0.843153	0.084916	0.789587	0.080453	0.819654
HCLKidne	MuSiC	sc-CMGAI	Distal_tub	700	-	-	0.047724	0.894244	0.050463	0.882396	0.045855	0.903429	0.045361	0.90994	0.052072	0.87416	0.048418	0.889524
HCLKidne	MuSiC	sc-CMGAI	Endothelia	700	-	-	0.04872	0.913613	0.051679	0.905251	0.049873	0.91019	0.047591	0.920861	0.048715	0.913506	0.046863	0.917557
HCLKidne	MuSiC	sc-CMGAI	Endothelia	700	-	-	0.047599	0.929259	0.049646	0.92795	0.047117	0.931455	0.046322	0.930187	0.047451	0.93115	0.046627	0.92994
HCLKidne	MuSiC	sc-CMGAI	Intercalate	700	-	-	0.128229	0.89362	0.125272	0.898283	0.127754	0.897151	0.134941	0.887548	0.136038	0.886415	0.126557	0.892344
HCLKidne	MuSiC	sc-CMGAI	Intercalate	700	-	-	0.069598	0.90454	0.0731	0.921048	0.071234	0.923615	0.076285	0.908711	0.076961	0.913877	0.072062	0.900374

HCLKidne	MuSiC	sc-CMGAI	Loop_of_H	700	-	-	0.04901	0.954262	0.040138	0.939686	0.037273	0.949891	0.041215	0.952633	0.04971	0.953481	0.047844	0.956325
HCLKidne	MuSiC	sc-CMGAI	Loop_of_h	700	-	-	0.061014	0.722444	0.059864	0.781697	0.059718	0.770169	0.059401	0.760612	0.058657	0.777676	0.058591	0.756849
HCLKidne	MuSiC	sc-CMGAI	Loop_of_h	700	-	-	0.071359	0.829295	0.076348	0.832173	0.077833	0.831572	0.075213	0.833627	0.070664	0.839126	0.069795	0.840929
HCLKidne	MuSiC	sc-CMGAI	Principle_	700	-	-	0.061028	0.81332	0.060984	0.81111	0.065653	0.785346	0.059235	0.825174	0.060874	0.805691	0.056565	0.839121
HCLKidne	MuSiC	sc-CMGAI	Proximal_	700	-	-	0.037998	0.932429	0.034724	0.941323	0.037017	0.93688	0.036335	0.936596	0.036165	0.937581	0.03657	0.934613
HCLKidne	MuSiC	sc-CMGAI	Proximal_	700	-	-	0.087344	0.684071	0.083685	0.686295	0.088159	0.701028	0.087746	0.651884	0.085423	0.686977	0.083261	0.692124
HCLKidne	MuSiC	sc-CMGAI	Proximal_	700	-	-	0.062238	0.875707	0.062157	0.882427	0.064127	0.870348	0.065452	0.864419	0.065736	0.868254	0.06125	0.883932
HCLKidne	MuSiC	sc-CMGAI	Smooth_m	700	-	-	0.042922	0.959889	0.043319	0.959102	0.045421	0.959294	0.043372	0.957547	0.044912	0.957596	0.041099	0.957218
HCLKidne	MuSiC	sc-CMGAI	B_cell(Pla	800	-	-	0.028308	0.965746	0.028999	0.966282	0.028145	0.966669	0.027151	0.966241	0.027241	0.966088	0.027823	0.967302
HCLKidne	MuSiC	sc-CMGAI	Dendritic_	800	-	-	0.03388	0.955651	0.032716	0.954627	0.032382	0.955833	0.033861	0.957713	0.032068	0.953106	0.03452	0.957437
HCLKidne	MuSiC	sc-CMGAI	Distal_tub	800	-	-	0.080235	0.83821	0.088348	0.789194	0.090557	0.789512	0.075274	0.841231	0.084153	0.794547	0.081426	0.81414
HCLKidne	MuSiC	sc-CMGAI	Distal_tub	800	-	-	0.047439	0.895179	0.050184	0.884097	0.045915	0.904027	0.046156	0.906847	0.051803	0.875094	0.048073	0.891033
HCLKidne	MuSiC	sc-CMGAI	Endothelia	800	-	-	0.048673	0.9134	0.051204	0.907892	0.049812	0.909742	0.0471	0.922588	0.04925	0.909757	0.047078	0.91618
HCLKidne	MuSiC	sc-CMGAI	Endothelia	800	-	-	0.047536	0.928938	0.049375	0.927728	0.047003	0.930611	0.045587	0.93194	0.047785	0.931552	0.046801	0.930471
HCLKidne	MuSiC	sc-CMGAI	Intercalate	800	-	-	0.12875	0.891443	0.124952	0.898105	0.125108	0.899255	0.136532	0.886112	0.137756	0.884754	0.125262	0.894143
HCLKidne	MuSiC	sc-CMGAI	Intercalate	800	-	-	0.070375	0.910009	0.072872	0.918822	0.071049	0.925303	0.076429	0.908686	0.077668	0.912867	0.072642	0.903195
HCLKidne	MuSiC	sc-CMGAI	Loop_of_H	800	-	-	0.048034	0.954426	0.039338	0.942175	0.037738	0.948717	0.041854	0.952671	0.049784	0.953603	0.047079	0.956459
HCLKidne	MuSiC	sc-CMGAI	Loop_of_h	800	-	-	0.060635	0.726975	0.059284	0.785821	0.059752	0.769346	0.059723	0.760313	0.058806	0.774139	0.058453	0.759777
HCLKidne	MuSiC	sc-CMGAI	Loop_of_h	800	-	-	0.072408	0.831624	0.076078	0.834757	0.077062	0.831695	0.074886	0.831444	0.069906	0.837893	0.069847	0.84006
HCLKidne	MuSiC	sc-CMGAI	Principle_	800	-	-	0.060732	0.814444	0.059973	0.817408	0.06585	0.783748	0.059497	0.822754	0.060746	0.806191	0.05912	0.821105
HCLKidne	MuSiC	sc-CMGAI	Proximal_	800	-	-	0.038096	0.932372	0.034596	0.94177	0.037087	0.936526	0.036536	0.935792	0.036605	0.936514	0.036658	0.934344
HCLKidne	MuSiC	sc-CMGAI	Proximal_	800	-	-	0.087636	0.68582	0.08357	0.6848	0.088106	0.705811	0.087387	0.657065	0.085295	0.693869	0.083328	0.688593
HCLKidne	MuSiC	sc-CMGAI	Proximal_	800	-	-	0.062222	0.87735	0.062502	0.879583	0.064678	0.869519	0.065235	0.865648	0.065566	0.871112	0.061084	0.884399
HCLKidne	MuSiC	sc-CMGAI	Smooth_m	800	-	-	0.042648	0.959129	0.04309	0.959048	0.044971	0.958527	0.043543	0.957867	0.044581	0.957792	0.041489	0.956989
HCLKidne	MuSiC	sc-CMGAI	B_cell(Pla	900	-	-	0.02848	0.965564	0.028964	0.96662	0.028181	0.967077	0.02761	0.965914	0.027829	0.966651	0.027682	0.96744
HCLKidne	MuSiC	sc-CMGAI	Dendritic_	900	-	-	0.033988	0.955582	0.03251	0.954872	0.032622	0.956287	0.034134	0.957503	0.032051	0.953332	0.034823	0.957067
HCLKidne	MuSiC	sc-CMGAI	Distal_tub	900	-	-	0.08003	0.837006	0.089126	0.788998	0.091376	0.786583	0.07523	0.844281	0.084659	0.786456	0.079827	0.824377
HCLKidne	MuSiC	sc-CMGAI	Distal_tub	900	-	-	0.048304	0.891588	0.050275	0.882591	0.046349	0.901196	0.045566	0.908081	0.052518	0.872204	0.047839	0.892646
HCLKidne	MuSiC	sc-CMGAI	Endothelia	900	-	-	0.048534	0.913653	0.051387	0.906512	0.049718	0.910389	0.04721	0.921884	0.048737	0.912375	0.04689	0.918329
HCLKidne	MuSiC	sc-CMGAI	Endothelia	900	-	-	0.047412	0.929553	0.049494	0.926148	0.04744	0.930338	0.045758	0.931562	0.047242	0.933009	0.046566	0.929055
HCLKidne	MuSiC	sc-CMGAI	Intercalate	900	-	-	0.127943	0.892363	0.12717	0.895983	0.124176	0.901115	0.136354	0.88472	0.1364	0.886342	0.128235	0.891846
HCLKidne	MuSiC	sc-CMGAI	Intercalate	900	-	-	0.070214	0.910155	0.073724	0.919117	0.070559	0.923922	0.07738	0.909563	0.076732	0.913845	0.072784	0.904694
HCLKidne	MuSiC	sc-CMGAI	Loop_of_H	900	-	-	0.048548	0.95525	0.040173	0.941357	0.038526	0.948519	0.041959	0.952468	0.050346	0.953787	0.047562	0.955309
HCLKidne	MuSiC	sc-CMGAI	Loop_of_h	900	-	-	0.060584	0.727801	0.059916	0.78381	0.060178	0.767368	0.059982	0.756822	0.058932	0.774787	0.058466	0.757596
HCLKidne	MuSiC	sc-CMGAI	Loop_of_h	900	-	-	0.073166	0.830492	0.076799	0.835441	0.078393	0.828951	0.076109	0.830425	0.071684	0.835568	0.069186	0.841282
HCLKidne	MuSiC	sc-CMGAI	Principle_	900	-	-	0.060267	0.817491	0.06092	0.810466	0.065521	0.788922	0.05871	0.825714	0.060765	0.806323	0.057176	0.834988

HCLKidne	MuSiC	sc-CMGAI	Proximal_	900	-	-	0.037782	0.933175	0.034667	0.941486	0.037013	0.936833	0.036312	0.936368	0.036762	0.936232	0.036976	0.933475
HCLKidne	MuSiC	sc-CMGAI	Proximal_	900	-	-	0.087157	0.685209	0.083337	0.691478	0.088372	0.702112	0.087966	0.64679	0.085544	0.687171	0.083429	0.68984
HCLKidne	MuSiC	sc-CMGAI	Proximal_	900	-	-	0.062482	0.876375	0.061988	0.882278	0.064568	0.869305	0.065741	0.862793	0.065387	0.870159	0.061531	0.88251
HCLKidne	MuSiC	sc-CMGAI	Smooth_m	900	-	-	0.042798	0.959139	0.043228	0.958979	0.045144	0.958574	0.04387	0.957386	0.044454	0.957469	0.040822	0.957475
HCLKidne	MuSiC	sc-CMGAI	B_cell(Pla	1000	-	-	0.028155	0.965545	0.02937	0.966184	0.028543	0.967089	0.027306	0.96641	0.027919	0.965867	0.027301	0.967785
HCLKidne	MuSiC	sc-CMGAI	Dendritic_	1000	-	-	0.033907	0.955801	0.032745	0.954737	0.032169	0.956038	0.033904	0.957854	0.032139	0.953362	0.034897	0.957464
HCLKidne	MuSiC	sc-CMGAI	Distal_tub	1000	-	-	0.080184	0.832811	0.088592	0.789888	0.090248	0.801782	0.07425	0.84709	0.083213	0.795295	0.081717	0.811073
HCLKidne	MuSiC	sc-CMGAI	Distal_tub	1000	-	-	0.047562	0.895396	0.050544	0.881389	0.045826	0.903262	0.046196	0.90577	0.051899	0.874972	0.048458	0.889435
HCLKidne	MuSiC	sc-CMGAI	Endothelia	1000	-	-	0.04849	0.914617	0.051731	0.904793	0.049829	0.909345	0.047131	0.92209	0.048711	0.912938	0.046926	0.917133
HCLKidne	MuSiC	sc-CMGAI	Endothelia	1000	-	-	0.04731	0.928995	0.049549	0.92675	0.04706	0.931967	0.045818	0.932158	0.047366	0.931865	0.046678	0.931082
HCLKidne	MuSiC	sc-CMGAI	Intercalate	1000	-	-	0.128074	0.892009	0.124227	0.89884	0.125825	0.897721	0.135942	0.885756	0.135554	0.886222	0.127064	0.893155
HCLKidne	MuSiC	sc-CMGAI	Intercalate	1000	-	-	0.070146	0.908205	0.073092	0.920583	0.07092	0.923933	0.076293	0.909373	0.077217	0.913584	0.073209	0.902427
HCLKidne	MuSiC	sc-CMGAI	Loop_of_H	1000	-	-	0.048622	0.953741	0.040153	0.940729	0.037477	0.949309	0.042274	0.951633	0.049737	0.954034	0.047728	0.95583
HCLKidne	MuSiC	sc-CMGAI	Loop_of_h	1000	-	-	0.060778	0.724269	0.059739	0.783811	0.059568	0.769243	0.059727	0.759261	0.059173	0.77347	0.058323	0.76377
HCLKidne	MuSiC	sc-CMGAI	Loop_of_h	1000	-	-	0.072726	0.82949	0.07727	0.833362	0.077193	0.830746	0.074784	0.830993	0.071737	0.835902	0.070628	0.840456
HCLKidne	MuSiC	sc-CMGAI	Principle_	1000	-	-	0.060785	0.813857	0.060984	0.810272	0.064381	0.792749	0.059149	0.824224	0.060077	0.811496	0.057876	0.830025
HCLKidne	MuSiC	sc-CMGAI	Proximal_	1000	-	-	0.037677	0.933264	0.034781	0.941583	0.037088	0.936651	0.036267	0.93671	0.03658	0.936877	0.036602	0.93439
HCLKidne	MuSiC	sc-CMGAI	Proximal_	1000	-	-	0.087604	0.684449	0.083502	0.688717	0.087653	0.710141	0.087368	0.656899	0.084893	0.689863	0.082596	0.69659
HCLKidne	MuSiC	sc-CMGAI	Proximal_	1000	-	-	0.062253	0.877387	0.062107	0.881588	0.063988	0.87224	0.06503	0.866436	0.064961	0.872928	0.06097	0.885806
HCLKidne	MuSiC	sc-CMGAI	Smooth_m	1000	-	-	0.042834	0.958968	0.043518	0.958833	0.045242	0.958353	0.043783	0.957451	0.044452	0.957801	0.041185	0.9577
HCLKidne	BisqueRN	Control	B_cell(Pla	0	0.096326	0.962844	-	-	-	-	-	-	-	-	-	-	-	-
HCLKidne	BisqueRN	Control	Dendritic_	0	0.075356	0.943005	-	-	-	-	-	-	-	-	-	-	-	-
HCLKidne	BisqueRN	Control	Distal_tub	0	0.088816	0.677738	-	-	-	-	-	-	-	-	-	-	-	-
HCLKidne	BisqueRN	Control	Distal_tub	0	0.077834	0.798487	-	-	-	-	-	-	-	-	-	-	-	-
HCLKidne	BisqueRN	Control	Endothelia	0	0.082451	0.883601	-	-	-	-	-	-	-	-	-	-	-	-
HCLKidne	BisqueRN	Control	Endothelia	0	0.099094	0.888234	-	-	-	-	-	-	-	-	-	-	-	-
HCLKidne	BisqueRN	Control	Intercalate	0	0.488911	0.791779	-	-	-	-	-	-	-	-	-	-	-	-
HCLKidne	BisqueRN	Control	Intercalate	0	0.075499	0.795975	-	-	-	-	-	-	-	-	-	-	-	-
HCLKidne	BisqueRN	Control	Loop_of_H	0	0.063421	0.938439	-	-	-	-	-	-	-	-	-	-	-	-
HCLKidne	BisqueRN	Control	Loop_of_h	0	0.084067	0.575618	-	-	-	-	-	-	-	-	-	-	-	-
HCLKidne	BisqueRN	Control	Loop_of_h	0	0.079143	0.662706	-	-	-	-	-	-	-	-	-	-	-	-
HCLKidne	BisqueRN	Control	Principle_	0	0.074938	0.750774	-	-	-	-	-	-	-	-	-	-	-	-
HCLKidne	BisqueRN	Control	Proximal_	0	0.072256	0.884956	-	-	-	-	-	-	-	-	-	-	-	-
HCLKidne	BisqueRN	Control	Proximal_	0	0.092971	0.570578	-	-	-	-	-	-	-	-	-	-	-	-
HCLKidne	BisqueRN	Control	Proximal_	0	0.096088	0.814413	-	-	-	-	-	-	-	-	-	-	-	-
HCLKidne	BisqueRN	Control	Smooth_m	0	0.091044	0.929942	-	-	-	-	-	-	-	-	-	-	-	-

HCLKidne	BisqueRN	sc-CMGAI	B_cell(Pla	100	-	-	0.082572	0.961262	0.082454	0.959425	0.081706	0.962662	0.082534	0.960649	0.082714	0.96453	0.081765	0.962918
HCLKidne	BisqueRN	sc-CMGAI	Dendritic_	100	-	-	0.066201	0.936552	0.067433	0.939978	0.066611	0.939382	0.066653	0.945552	0.066904	0.932963	0.066877	0.938377
HCLKidne	BisqueRN	sc-CMGAI	Distal_tub	100	-	-	0.082228	0.684597	0.095739	0.432512	0.096706	0.296442	0.082151	0.646745	0.091839	0.546727	0.089719	0.504289
HCLKidne	BisqueRN	sc-CMGAI	Distal_tub	100	-	-	0.079041	0.757123	0.075947	0.826229	0.077298	0.770286	0.079436	0.768023	0.080218	0.76749	0.080086	0.721812
HCLKidne	BisqueRN	sc-CMGAI	Endothelia	100	-	-	0.080722	0.863297	0.084852	0.824558	0.080296	0.810959	0.080411	0.860663	0.085922	0.83589	0.084786	0.837777
HCLKidne	BisqueRN	sc-CMGAI	Endothelia	100	-	-	0.093629	0.851972	0.090525	0.885237	0.092535	0.857381	0.091053	0.882929	0.089644	0.866402	0.0899	0.880208
HCLKidne	BisqueRN	sc-CMGAI	Intercalate	100	-	-	0.332235	0.853837	0.33512	0.853104	0.331002	0.86512	0.333567	0.855762	0.333468	0.857873	0.334933	0.851017
HCLKidne	BisqueRN	sc-CMGAI	Intercalate	100	-	-	0.081059	0.766174	0.077092	0.757101	0.077221	0.759276	0.079786	0.818362	0.07592	0.746792	0.078599	0.777113
HCLKidne	BisqueRN	sc-CMGAI	Loop_of_H	100	-	-	0.068902	0.941074	0.071568	0.900597	0.067842	0.889994	0.068903	0.932034	0.070984	0.922313	0.0686	0.937455
HCLKidne	BisqueRN	sc-CMGAI	Loop_of_h	100	-	-	0.080756	0.433073	0.076631	0.57816	0.081826	0.411594	0.080146	0.468592	0.079931	0.534904	0.080236	0.465128
HCLKidne	BisqueRN	sc-CMGAI	Loop_of_h	100	-	-	0.082371	0.655203	0.080938	0.66639	0.082419	0.596466	0.079921	0.666363	0.080018	0.653721	0.080498	0.69285
HCLKidne	BisqueRN	sc-CMGAI	Principle_	100	-	-	0.076236	0.685811	0.07336	0.707703	0.077411	0.644927	0.085874	0.521648	0.076259	0.676956	0.080442	0.610859
HCLKidne	BisqueRN	sc-CMGAI	Proximal_	100	-	-	0.072001	0.899104	0.069854	0.882817	0.071955	0.854365	0.071161	0.896974	0.0716	0.882584	0.069699	0.910223
HCLKidne	BisqueRN	sc-CMGAI	Proximal_	100	-	-	0.101478	0.450289	0.103328	0.413069	0.106344	0.347414	0.100643	0.463394	0.100625	0.493257	0.103796	0.410313
HCLKidne	BisqueRN	sc-CMGAI	Proximal_	100	-	-	0.092906	0.779188	0.096631	0.747809	0.096647	0.68878	0.099179	0.694479	0.09414	0.662931	0.098919	0.696021
HCLKidne	BisqueRN	sc-CMGAI	Smooth_m	100	-	-	0.08657	0.900967	0.08539	0.933436	0.086219	0.923673	0.086643	0.929275	0.088192	0.916165	0.085376	0.908059
HCLKidne	BisqueRN	sc-CMGAI	B_cell(Pla	200	-	-	0.082455	0.959935	0.082469	0.959247	0.08212	0.961336	0.082471	0.960836	0.082552	0.962665	0.081953	0.962794
HCLKidne	BisqueRN	sc-CMGAI	Dendritic_	200	-	-	0.066361	0.936537	0.067079	0.933098	0.066177	0.931759	0.06636	0.944394	0.067644	0.93632	0.068891	0.940752
HCLKidne	BisqueRN	sc-CMGAI	Distal_tub	200	-	-	0.084905	0.663826	0.091426	0.592178	0.096681	0.273941	0.082248	0.592728	0.087647	0.591757	0.088351	0.499747
HCLKidne	BisqueRN	sc-CMGAI	Distal_tub	200	-	-	0.078493	0.79358	0.078866	0.755298	0.075708	0.769793	0.081286	0.756184	0.082746	0.735913	0.083208	0.705995
HCLKidne	BisqueRN	sc-CMGAI	Endothelia	200	-	-	0.081614	0.850617	0.086528	0.807085	0.082437	0.745033	0.079346	0.844481	0.083538	0.834034	0.084816	0.83728
HCLKidne	BisqueRN	sc-CMGAI	Endothelia	200	-	-	0.095702	0.823946	0.090267	0.893561	0.092851	0.853889	0.093524	0.865543	0.09468	0.855022	0.090204	0.88362
HCLKidne	BisqueRN	sc-CMGAI	Intercalate	200	-	-	0.33513	0.828235	0.335873	0.857524	0.328362	0.845566	0.334413	0.8507	0.333071	0.85314	0.334816	0.846788
HCLKidne	BisqueRN	sc-CMGAI	Intercalate	200	-	-	0.084614	0.77036	0.076194	0.76974	0.075483	0.759253	0.082449	0.790428	0.074229	0.784795	0.079144	0.793344
HCLKidne	BisqueRN	sc-CMGAI	Loop_of_H	200	-	-	0.070383	0.944661	0.07867	0.852651	0.068152	0.862633	0.069974	0.924225	0.0715	0.912726	0.068467	0.941218
HCLKidne	BisqueRN	sc-CMGAI	Loop_of_h	200	-	-	0.078312	0.498797	0.077874	0.56283	0.084185	0.356485	0.080467	0.474334	0.0797	0.516409	0.078599	0.505537
HCLKidne	BisqueRN	sc-CMGAI	Loop_of_h	200	-	-	0.084604	0.64132	0.079158	0.692294	0.085198	0.535008	0.080853	0.661254	0.080285	0.657103	0.081808	0.690404
HCLKidne	BisqueRN	sc-CMGAI	Principle_	200	-	-	0.076389	0.700284	0.076832	0.664886	0.077189	0.654786	0.089343	0.45823	0.080749	0.609398	0.084264	0.559276
HCLKidne	BisqueRN	sc-CMGAI	Proximal_	200	-	-	0.072065	0.902544	0.071166	0.866426	0.075443	0.785349	0.07135	0.88797	0.073985	0.84994	0.070402	0.894018
HCLKidne	BisqueRN	sc-CMGAI	Proximal_	200	-	-	0.10461	0.411884	0.100694	0.474963	0.104686	0.390976	0.105024	0.376636	0.102944	0.437352	0.107204	0.343989
HCLKidne	BisqueRN	sc-CMGAI	Proximal_	200	-	-	0.093307	0.750043	0.096537	0.726955	0.095679	0.711782	0.099887	0.693339	0.095664	0.700873	0.09802	0.694579
HCLKidne	BisqueRN	sc-CMGAI	Smooth_m	200	-	-	0.08784	0.894669	0.086465	0.924739	0.087956	0.897721	0.088021	0.929274	0.089132	0.9188	0.086266	0.909681
HCLKidne	BisqueRN	sc-CMGAI	B_cell(Pla	300	-	-	0.082215	0.959494	0.082465	0.960226	0.081774	0.960161	0.082715	0.961064	0.082549	0.96314	0.081993	0.961523
HCLKidne	BisqueRN	sc-CMGAI	Dendritic_	300	-	-	0.066766	0.937489	0.067636	0.931088	0.06643	0.93168	0.067916	0.946005	0.067247	0.930515	0.069133	0.937569
HCLKidne	BisqueRN	sc-CMGAI	Distal_tub	300	-	-	0.083849	0.688123	0.087877	0.623606	0.092113	0.474989	0.084339	0.546028	0.086029	0.617974	0.087007	0.51525
HCLKidne	BisqueRN	sc-CMGAI	Distal_tub	300	-	-	0.073586	0.835809	0.080468	0.769112	0.082816	0.758944	0.081027	0.776399	0.084215	0.720908	0.082449	0.73139

HCLKidne	BisqueRN	sc-CMGAI	Endothelia	300	-	-	0.082506	0.837373	0.086317	0.794815	0.081932	0.750539	0.080338	0.85221	0.08424	0.838735	0.083598	0.841925
HCLKidne	BisqueRN	sc-CMGAI	Endothelia	300	-	-	0.096071	0.824063	0.090052	0.881825	0.09435	0.849399	0.091319	0.883203	0.093664	0.848898	0.09091	0.888329
HCLKidne	BisqueRN	sc-CMGAI	Intercalate	300	-	-	0.3359	0.822649	0.335127	0.85521	0.32864	0.843386	0.334831	0.853963	0.332525	0.856754	0.334428	0.843685
HCLKidne	BisqueRN	sc-CMGAI	Intercalate	300	-	-	0.086291	0.786162	0.076088	0.796045	0.078486	0.727034	0.083176	0.785844	0.073042	0.785187	0.07945	0.80387
HCLKidne	BisqueRN	sc-CMGAI	Loop_of_H	300	-	-	0.070353	0.947477	0.08016	0.821727	0.069453	0.8335	0.070346	0.925258	0.072047	0.90591	0.070101	0.93483
HCLKidne	BisqueRN	sc-CMGAI	Loop_of_h	300	-	-	0.077061	0.537063	0.076486	0.586542	0.083433	0.372495	0.078032	0.573813	0.080185	0.505329	0.078688	0.502508
HCLKidne	BisqueRN	sc-CMGAI	Loop_of_h	300	-	-	0.084461	0.675872	0.079851	0.678381	0.083577	0.573495	0.081214	0.675085	0.080998	0.634831	0.081356	0.697143
HCLKidne	BisqueRN	sc-CMGAI	Principle_	300	-	-	0.079411	0.666875	0.08144	0.601377	0.082694	0.573108	0.090738	0.430142	0.078895	0.65221	0.085547	0.544233
HCLKidne	BisqueRN	sc-CMGAI	Proximal_	300	-	-	0.072334	0.905491	0.071652	0.855683	0.076043	0.783756	0.072109	0.878082	0.075166	0.829833	0.070825	0.89458
HCLKidne	BisqueRN	sc-CMGAI	Proximal_	300	-	-	0.1042	0.430428	0.10002	0.499443	0.104603	0.388937	0.104135	0.408707	0.103091	0.449974	0.108975	0.319222
HCLKidne	BisqueRN	sc-CMGAI	Proximal_	300	-	-	0.094206	0.752454	0.096134	0.750168	0.097012	0.709685	0.098582	0.718093	0.095004	0.649375	0.097604	0.712349
HCLKidne	BisqueRN	sc-CMGAI	Smooth_m	300	-	-	0.088196	0.881133	0.086558	0.918293	0.088993	0.89462	0.088118	0.92705	0.089632	0.915218	0.086852	0.896169
HCLKidne	BisqueRN	sc-CMGAI	B_cell(Pla	400	-	-	0.082447	0.959532	0.08215	0.959576	0.081722	0.960689	0.082447	0.959869	0.0825	0.963298	0.082007	0.962128
HCLKidne	BisqueRN	sc-CMGAI	Dendritic_	400	-	-	0.066575	0.934392	0.067074	0.927562	0.067196	0.933187	0.068069	0.945347	0.067243	0.921006	0.069192	0.940083
HCLKidne	BisqueRN	sc-CMGAI	Distal_tub	400	-	-	0.080835	0.692772	0.086444	0.644163	0.090169	0.515325	0.083015	0.568049	0.085629	0.612844	0.088291	0.478888
HCLKidne	BisqueRN	sc-CMGAI	Distal_tub	400	-	-	0.075455	0.830038	0.081785	0.747371	0.084129	0.702112	0.078582	0.77869	0.083699	0.726219	0.083108	0.704499
HCLKidne	BisqueRN	sc-CMGAI	Endothelia	400	-	-	0.081923	0.837041	0.085727	0.785906	0.083383	0.710902	0.079333	0.851241	0.083766	0.840982	0.083167	0.839237
HCLKidne	BisqueRN	sc-CMGAI	Endothelia	400	-	-	0.095865	0.821968	0.08914	0.888673	0.092877	0.853798	0.091304	0.883656	0.094342	0.856599	0.090389	0.885974
HCLKidne	BisqueRN	sc-CMGAI	Intercalate	400	-	-	0.335018	0.831246	0.335432	0.853971	0.332533	0.793021	0.334355	0.848186	0.332021	0.847195	0.334877	0.843693
HCLKidne	BisqueRN	sc-CMGAI	Intercalate	400	-	-	0.08432	0.820943	0.074614	0.805326	0.075216	0.778689	0.086926	0.769609	0.075137	0.779783	0.080244	0.809849
HCLKidne	BisqueRN	sc-CMGAI	Loop_of_H	400	-	-	0.069725	0.946089	0.081052	0.78171	0.071507	0.832417	0.072447	0.919193	0.073698	0.903317	0.070241	0.936935
HCLKidne	BisqueRN	sc-CMGAI	Loop_of_h	400	-	-	0.077885	0.511803	0.076809	0.582199	0.082225	0.404415	0.079582	0.515491	0.079379	0.548724	0.07881	0.494807
HCLKidne	BisqueRN	sc-CMGAI	Loop_of_h	400	-	-	0.083987	0.668784	0.080187	0.665872	0.084347	0.553279	0.080837	0.679743	0.081445	0.622282	0.081405	0.701134
HCLKidne	BisqueRN	sc-CMGAI	Principle_	400	-	-	0.079069	0.676672	0.07974	0.639422	0.083166	0.567049	0.09155	0.413579	0.084531	0.549896	0.085075	0.551038
HCLKidne	BisqueRN	sc-CMGAI	Proximal_	400	-	-	0.073308	0.896231	0.072544	0.832673	0.076738	0.764632	0.072756	0.875659	0.076644	0.786597	0.070598	0.894928
HCLKidne	BisqueRN	sc-CMGAI	Proximal_	400	-	-	0.104221	0.429313	0.100197	0.49812	0.105527	0.371092	0.105727	0.372484	0.105646	0.396713	0.107959	0.330172
HCLKidne	BisqueRN	sc-CMGAI	Proximal_	400	-	-	0.092731	0.766413	0.095666	0.737801	0.097879	0.688754	0.099684	0.677645	0.094618	0.626884	0.097712	0.727648
HCLKidne	BisqueRN	sc-CMGAI	Smooth_m	400	-	-	0.088306	0.892478	0.086567	0.914203	0.088308	0.891884	0.088557	0.928324	0.089871	0.912169	0.087315	0.899922
HCLKidne	BisqueRN	sc-CMGAI	B_cell(Pla	500	-	-	0.082202	0.959116	0.082363	0.960487	0.081844	0.961041	0.082639	0.960021	0.082561	0.963642	0.081821	0.961685
HCLKidne	BisqueRN	sc-CMGAI	Dendritic_	500	-	-	0.066751	0.929223	0.067428	0.930642	0.066968	0.931472	0.067587	0.945351	0.067752	0.927899	0.068761	0.937634
HCLKidne	BisqueRN	sc-CMGAI	Distal_tub	500	-	-	0.080517	0.637309	0.087269	0.631174	0.089281	0.58031	0.081997	0.573554	0.08322	0.617067	0.087726	0.50093
HCLKidne	BisqueRN	sc-CMGAI	Distal_tub	500	-	-	0.077772	0.823056	0.081451	0.73829	0.08518	0.726954	0.079993	0.747206	0.087082	0.667693	0.082373	0.716594
HCLKidne	BisqueRN	sc-CMGAI	Endothelia	500	-	-	0.081914	0.839413	0.085541	0.789983	0.082329	0.739464	0.079965	0.845232	0.083246	0.823165	0.083988	0.828305
HCLKidne	BisqueRN	sc-CMGAI	Endothelia	500	-	-	0.09558	0.822703	0.089784	0.886123	0.092486	0.856924	0.090551	0.881935	0.094836	0.84613	0.091255	0.887002
HCLKidne	BisqueRN	sc-CMGAI	Intercalate	500	-	-	0.335045	0.835707	0.334672	0.85716	0.331146	0.792428	0.334518	0.845953	0.334558	0.81828	0.334332	0.848356
HCLKidne	BisqueRN	sc-CMGAI	Intercalate	500	-	-	0.084938	0.852466	0.077664	0.787966	0.074509	0.769687	0.087927	0.766497	0.077488	0.810065	0.078734	0.799777

HCLKidne	BisqueRN	sc-CMGAI	Loop_of_H	500	-	-	0.069567	0.944307	0.082353	0.786637	0.069521	0.834441	0.074429	0.910092	0.072516	0.909998	0.071437	0.928339
HCLKidne	BisqueRN	sc-CMGAI	Loop_of_h	500	-	-	0.077319	0.528482	0.07622	0.601682	0.083078	0.381325	0.078981	0.538299	0.078568	0.555417	0.078405	0.511486
HCLKidne	BisqueRN	sc-CMGAI	Loop_of_h	500	-	-	0.084405	0.670326	0.079954	0.681317	0.084409	0.550666	0.080743	0.677495	0.081	0.654037	0.081576	0.686742
HCLKidne	BisqueRN	sc-CMGAI	Principle_	500	-	-	0.078497	0.688271	0.083734	0.558236	0.083531	0.560265	0.092587	0.386937	0.083826	0.564633	0.086501	0.522447
HCLKidne	BisqueRN	sc-CMGAI	Proximal_	500	-	-	0.073353	0.891481	0.073043	0.83723	0.077007	0.748156	0.072113	0.872583	0.075605	0.825134	0.070939	0.896354
HCLKidne	BisqueRN	sc-CMGAI	Proximal_	500	-	-	0.104799	0.417722	0.10078	0.483951	0.104213	0.402947	0.103453	0.442915	0.105199	0.415603	0.107705	0.334411
HCLKidne	BisqueRN	sc-CMGAI	Proximal_	500	-	-	0.093081	0.766985	0.096922	0.741512	0.096839	0.719651	0.099115	0.667301	0.094755	0.650855	0.096692	0.74101
HCLKidne	BisqueRN	sc-CMGAI	Smooth_m	500	-	-	0.088065	0.89491	0.087088	0.90336	0.088174	0.883677	0.088336	0.92724	0.089418	0.906555	0.086896	0.896021
HCLKidne	BisqueRN	sc-CMGAI	B_cell(Pla	600	-	-	0.082375	0.959359	0.082136	0.959596	0.081706	0.960315	0.082474	0.960076	0.082502	0.963938	0.081814	0.961148
HCLKidne	BisqueRN	sc-CMGAI	Dendritic_	600	-	-	0.066133	0.93339	0.067588	0.928213	0.067155	0.930273	0.067491	0.944446	0.067594	0.926336	0.06928	0.94079
HCLKidne	BisqueRN	sc-CMGAI	Distal_tub	600	-	-	0.081746	0.601708	0.084942	0.625985	0.092171	0.467336	0.081928	0.57728	0.082821	0.600641	0.087949	0.485099
HCLKidne	BisqueRN	sc-CMGAI	Distal_tub	600	-	-	0.076006	0.84436	0.081325	0.756875	0.083588	0.718031	0.080154	0.757296	0.087667	0.664602	0.08185	0.726427
HCLKidne	BisqueRN	sc-CMGAI	Endothelia	600	-	-	0.082316	0.83232	0.085752	0.776664	0.082714	0.711033	0.0799	0.84091	0.08469	0.816807	0.083461	0.838092
HCLKidne	BisqueRN	sc-CMGAI	Endothelia	600	-	-	0.093325	0.82504	0.088999	0.887054	0.094226	0.846164	0.091434	0.88064	0.094382	0.819836	0.089874	0.887338
HCLKidne	BisqueRN	sc-CMGAI	Intercalate	600	-	-	0.335668	0.828464	0.33538	0.84256	0.333489	0.775685	0.33464	0.853363	0.333663	0.811452	0.334268	0.845111
HCLKidne	BisqueRN	sc-CMGAI	Intercalate	600	-	-	0.087493	0.853255	0.075658	0.809483	0.074881	0.779272	0.086475	0.780673	0.074511	0.811244	0.080985	0.809402
HCLKidne	BisqueRN	sc-CMGAI	Loop_of_H	600	-	-	0.070288	0.947143	0.083715	0.750734	0.071923	0.817369	0.074017	0.906782	0.073099	0.907674	0.071514	0.930087
HCLKidne	BisqueRN	sc-CMGAI	Loop_of_h	600	-	-	0.076577	0.548152	0.07634	0.587281	0.081034	0.442205	0.078024	0.561491	0.078635	0.560833	0.078143	0.51417
HCLKidne	BisqueRN	sc-CMGAI	Loop_of_h	600	-	-	0.083947	0.708464	0.080448	0.651457	0.084569	0.546631	0.081108	0.67465	0.082019	0.611826	0.08185	0.693408
HCLKidne	BisqueRN	sc-CMGAI	Principle_	600	-	-	0.078843	0.68747	0.08224	0.590328	0.081326	0.602539	0.091741	0.411844	0.084431	0.557236	0.085271	0.552695
HCLKidne	BisqueRN	sc-CMGAI	Proximal_	600	-	-	0.07304	0.896448	0.073323	0.811622	0.076985	0.749317	0.073256	0.873382	0.076859	0.779755	0.071076	0.887776
HCLKidne	BisqueRN	sc-CMGAI	Proximal_	600	-	-	0.105864	0.397054	0.101425	0.48159	0.104819	0.392343	0.103633	0.433258	0.105132	0.431227	0.107852	0.328647
HCLKidne	BisqueRN	sc-CMGAI	Proximal_	600	-	-	0.092859	0.778092	0.096917	0.726357	0.097302	0.714675	0.099444	0.660055	0.094369	0.642008	0.097208	0.73546
HCLKidne	BisqueRN	sc-CMGAI	Smooth_m	600	-	-	0.088705	0.883681	0.087121	0.901751	0.088616	0.878373	0.088435	0.925936	0.090136	0.911649	0.087114	0.891519
HCLKidne	BisqueRN	sc-CMGAI	B_cell(Pla	700	-	-	0.082196	0.959604	0.082261	0.96035	0.081507	0.959044	0.082628	0.960342	0.082497	0.964086	0.081617	0.960413
HCLKidne	BisqueRN	sc-CMGAI	Dendritic_	700	-	-	0.066719	0.93161	0.067213	0.927281	0.06736	0.92957	0.067848	0.944861	0.067525	0.929497	0.069379	0.937239
HCLKidne	BisqueRN	sc-CMGAI	Distal_tub	700	-	-	0.081153	0.587173	0.085362	0.639625	0.093307	0.407785	0.082895	0.558981	0.082329	0.610002	0.085773	0.513692
HCLKidne	BisqueRN	sc-CMGAI	Distal_tub	700	-	-	0.077907	0.827093	0.082409	0.726471	0.085356	0.707551	0.080074	0.749958	0.087522	0.656847	0.082756	0.697537
HCLKidne	BisqueRN	sc-CMGAI	Endothelia	700	-	-	0.081731	0.823476	0.085007	0.764328	0.083331	0.69306	0.079245	0.839673	0.083237	0.819405	0.08244	0.824208
HCLKidne	BisqueRN	sc-CMGAI	Endothelia	700	-	-	0.094161	0.815835	0.090093	0.886499	0.093982	0.846396	0.091364	0.879158	0.094837	0.830824	0.092031	0.877713
HCLKidne	BisqueRN	sc-CMGAI	Intercalate	700	-	-	0.33436	0.840806	0.335801	0.832133	0.334775	0.758304	0.334478	0.850455	0.335331	0.806608	0.3337	0.833279
HCLKidne	BisqueRN	sc-CMGAI	Intercalate	700	-	-	0.087266	0.852283	0.075562	0.818668	0.073962	0.803691	0.088797	0.748929	0.078129	0.822145	0.080104	0.80895
HCLKidne	BisqueRN	sc-CMGAI	Loop_of_H	700	-	-	0.070728	0.947567	0.08402	0.732515	0.070266	0.826061	0.074505	0.898441	0.072674	0.907039	0.072143	0.923052
HCLKidne	BisqueRN	sc-CMGAI	Loop_of_h	700	-	-	0.07662	0.548413	0.076124	0.593598	0.081181	0.433734	0.079664	0.516417	0.077962	0.580891	0.078948	0.485732
HCLKidne	BisqueRN	sc-CMGAI	Loop_of_h	700	-	-	0.083363	0.710807	0.080097	0.658032	0.084711	0.543398	0.080516	0.673169	0.081039	0.652325	0.08162	0.674551
HCLKidne	BisqueRN	sc-CMGAI	Principle_	700	-	-	0.079894	0.675386	0.081749	0.606522	0.080594	0.623313	0.090245	0.452472	0.084083	0.56469	0.087947	0.498545

HCLKidne	BisqueRN	sc-CMGAI	Proximal_	700	-	-	0.073255	0.893294	0.07383	0.827669	0.077309	0.740251	0.072503	0.870754	0.077012	0.795971	0.071201	0.890717
HCLKidne	BisqueRN	sc-CMGAI	Proximal_	700	-	-	0.105669	0.395426	0.100698	0.502241	0.10539	0.382799	0.105277	0.391129	0.105694	0.412252	0.110636	0.294404
HCLKidne	BisqueRN	sc-CMGAI	Proximal_	700	-	-	0.093978	0.770807	0.097064	0.720142	0.097533	0.715767	0.098598	0.663877	0.095123	0.64146	0.097384	0.739686
HCLKidne	BisqueRN	sc-CMGAI	Smooth_m	700	-	-	0.088494	0.888663	0.086955	0.895566	0.088874	0.87359	0.088405	0.926437	0.089759	0.905744	0.087765	0.874093
HCLKidne	BisqueRN	sc-CMGAI	B_cell(Pla	800	-	-	0.082275	0.959653	0.082052	0.959506	0.081592	0.959338	0.082572	0.95971	0.082493	0.963709	0.081535	0.960416
HCLKidne	BisqueRN	sc-CMGAI	Dendritic_	800	-	-	0.067131	0.9313	0.067178	0.928472	0.067266	0.926486	0.06805	0.944554	0.067789	0.926392	0.069221	0.939207
HCLKidne	BisqueRN	sc-CMGAI	Distal_tub	800	-	-	0.080591	0.625105	0.083158	0.640537	0.089268	0.577707	0.082017	0.563576	0.08195	0.609636	0.086703	0.499367
HCLKidne	BisqueRN	sc-CMGAI	Distal_tub	800	-	-	0.074759	0.838988	0.083563	0.72316	0.087851	0.661629	0.079771	0.755686	0.087042	0.668623	0.082406	0.719748
HCLKidne	BisqueRN	sc-CMGAI	Endothelia	800	-	-	0.081521	0.826834	0.084767	0.763199	0.082953	0.694163	0.079197	0.826713	0.083752	0.813172	0.082396	0.819832
HCLKidne	BisqueRN	sc-CMGAI	Endothelia	800	-	-	0.094431	0.793838	0.089081	0.887742	0.093724	0.849625	0.091232	0.881658	0.094766	0.832721	0.09267	0.875755
HCLKidne	BisqueRN	sc-CMGAI	Intercalate	800	-	-	0.335042	0.836007	0.336251	0.809959	0.335957	0.745708	0.334885	0.847388	0.333901	0.819118	0.333729	0.827615
HCLKidne	BisqueRN	sc-CMGAI	Intercalate	800	-	-	0.08846	0.835778	0.07561	0.828993	0.073948	0.80681	0.092582	0.718193	0.07724	0.818461	0.080681	0.810125
HCLKidne	BisqueRN	sc-CMGAI	Loop_of_H	800	-	-	0.071099	0.948118	0.086237	0.708088	0.072008	0.813943	0.075062	0.897807	0.073489	0.906937	0.071633	0.923735
HCLKidne	BisqueRN	sc-CMGAI	Loop_of_h	800	-	-	0.076904	0.536723	0.075912	0.600261	0.080125	0.46504	0.078938	0.5458	0.078091	0.577901	0.078471	0.497095
HCLKidne	BisqueRN	sc-CMGAI	Loop_of_h	800	-	-	0.084063	0.711744	0.079793	0.655194	0.084634	0.544985	0.080386	0.683806	0.081304	0.644196	0.081474	0.676367
HCLKidne	BisqueRN	sc-CMGAI	Principle_	800	-	-	0.079484	0.68633	0.083084	0.58662	0.08364	0.567394	0.091672	0.417952	0.084211	0.563934	0.087378	0.510145
HCLKidne	BisqueRN	sc-CMGAI	Proximal_	800	-	-	0.073444	0.893042	0.074799	0.788424	0.077553	0.735197	0.072328	0.872009	0.07697	0.788091	0.071262	0.88713
HCLKidne	BisqueRN	sc-CMGAI	Proximal_	800	-	-	0.107039	0.355042	0.10087	0.491431	0.103994	0.431825	0.10424	0.4258	0.106189	0.395865	0.110553	0.291367
HCLKidne	BisqueRN	sc-CMGAI	Proximal_	800	-	-	0.093197	0.777322	0.096969	0.725327	0.097002	0.728794	0.098725	0.667017	0.095441	0.612542	0.097224	0.736343
HCLKidne	BisqueRN	sc-CMGAI	Smooth_m	800	-	-	0.088224	0.881759	0.087128	0.894937	0.088754	0.87019	0.088529	0.925995	0.089893	0.906472	0.087551	0.877029
HCLKidne	BisqueRN	sc-CMGAI	B_cell(Pla	900	-	-	0.082328	0.959698	0.082215	0.959564	0.081583	0.958596	0.082493	0.959586	0.082439	0.96359	0.081721	0.960934
HCLKidne	BisqueRN	sc-CMGAI	Dendritic_	900	-	-	0.067163	0.929509	0.067279	0.926239	0.067628	0.926676	0.068165	0.945216	0.067545	0.928785	0.069706	0.939632
HCLKidne	BisqueRN	sc-CMGAI	Distal_tub	900	-	-	0.080086	0.623928	0.081626	0.644979	0.089331	0.572358	0.08245	0.552655	0.081904	0.607699	0.086757	0.49615
HCLKidne	BisqueRN	sc-CMGAI	Distal_tub	900	-	-	0.076636	0.838327	0.084574	0.709253	0.090424	0.632285	0.080116	0.741971	0.08863	0.636913	0.082786	0.700788
HCLKidne	BisqueRN	sc-CMGAI	Endothelia	900	-	-	0.08149	0.82599	0.084873	0.75469	0.083368	0.687745	0.079298	0.827193	0.083105	0.806851	0.082899	0.82334
HCLKidne	BisqueRN	sc-CMGAI	Endothelia	900	-	-	0.093841	0.786933	0.089489	0.8854	0.093825	0.848017	0.091097	0.878086	0.095119	0.813776	0.090486	0.882329
HCLKidne	BisqueRN	sc-CMGAI	Intercalate	900	-	-	0.334077	0.838295	0.336507	0.800615	0.336957	0.734213	0.335123	0.844321	0.336561	0.783851	0.333883	0.836237
HCLKidne	BisqueRN	sc-CMGAI	Intercalate	900	-	-	0.087695	0.839269	0.075552	0.8283	0.073741	0.806218	0.0928	0.717757	0.081067	0.824132	0.08245	0.806258
HCLKidne	BisqueRN	sc-CMGAI	Loop_of_H	900	-	-	0.070724	0.946656	0.08403	0.729365	0.071575	0.812198	0.07567	0.891408	0.072371	0.911274	0.071447	0.928035
HCLKidne	BisqueRN	sc-CMGAI	Loop_of_h	900	-	-	0.076909	0.533378	0.076768	0.581206	0.080591	0.451367	0.079108	0.539362	0.077701	0.589941	0.077394	0.530817
HCLKidne	BisqueRN	sc-CMGAI	Loop_of_h	900	-	-	0.083617	0.711628	0.079713	0.651396	0.084789	0.540825	0.080472	0.673389	0.081072	0.650639	0.081882	0.680731
HCLKidne	BisqueRN	sc-CMGAI	Principle_	900	-	-	0.079957	0.67346	0.083977	0.567628	0.080423	0.627372	0.089588	0.467516	0.085079	0.54614	0.088748	0.473072
HCLKidne	BisqueRN	sc-CMGAI	Proximal_	900	-	-	0.073813	0.890558	0.07359	0.808838	0.077729	0.725456	0.072222	0.874358	0.077413	0.782813	0.07099	0.892108
HCLKidne	BisqueRN	sc-CMGAI	Proximal_	900	-	-	0.10654	0.362616	0.101017	0.486634	0.105453	0.38406	0.10534	0.395397	0.105958	0.405719	0.108801	0.312992
HCLKidne	BisqueRN	sc-CMGAI	Proximal_	900	-	-	0.093497	0.781094	0.096002	0.74139	0.096833	0.738773	0.098276	0.667083	0.095063	0.645217	0.09698	0.74616
HCLKidne	BisqueRN	sc-CMGAI	Smooth_m	900	-	-	0.088383	0.879788	0.087317	0.888323	0.088848	0.869305	0.088541	0.92569	0.089853	0.899142	0.087327	0.878722

HCLKidne	BisqueRN	sc-CMGAI	B_cell(Pla	1000	-	-	0.082273	0.959797	0.082152	0.959448	0.081598	0.957831	0.082629	0.959984	0.08242	0.96369	0.081687	0.959848
HCLKidne	BisqueRN	sc-CMGAI	Dendritic_	1000	-	-	0.066903	0.927803	0.067775	0.926319	0.067608	0.926663	0.067979	0.945229	0.067835	0.923926	0.069345	0.937793
HCLKidne	BisqueRN	sc-CMGAI	Distal_tub	1000	-	-	0.081504	0.578098	0.082013	0.642719	0.091687	0.482022	0.081734	0.578248	0.081444	0.61254	0.086422	0.50212
HCLKidne	BisqueRN	sc-CMGAI	Distal_tub	1000	-	-	0.079059	0.815265	0.087844	0.652835	0.087708	0.67275	0.079939	0.744128	0.089849	0.603195	0.084671	0.664741
HCLKidne	BisqueRN	sc-CMGAI	Endothelia	1000	-	-	0.081338	0.824859	0.08503	0.748484	0.084035	0.665015	0.079098	0.828716	0.082287	0.812105	0.082299	0.821889
HCLKidne	BisqueRN	sc-CMGAI	Endothelia	1000	-	-	0.093429	0.794109	0.088808	0.884151	0.094068	0.846789	0.091132	0.880929	0.095169	0.820233	0.09093	0.887596
HCLKidne	BisqueRN	sc-CMGAI	Intercalate	1000	-	-	0.333616	0.84177	0.336989	0.793479	0.340818	0.706142	0.335048	0.85013	0.336499	0.774515	0.332831	0.839151
HCLKidne	BisqueRN	sc-CMGAI	Intercalate	1000	-	-	0.088704	0.827411	0.076186	0.819757	0.076627	0.794947	0.092699	0.713317	0.079399	0.81153	0.080449	0.806214
HCLKidne	BisqueRN	sc-CMGAI	Loop_of_H	1000	-	-	0.071148	0.945684	0.082442	0.739088	0.070587	0.819887	0.074813	0.905903	0.073355	0.906266	0.071585	0.925185
HCLKidne	BisqueRN	sc-CMGAI	Loop_of_h	1000	-	-	0.076831	0.538527	0.076297	0.593508	0.079353	0.498381	0.078587	0.557816	0.077373	0.611907	0.077779	0.521813
HCLKidne	BisqueRN	sc-CMGAI	Loop_of_h	1000	-	-	0.083451	0.709224	0.079976	0.643921	0.084715	0.543364	0.080357	0.685997	0.08182	0.631138	0.081428	0.680389
HCLKidne	BisqueRN	sc-CMGAI	Principle_	1000	-	-	0.079183	0.687712	0.084372	0.564236	0.083672	0.564994	0.090201	0.455877	0.085548	0.536865	0.088685	0.473791
HCLKidne	BisqueRN	sc-CMGAI	Proximal_	1000	-	-	0.073948	0.887881	0.074432	0.81033	0.078288	0.717239	0.072652	0.872473	0.077583	0.774336	0.07111	0.887375
HCLKidne	BisqueRN	sc-CMGAI	Proximal_	1000	-	-	0.107309	0.340157	0.102048	0.485246	0.105844	0.374788	0.104924	0.406568	0.106459	0.391302	0.109648	0.307823
HCLKidne	BisqueRN	sc-CMGAI	Proximal_	1000	-	-	0.093409	0.777553	0.09622	0.715352	0.097905	0.709416	0.097787	0.68317	0.095495	0.625553	0.096815	0.748371
HCLKidne	BisqueRN	sc-CMGAI	Smooth_m	1000	-	-	0.088718	0.875121	0.087365	0.886386	0.088881	0.866011	0.08847	0.926207	0.090056	0.889128	0.087312	0.881894
PBMCs	SCDC	Control	cell_1	0	0.041364	0.972381	-	-	-	-	-	-	-	-	-	-	-	-
PBMCs	SCDC	Control	cell_2	0	0.093243	0.943421	-	-	-	-	-	-	-	-	-	-	-	-
PBMCs	SCDC	Control	cell_3	0	0.121833	0.943603	-	-	-	-	-	-	-	-	-	-	-	-
PBMCs	SCDC	Control	cell_4	0	0.051764	0.974499	-	-	-	-	-	-	-	-	-	-	-	-
PBMCs	SCDC	Control	cell_6	0	0.048252	0.976697	-	-	-	-	-	-	-	-	-	-	-	-
PBMCs	SCDC	Control	cell_7	0	0.050966	0.97781	-	-	-	-	-	-	-	-	-	-	-	-
PBMCs	SCDC	sc-CMGAI	cell_1	100	-	-	0.047857	0.965681	0.046054	0.966008	0.048717	0.964901	0.045699	0.966749	0.045866	0.967031	0.047067	0.965571
PBMCs	SCDC	sc-CMGAI	cell_2	100	-	-	0.101817	0.929058	0.10684	0.936437	0.110172	0.938691	0.11927	0.908528	0.113305	0.935891	0.100568	0.927997
PBMCs	SCDC	sc-CMGAI	cell_3	100	-	-	0.08249	0.938844	0.085877	0.941637	0.087666	0.935943	0.093618	0.9222	0.093112	0.932369	0.083398	0.938679
PBMCs	SCDC	sc-CMGAI	cell_4	100	-	-	0.068916	0.963495	0.06665	0.968886	0.069262	0.967628	0.064195	0.970339	0.064589	0.96984	0.0695	0.964976
PBMCs	SCDC	sc-CMGAI	cell_6	100	-	-	0.048554	0.975863	0.047221	0.976574	0.051482	0.974955	0.046584	0.977177	0.052903	0.974257	0.049903	0.973285
PBMCs	SCDC	sc-CMGAI	cell_7	100	-	-	0.061549	0.969996	0.066185	0.967229	0.061665	0.970954	0.068034	0.965323	0.063997	0.968747	0.062085	0.972026
PBMCs	SCDC	sc-CMGAI	cell_1	200	-	-	0.046387	0.96602	0.04564	0.966809	0.047208	0.965196	0.045437	0.966887	0.045479	0.967173	0.047273	0.965556
PBMCs	SCDC	sc-CMGAI	cell_2	200	-	-	0.102084	0.933721	0.110672	0.937312	0.111212	0.938071	0.115017	0.910886	0.108338	0.936613	0.102921	0.926298
PBMCs	SCDC	sc-CMGAI	cell_3	200	-	-	0.084369	0.939043	0.091192	0.938443	0.089674	0.935315	0.089847	0.926085	0.090891	0.936972	0.084864	0.936534
PBMCs	SCDC	sc-CMGAI	cell_4	200	-	-	0.066633	0.966086	0.06555	0.968077	0.066636	0.969647	0.063922	0.971721	0.06206	0.97213	0.069325	0.965325
PBMCs	SCDC	sc-CMGAI	cell_6	200	-	-	0.048772	0.975445	0.046202	0.976621	0.051895	0.974503	0.048442	0.976161	0.052298	0.974488	0.04991	0.97376
PBMCs	SCDC	sc-CMGAI	cell_7	200	-	-	0.060891	0.970271	0.064877	0.968148	0.061279	0.97089	0.066258	0.966564	0.062822	0.969367	0.060744	0.97253
PBMCs	SCDC	sc-CMGAI	cell_1	300	-	-	0.047153	0.965028	0.045583	0.966848	0.046748	0.966223	0.045756	0.966596	0.045072	0.967751	0.046425	0.96641
PBMCs	SCDC	sc-CMGAI	cell_2	300	-	-	0.099156	0.936738	0.104059	0.939034	0.112247	0.941627	0.114957	0.912183	0.108463	0.934829	0.102005	0.926652

PBMCs	SCDC	sc-CMGAI cell_3	300	-	-	0.083996	0.939727	0.086851	0.942071	0.090735	0.935986	0.089276	0.926049	0.091617	0.937396	0.085729	0.937415
PBMCs	SCDC	sc-CMGAI cell_4	300	-	-	0.067338	0.965309	0.065432	0.968984	0.066075	0.969693	0.064285	0.971023	0.061461	0.97217	0.067158	0.966862
PBMCs	SCDC	sc-CMGAI cell_6	300	-	-	0.047695	0.975811	0.046169	0.976783	0.052587	0.974338	0.048768	0.976096	0.051268	0.974479	0.049045	0.974224
PBMCs	SCDC	sc-CMGAI cell_7	300	-	-	0.059531	0.971049	0.063498	0.969431	0.06113	0.97053	0.065867	0.96747	0.06302	0.969009	0.061848	0.972043
PBMCs	SCDC	sc-CMGAI cell_1	400	-	-	0.04628	0.965955	0.045377	0.96695	0.047052	0.965445	0.045293	0.967332	0.045235	0.967308	0.046722	0.965992
PBMCs	SCDC	sc-CMGAI cell_2	400	-	-	0.099319	0.934573	0.108553	0.93694	0.11011	0.941104	0.113	0.915199	0.111651	0.936067	0.101459	0.927658
PBMCs	SCDC	sc-CMGAI cell_3	400	-	-	0.083138	0.940009	0.089249	0.939103	0.091112	0.935993	0.090132	0.927388	0.093993	0.935268	0.08549	0.937637
PBMCs	SCDC	sc-CMGAI cell_4	400	-	-	0.066051	0.966127	0.065483	0.968624	0.065634	0.970542	0.063749	0.971542	0.06155	0.971876	0.067558	0.966498
PBMCs	SCDC	sc-CMGAI cell_6	400	-	-	0.047805	0.975894	0.046246	0.976753	0.051028	0.974924	0.047393	0.976547	0.051461	0.974515	0.048792	0.974303
PBMCs	SCDC	sc-CMGAI cell_7	400	-	-	0.06037	0.970629	0.065086	0.967564	0.060087	0.971766	0.065389	0.96816	0.063193	0.968598	0.061467	0.972369
PBMCs	SCDC	sc-CMGAI cell_1	500	-	-	0.046084	0.965911	0.045793	0.966567	0.047101	0.965843	0.045191	0.967506	0.045069	0.967689	0.046733	0.965907
PBMCs	SCDC	sc-CMGAI cell_2	500	-	-	0.100698	0.933132	0.109079	0.937153	0.113702	0.938597	0.112352	0.915147	0.108935	0.938278	0.101101	0.92797
PBMCs	SCDC	sc-CMGAI cell_3	500	-	-	0.084422	0.939165	0.090034	0.939211	0.091263	0.934724	0.088948	0.928278	0.092856	0.936355	0.083783	0.938429
PBMCs	SCDC	sc-CMGAI cell_4	500	-	-	0.06557	0.966868	0.065707	0.968718	0.06563	0.970079	0.06271	0.972364	0.061497	0.972015	0.067506	0.966549
PBMCs	SCDC	sc-CMGAI cell_6	500	-	-	0.047384	0.976169	0.046339	0.976809	0.052354	0.974474	0.047633	0.976766	0.051586	0.974326	0.048628	0.974522
PBMCs	SCDC	sc-CMGAI cell_7	500	-	-	0.060688	0.970656	0.064991	0.967807	0.061705	0.970351	0.065234	0.968021	0.062335	0.969527	0.06067	0.972729
PBMCs	SCDC	sc-CMGAI cell_1	600	-	-	0.046389	0.966144	0.045529	0.966713	0.046681	0.965967	0.045308	0.967363	0.045289	0.967365	0.046602	0.966181
PBMCs	SCDC	sc-CMGAI cell_2	600	-	-	0.099037	0.935449	0.107471	0.938514	0.108507	0.941301	0.113325	0.914523	0.10847	0.937904	0.101337	0.92734
PBMCs	SCDC	sc-CMGAI cell_3	600	-	-	0.083612	0.940595	0.088633	0.940642	0.090309	0.936975	0.089597	0.926298	0.091496	0.937863	0.084349	0.938147
PBMCs	SCDC	sc-CMGAI cell_4	600	-	-	0.066263	0.965826	0.065731	0.968481	0.065023	0.970415	0.063249	0.972556	0.061404	0.972397	0.067356	0.966458
PBMCs	SCDC	sc-CMGAI cell_6	600	-	-	0.047612	0.976091	0.045948	0.976849	0.050796	0.974996	0.048702	0.976071	0.051819	0.974215	0.048536	0.974631
PBMCs	SCDC	sc-CMGAI cell_7	600	-	-	0.059787	0.970877	0.064571	0.968058	0.059804	0.971965	0.064563	0.968022	0.062521	0.96923	0.060893	0.972503
PBMCs	SCDC	sc-CMGAI cell_1	700	-	-	0.046434	0.965739	0.045522	0.966727	0.046845	0.966305	0.045512	0.967278	0.045158	0.967586	0.046571	0.966176
PBMCs	SCDC	sc-CMGAI cell_2	700	-	-	0.099775	0.935388	0.107654	0.938405	0.111189	0.941262	0.11236	0.913242	0.107227	0.936549	0.10113	0.927928
PBMCs	SCDC	sc-CMGAI cell_3	700	-	-	0.0838	0.940227	0.088719	0.940122	0.090968	0.935479	0.089491	0.927877	0.09097	0.937761	0.085419	0.93807
PBMCs	SCDC	sc-CMGAI cell_4	700	-	-	0.066073	0.966173	0.065169	0.969191	0.066105	0.969447	0.063498	0.972585	0.061044	0.972376	0.067519	0.966428
PBMCs	SCDC	sc-CMGAI cell_6	700	-	-	0.047701	0.975842	0.046169	0.97671	0.052226	0.974301	0.047728	0.976619	0.051364	0.974345	0.048789	0.974409
PBMCs	SCDC	sc-CMGAI cell_7	700	-	-	0.060164	0.970641	0.064626	0.968063	0.060687	0.971151	0.065006	0.968309	0.061642	0.969856	0.060733	0.972478
PBMCs	SCDC	sc-CMGAI cell_1	800	-	-	0.04649	0.96557	0.045405	0.966921	0.046528	0.966158	0.045191	0.96764	0.045138	0.967517	0.046879	0.965755
PBMCs	SCDC	sc-CMGAI cell_2	800	-	-	0.09853	0.934564	0.104796	0.938095	0.10797	0.941439	0.112204	0.91511	0.110356	0.936348	0.100157	0.92815
PBMCs	SCDC	sc-CMGAI cell_3	800	-	-	0.083102	0.941353	0.086952	0.941537	0.088263	0.937484	0.089438	0.927507	0.093454	0.93586	0.083677	0.939517
PBMCs	SCDC	sc-CMGAI cell_4	800	-	-	0.066332	0.966272	0.06553	0.969081	0.065667	0.970523	0.06297	0.972684	0.06105	0.972451	0.067112	0.966374
PBMCs	SCDC	sc-CMGAI cell_6	800	-	-	0.048081	0.975379	0.046178	0.976792	0.052985	0.974149	0.047585	0.976669	0.051739	0.974208	0.048678	0.974533
PBMCs	SCDC	sc-CMGAI cell_7	800	-	-	0.059842	0.97092	0.063744	0.968737	0.059979	0.971927	0.064789	0.968042	0.062296	0.969094	0.06068	0.972833
PBMCs	SCDC	sc-CMGAI cell_1	900	-	-	0.046616	0.9654	0.045711	0.966564	0.046483	0.966215	0.045054	0.967692	0.045337	0.967288	0.046693	0.966031
PBMCs	SCDC	sc-CMGAI cell_2	900	-	-	0.099884	0.935634	0.10432	0.938787	0.111062	0.939266	0.113399	0.915488	0.107773	0.93773	0.101182	0.927976

PBMCs	SCDC	sc-CMGAI	cell_3	900	-	-	0.084405	0.939933	0.087657	0.941624	0.090768	0.936078	0.090009	0.926865	0.091194	0.93781	0.08451	0.938752
PBMCs	SCDC	sc-CMGAI	cell_4	900	-	-	0.06622	0.966424	0.064967	0.969866	0.065106	0.970826	0.063288	0.97225	0.061243	0.972086	0.067939	0.966196
PBMCs	SCDC	sc-CMGAI	cell_6	900	-	-	0.047809	0.975844	0.045176	0.977409	0.052368	0.974413	0.047634	0.976655	0.05187	0.974147	0.04892	0.974258
PBMCs	SCDC	sc-CMGAI	cell_7	900	-	-	0.060183	0.970576	0.063429	0.968981	0.060655	0.971231	0.064961	0.968108	0.062229	0.969488	0.060851	0.972455
PBMCs	SCDC	sc-CMGAI	cell_1	1000	-	-	0.046498	0.965842	0.04565	0.966558	0.046502	0.966272	0.045373	0.967364	0.045076	0.967486	0.046776	0.965704
PBMCs	SCDC	sc-CMGAI	cell_2	1000	-	-	0.100354	0.936222	0.107137	0.939016	0.10872	0.940381	0.11317	0.914348	0.107353	0.936438	0.100444	0.929069
PBMCs	SCDC	sc-CMGAI	cell_3	1000	-	-	0.084957	0.939126	0.088846	0.940706	0.089736	0.936733	0.089432	0.927377	0.091763	0.938006	0.084747	0.938764
PBMCs	SCDC	sc-CMGAI	cell_4	1000	-	-	0.066659	0.965911	0.065211	0.969445	0.064949	0.970777	0.063601	0.972338	0.060809	0.972285	0.067407	0.966524
PBMCs	SCDC	sc-CMGAI	cell_6	1000	-	-	0.048021	0.975587	0.045786	0.977117	0.051395	0.974705	0.048042	0.976561	0.051274	0.974391	0.048222	0.974793
PBMCs	SCDC	sc-CMGAI	cell_7	1000	-	-	0.060083	0.970745	0.064311	0.968408	0.060094	0.971678	0.065277	0.967741	0.061884	0.969626	0.060807	0.972663
PBMCs	MuSiC	Control	cell_1	0	0.043907	0.971909	-	-	-	-	-	-	-	-	-	-	-	-
PBMCs	MuSiC	Control	cell_2	0	0.0649	0.957132	-	-	-	-	-	-	-	-	-	-	-	-
PBMCs	MuSiC	Control	cell_3	0	0.067729	0.963587	-	-	-	-	-	-	-	-	-	-	-	-
PBMCs	MuSiC	Control	cell_4	0	0.055521	0.974585	-	-	-	-	-	-	-	-	-	-	-	-
PBMCs	MuSiC	Control	cell_6	0	0.046661	0.976979	-	-	-	-	-	-	-	-	-	-	-	-
PBMCs	MuSiC	Control	cell_7	0	0.047287	0.97859	-	-	-	-	-	-	-	-	-	-	-	-
PBMCs	MuSiC	sc-CMGAI	cell_1	100	-	-	0.050816	0.967962	0.05426	0.965915	0.049847	0.967308	0.053942	0.964802	0.052196	0.9661	0.050415	0.967284
PBMCs	MuSiC	sc-CMGAI	cell_2	100	-	-	0.095173	0.938464	0.092029	0.941206	0.084776	0.947266	0.113346	0.919433	0.094891	0.932632	0.102857	0.936542
PBMCs	MuSiC	sc-CMGAI	cell_3	100	-	-	0.06696	0.958801	0.063283	0.959334	0.064311	0.958271	0.073067	0.95141	0.066703	0.95523	0.068499	0.95846
PBMCs	MuSiC	sc-CMGAI	cell_4	100	-	-	0.067039	0.975075	0.073655	0.973661	0.068282	0.974452	0.075058	0.971419	0.070481	0.97378	0.065568	0.975175
PBMCs	MuSiC	sc-CMGAI	cell_6	100	-	-	0.0445	0.977355	0.042817	0.978659	0.044833	0.977041	0.042948	0.978559	0.045737	0.976236	0.045325	0.976271
PBMCs	MuSiC	sc-CMGAI	cell_7	100	-	-	0.052535	0.974326	0.055319	0.972659	0.05235	0.974923	0.059564	0.96881	0.055724	0.972311	0.052228	0.975445
PBMCs	MuSiC	sc-CMGAI	cell_1	200	-	-	0.050949	0.968169	0.052472	0.967533	0.050249	0.966812	0.054568	0.964956	0.052023	0.966749	0.050093	0.967405
PBMCs	MuSiC	sc-CMGAI	cell_2	200	-	-	0.097882	0.937713	0.088987	0.940432	0.086533	0.945771	0.110079	0.920494	0.095967	0.931009	0.101442	0.936236
PBMCs	MuSiC	sc-CMGAI	cell_3	200	-	-	0.067786	0.956937	0.062879	0.95948	0.063401	0.95968	0.071802	0.953719	0.067045	0.955229	0.068829	0.958374
PBMCs	MuSiC	sc-CMGAI	cell_4	200	-	-	0.066457	0.975403	0.069246	0.974542	0.06994	0.97415	0.075376	0.971133	0.06993	0.97333	0.066386	0.974833
PBMCs	MuSiC	sc-CMGAI	cell_6	200	-	-	0.044824	0.977204	0.0427	0.978689	0.045012	0.977025	0.043724	0.977986	0.045574	0.976282	0.044764	0.976807
PBMCs	MuSiC	sc-CMGAI	cell_7	200	-	-	0.051514	0.974808	0.056451	0.972361	0.052271	0.975012	0.058754	0.968912	0.054411	0.97306	0.051908	0.975082
PBMCs	MuSiC	sc-CMGAI	cell_1	300	-	-	0.051489	0.967612	0.052304	0.967261	0.049444	0.967547	0.053203	0.965779	0.052163	0.966599	0.049924	0.967786
PBMCs	MuSiC	sc-CMGAI	cell_2	300	-	-	0.095393	0.941256	0.088	0.942521	0.084752	0.945817	0.112611	0.921291	0.096966	0.930478	0.1033	0.935098
PBMCs	MuSiC	sc-CMGAI	cell_3	300	-	-	0.066154	0.959597	0.062671	0.959784	0.064001	0.958498	0.07371	0.952556	0.067626	0.955057	0.070061	0.957919
PBMCs	MuSiC	sc-CMGAI	cell_4	300	-	-	0.066516	0.975137	0.070339	0.974241	0.068678	0.974152	0.073074	0.972336	0.069314	0.974184	0.066076	0.974927
PBMCs	MuSiC	sc-CMGAI	cell_6	300	-	-	0.04489	0.977113	0.042712	0.978689	0.045341	0.976692	0.043504	0.978043	0.045601	0.976228	0.044873	0.976697
PBMCs	MuSiC	sc-CMGAI	cell_7	300	-	-	0.050945	0.975287	0.055594	0.973182	0.052772	0.974649	0.05738	0.970329	0.054545	0.972773	0.052817	0.974991
PBMCs	MuSiC	sc-CMGAI	cell_1	400	-	-	0.050773	0.968114	0.052398	0.967155	0.049808	0.967161	0.053735	0.965599	0.052204	0.966807	0.049894	0.967714
PBMCs	MuSiC	sc-CMGAI	cell_2	400	-	-	0.096523	0.940274	0.089649	0.939911	0.083613	0.948027	0.111733	0.923626	0.094874	0.931104	0.102232	0.936201

PBMCs	MuSiC	sc-CMGAI cell_3	400	-	-	0.066205	0.959391	0.063117	0.959154	0.063366	0.959199	0.07199	0.953377	0.067854	0.954029	0.06925	0.95823
PBMCs	MuSiC	sc-CMGAI cell_4	400	-	-	0.06588	0.975094	0.070557	0.974195	0.069231	0.973964	0.07393	0.97191	0.06918	0.97395	0.065386	0.975107
PBMCs	MuSiC	sc-CMGAI cell_6	400	-	-	0.044688	0.977382	0.042926	0.978526	0.044851	0.977268	0.043331	0.978148	0.045572	0.976352	0.044966	0.97663
PBMCs	MuSiC	sc-CMGAI cell_7	400	-	-	0.0515	0.974906	0.057296	0.971829	0.052157	0.97498	0.056235	0.971408	0.054972	0.972538	0.052093	0.975146
PBMCs	MuSiC	sc-CMGAI cell_1	500	-	-	0.051318	0.967895	0.052594	0.967208	0.049523	0.967294	0.053374	0.96535	0.051854	0.967002	0.050313	0.967548
PBMCs	MuSiC	sc-CMGAI cell_2	500	-	-	0.096403	0.939023	0.090546	0.938959	0.085205	0.945719	0.112212	0.923255	0.095551	0.931689	0.102488	0.936154
PBMCs	MuSiC	sc-CMGAI cell_3	500	-	-	0.066737	0.95815	0.063115	0.959219	0.063994	0.958349	0.072224	0.953731	0.066824	0.955464	0.06915	0.958502
PBMCs	MuSiC	sc-CMGAI cell_4	500	-	-	0.06637	0.975281	0.070741	0.974207	0.068582	0.974306	0.074228	0.971867	0.068956	0.973751	0.066175	0.974824
PBMCs	MuSiC	sc-CMGAI cell_6	500	-	-	0.044342	0.977616	0.04266	0.978731	0.045058	0.976987	0.043174	0.978332	0.045473	0.976384	0.044648	0.976916
PBMCs	MuSiC	sc-CMGAI cell_7	500	-	-	0.051825	0.974565	0.05709	0.97178	0.052985	0.974382	0.057232	0.97047	0.054562	0.972916	0.052087	0.97494
PBMCs	MuSiC	sc-CMGAI cell_1	600	-	-	0.050913	0.967952	0.052497	0.967454	0.04946	0.967472	0.053238	0.965776	0.051589	0.96703	0.049973	0.96762
PBMCs	MuSiC	sc-CMGAI cell_2	600	-	-	0.095976	0.939992	0.089459	0.940031	0.084241	0.947017	0.112232	0.921121	0.09565	0.931494	0.103639	0.936007
PBMCs	MuSiC	sc-CMGAI cell_3	600	-	-	0.066129	0.959189	0.062979	0.959378	0.063088	0.959704	0.073071	0.953217	0.06724	0.955277	0.069601	0.958531
PBMCs	MuSiC	sc-CMGAI cell_4	600	-	-	0.065858	0.97522	0.069953	0.974336	0.068132	0.974101	0.073717	0.972237	0.068758	0.973982	0.065929	0.974973
PBMCs	MuSiC	sc-CMGAI cell_6	600	-	-	0.044476	0.977501	0.042708	0.978669	0.044531	0.977393	0.043584	0.978044	0.045669	0.976268	0.044638	0.976852
PBMCs	MuSiC	sc-CMGAI cell_7	600	-	-	0.050919	0.975202	0.056589	0.972039	0.052255	0.974807	0.057108	0.970509	0.05433	0.97303	0.052188	0.975051
PBMCs	MuSiC	sc-CMGAI cell_1	700	-	-	0.050835	0.968156	0.052382	0.96733	0.049435	0.967744	0.05376	0.965434	0.05187	0.967039	0.050111	0.967735
PBMCs	MuSiC	sc-CMGAI cell_2	700	-	-	0.094695	0.940761	0.089771	0.94071	0.083329	0.947371	0.112536	0.922833	0.094557	0.932481	0.103308	0.935325
PBMCs	MuSiC	sc-CMGAI cell_3	700	-	-	0.066033	0.958921	0.063255	0.959019	0.063559	0.958934	0.072642	0.953551	0.067012	0.955101	0.069608	0.958273
PBMCs	MuSiC	sc-CMGAI cell_4	700	-	-	0.065417	0.975065	0.07032	0.974166	0.06859	0.97402	0.074781	0.971728	0.068777	0.973796	0.066071	0.97494
PBMCs	MuSiC	sc-CMGAI cell_6	700	-	-	0.044368	0.977568	0.042742	0.978684	0.044883	0.977138	0.043413	0.978098	0.045452	0.97641	0.044714	0.976817
PBMCs	MuSiC	sc-CMGAI cell_7	700	-	-	0.051503	0.974845	0.056533	0.9725	0.052624	0.974645	0.057239	0.970487	0.05394	0.973395	0.051939	0.975238
PBMCs	MuSiC	sc-CMGAI cell_1	800	-	-	0.051069	0.967967	0.052725	0.967335	0.049549	0.96748	0.053572	0.965536	0.051758	0.967046	0.049665	0.967875
PBMCs	MuSiC	sc-CMGAI cell_2	800	-	-	0.0947	0.940997	0.090422	0.93985	0.084614	0.94709	0.110471	0.923533	0.095454	0.930985	0.102729	0.936913
PBMCs	MuSiC	sc-CMGAI cell_3	800	-	-	0.066019	0.959172	0.062592	0.960027	0.063593	0.959033	0.07189	0.953567	0.067287	0.95511	0.069285	0.958766
PBMCs	MuSiC	sc-CMGAI cell_4	800	-	-	0.066019	0.97515	0.070495	0.97418	0.06908	0.974252	0.074362	0.971723	0.068479	0.974089	0.065585	0.974687
PBMCs	MuSiC	sc-CMGAI cell_6	800	-	-	0.044775	0.977124	0.042677	0.978725	0.0453	0.976821	0.043233	0.978264	0.045659	0.97623	0.044671	0.976924
PBMCs	MuSiC	sc-CMGAI cell_7	800	-	-	0.051321	0.974934	0.056106	0.972534	0.052081	0.974999	0.057285	0.970565	0.054466	0.972822	0.051972	0.975274
PBMCs	MuSiC	sc-CMGAI cell_1	900	-	-	0.051519	0.967607	0.052733	0.967193	0.049616	0.967504	0.053359	0.965706	0.051516	0.967188	0.050036	0.967672
PBMCs	MuSiC	sc-CMGAI cell_2	900	-	-	0.094837	0.940554	0.09053	0.940962	0.08357	0.947908	0.111479	0.924014	0.095616	0.931692	0.103859	0.935797
PBMCs	MuSiC	sc-CMGAI cell_3	900	-	-	0.066267	0.958383	0.062747	0.959803	0.063619	0.958956	0.072353	0.953752	0.067325	0.954919	0.069726	0.958723
PBMCs	MuSiC	sc-CMGAI cell_4	900	-	-	0.066922	0.97517	0.070219	0.974306	0.068361	0.974392	0.074115	0.971859	0.068504	0.974034	0.066206	0.974651
PBMCs	MuSiC	sc-CMGAI cell_6	900	-	-	0.04443	0.977546	0.042624	0.978832	0.044993	0.977016	0.043116	0.978372	0.045694	0.976196	0.044771	0.976727
PBMCs	MuSiC	sc-CMGAI cell_7	900	-	-	0.05165	0.974708	0.055889	0.972652	0.052468	0.974778	0.057029	0.970563	0.05434	0.973037	0.05194	0.975276
PBMCs	MuSiC	sc-CMGAI cell_1	1000	-	-	0.051163	0.967816	0.052399	0.967418	0.049199	0.967803	0.053722	0.965336	0.052134	0.966824	0.049914	0.967834
PBMCs	MuSiC	sc-CMGAI cell_2	1000	-	-	0.094427	0.941211	0.088956	0.941024	0.083674	0.947377	0.110815	0.92398	0.096084	0.930968	0.102162	0.936378

PBMCs	MuSiC	sc-CMGAI	cell_3	1000	-	-	0.065972	0.958703	0.062496	0.959856	0.063108	0.959561	0.071706	0.95399	0.067218	0.955439	0.069053	0.95855
PBMCs	MuSiC	sc-CMGAI	cell_4	1000	-	-	0.066491	0.975049	0.070405	0.974244	0.068036	0.974294	0.074858	0.971662	0.068713	0.973986	0.065753	0.974922
PBMCs	MuSiC	sc-CMGAI	cell_6	1000	-	-	0.044595	0.977392	0.042687	0.978714	0.044782	0.977211	0.043379	0.978181	0.04569	0.976175	0.044668	0.97683
PBMCs	MuSiC	sc-CMGAI	cell_7	1000	-	-	0.051367	0.974919	0.056518	0.972237	0.052499	0.974778	0.057322	0.970545	0.054167	0.973109	0.052106	0.975094
PBMCs	BisqueRN	Control	cell_1	0	0.203851	0.956525	-	-	-	-	-	-	-	-	-	-	-	-
PBMCs	BisqueRN	Control	cell_2	0	0.24549	0.709101	-	-	-	-	-	-	-	-	-	-	-	-
PBMCs	BisqueRN	Control	cell_3	0	0.356833	0.747027	-	-	-	-	-	-	-	-	-	-	-	-
PBMCs	BisqueRN	Control	cell_4	0	0.235744	0.930792	-	-	-	-	-	-	-	-	-	-	-	-
PBMCs	BisqueRN	Control	cell_6	0	0.22754	0.959776	-	-	-	-	-	-	-	-	-	-	-	-
PBMCs	BisqueRN	Control	cell_7	0	0.222654	0.881013	-	-	-	-	-	-	-	-	-	-	-	-
PBMCs	BisqueRN	sc-CMGAI	cell_1	100	-	-	0.125913	0.944656	0.125709	0.935935	0.125115	0.929027	0.126335	0.932906	0.12695	0.927128	0.12543	0.936096
PBMCs	BisqueRN	sc-CMGAI	cell_2	100	-	-	0.198936	0.625695	0.196316	0.667069	0.200461	0.583483	0.199254	0.605142	0.197383	0.635838	0.196897	0.642964
PBMCs	BisqueRN	sc-CMGAI	cell_3	100	-	-	0.232246	0.865301	0.234816	0.860039	0.235031	0.862299	0.232505	0.871471	0.23888	0.836552	0.23425	0.885822
PBMCs	BisqueRN	sc-CMGAI	cell_4	100	-	-	0.181603	0.913085	0.184375	0.914104	0.182934	0.930798	0.180998	0.941928	0.183637	0.906948	0.182265	0.919639
PBMCs	BisqueRN	sc-CMGAI	cell_6	100	-	-	0.142661	0.959829	0.143227	0.963584	0.148032	0.955188	0.144226	0.965661	0.147335	0.957393	0.148231	0.952783
PBMCs	BisqueRN	sc-CMGAI	cell_7	100	-	-	0.199279	0.910254	0.196691	0.915248	0.198828	0.923501	0.199072	0.906029	0.195436	0.929562	0.199768	0.913638
PBMCs	BisqueRN	sc-CMGAI	cell_1	200	-	-	0.126669	0.942764	0.126039	0.934527	0.126601	0.921485	0.127404	0.924811	0.127735	0.918946	0.126599	0.931126
PBMCs	BisqueRN	sc-CMGAI	cell_2	200	-	-	0.202506	0.597773	0.20246	0.580746	0.205581	0.5481	0.2053	0.543517	0.199898	0.625889	0.200964	0.589586
PBMCs	BisqueRN	sc-CMGAI	cell_3	200	-	-	0.235447	0.846938	0.239532	0.818544	0.237365	0.851325	0.235377	0.850021	0.241747	0.825251	0.234645	0.889869
PBMCs	BisqueRN	sc-CMGAI	cell_4	200	-	-	0.181775	0.917229	0.186863	0.894068	0.185465	0.922208	0.184789	0.938426	0.186978	0.896178	0.184501	0.913644
PBMCs	BisqueRN	sc-CMGAI	cell_6	200	-	-	0.146762	0.959536	0.145721	0.962369	0.153384	0.950245	0.147239	0.964256	0.150084	0.956891	0.151127	0.952353
PBMCs	BisqueRN	sc-CMGAI	cell_7	200	-	-	0.201174	0.904531	0.199045	0.912926	0.201259	0.920287	0.200518	0.905326	0.196181	0.934236	0.203376	0.899129
PBMCs	BisqueRN	sc-CMGAI	cell_1	300	-	-	0.128712	0.937185	0.126492	0.930533	0.126153	0.921984	0.127654	0.919317	0.129007	0.913979	0.126941	0.929615
PBMCs	BisqueRN	sc-CMGAI	cell_2	300	-	-	0.204398	0.612077	0.204558	0.560528	0.208383	0.524966	0.209086	0.503377	0.200992	0.643631	0.202509	0.601798
PBMCs	BisqueRN	sc-CMGAI	cell_3	300	-	-	0.236668	0.836633	0.239552	0.823661	0.240573	0.830661	0.237689	0.835308	0.244791	0.813717	0.237572	0.877952
PBMCs	BisqueRN	sc-CMGAI	cell_4	300	-	-	0.183308	0.908168	0.188311	0.892354	0.1864	0.918884	0.187229	0.931137	0.188066	0.884625	0.184708	0.915374
PBMCs	BisqueRN	sc-CMGAI	cell_6	300	-	-	0.14657	0.960325	0.147651	0.961562	0.15558	0.946843	0.148916	0.963093	0.151996	0.956568	0.152642	0.951725
PBMCs	BisqueRN	sc-CMGAI	cell_7	300	-	-	0.201291	0.914488	0.200782	0.920141	0.200089	0.926955	0.201609	0.90928	0.196781	0.934245	0.204039	0.908742
PBMCs	BisqueRN	sc-CMGAI	cell_1	400	-	-	0.12817	0.937588	0.127618	0.926354	0.127145	0.918526	0.127599	0.919515	0.129651	0.912245	0.128134	0.927316
PBMCs	BisqueRN	sc-CMGAI	cell_2	400	-	-	0.205966	0.605002	0.206763	0.54747	0.2093	0.54061	0.211036	0.477307	0.201906	0.654422	0.202951	0.626563
PBMCs	BisqueRN	sc-CMGAI	cell_3	400	-	-	0.238153	0.830757	0.243311	0.790696	0.241131	0.826695	0.238325	0.833684	0.246248	0.81433	0.238969	0.865794
PBMCs	BisqueRN	sc-CMGAI	cell_4	400	-	-	0.183372	0.91068	0.189758	0.874164	0.18723	0.914728	0.187416	0.932632	0.187984	0.883598	0.185249	0.909505
PBMCs	BisqueRN	sc-CMGAI	cell_6	400	-	-	0.14619	0.960623	0.146293	0.961421	0.156031	0.945221	0.149657	0.96264	0.153414	0.956743	0.153709	0.950323
PBMCs	BisqueRN	sc-CMGAI	cell_7	400	-	-	0.20174	0.918924	0.200609	0.916825	0.200816	0.930646	0.201881	0.907936	0.197263	0.931126	0.203492	0.91292
PBMCs	BisqueRN	sc-CMGAI	cell_1	500	-	-	0.128863	0.935283	0.127511	0.926138	0.127681	0.916862	0.128618	0.916848	0.129575	0.910918	0.128906	0.924371
PBMCs	BisqueRN	sc-CMGAI	cell_2	500	-	-	0.207854	0.613619	0.206758	0.550081	0.210552	0.536907	0.211899	0.474509	0.202243	0.666091	0.204352	0.593035

PBMCs	BisqueRN	sc-CMGAI	cell_3	500	-	-	0.238897	0.829399	0.243265	0.799033	0.241724	0.824702	0.239609	0.826432	0.246542	0.818244	0.239296	0.862442
PBMCs	BisqueRN	sc-CMGAI	cell_4	500	-	-	0.183872	0.909327	0.18949	0.878628	0.187862	0.906871	0.188099	0.923237	0.188428	0.886817	0.185746	0.903026
PBMCs	BisqueRN	sc-CMGAI	cell_6	500	-	-	0.147295	0.961344	0.14777	0.961633	0.157502	0.942924	0.149127	0.963595	0.154019	0.955415	0.15336	0.951934
PBMCs	BisqueRN	sc-CMGAI	cell_7	500	-	-	0.201786	0.920502	0.201359	0.91103	0.201131	0.921135	0.201329	0.89513	0.197538	0.932277	0.204347	0.899675
PBMCs	BisqueRN	sc-CMGAI	cell_1	600	-	-	0.128437	0.935541	0.128152	0.924501	0.127705	0.915684	0.128249	0.918847	0.130166	0.908933	0.12868	0.923906
PBMCs	BisqueRN	sc-CMGAI	cell_2	600	-	-	0.207631	0.634036	0.206288	0.576601	0.210948	0.560468	0.212911	0.465124	0.202171	0.68483	0.204629	0.61341
PBMCs	BisqueRN	sc-CMGAI	cell_3	600	-	-	0.240788	0.821095	0.243877	0.800974	0.242717	0.815523	0.24092	0.827507	0.247118	0.82347	0.240742	0.852384
PBMCs	BisqueRN	sc-CMGAI	cell_4	600	-	-	0.183381	0.911464	0.19002	0.869562	0.188224	0.905063	0.187858	0.927044	0.188995	0.879304	0.185546	0.906083
PBMCs	BisqueRN	sc-CMGAI	cell_6	600	-	-	0.148011	0.961074	0.147334	0.960768	0.156717	0.944342	0.151645	0.96076	0.155363	0.954163	0.153759	0.949716
PBMCs	BisqueRN	sc-CMGAI	cell_7	600	-	-	0.201933	0.915298	0.200485	0.913448	0.200519	0.929445	0.201425	0.893265	0.196758	0.932525	0.203874	0.907663
PBMCs	BisqueRN	sc-CMGAI	cell_1	700	-	-	0.129489	0.93325	0.127576	0.925123	0.127916	0.916328	0.128352	0.917288	0.129854	0.908877	0.128542	0.922979
PBMCs	BisqueRN	sc-CMGAI	cell_2	700	-	-	0.209253	0.630953	0.207341	0.557747	0.211303	0.570422	0.213363	0.462708	0.202458	0.691856	0.204335	0.643514
PBMCs	BisqueRN	sc-CMGAI	cell_3	700	-	-	0.241519	0.81331	0.242798	0.808956	0.24323	0.813257	0.242303	0.819882	0.247782	0.821271	0.242545	0.83784
PBMCs	BisqueRN	sc-CMGAI	cell_4	700	-	-	0.183518	0.905631	0.190129	0.874479	0.188158	0.90327	0.188612	0.92217	0.188936	0.882153	0.185151	0.908192
PBMCs	BisqueRN	sc-CMGAI	cell_6	700	-	-	0.148649	0.959742	0.147523	0.960727	0.157984	0.941653	0.150755	0.961559	0.155485	0.954284	0.153658	0.949204
PBMCs	BisqueRN	sc-CMGAI	cell_7	700	-	-	0.200893	0.920891	0.200628	0.915375	0.200308	0.924477	0.20176	0.891807	0.196703	0.929875	0.202705	0.909147
PBMCs	BisqueRN	sc-CMGAI	cell_1	800	-	-	0.129402	0.932949	0.128077	0.923881	0.128392	0.916294	0.128596	0.915937	0.130173	0.90931	0.129129	0.921294
PBMCs	BisqueRN	sc-CMGAI	cell_2	800	-	-	0.209411	0.639341	0.206008	0.593748	0.211413	0.594739	0.213968	0.4584	0.203037	0.689381	0.206052	0.631258
PBMCs	BisqueRN	sc-CMGAI	cell_3	800	-	-	0.241817	0.814594	0.244655	0.800742	0.243104	0.812168	0.242789	0.816252	0.249023	0.814378	0.243225	0.827476
PBMCs	BisqueRN	sc-CMGAI	cell_4	800	-	-	0.183282	0.906595	0.190247	0.872127	0.188175	0.899206	0.189252	0.917305	0.188127	0.88241	0.185488	0.903566
PBMCs	BisqueRN	sc-CMGAI	cell_6	800	-	-	0.149398	0.959479	0.147234	0.961065	0.159058	0.938032	0.151114	0.960688	0.156732	0.953307	0.154513	0.948028
PBMCs	BisqueRN	sc-CMGAI	cell_7	800	-	-	0.20144	0.914341	0.201032	0.914238	0.20063	0.926087	0.201613	0.888163	0.19661	0.929787	0.202778	0.910688
PBMCs	BisqueRN	sc-CMGAI	cell_1	900	-	-	0.129655	0.930815	0.128489	0.921719	0.128501	0.915168	0.128664	0.914836	0.130072	0.909993	0.129321	0.920684
PBMCs	BisqueRN	sc-CMGAI	cell_2	900	-	-	0.209933	0.647998	0.206496	0.59259	0.212484	0.578913	0.214016	0.464436	0.203452	0.692587	0.206158	0.62374
PBMCs	BisqueRN	sc-CMGAI	cell_3	900	-	-	0.242852	0.805596	0.244192	0.801351	0.244028	0.804669	0.243555	0.815645	0.248383	0.822491	0.243447	0.825961
PBMCs	BisqueRN	sc-CMGAI	cell_4	900	-	-	0.183573	0.907772	0.190548	0.867845	0.188706	0.890737	0.18982	0.911757	0.188385	0.883235	0.185581	0.898072
PBMCs	BisqueRN	sc-CMGAI	cell_6	900	-	-	0.14873	0.96082	0.146434	0.961806	0.159236	0.937641	0.151147	0.96055	0.15697	0.952986	0.154139	0.948131
PBMCs	BisqueRN	sc-CMGAI	cell_7	900	-	-	0.201087	0.912085	0.200614	0.916591	0.200598	0.925767	0.201028	0.88801	0.196899	0.929973	0.20286	0.910528
PBMCs	BisqueRN	sc-CMGAI	cell_1	1000	-	-	0.129467	0.932435	0.128324	0.92221	0.1285	0.914502	0.12879	0.916027	0.130238	0.90973	0.129454	0.919662
PBMCs	BisqueRN	sc-CMGAI	cell_2	1000	-	-	0.210989	0.6211	0.206987	0.577297	0.212262	0.59287	0.214769	0.453688	0.203287	0.697573	0.206971	0.635518
PBMCs	BisqueRN	sc-CMGAI	cell_3	1000	-	-	0.242392	0.809623	0.244437	0.800048	0.243648	0.806443	0.243842	0.812478	0.249608	0.815663	0.244198	0.815099
PBMCs	BisqueRN	sc-CMGAI	cell_4	1000	-	-	0.183341	0.906075	0.190378	0.866019	0.189179	0.88878	0.189175	0.915584	0.188197	0.881369	0.185613	0.89944
PBMCs	BisqueRN	sc-CMGAI	cell_6	1000	-	-	0.149223	0.959809	0.146055	0.961548	0.158692	0.937542	0.152264	0.958998	0.157035	0.952987	0.153932	0.948228
PBMCs	BisqueRN	sc-CMGAI	cell_7	1000	-	-	0.201045	0.914094	0.200653	0.910502	0.20014	0.928108	0.201245	0.884454	0.196422	0.928015	0.202019	0.916119
